# Silver(I) triflate-catalyzed post-Ugi synthesis of pyrazolodiazepines

**DOI:** 10.3762/bjoc.21.74

**Published:** 2025-05-08

**Authors:** Muhammad Hasan, Anatoly A Peshkov, Syed Anis Ali Shah, Andrey Belyaev, Chang-Keun Lim, Shunyi Wang, Vsevolod A Peshkov

**Affiliations:** 1 College of Chemistry Chemical Engineering and Material Science, Soochow University, Suzhou, 215123, P.R. China; 2 Department of Chemistry, School of Sciences and Humanities, Nazarbayev University, Astana, 010000, Kazakhstan; 3 Department of Chemistry, University of Eastern Finland, FI-80101 Joensuu, Finland; 4 Department of Chemical and Materials Engineering, School of Engineering and Digital Sciences, Nazarbayev University, Astana, 010000, Kazakhstan

**Keywords:** heterocycles, post-MCR transformations, pyrazolodiazepines, silver catalysis, Ugi reaction

## Abstract

A silver(I) triflate-catalyzed post-Ugi assembly of novel pyrazolo[1,5-*a*][1,4]diazepine scaffolds is reported offering high yields (up to 98%) under mild conditions. The synthetic sequence involves the Ugi four-component reaction (U4CR) of pyrazole-3-carbaldehydes, primary amines, 3-substituted propiolic acids, and isocyanides, followed by a silver(I) triflate-catalyzed intramolecular heteroannulation of the resulting pyrazole-tethered propargylamides occurring in a 7-*endo*-*dig* fashion. The approach is scalable and tolerates a diverse range of substitution patterns.

## Introduction

Synthetic chemists are continuously involved in the development of methodologies for accessing new heterocyclic scaffolds that resemble naturally occurring products and biologically active molecules [1–2]. Nitrogen heterocycles draw particular attention due to their modular hydrogen bond donor/acceptor profile that can be tuned by varying the nitrogen position and the degree of unsaturation as well as by fusion with other rings [3].

**Figure 1:**
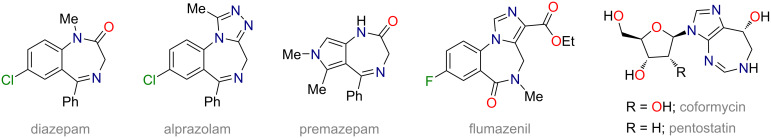
Representative diazepine-fused heterocycles.

Benzodiazepines, which contain a seven-membered diazepine core fused to a benzene ring, are recognized as privileged scaffolds [4–6] and are well-known for their use as anxiolytics, hypnotics, and muscle relaxants (Figure 1) [7–8]. Diazepam, sold under the brand name Valium, is among the first marketed medications of the benzodiazepine family [9]. Alprazolam, sold under the brand name Xanax, is a fast-acting, potent tranquilizer with moderate duration, belonging to the triazolobenzodiazepine family [10]. It is derived through bioisosteric replacement of the benzodiazepine amide moiety with a triazole ring. Replacements of the benzene ring with thiophene and even with pyrrole are also known. Premazepam, which features a pyrrolodiazepine core, has been studied both alone and in combination with diazepam for its antianxiety and sedative effects [11]. Flumazenil comprises an imidazobenzodiazepine scaffold and its intravenous administration is used to treat benzodiazepine overdoses [12–13] and to reverse anesthesia [14]. The imidazo[4,5-*d*][1,3]diazepine core is present in pentostatin and coformycin, which are naturally occurring *N*-nucleoside inhibitors of adenosine deaminase, known for their antibiotic and anticancer properties [15–17]. These examples highlight the importance of developing novel synthetic methods to access diazepines fused with other nitrogen-containing heterocycles towards their broader exploration in high-throughput screening for identifying new drug candidates.

Traditional synthetic methods for producing medium-ring nitrogen-containing heterocycles often involve complex procedures and harsh reaction conditions, resulting in limited substituent and scaffold diversity [18–19]. In this regard, multicomponent reactions (MCRs) have gained increasing attention for their operational simplicity, efficiency, robustness, atom economy, and potential for diversity-oriented synthesis [20–23]. For example, the Ugi four-component reaction (U4CR), involving a carbonyl compound, a primary amine, a carboxylic acid, and an isocyanide, provides a straightforward method for constructing dipeptide-like adducts [24–27]. These adducts can subsequently be rigidified into heterocyclic peptidomimetics through various post-MCR transformations [28–32].

**Scheme 1:**
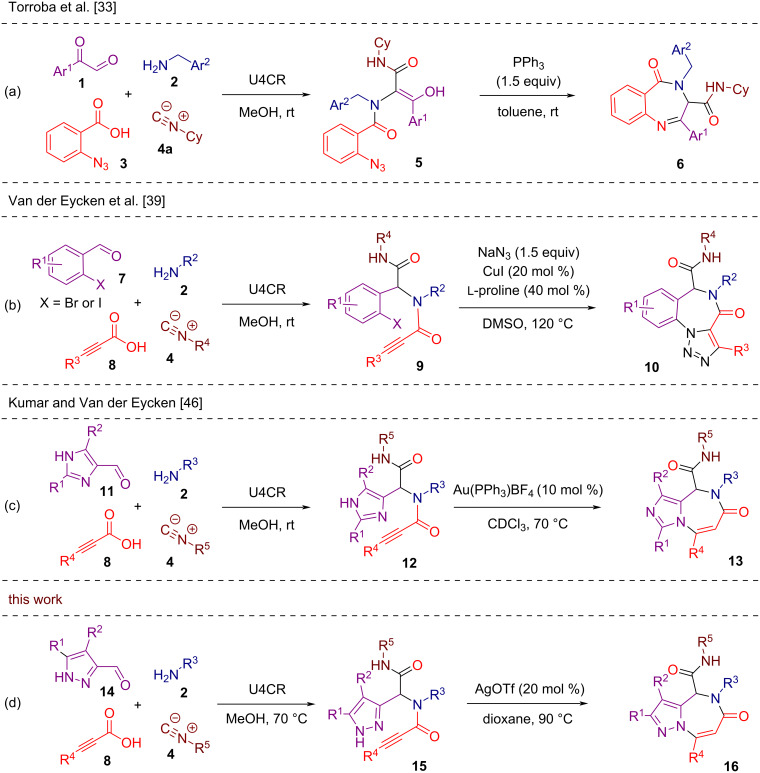
Post-Ugi synthesis of benzodiazepines and heteroaryl-fused diazepines.

The construction of benzodiazepine cores has also been extensively explored through various post-Ugi transformations. In 2009, Torroba and co-workers developed a strategy towards β-turn mimetic benzo[*e*][1,4]diazepines **6** involving the Ugi reaction between arylglyoxals **1**, benzylamines **2**, *o*-azidobenzoic acid (**3**), and cyclohexyl isocyanide (**4a**), followed by a triphenylphosphine-promoted tandem Staudinger/aza-Wittig cyclization (Scheme 1a) [33]. The overall strategy was enabled by the presence of an azide group in the carboxylic acid component **3** and additional keto-carbonyl group in aryl glyoxal **1**. This approach was further advanced by Ding's group to produce a broader range of benzodiazepines with diverse substitution patterns by shuffling the necessary functional groups within the Ugi reaction components [34–36]. In 2015, García-Valverde and co-workers described an alternative synthesis of benzo[*e*][1,4]diazepines **6**, exploring the nitro group of 2-nitrobenzoic acid as a masked amino group. The release was achieved through the post-Ugi reduction of the nitro group with SnCl_2_ triggering concomitant intramolecular condensation with the arylglyoxal-derived keto-carbonyl group [37]. In 2024, the same group streamlined this strategy by utilizing unprotected anthranilic acids, enabling the assembly of benzo[*e*][1,4]diazepines **6** directly during the Ugi reaction step [38].

In 2013, Van der Eycken and co-workers employed the U4CR of *ortho*-halogenated benzaldehydes **7**, primary amines **2**, 3-substituted propiolic acids **8**, and isocyanides **4** to synthesize propargylamides **9**. These propargylic Ugi adducts **9** were subsequently subjected to a Cu-catalyzed tandem azide–alkyne cycloaddition/Ullmann coupling resulting in the formation of the tricyclic triazolo[1,5-*a*][1,4]benzodiazepine scaffold **10** (Scheme 1b) [39].

Triple bond-containing Ugi adducts showed a great promise for the assembly of various seven-membered nitrogen-containing heterocyclic cores through transition-metal-catalyzed alkyne hydroarylations [40–44] and hydroalkoxylations [45].

In 2013, Van der Eycken and co-workers described an intramolecular cationic gold-catalyzed post-Ugi heteroannulation of imidazoles with activated alkynes for the diversity-oriented synthesis of imidazo[1,4]diazepines **13** from Ugi-derived propargylamides **12** (Scheme 1c) [46]. In 2019, Li, Yang, Van der Eycken and co-workers reported a modification of this strategy relying on thermal activation instead of cationic gold catalysis [47]. The approach worked particularly well with substrates featuring terminal alkynes.

Inspired by these developments and taking into account chemical and pharmacological importance of fused pyrazole derivatives [48–53], we herein report the post-Ugi assembly of novel pyrazolo[1,5-*a*][1,4]diazepine scaffolds **16** (Scheme 1d). The synthetic sequence involves an Ugi reaction of pyrazole-3-carbaldehydes **14**, primary amines **2**, 3-substituted propiolic acids **8**, and isocyanides **4** followed by a silver-catalyzed heteroannulation of the resulting Ugi adducts **15**. While several protocols for synthesizing medium-ring-fused pyrazoles have been reported recently [54–58], our method offers notable advantages, including operational simplicity, the accessibility of starting materials, and a diversity-oriented approach.

## Results and Discussion

**Scheme 2:**
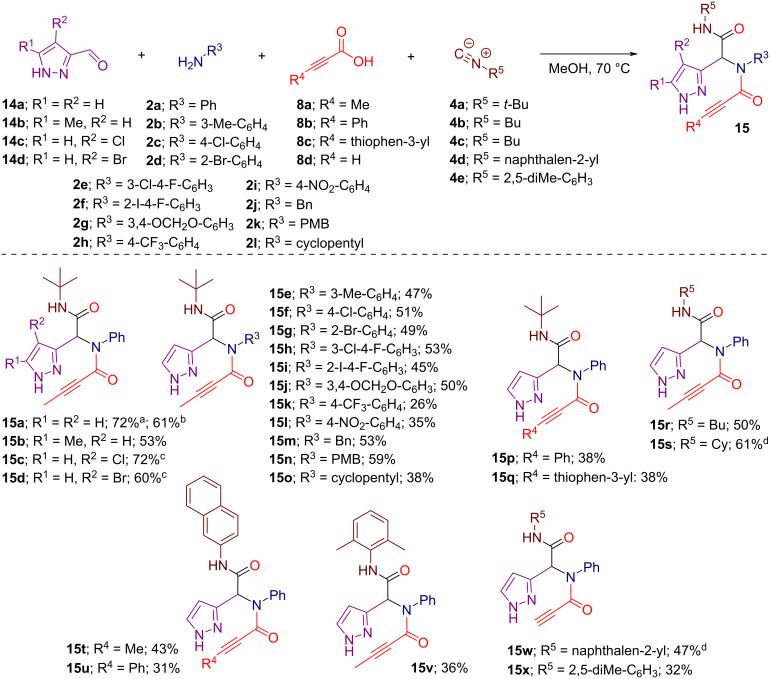
Synthesis of pyrazole-tethered propargylamides **15** via U4CR. Conditions: Unless otherwise specified, the reactions were run on a 1.0 mmol scale in methanol (5 mL). The reactions were conducted in screw cap vials at 70 °C for 24 hours and isolated yields are reported. ^a^Conducted on a 6.0 mmol scale. ^b^Conducted at room temperature. ^c^Conducted on a 2.0 mmol scale. ^d^Conducted on a 1.5 mmol scale.

First, we prepared a series of acyclic pyrazole-tethered propargylamide precursors **15** via the Ugi four-component reaction (U4CR) of 1*H*-pyrazole-3-carbaldehydes **14a**–**d**, primary amines **2a**–**l**, propiolic acids **8a**–**d**, and isocyanides **4a**–**e**. By conducting the reactions in methanol at 70 °C, we obtained the desired Ugi adducts **15a**–**x** in fair to good yields of 26–72% allowing for the variation of substituents across all components of the U4CR (Scheme 2). Notably, the Ugi reaction toward substrate **15a**, when performed at room temperature, proceeded with lower efficiency compared to the reaction at 70 °C, leaving some of the starting 1*H*-pyrazole-3-carbaldehyde (**14a**) unreacted.

Propargylamide **15a** was selected as a model substrate to optimize the reaction conditions for the intramolecular heteroannulation leading to the pyrazolo[1,5-*a*][1,4]diazepine scaffold. Our initial choice was to investigate the use of silver(I) triflate (AgOTf) as a catalyst in toluene, given its previously demonstrated efficiency in various transformations involving the activation of triple bonds towards nucleophilic attack by nitrogen nucleophiles [59–60]. When the reaction was conducted with 5 mol % of AgOTf in toluene at 80 °C for 20 hours, pyrazolo[1,5-*a*][1,4]diazepine **16a** was obtained in 46% yield, while complete conversion of the starting material **15a** was not achieved (Table 1, entry 1). Increasing the temperature to 90 °C and the catalyst loading to 10 mol % enabled full conversion of **15a** and improved the yield of product **16a** (Table 1, entries 2 and 3). The use of other silver salts including AgBF_4_, Ag(NTf)_2_ and AgNO_3_ did not lead to improved results, with Ag(NTf)_2_ being particularly ineffective (Table 1, entries 4–6). Next, various solvents were screened with TFE, DCE, acetonitrile, and chlorobenzene providing low to moderate yields, whereas dioxane emerged as the preferred solvent allowing to bring the yield of pyrazolodiazepine **16a** to 66% (Table 1, entries 7–11). Finally, further increasing the catalyst loading to 20 mol % resulted in an additional improvement in the yield of **16a** in both toluene and dioxane (Table 1, entries 12 and 13). The best result was achieved using 20 mol % of AgOTf in dioxane, with the reaction conducted at 90 °C for 7 hours furnishing **16a** in an excellent 94% yield (Table 1, entry 13). Catalytic systems based on other transition metals including Cu(OTf)_2_ and the AuPPh_3_Cl/AgOTf combination were also tested (Table 1, entries 14 and 15). However, their performance was significantly poorer compared to AgOTf.

**Table 1:** Optimization reactions of the intramolecular post-Ugi heteroannulation.^a^

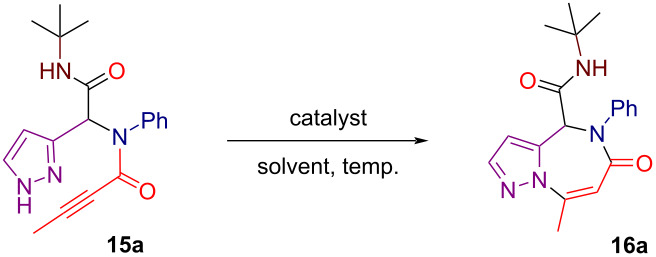						

Entry	Catalyst	Solvent	Time [h]	Temp. [°C]	Yield of **12** [%]^b^	Conversion [%]

1	AgOTf (5 mol %)	toluene	20	80	46	65
2	AgOTf (5 mol %)	toluene	27	90	49	100
3	AgOTf (10 mol %)	toluene	12	90	60	100
4	AgBF_4_ (10 mol %)	toluene	12	90	49	100
5	Ag(NTf)_2_ (10 mol %)	toluene	12	90	2	64
6	AgNO_3_ (10 mol %)	toluene	12	90	45	100
7	AgOTf (10 mol %)	TFE	12	90	16	27
8	AgOTf (10 mol %)	dioxane	12	90	66	100
9	AgOTf (10 mol %)	DCE	12	90	27	50
10	AgOTf (10 mol %)	chlorobenzene	12	90	53	100
11	AgOTf (10 mol %)	acetonitrile	12	90	24	100
12	AgOTf (20 mol %)	toluene	7	90	82	100
13	AgOTf (20 mol %)	dioxane	7	90	94^c^	100
14	AuPPh_3_Cl/AgOTf (5 mol %)	DCM	24	rt	1	31
15	Cu(OTf)_2_ (20 mol %)	toluene	12	90	4	100
16	–	dioxane	7	90	–^d^	–^d^

^a^All reactions were carried out on a 0.2 mmol scale in 1.0 mL of solvent in a sealed vial. ^b^Determined by ^1^H NMR spectroscopy using 2,4,6-trimethoxybenzaldehyde as internal standard. ^c^Also, corresponds to the isolated yield. ^d^No consumption of **15a** was observed.

**Scheme 3:**
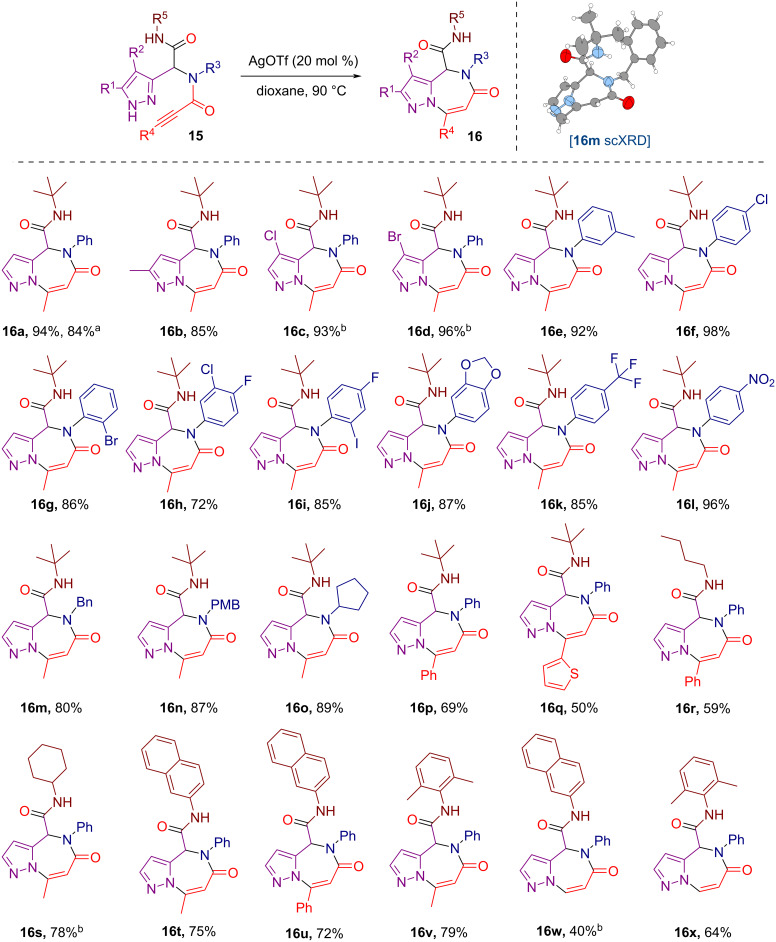
Scope of the silver(I) triflate-catalyzed synthesis of pyrazolo[1,5-*a*][1,4]diazepines. Conditions: Unless otherwise specified, the reactions were run on 0.2 mmol scale using 20 mol % of AgOTf in dioxane (1 mL). The reactions were conducted in screw cap vials at 90 °C for 7 hours and isolated yields are reported. ^a^Conducted on a 3.5 mmol scale. ^b^Conducted on a 0.5 mmol scale.

To evaluate the scope and limitations of the optimized protocol (Table 1, entry 13), a series of U4CR-derived pyrazole-tethered propargylamides **15** was subjected to the silver(I) triflate-catalyzed heteroannulation into pyrazolo[1,5-*a*][1,4]diazepines **16** (Scheme 3). Employing substrates **15a**–**d**, derived from different 1*H*-pyrazole-3-carbaldehydes, the corresponding pyrazolodiazepines **16a**–**d** were obtained in consistently high yields of 84–96%. Notably, the synthesis of the parent pyrazolodiazepine **16a** was scaled up to 3.5 mmol without a substantial decrease in isolated yield.

To explore the influence of the amine component on the process, an extensive subset of aniline-derived substrates **15e**–**l** was tested. The process proceeded efficiently in all cases, yielding pyrazolodiazepines **16e**–**l** and demonstrating tolerance for various substituents on the aniline-derived aromatic fragment, including alkyl, halogens, electron-donating alkoxy groups, as well as electron-withdrawing trifluoromethyl and nitro groups. Furthermore, substrates **15m**–**o** derived from aliphatic amines, also performed well, furnishing pyrazolodiazepines **16m**–**o** in up to 89% yield. The structure of **16m**, a representative compound of this series, was confirmed through single-crystal X-ray diffraction (scXRD) analysis.

Another set of pyrazolodiazepines **16p**–**v** was readily obtained from the substrates **15p**–**v** stemming from various 3-substituted propiolic acids and aliphatic or aromatic isocyanides. Finally, the annulation of substrates **15w** and **15x**, featuring a terminal alkyne, also proceeded in a 7-*endo*-*dig* fashion, yielding pyrazolodiazepines **16w** and **16x**, respectively. Such an outcome is notable, as related carbocyclizations often switch to an *exo* mode when shifting from internal to terminal alkynes [61–63].

**Scheme 4:**
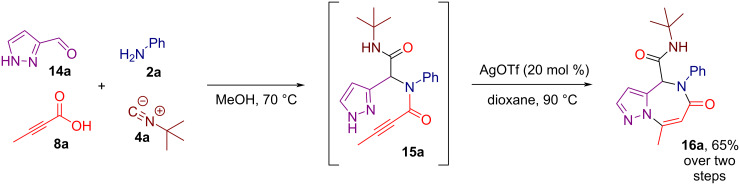
Telescope procedure for the synthesis of **16a**.

To demonstrate the robustness of our methodology, we tested a telescope procedure in which, after the Ugi step, the product was not isolated. Instead, the reaction mixture was concentrated and directly subjected to the subsequent heteroannulation step. This approach proved to be feasible, affording the model pyrazolo[1,5-*a*][1,4]diazepine **16a** in 65% overall yield over two steps (Scheme 4).

**Scheme 5:**
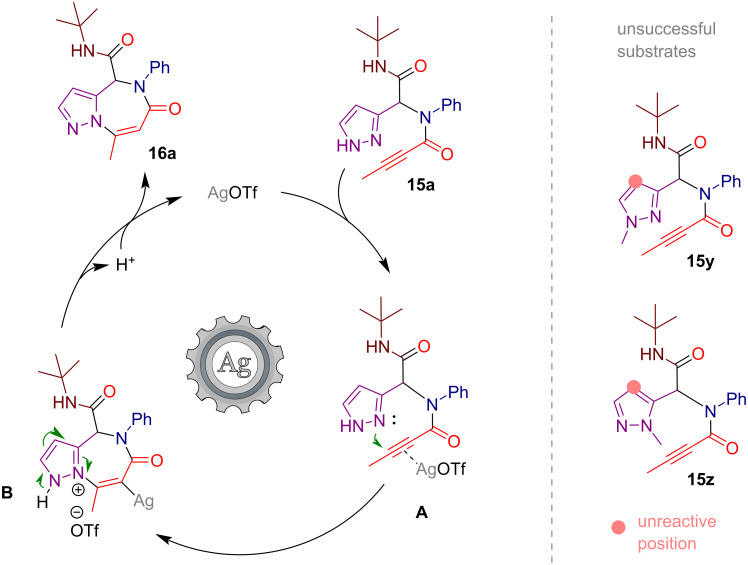
Tentative mechanism for the silver-catalyzed heteroannulation.

The tentative mechanism for the studied silver(I)-catalyzed intramolecular heteroannulation reaction is depicted in Scheme 5. The process begins with the π-coordination of the silver catalyst to the triple bond in the Ugi-adduct **15a** generating intermediate **A**. This is followed by a nucleophilic attack by the pyrazole nitrogen on the activated alkyne in an *endo*-*dig* fashion, forming a 7-membered ring. The resulting intermediate **B** undergoes proton transfer from the second pyrazole nitrogen to the vinyl silver moiety yielding pyrazolo[1,5-*a*][1,4]diazepine **16a** and regenerating the silver catalyst. Methylation of either pyrazole nitrogen atom prevents the reaction by blocking the nucleophilic attack on the triple bond or the subsequent proton transfer step, thereby preventing substrates **15y** and **15z** from undergoing the described heteroannulation. Additionally, the alternative carbocyclization pathway cannot occur, as the vacant 4C position of the pyrazole ring lacks sufficient nucleophilicity.

**Scheme 6:**
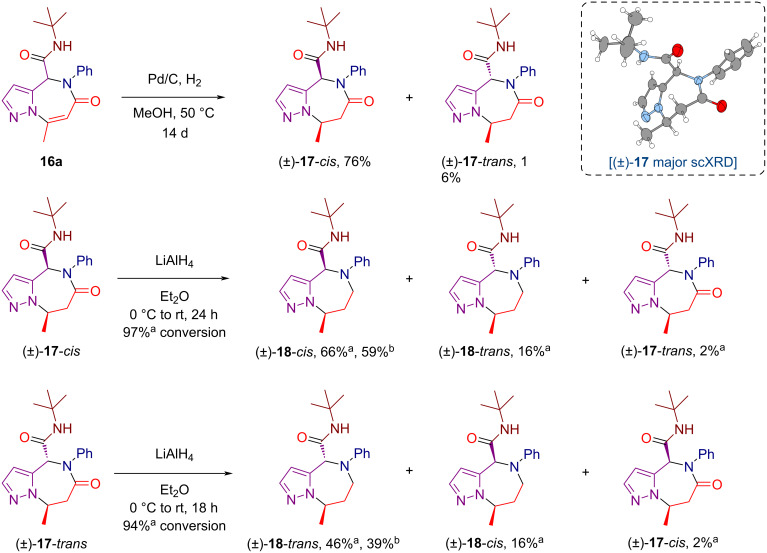
Reductive post-assembly modifications of the pyrazolo[1,5-*a*][1,4]diazepine core. ^a^Determined by ^1^H NMR spectroscopy analysis of crude reaction mixtures. ^b^Isolated yield.

To further expand the scope of our chemistry, we conducted a series of reductive post-assembly modifications of the pyrazolo[1,5-*a*][1,4]diazepine core, following our recently developed strategy for enriching the sp^3^ character in post-Ugi scaffolds (Scheme 6) [64–65]. First, we attempted heterogeneous hydrogenation of the alkene functionality in compound **16a** under 1 atm hydrogen pressure using Pd/C as a catalyst. Although the reaction proved sluggish, we were able to drive it to completion over a prolonged reaction time of 14 days, obtaining a separable mixture of the major *cis* and minor *trans* diastereomers of the sp^3^-enriched pyrazolodiazepine **17**. The relative configuration of the stereocenters in the major *cis* isomer of **17** was established via scXRD analysis. Both diastereomers of **17** were found to be amenable to chemoselective amide reduction with LiAlH₄, which led to a further decrease in the degree of unsaturation of the pyrazolodiazepine core, while the more sterically hindered exocyclic amide moiety remained intact. However, both reactions were accompanied by partial epimerization, and careful kinetic control was therefore required to obtain non-epimerized diastereomers of the resulting pyrazolodiazepine **18** as the major reaction products.

## Conclusion

We have developed a straightforward, diversity-oriented approach for the synthesis of a novel pyrazolo[1,5-*a*][1,4]diazepine scaffold starting from readily available building blocks. The first step, the Ugi-4CR, introduces molecular diversity, while the second step, an efficient silver(I)-catalyzed heteroannulation, facilitates the formation of the pyrazolodiazepine core via alkyne activation towards the nucleophilic attack by the pyrazole nitrogen. This strategy expands the array of post-Ugi transformations leading to the formation of fused seven-membered heterocycles. A series of target pyrazolodiazepines were generated through the variation of substitution patterns on all components of the Ugi reaction while several limitations of the methodology were also identified. In addition, a sequence of reductive post-assembly modifications aimed at increasing the sp³ character of the pyrazolo[1,5-*a*][1,4]diazepine core was successfully implemented.

## Supporting Information

Deposition Numbers 2410526 (for **16m**) and 2441192 (for **17-***cis*) contain the supplementary crystallographic data for this paper. These data are provided free of charge by the joint Cambridge Crystallographic Data Centre and Fachinformationszentrum Karlsruhe Access Structures service.

File 1 Detailed descriptions of the experimental procedures, product characterization data and the copies of NMR spectra.

## Data Availability

All data that supports the findings of this study is available in the published article and/or the supporting information of this article.
