# Supplementary material for: Silver(I) triflate-catalyzed post-Ugi synthesis of pyrazolodiazepines
Source: Beilstein J Org Chem. 2025 May 8;21:915–25. doi: 10.3762/bjoc.21.74 (PMC12067096; doi:10.3762/bjoc.21.74)

BEILSTEIN JOURNAL OF ORGANIC CHEMISTRY

Supporting Information

for

Silver(I) triflate-catalyzed post-Ugi synthesis of pyrazolodiazepines

Muhammad Hasan, Anatoly A. Peshkov, Syed Anis Ali Shah, Andrey Belyaev, Chang-Keun Lim, Shunyi Wang and Vsevolod A. Peshkov

Beilstein J. Org. Chem. **2025,** *21,* 915–925. doi:10.3762/bjoc.21.74

Detailed descriptions of the experimental procedures, product characterization data and the copies of NMR spectra

License and Terms: This is a supporting information file under the terms of the Creative Commons Attribution License (https://creativecommons.org/licenses/by/4.0). Please note that the reuse, redistribution and reproduction in particular requires that the author(s) and source are credited and that individual graphics may be subject to special legal provisions.
The license is subject to the *Beilstein Journal of Organic Chemistry* terms and conditions: (https://www.beilstein-journals.org/bjoc/terms)

Table of content

General Remarks	S2
Synthesis and characterization	S3
General procedure for the synthesis of Ugi adducts **15**	S3
Large scale procedure for the synthesis of Ugi adduct **15a**	S3
General procedure for the synthesis of pyrazolodiazepines **16**	S11
Telescope procedure for the synthesis of pyrazolodiazepine **16a**	S18
Procedure for heterogeneous hydrogenation of **16a**	S19
Procedure for the synthesis of pyrazolodiazepine **18**	S19
X-ray diffraction analysis	S22
Copies of NMR spectra	S25-S78

General Remarks

Materials and solvents. All reagents and solvents were purchased from commercial sources and used without further purification.

NMR spectroscopy. NMR spectroscopic data were recorded with a JEOL JNM-ECA 500 MHz spectrometer (500.16 MHz for ^{1}H and 125.78 MHz for ^{13}C{^{1}H}), Bruker Avance III 300 MHz spectrometer (300.13 MHz for ^{1}H and 75.47 MHz for ^{13}C{^{1}H}) and a Bruker Avance III 400 MHz spectrometer (400.13 MHz for ^{1}H and 100.61 MHz for ^{13}C{^{1}H}), in DMSO-*d*6 or $CDCl_3$ and were referenced to solvent residual proton signal (δH = 2.50 and 7.26 ppm, respectively) or to TMS signal (δH = 0 ppm) and solvent carbon signal (δC = 39.52 and 77.16 ppm, respectively).

Mass spectrometry. Mass spectra were recorded by using BRUKER microTOF-Q III instrument with ESI source.

Melting points. Melting points were determined in open capillary tubes on Stuart Scientific SMP3 melting point apparatus.

Synthesis and characterization

General procedure for the synthesis of Ugi adducts 15:

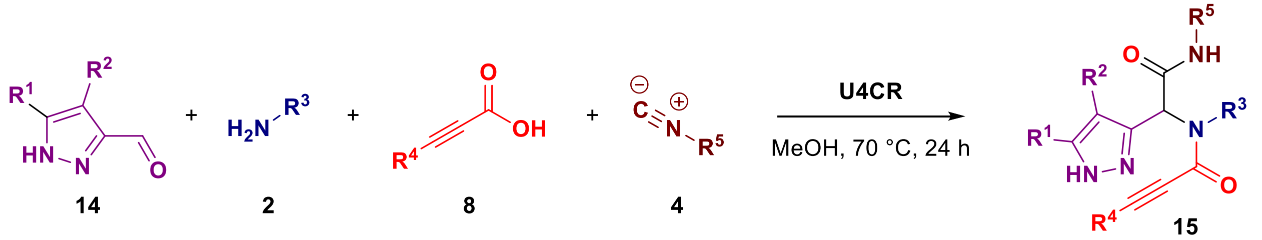

Pyrazole-3-carbaldehyde **14** (1 mmol, 1.0 equiv) was placed in screw cap vial charged with magnetic stirrer followed by the addition of methanol (5.0 mL), 3-substituted propiolic acid **8**, (1.0 mmol, 1.0 equiv), amine **2** (1.0 mmol, 1.0 equiv) and isocyanide **4** (1.0 mmol, 1.0 equiv). The reaction mixture was sealed and stirred at 70 °C for 24 h. The resulting mixture was diluted with EtOAc and concentrated with silica. Column chromatography was performed using a petroleum ether/ethyl acetate as the eluent (the ratio was adjusted based on TLC analysis), affording Ugi adduct **15**. Most products **15** were additionally purified by consecutive trituration with n-hexane and diethyl ether.

Large scale procedure for the synthesis of Ugi adduct 15a:

Pyrazole-3-carboxaldehyde (**14a**, 577 mg, 6 mmol, 1.0 equiv) was placed in screw cap vial charged with magnetic stirrer followed by the addition of methanol (30 mL), 2-butynoic acid (**8a**, 505 mg, 6.0 mmol, 1.0 equiv), aniline (**2a**, 559 mg, 6.0 mmol, 1.0 equiv) and tert-butyl isocyanide (**4a**, 499 mg, 6.0 mmol, 1.0 equiv). The reaction mixture was sealed and stirred at 70 °C for 24 h. The resulting mixture was diluted with EtOAc and concentrated with silica. Column chromatography was performed using a hexane/ethyl acetate as the eluent (the ratio was adjusted based on TLC analysis), affording Ugi adduct **15a**.

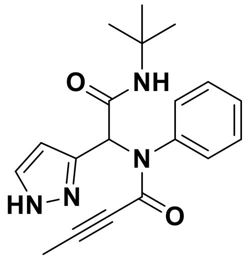	**N-(2-(tert-butylamino)-2-oxo-1-(1H-pyrazol-3-yl)ethyl)-N-phenylbut-2-ynamide (15a).** Additional purification involved consecutive trituration with cold pentane and diethyl ether. Yield: 1.47 g, 72% (6.0 mmol scale), white powder, m.p. = 191-193 °C; **^{1}H NMR** (400 MHz, $CDCl_3$) δ 10.83 (bs, 1H), 7.46 (d, J = 2.1 Hz, 1H), 7.30 – 7.25 (m, 3H), 7.19 – 7.08 (m, 2H), 6.40 (bs, 1H), 6.24 (d, J = 2.1 Hz, 1H), 5.98 (s, 1H), 1.70 (s, 3H), 1.35 (s, 9H). **^{13}C{^{1}H} NMR** (101 MHz, $CDCl_3$) δ 167.0, 155.0, 140.0, 134.1 (bs), 129.7, 128.8, 128.6, 106.7, 92.0, 73.8, 58.7 (bs), 51.8, 28.6, 4.0. **HRMS** (ESI): m/z $[M+H]^+$ for $C_{19}H_{23}N_4O_2^+$, calcd 339.1816, found 339.1808.

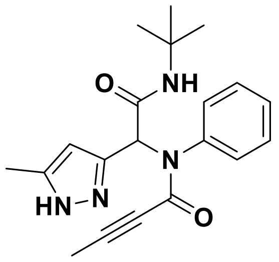	**N-(2-(tert-butylamino)-1-(5-methyl-1H-pyrazol-3-yl)-2-oxoethyl)-N-phenylbut-2-ynamide (15b).**<br>Yield: 186 mg, 53%, white powder, m.p. = 85-87 °C; ^{1}**H NMR** (400 MHz, $CDCl_3$) δ 7.31 – 7.27 (m, 3H), 7.22 – 7.16 (m, 2H), 6.43 (bs, 1H), 6.01 (s, 1H), 5.81 (s, 1H), 2.24 (s, 3H), 1.70 (s, 3H), 1.34 (s, 9H). ^{13}**C{^{1}H} NMR** (101 MHz, $CDCl_3$) δ 166.8, 155.0, 143.5, 140.5, 129.5, 128.8, 128.5, 106.1, 91.8, 73.9, 59.5, 51.7, 28.6, 12.0, 4.0. **HRMS** (ESI): m/z $[M+H]^+$ for $C_{20}H_{25}N_4O_2^+$, calcd 353.1972, found 353.1971.
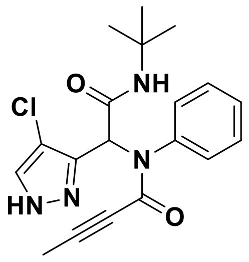	**N-(2-(tert-butylamino)-1-(4-chloro-1H-pyrazol-3-yl)-2-oxoethyl)-N-phenylbut-2-ynamide (15c).**<br>No trituration was performed after column chromatography. Yield: 537 mg, 72% (2.0 mmol scale); white solid, m.p. = 200-202 °C; ^{1}**H NMR** (500 MHz, $CDCl_3$) δ 11.26 (bs, 1H), 7.39 (s, 1H), 7.32 – 7.23 (m, 3H), 7.08 – 6.98 (m, 2H), 6.62 (bs, 1H), 6.22 (s, 1H), 1.72 (s, 3H), 1.41 (s, 9H). ^{13}**C{^{1}H} NMR** (126 MHz, $CDCl_3$) δ 167.1, 155.3, 138.3, 137.0 (bs), 133.0 (bs), 129.2, 129.2, 129.1, 110.9, 93.2, 73.2, 53.2, 52.2, 28.6, 4.1. **HRMS** (ESI): m/z $[M+H]^+$ for $C_{19}H_{22}ClN_4O_2^+$, calcd 373.1426, found 373.1426.
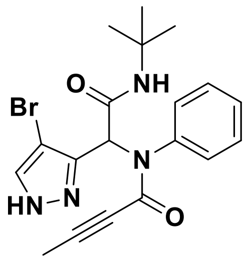	**N-(1-(4-bromo-1H-pyrazol-3-yl)-2-(tert-butylamino)-2-oxoethyl)-N-phenylbut-2-ynamide(15d).**<br>No trituration was performed after column chromatography. Yield: 504 mg, 60% (2.0 mmol scale); white solid, m.p. = 206-208 °C; ^{1}**H NMR** (500 MHz, $CDCl_3$) δ 11.45 (bs, 1H), 7.43 (s, 1H), 7.32 – 7.21 (m, 3H), 7.09 – 6.97 (m, 2H), 6.66 (bs, 1H), 6.22 (s, 1H), 1.72 (s, 3H), 1.41 (s, 9H). ^{13}**C{^{1}H} NMR** (126 MHz, $CDCl_3$) δ 167.1, 155.3, 139.3 (bs), 138.2, 134.3 (bs), 129.2, 129.2, 129.1, 95.9, 93.2, 73.2, 53.7, 52.1, 28.6, 4.1. **HRMS** (ESI): m/z $[M+H]^+$ for $C_{19}H_{22}BrN_4O_2^+$, calcd 417.0921, found 417.0926.
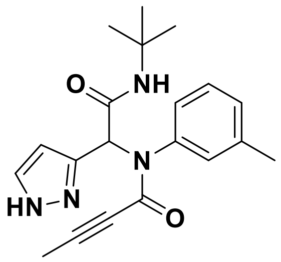	**N-(2-(tert-butylamino)-2-oxo-1-(1H-pyrazol-3-yl)ethyl)-N-(m-tolyl)but-2-ynamide (15e).**<br>Yield: 165 mg, 47%, white powder, m.p. = 75-77 °C; ^{1}**H NMR** (400 MHz, $CDCl_3$) δ 7.49 – 7.45 (m, 1H), 7.20 – 7.06 (m, 2H), 6.99 – 6.95 (m, 1H), 6.94 – 6.89 (m, 1H), 6.37 (bs, 1H), 6.27 – 6.23 (m, 1H), 5.92 (s, 1H), 2.28 (s, 3H), 1.71 (s, 3H), 1.35 (s, 9H). ^{13}**C{^{1}H} NMR** (101 MHz, $CDCl_3$) δ 167.1, 154.9, 144.1 (bs), 140.0, 138.5, 132.4 (bs), 130.0, 129.1, 128.3, 126.6, 106.4 (bs), 91.6, 73.9, 59.6, 51.6, 28.5, 21.1, 3.9. **HRMS** (ESI): m/z $[M+H]^+$ for $C_{20}H_{25}N_4O_2^+$, calcd 353.1972, found 353.1972.

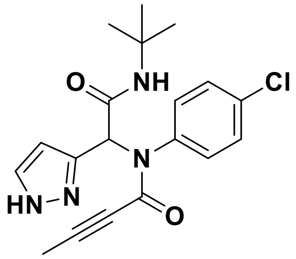	**N-(2-(tert-butylamino)-2-oxo-1-(1H-pyrazol-3-yl)ethyl)-N-(4-chlorophenyl)but-2-ynamide (15f).**<br>Yield: 190 mg, 51%, white powder, m.p. = 179-181 °C; **^{1}H NMR** (400 MHz, $CDCl_3$) δ 7.46 (d, J = 2.2 Hz, 1H), 7.23 (d, J = 8.7 Hz, 2H), 7.11 (d, J = 8.6 Hz, 2H), 6.40 (bs, 1H), 6.20 (d, J = 2.2 Hz, 1H), 5.99 (s, 1H), 1.72 (s, 3H), 1.33 (s, 9H). **^{13}C{^{1}H} NMR** (101 MHz, $CDCl_3$) δ 167.1, 154.7, 143.7 (bs), 138.5, 134.2, 132.3 (bs), 131.4, 128.7, 106.5, 92.1, 73.7, 58.9, 51.7, 28.5, 3.9. **HRMS** (ESI): m/z $[M+H]^+$ for $C_{19}H_{22}ClN_4O_2^+$, calcd 373.1426, found 373.1420.
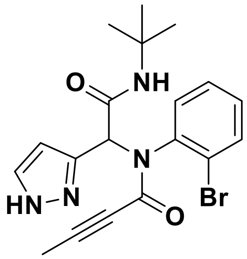	**N-(2-bromophenyl)-N-(2-(tert-butylamino)-2-oxo-1-(1H-pyrazol-3-yl)ethyl)but-2-ynamide (15g).**<br>Yield: 204 mg, 49%, white powder, m.p. = 160-163 °C; **^{1}H NMR** (400 MHz, $CDCl_3$, observed as a mixture of rotamers ≈ 65:35) δ 7.71 (dd, J = 7.9, 1.7 Hz, 0.65H, major), 7.59 (dd, J = 8.0, 1.5 Hz, 0.35H, minor), 7.53 (d, J = 2.0 Hz, 0.35H, minor), 7.47 (dd, J = 7.9, 1.7 Hz, 0.35H, minor), 7.43 (dd, J = 8.0, 1.5 Hz, 0.65H, major), 7.32 (d, J = 2.1 Hz, 0.65H, major), 7.31 – 7.26 (m, 1H, major + minor), 7.19 (td, J = 7.7, 1.7 Hz, 0.35H, minor), 7.13 (td, J = 7.7, 1.7 Hz, 0.65H, major), 6.45 – 6.39 (m, 1H [(bs, 0.65H, major) + (d, *J* = 2.1 Hz, 0.35H, minor)]), 6.06 (d, J = 2.2 Hz, 0.65H, major), 6.04 (s, 0.65H, major), 5.99 (bs, 0.35H, minor), 5.44 (s, 0.35H, minor), 1.70 (s, 1.05H, minor), 1.69 (s, 1.95H, major), 1.36 (s, 5.85H, major), 1.31 (s, 3.15H, minor). **^{13}C{^{1}H} NMR** (101 MHz, $CDCl_3$) δ 167.6 (major), 165.8 (minor), 155.4 (minor), 155.0 (major), 142.3 (minor), 141.3 (major), 140.7 (minor), 138.6 (major), 134.6 (minor), 133.1 (minor), 132.8 (major), 132.6 (major), 132.4 (major), 131.6 (minor), 130.2 (major), 130.1 (minor), 128.4 (minor), 128.0 (major), 126.2 (major), 125.0 (minor), 107.2 (minor), 106.9 (major), 91.0 (minor), 90.5 (major), 73.85 (major), 73.80 (minor), 61.1 (minor), 58.6 (major), 51.9 (minor), 51.8 (major), 28.61 (major), 28.57 (minor), 3.9 (major + minor). **HRMS** (ESI): m/z $[M+H]^+$ for $C_{19}H_{22}BrN_4O_2^+$, calcd 417.0921, found 417.0922.
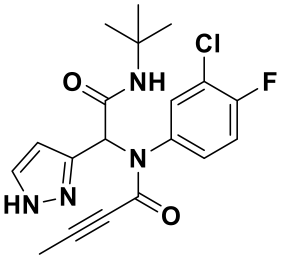	**N-(2-(tert-butylamino)-2-oxo-1-(1H-pyrazol-3-yl)ethyl)-N-(3-chloro-4-fluorophenyl)but-2-ynamide (15h).**<br>Yield: 207 mg, 53%, white powder, m.p. = 131-133 °C; **^{1}H NMR** (400 MHz, $CDCl_3$, observed as a mixture of rotamers ≈ 80:20, only signals belonging to the major rotamer are listed) δ 9.79 (bs, 1H), 7.44 (d, J = 2.3 Hz, 1H), 7.33 – 7.29 (m, 1H), 7.14 – 7.07 (m, 1H), 6.95 (t, J = 8.7 Hz, 1H), 6.71 (s, 1H), 6.08 (d, J = 2.2 Hz, 1H), 6.07 (s, 1H), 1.68 (s, 3H), 1.27 (s, 9H). **^{13}C{^{1}H} NMR** (101 MHz, $CDCl_3$) δ 167.0, 157.7 (d, J = 250.7 Hz), 154.7, 144.0, 136.4 (d, J = 3.7 Hz), 132.5, 131.9, 130.5 (d, J = 7.5 Hz), 120.5 (d, J = 18.7 Hz),

	116.1 (d, J = 22.0 Hz), 106.5, 92.5, 73.6, 58.9, 51.8, 28.5, 3.9. **HRMS** (ESI): m/z $[M+H]^+$ for $C_{19}H_{21}ClFN_4O_2^+$, calcd 391.1332, found 391.1330.
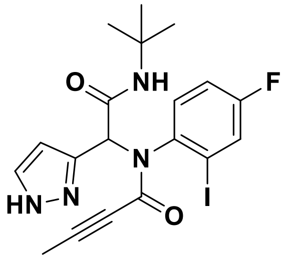	**N-(2-(tert-butylamino)-2-oxo-1-(1H-pyrazol-3-yl)ethyl)-N-(4-fluoro-2-iodophenyl)but-2-ynamide (15i).** Yield: 215 mg, 45%, white powder, m.p. = 191-193 °C; **^{1}H NMR** (400 MHz, $CDCl_3$, observed as a mixture of rotamers ≈ 70:30) δ 7.83 (dd, J = 8.8, 5.6 Hz, 0.70H, major), 7.65 (dd, J = 8.8, 5.5 Hz, 0.30H, minor), 7.57 (d, J = 2.0 Hz, 0.30H, minor), 7.54 (dd, J = 7.7, 2.9 Hz, 0.30H, minor), 7.37 (dd, J = 7.8, 2.8 Hz, 0.70H, major), 7.35 (d, J = 2.3 Hz, 0.70H, major), 7.12 – 7.01 (m, 1H, major + minor), 6.45 (bs, 0.3H, minor), 6.25 (bs, 0.70H, major), 6.11 (s, 0.70H, major), 6.01 – 5.96 (m, 0.70H, major), 5.81 – 5.73 (m, 0.30H, minor), 5.23 (s, 0.30H, minor), 1.73 (s, 0.90H, minor), 1.71 (s, 2.10H, major), 1.34 (s, 6.30H, major), 1.30 (s, 2.70H, minor). **^{13}C{^{1}H} NMR** (101 MHz, $CDCl_3$, only signals belonging to the major rotamer are listed) δ 167.7, 161.4 (d, J = 254.0 Hz), 155.1, 138.4 (d, J = 3.5 Hz), 133.2 (d, J = 9.0 Hz), 125.8 (d, J = 24.7 Hz), 115.8 (d, J = 21.9 Hz), 107.2, 103.9 (d, J = 8.7 Hz), 91.1, 74.1, 58.6, 51.9, 28.7, 4.0. **HRMS** (ESI): m/z $[M+H]^+$ for $C_{19}H_{21}FIN_4O_2^+$, calcd 483.0688, found 483.0688.
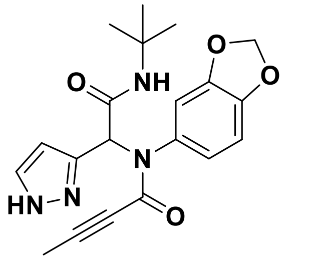	**N-(benzo[d][1,3]dioxol-5-yl)-N-(2-(tert-butylamino)-2-oxo-1-(1H-pyrazol-3-yl)ethyl)but-2-ynamide (15j).** Yield: 191 mg, 50%, dark brown powder, m.p. = 78-81 °C; **^{1}H NMR** (400 MHz, $CDCl_3$) δ 8.27 (bs, 1H), 7.44 (s, 1H), 6.69 – 6.60 (m, 3H), 6.55 (s, 1H), 6.20 – 6.14 (m, 1H), 5.98 (bs, 1H), 5.94 (s, 2H), 1.73 (s, 3H), 1.31 (s, 9H). **^{13}C{^{1}H} NMR** (101 MHz, $CDCl_3$) δ 167.0, 155.3, 147.6, 147.5, 133.7 (bs), 133.6, 123.7, 110.6, 107.7, 106.7, 101.7, 92.0, 73.8, 58.7, 51.8, 28.6, 4.1. **HRMS** (ESI): m/z $[M+H]^+$ for $C_{20}H_{23}N_4O_4^+$, calcd 383.1714, found 383.1712.
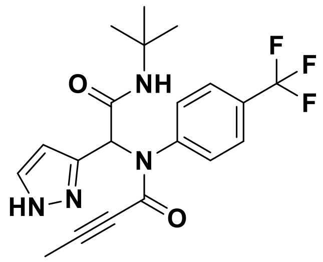	**N-(2-(tert-butylamino)-2-oxo-1-(1H-pyrazol-3-yl)ethyl)-N-(4(trifluoromethyl)phenyl)but-2-ynamide (15k).** Yield: 106 mg, 26%, light yellow powder, m.p. = 155-157 °C; **^{1}H NMR** (400 MHz, $CDCl_3$) δ 9.14 (bs, 1H), 7.49 (d, J = 8.2 Hz, 2H), 7.45 – 7.41 (m, 1H), 7.37 (d, J = 8.0 Hz, 2H), 6.71 – 6.60 (m, 1H), 6.15 – 6.11 (m, 1H), 6.06 (s, 1H), 1.66 (s, 3H), 1.29 (s, 9H). **^{13}C{^{1}H} NMR** (101 MHz, $CDCl_3$) δ 166.6, 154.4, 143.3, 143.1, 132.9, 130.3, 125.7 (q, J = 3.85 Hz), 123.8 (q, J = 272.6 Hz), 106.7, 92.5, 73.6, 59.0, 51.8, 28.5, 3.9. **HRMS** (ESI): m/z $[M+H]^+$ for $C_{20}H_{22}F_3N_4O_2^+$, calcd 407.1689, found 407.1685.

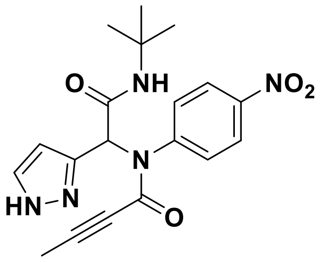	**N-(2-(tert-butylamino)-2-oxo-1-(1H-pyrazol-3-yl)ethyl)-N-(4-nitrophenyl)but-2-ynamide (15l).** Yield: 134 mg, 35%, white powder, m.p = 165-167 °C; **^{1}H NMR** (400 MHz, $CDCl_3$) δ 8.13 (d, J = 9.0 Hz, 2H), 7.51 – 7.40 (m, 3H), 6.35 (bs, 1H), 6.23 (d, J = 2.3 Hz, 1H), 6.00 (bs, 1H), 1.74 (s, 3H), 1.33 (s, 9H). **^{13}C{^{1}H} NMR** (101 MHz, $CDCl_3$) δ 166.8, 154.1, 147.2, 145.9, 144.0, 132.1, 131.1, 123.8, 106.6, 92.8, 73.6, 59.2, 52.0, 28.6, 4.0. **HRMS** (ESI): m/z $[M+H]^+$ for $C_{19}H_{22}N_5O_4^+$, calcd 384.1666, found 384.1664.
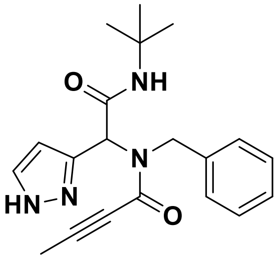	**N-benzyl-N-(2-(tert-butylamino)-2-oxo-1-(1H-pyrazol-3-yl)ethyl)but-2-ynamide (15m).** Yield: 188 mg, 53%, white powder, m.p. = 82-84 °C; **^{1}H NMR** (400 MHz, $CDCl_3$, observed as a mixture of rotamers ≈ 80:20) δ 7.49 (d, J = 2.2 Hz, 0.20H), 7.41 (d, J = 2.0 Hz, 0.80H, major), 7.25 – 7.11 (m, 5H, major + minor), 6.34 (bs, 0.20H, minor), 6.24 (d, J = 2.3 Hz, 0.20H, minor), 6.18 (bs, 0.80H, major), 6.15 (d, J = 2.1 Hz, 0.80H, major), 6.00 (s, 0.20H, minor), 5.69 (s, 0.80H, major), 4.92 (d, J = 16.2 Hz, 0.80H, major), 4.76 (d, J = 16.1 Hz, 0.80H, major), 4.62 (d, J = 15.1 Hz, 0.2H, minor), 4.58 (d, J = 15.1 Hz, 0.2H, minor), 1.96 (s, 0.60H, minor), 1.95 (s, 2.40H, major), 1.23 (s, 7.20H, major), 1.16 (s, 1.80H, minor). **^{13}C{^{1}H} NMR** (101 MHz, $CDCl_3$) δ 166.9, 156.2, 136.7, 128.6, 127.6, 106.8, 91.3, 73.5, 55.8, 52.6, 51.8, 28.6, 4.2. **HRMS** (ESI): m/z $[M+H]^+$ for $C_{20}H_{25}N_4O_2^+$, calcd 353.1972, found 353.1974.
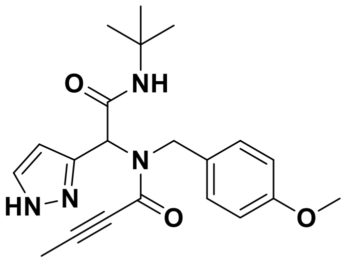	**N-(2-(tert-butylamino)-2-oxo-1-(1H-pyrazol-3-yl)ethyl)-N-(4-methoxybenzyl)but-2-ynamide (15n)** Yield: 227 mg, 59%, white powder, m.p. = 71-74 °C; **^{1}H NMR** (400 MHz, $CDCl_3$, observed as a mixture of rotamers ≈ 80:20) δ 11.07 (bs, 1H, major + minor), 7.52 (d, J = 2.2 Hz, 0.20H, minor), 7.44 (d, J = 2.0 Hz, 0.80H, major), 7.14 (d, J = 8.5 Hz, 0.40H, minor), 7.08 (d, J = 8.6 Hz, 1.60H, major), 6.82 – 6.74 (m, 2H, major + minor), 6.22 (d, J = 2.2 Hz, 0.20H, minor), 6.17 – 6.00 (m, 1.80H, major + minor), 5.91 (bs, 0.20H, minor), 5.57 (s, 0.8H, major), 4.88 (d, J = 15.8 Hz, 0.80H, major), 4.62 (d, J = 15.9 Hz, 0.80H, major), 4.59 – 4.49 (m, 0.40H, minor), 3.77 (s, 2.40H, major), 3.76 (s, 0.60H, minor), 2.00 (s, 2.40H, major), 1.95 (s, 0.60H, minor), 1.23 (s, 7.20H, major), 1.17 (s, 1.80H, minor). **^{13}C{^{1}H} NMR** (101 MHz, $CDCl_3$) δ 167.1 (minor), 167.0 (major), 158.6 (major), 158.4 (minor), 155.9 (minor), 155.6 (major), 145.4 (bs, minor), 143.8 (bs, major), 131.9 (bs, major), 130.7 (bs, minor), 129.4 (minor), 129.3 (minor), 128.8 (major), 128.5 (major), 113.5 (major), 113.4 (minor), 105.8 (major), 105.5 (minor), 90.7 (minor), 90.4 (major), 73.5 (major), 73.3 (minor), 60.9, 56.6, 54.9 (major),

	51.5, 51.2 (major), 46.8, 28.20 (major), 28.16 (minor), 3.9 (minor), 3.8 (major). **HRMS** (ESI): m/z $[M+H]^+$ for $C_{21}H_{27}N_4O_3^+$, calcd 383.2078, found 383.2079.
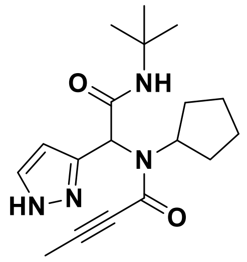	**N-(2-(tert-butylamino)-2-oxo-1-(1H-pyrazol-3-yl)ethyl)-N-cyclopentylbut-2-ynamide (15o).**<br>Yield: 126 mg, 38%, white powder, m.p. = 130-133 °C; **^{1}H NMR** (400 MHz, $CDCl_3$, observed as a mixture of rotamers ≈ 93:7, only signals belonging to the major rotamer are listed) δ 10.89 (bs, 1H), 7.54 (d, J = 2.0 Hz, 1H), 6.32 (d, J = 2.0 Hz, 1H), 5.79 (bs, 1H), 4.88 (s, 1H), 4.85 – 4.75 (m, 1H), 2.13 – 2.04 (m, 1H), 2.03 (s, 3H), 1.95 – 1.84 (m, 1H), 1.81 – 1.51 (m, 6H), 1.28 (s, 9H). **^{13}C{^{1}H} NMR** (101 MHz, $CDCl_3$, only signals belonging to the major rotamer are listed) δ 166.9, 155.1, 145.9, 132.6, 105.5, 90.4, 73.5, 61.0, 56.1, 51.5, 29.72, 29.70, 28.5, 24.2, 24.1, 4.2. **HRMS** (ESI): m/z $[M+H]^+$ for $C_{18}H_{27}N_4O_2^+$, calcd 331.2129, found 331.2121.
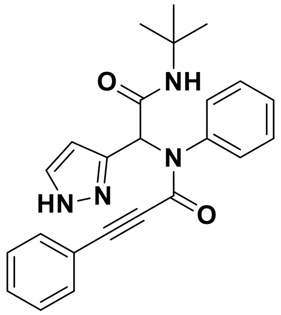	**N-(2-(tert-butylamino)-2-oxo-1-(1H-pyrazol-3-yl)ethyl)-N,3-diphenylpropiolamide (15p).**<br>Yield: 152 mg, 38%, white powder, m.p. = 214-216 °C; **^{1}H NMR** (400 MHz, $CDCl_3$) δ 7.48 (d, J = 2.1 Hz, 1H), 7.37 – 7.28 (m, 4H), 7.26 – 7.18 (m, 4H), 7.08 – 7.02 (m, 2H), 6.49 (bs, 1H), 6.28 (d, J = 2.1 Hz, 1H), 6.09 (s, 1H), 1.37 (s, 9H). **^{13}C{^{1}H} NMR** (101 MHz, $CDCl_3$) δ 166.9, 155.2, 140.1, 134.7, 132.7, 130.3, 130.0, 128.9, 128.9, 128.5, 120.2, 107.0, 82.3, 58.4, 51.9, 28.7. **HRMS** (ESI): m/z $[M+H]^+$ for $C_{24}H_{25}N_4O_2^+$, calcd 401.1972, found 401.1968.
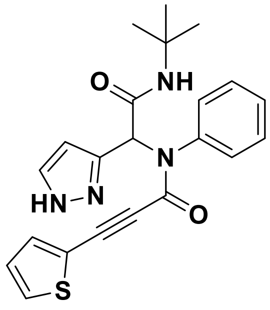	**N-(2-(tert-butylamino)-2-oxo-1-(1H-pyrazol-3-yl)ethyl)-N-phenyl-3-(thiophen-2-yl) propiolamide (15q).**<br>Yield: 154 mg, 38%, white powder, m.p. = 231-233 °C; **^{1}H NMR** (400 MHz, $CDCl_3$) δ 7.51 – 7.45 (m, 1H), 7.37 – 7.28 (m, 3H), 7.26 – 7.19 (m, 3H), 7.19 – 7.14 (m, 1H), 6.72 (d, J = 5.0 Hz, 1H), 6.44 (bs, 1H), 6.31 – 6.26 (m, 1H), 6.08 (s, 1H), 1.37 (s, 9H). **^{13}C{^{1}H} NMR** (101 MHz, DMSO-*d*6) δ 167.4, 153.3, 139.5, 133.2, 130.7, 129.3, 128.4, 127.9, 127.8, 127.5, 118.4, 105.4, 86.0, 82.6, 50.4, 28.3, 28.0. **HRMS** (ESI): m/z $[M+H]^+$ for $C_{22}H_{23}N_4O_2S^+$, calcd 407.1536, found 407.1530.
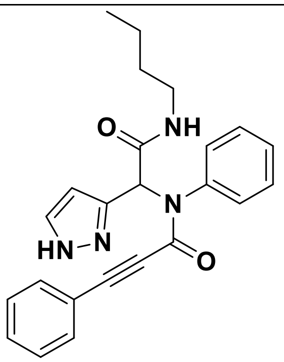	**N-(2-(butylamino)-2-oxo-1-(1H-pyrazol-3-yl)ethyl)-N,3-diphenylpropiolamide (15r).**<br>Yield: 201 mg, 50%, white powder, m.p. = 146-148 °C; **^{1}H NMR** (400 MHz, $CDCl_3$) δ 7.49 (d, J = 2.1 Hz, 1H), 7.37 – 7.27 (m, 6H), 7.24 – 7.18 (m, 2H), 7.08 – 7.02 (m, 2H), 6.65 (bs, 1H), 6.30 (d, J = 2.0 Hz, 1H), 6.11 (s, 1H), 3.36 – 3.23 (m, 2H), 1.55 – 1.44 (m, 2H), 1.38 – 1.26 (m, 2H), 0.90 (t, J = 7.3 Hz, 3H). **^{13}C{^{1}H} NMR** (101 MHz, $CDCl_3$) δ 168.0, 155.0, 143.7 (bs), 140.2, 132.5, 132.3 (bs), 130.14, 130.12, 128.7, 128.6, 128.3, 120.1, 106.5, 92.5,

	82.3, 59.0, 39.7, 31.3, 20.0, 13.7. **HRMS** (ESI): m/z $[M+H]^+$ for $C_{24}H_{25}N_4O_2^+$, calcd 401.1972, found 401.1971.
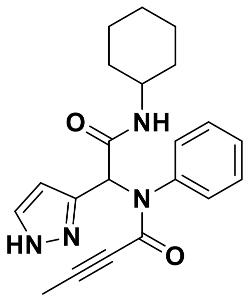	**N-(2-(cyclohexylamino)-2-oxo-1-(1H-pyrazol-3-yl)ethyl)-N-phenylbut-2-ynamide (15s).** Yield: 333 mg, 61% (1.5 mmol scale), white powder m.p. =170-172 °C; **^{1}H NMR** (500 MHz, DMSO-*d*6, observed as a mixture of rotamers ≈ 90:10, only signals belonging to the major rotamer are listed) δ 12.73 (bs, 1H), 7.99 (bd, J = 7.8 Hz, 1H), 7.37 (bs, 1H), 7.29 – 7.07 (m, 5H), 6.02 (s, 1H), 5.72 (d, J = 2.2 Hz, 1H), 3.61 – 3.52 (m, 1H), 1.75 – 1.48 (m, 8H), 1.33 – 0.95 (m, 5H). **^{13}C{^{1}H} NMR** (126 MHz, DMSO-*d*6) δ 167.3, 153.3, 139.4, 130.5, 127.8, 127.7, 105.5, 90.2, 74.4, 57.9, 48.0, 32.2, 32.1, 25.2, 24.6, 24.5, 3.2.
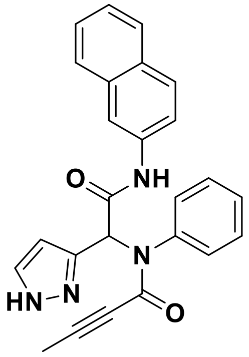	**N-(2-(naphthalen-2-ylamino)-2-oxo-1-(1H-pyrazol-3-yl)ethyl)-N-phenylbut-2-ynamide (15t).** Yield: 177 mg, 43%, white powder, m.p. = 103-105 °C; **^{1}H NMR** (400 MHz, $CDCl_3$) δ 9.11 (bs, 1H), 8.29 – 8.21 (m, 1H), 7.82 – 7.70 (m, 3H), 7.49 (d, J = 2.2 Hz, 1H), 7.48 – 7.35 (m, 3H), 7.32 – 7.23 (m, 5H), 6.32 (s, 1H), 6.30 (d, J = 2.3 Hz, 1H), 1.70 (s, 3H). **^{13}C{^{1}H} NMR** (101 MHz, $CDCl_3$) δ 167.2, 155.4, 143.2, 139.6, 135.6, 133.9, 132.5, 130.7, 130.2, 128.7, 128.6, 128.6, 127.8, 127.5, 126.4, 125.0, 120.2, 116.8, 106.9, 92.4, 73.7, 59.5, 3.9. **HRMS** (ESI): m/z $[M+H]^+$ for $C_{25}H_{21}N_4O_2^+$, calcd 409.1659, found 409.1661.
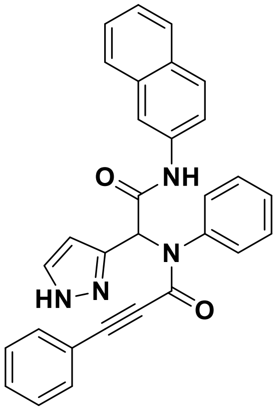	**N-(2-(naphthalen-2-ylamino)-2-oxo-1-(1H-pyrazol-3-yl)ethyl)-N,3-diphenylpropiol amide (15u).** Yield: 145 mg, 31%, white powder, m.p. = 186-189 °C; **^{1}H NMR** (400 MHz, DMSO-*d*6) δ 12.85 (bs, 1H), 10.61 (s, 1H), 8.47 (s, 1H), 7.96 – 7.77 (m, 3H), 7.70 – 7.61 (m, 1H), 7.58 – 7.24 (m, 11H), 7.14 – 7.03 (m, 2H), 6.46 (s, 1H), 6.05 – 5.94 (m, 1H). **^{13}C{^{1}H} NMR** (101 MHz, $CDCl_3$) δ 167.7, 153.6, 144.0 (bs), 139.3, 136.6, 133.5, 132.0, 130.9, 130.5, 129.8, 128.8, 128.4, 128.1, 127.5, 127.4, 126.5, 124.7, 119.9, 119.4, 115.4, 105.9, 90.6, 82.8, 59.2 (bs). **HRMS** (ESI): m/z $[M+H]^+$ for $C_{30}H_{23}N_4O_2^+$, calcd 471.1816, found 471.1825.
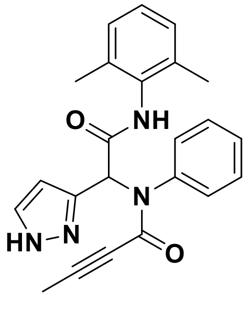	**N-(2-((2,6-dimethylphenyl)amino)-2-oxo-1-(1H-pyrazol-3-yl)ethyl)-N-phenylbut-2-ynamide (15v).** Yield: 141 mg, 36%, white powder, m.p. = 169-173 °C; **^{1}H NMR** (400 MHz, $CDCl_3$) δ 8.03 (bs, 1H), 7.48 (d, J = 2.2 Hz, 1H), 7.31 – 7.26 (m, 5H), 7.10 – 7.01 (m, 3H), 6.34 (d, J = 2.1 Hz, 1H), 6.18 (bs, 1H), 2.18 (s, 6H), 1.71 (s, 3H). **^{13}C{^{1}H} NMR** (101 MHz, $CDCl_3$) δ 166.8, 155.2, 140.4, 135.6, 133.7, 132.7 (bs), 129.9, 128.8, 128.5, 128.1, 127.4, 106.7, 91.9, 73.9, 59.3, 18.4,

	4.0. **HRMS** (ESI): m/z $[M+H]^+$ for $C_{23}H_{23}N_4O_2^+$, calcd 387.1816, found 387.1816.
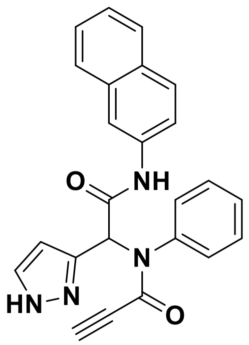	**N-(2-(naphthalen-2-ylamino)-2-oxo-1-(1H-pyrazol-3-yl)ethyl)-N-phenylpropiolamide (15w)**<br>Yield: 280 mg, 47% (1.5 mmol scale), brownish powder m.p. = 165-167 °C; **^{1}H NMR** (500 MHz, DMSO-*d6*) δ 12.75 (bs, 1H), 10.51 (bs, 1H), 8.42 – 8.36 (m, 1H), 7.90 – 7.79 (m, 3H), 7.55 (dd, J = 8.8, 2.1 Hz, 1H), 7.52 – 7.30 (m, 5H), 7.29 – 7.18 (m, 3H), 6.28 (bs, 1H), 5.88 (bs, 1H), 4.24 (s, 1H). **^{13}C{^{1}H} NMR** (126 MHz, DMSO-*d6*) δ 167.6, 152.8, 144.3 (bs), 138.8, 136.6, 133.5, 130.8, 129.8, 129.2 (bs), 128.4, 128.2, 128.1, 127.5, 127.4, 126.5, 124.7, 119.8, 115.3, 105.8 (bs), 83.4, 76.6, 59.5.
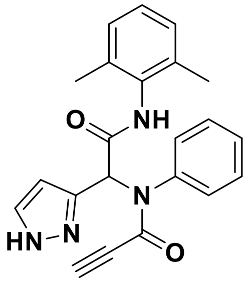	**N-(2-((2,6-dimethylphenyl)amino)-2-oxo-1-(1H-pyrazol-3-yl)ethyl)-N-phenylpropiol amide (15x).**<br>Yield: 120 mg, 32%, white powder, m.p. = 173-175 °C; **^{1}H NMR** (400 MHz, $CDCl_3$) δ 8.35 (bs, 1H), 7.37 (d, J = 2.3 Hz, 1H), 7.32 – 7.23 (m, 5H), 7.06 – 6.96 (m, 3H), 6.26 (bs, 1H), 6.21 (d, J = 2.3 Hz, 1H), 2.81 (s, 1H), 2.11 (s, 6H). **^{13}C{^{1}H} NMR** (101 MHz, $CDCl_3$) δ 166.6, 153.8, 143.9, 139.7, 135.6, 133.5, 132.0, 130.1, 128.9, 128.2, 127.4, 106.7, 81.0, 75.9, 59.5, 18.4. **HRMS** (ESI): m/z $[M+H]^+$ for $C_{22}H_{21}N_4O_2^+$, calcd 373.1659, found 373.1665.
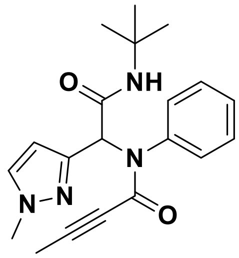	**N-(2-(tert-butylamino)-1-(1-methyl-1H-pyrazol-3-yl)-2-oxoethyl)-N-phenylbut-2-ynamide (15y)**<br>Yield: 151 mg, 43%, white powder, m.p. = 183-184 °C; **^{1}H NMR** (400 MHz, $CDCl_3$) δ 7.32 – 7.25 (m, 5H), 7.20 (d, J = 2.3 Hz, 1H), 6.68 (bs, 1H), 6.09 (d, J = 2.2 Hz, 1H), 5.84 (s, 1H), 3.84 (s, 3H), 1.68 (s, 3H), 1.33 (s, 9H). **^{13}C{^{1}H} NMR** (101 MHz, $CDCl_3$) δ 166.9, 154.6, 147.1, 140.9, 131.0, 129.8, 128.5, 128.1, 107.0, 90.8, 74.2, 60.8, 51.5, 39.0, 28.6, 3.9. **HRMS** (ESI): m/z $[M+H]^+$ for $C_{20}H_{25}N_4O_2^+$, calcd 353.1972, found 353.1974.
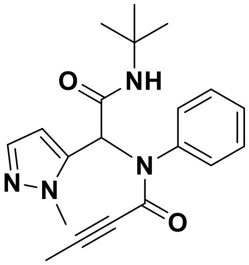	**N-(2-(tert-butylamino)-1-(1-methyl-1H-pyrazol-5-yl)-2-oxoethyl)-N-phenylbut-2-ynamide (15z)**<br>Yield: 105 mg, 30%, white powder, m.p. = 188-189 °C; **^{1}H NMR** (400 MHz, $CDCl_3$) δ 7.28 – 7.21 (m, 4H), 7.15 – 7.01 (m, 2H), 6.30 (s, 1H), 6.07 (d, J = 2.0 Hz, 1H), 5.79 (bs, 1H), 3.78 (s, 3H), 1.70 (s, 3H), 1.37 (s, 9H). **^{13}C{^{1}H} NMR** (101 MHz, $CDCl_3$) δ 165.9, 155.2, 138.5, 135.5, 129.9, 128.7, 128.6, 109.1, 92.5, 73.6, 55.2, 52.1, 36.8, 28.7, 4.0. **HRMS** (ESI): m/z $[M+H]^+$ for $C_{20}H_{25}N_4O_2^+$, calcd 353.1972, found 353.1970.

General procedure for the synthesis of pyrazolodiazepines 16:

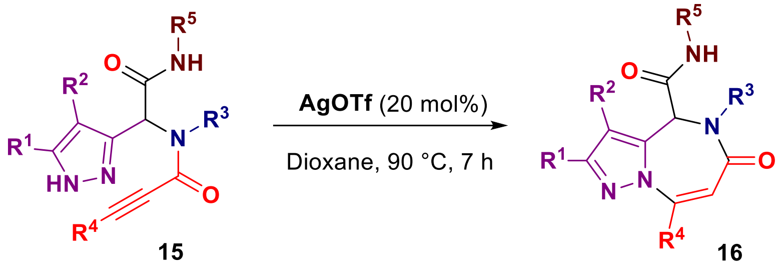

A mixture of Ugi adduct **15** (0.2 mmol, 1 equiv), Ag(OTf) catalyst (10.2 mg, 20 mol%) were taken in a screw cap vial, followed by the addition of 1,4-dioxane (1.0 mL). The reaction mixture was then stirred for 7 hours at 90 °C. The reaction was monitored using thin layer chromatography (TLC). After completion of the reaction, the reaction vial was cooled down to room temperature and the resulting mixture was diluted with EtOAc. After dilution silica gel was added, and then solvent was removed under reduced pressure. Column chromatography was performed using a petroleum ether/ethyl acetate mixture as the eluent (the ratio was adjusted based on TLC analysis), affording pyrazolodiazepine **16**. Most products **16** were additionally purified by consecutive trituration with n-hexane and diethyl ether.

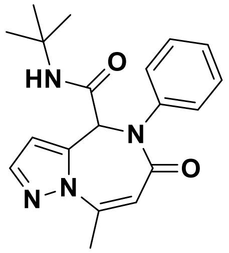	**N-(tert-butyl)-8-methyl-6-oxo-5-phenyl-5,6-dihydro-4H-pyrazolo[1,5-a][1,4]diazepine-4-carboxamide (16a).** No trituration was performed after column chromatography. Yield: 64 mg, 94%, 1.0 g, 84% (3.5 mmol scale); white powder, m.p. = 181-183 °C; **^{1}H NMR** (400 MHz, $CDCl_3$) δ 7.71 (d, J = 1.7 Hz, 1H), 7.42 – 7.36 (m, 2H), 7.35 – 7.26 (m, 3H), 6.40 (d, J = 1.7 Hz, 1H), 5.87 (q, J = 1.2 Hz, 1H), 5.34 (s, 1H), 5.20 (bs, 1H), 2.43 (d, J = 1.0 Hz, 3H), 1.27 (s, 9H). **$^{13}C\{^1H\}$ NMR** (101 MHz, $CDCl_3$) δ 164.74, 164.67, 143.1, 141.7, 140.6, 139.1, 129.6, 127.7, 126.6, 112.0, 107.8, 60.6, 52.3, 28.5, 20.8. **HRMS** (ESI): m/z $[M+H]^+$ for $C_{19}H_{23}N_4O_2^+$, calcd 339.1816, found 339.1816.
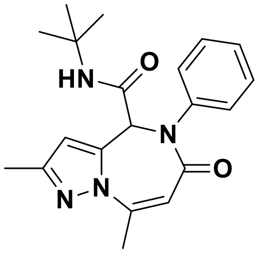	**N-butyl-6-oxo-5,8-diphenyl-5,6-dihydro-4H-pyrazolo[1,5-a][1,4]diazepine-4-carboxamide (16b).** Yield: 60 mg, 85 %, white powder, m.p. = 163-165 °C; **^{1}H NMR** (400 MHz, $CDCl_3$) δ 7.44 – 7.37 (m, 2H), 7.36 – 7.27 (m, 3H), 6.20 (s, 1H), 5.79 (q, J = 1.0 Hz, 1H), 5.27 (s, 1H), 5.26 (bs, 1H), 2.41 (d, J = 1.0 Hz, 3H), 2.35 (s, 3H), 1.29 (s, 9H). **$^{13}C\{^1H\}$ NMR** (101 MHz, $CDCl_3$) δ 165.0, 164.8, 151.3, 143.3, 140.9, 139.8, 129.6, 127.7, 126.6, 111.0, 108.0, 60.8, 52.3, 28.6, 21.0, 14.0. **HRMS** (ESI): m/z $[M+H]^+$ for $C_{20}H_{25}N_4O_2^+$, calcd 353.1972, found 353.1973.

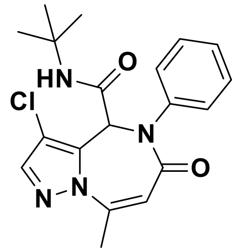	**N-(tert-butyl)-3-chloro-8-methyl-6-oxo-5-phenyl-5,6-dihydro-4H-pyrazolo[1,5-a][1,4]diazepine-4-carboxamide (16c).** No trituration was performed after column chromatography. Yield: 173 mg, 93% (0.5 mmol scale); white solid, m.p. = 182-183 °C; **^{1}H NMR** (500 MHz, $CDCl_3$) δ 7.73 (s, 1H), 7.48 – 7.42 (m, 2H), 7.36 – 7.31 (m, 3H), 5.89 (q, J = 1.1 Hz, 1H), 5.46 (s, 1H), 5.34 (bs, 1H), 2.42 (d, J = 1.1 Hz, 3H), 1.31 (s, 9H). **^{13}C{^{1}H} NMR** (126 MHz, $CDCl_3$) δ 164.3, 164.1, 142.7, 140.7, 139.8, 136.3, 129.8, 127.9, 126.3, 112.2, 111.4, 58.2, 52.6, 28.6, 20.2. **HRMS** (ESI): m/z $[M+H]^+$ for $C_{19}H_{22}ClN_4O_2^+$, calcd 373.1426, found 373.1422.
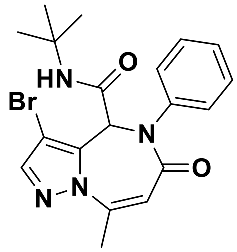	**3-bromo-N-(tert-butyl)-8-methyl-6-oxo-5-phenyl-5,6-dihydro-4H-pyrazolo[1,5-a][1,4]diazepine-4-carboxamide (16d).** No trituration was performed after column chromatography. Yield: 201 mg, 96% (0.5 mmol scale); white solid, m.p. = 205-206 °C; **^{1}H NMR** (500 MHz, $CDCl_3$) δ 7.75 (s, 1H), 7.48 – 7.41 (m, 2H), 7.37 – 7.31 (m, 3H), 5.90 (q, J = 1.2 Hz, 1H), 5.45 (s, 1H), 5.29 (bs, 1H), 2.42 (d, J = 1.1 Hz, 3H), 1.31 (s, 9H). **^{13}C{^{1}H} NMR** (126 MHz, $CDCl_3$) δ 164.2, 164.0, 142.8, 141.7, 140.6, 138.1, 129.7, 127.9, 126.4, 112.3, 96.3, 59.0, 52.6, 28.6, 20.3. **HRMS** (ESI): m/z $[M+H]^+$ for $C_{19}H_{22}BrN_4O_2^+$, calcd 417.0921, found 417.0921.
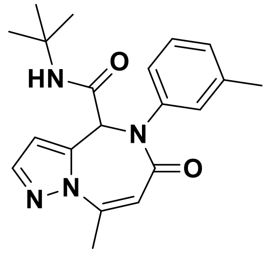	**N-(tert-butyl)-8-methyl-6-oxo-5-(m-tolyl)-5,6-dihydro-4H-pyrazolo[1,5-a][1,4]diazepine-4-carboxamide (16e).** Yield: 65 mg, 92%, white powder, m.p. = 189-192 °C; **^{1}H NMR** (400 MHz, $CDCl_3$) δ 7.72 (d, J = 1.7 Hz, 1H), 7.31 – 7.26 (m, 1H), 7.16 – 7.08 (m, 3H), 6.41 (d, J = 1.7 Hz, 1H), 5.87 (q, J = 1.1 Hz, 1H), 5.32 (s, 1H), 5.22 (bs, 1H), 2.44 (d, J = 1.1 Hz, 3H), 2.35 (s, 3H), 1.28 (s, 9H). **^{13}C{^{1}H} NMR** (101 MHz, $CDCl_3$) δ 164.8, 164.7, 143.1, 141.7, 140.6, 139.7, 139.2, 129.5, 128.6, 127.3, 123.4, 112.1, 107.8, 60.7, 52.3, 28.6, 21.5, 20.9. **HRMS** (ESI): m/z $[M+H]^+$ for $C_{20}H_{25}N_4O_2^+$, calcd 353.1972, found 353.1972.
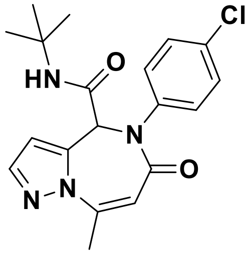	**N-(tert-butyl)-5-(4-chlorophenyl)-8-methyl-6-oxo-5,6-dihydro-4H-pyrazolo[1,5-a][1,4]diazepine-4-carboxamide (16f).** Yield: 73 mg, 98%, white powder, m.p. = 198-201 °C; **^{1}H NMR** (400 MHz, $CDCl_3$) δ 7.75 (d, J = 1.7 Hz, 1H), 7.37 (d, J = 8.8 Hz, 2H), 7.31 (d, J = 8.8 Hz, 2H), 6.44 (d, J = 1.8 Hz, 1H), 5.90 – 5.84 (m, 1H), 5.32 (s, 1H), 5.05 (bs, 1H), 2.46 (d, J = 1.1 Hz, 3H), 1.27 (s, 9H). **^{13}C{^{1}H} NMR** (101 MHz, $CDCl_3$) δ 164.7, 164.5, 141.8, 141.6, 140.5, 138.8, 133.3, 129.6, 128.2, 112.0, 107.8, 60.4, 52.3, 28.5, 20.9. **HRMS** (ESI): m/z $[M+H]^+$ for $C_{19}H_{22}ClN_4O_2^+$, calcd 373.1426, found 373.1424.

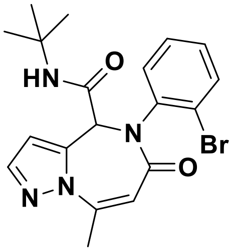	**5-(2-bromophenyl)-N-(tert-butyl)-8-methyl-6-oxo-5,6-dihydro-4H-pyrazolo[1,5-a][1,4]diazepine-4-carboxamide (16g).** Yield: 72 mg, 86%, white powder, m.p. = 179-183 °C; **^{1}H NMR** (400 MHz, $CDCl_3$, observed as a mixture of rotamers ≈ 67:33) δ 7.81 (dd, J = 8.0, 1.7 Hz, 0.67H, major), 7.75 – 7.72 (m, 1H, major + minor), 7.70 (dd, J = 8.0, 1.5 Hz, 0.33H, minor), 7.59 (dd, J = 8.0, 1.4 Hz, 0.67H, major), 7.45 – 7.39 (m, 1H, major + minor), 7.36 – 7.31 (m, 0.33H, minor), 7.28 – 7.23 (m, 0.33H, minor), 7.23 – 7.18 (m, 0.67H, major), 7.06 (dd, J = 7.8, 1.6 Hz, 0.33H, minor), 6.45 (d, J = 1.7 Hz, 0.67H, major), 6.42 (d, J = 1.7 Hz, 0.33H, minor), 5.90 – 5.87 (m, 0.67H, major), 5.86 – 5.84 (m, 0.33H, minor), 5.20 (s, 0.33H, minor), 5.14 (s, 0.67H, major), 4.93 (bs, 0.67H, major), 2.49 – 2.43 (m, 3H, major + minor), 1.38 (s, 3H, minor), 1.25 (s, 6H, major). **^{13}C{^{1}H} NMR** (101 MHz, $CDCl_3$) δ 164.77 (minor), 164.73 (major), 164.45 (major), 164.39 (minor), 143.1 (minor), 142.4 (major), 141.8 (minor), 141.7 (major), 141.6 (minor), 140.7 (major), 140.4 (minor), 139.1 (major), 133.7 (minor), 133.2 (major), 130.7 (major), 130.1 (minor), 129.7 (major), 129.5 (minor), 128.9 (major), 128.4 (minor), 122.9 (major), 122.4 (minor), 111.6 (major), 110.2 (minor), 108.6 (major), 108.1 (minor), 60.9 (minor), 60.1 (major), 52.9 (minor), 52.2 (major), 28.52 (major), 28.47 (minor), 21.1 (major), 20.7 (minor). **HRMS** (ESI): m/z $[M+H]^+$ for $C_{19}H_{22}BrN_4O_2^+$, calcd 417.0921, found 417.0920.
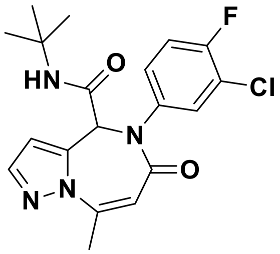	**N-(tert-butyl)-5-(3-chloro-4-fluorophenyl)-8-methyl-6-oxo-5,6-dihydro-4H-pyrazolo[1,5-a][1,4]diazepine-4-carboxamide (16h).** Yield: 56 mg, 72%, white powder, m.p. = 228-231 °C; **^{1}H NMR** (400 MHz, $CDCl_3$) δ 7.75 (d, J = 1.7 Hz, 1H), 7.45 (dd, J = 6.6, 2.6 Hz, 1H), 7.27 (ddd, J = 8.7, 4.3, 2.6 Hz, 1H), 7.15 (t, J = 8.7 Hz, 1H), 6.44 (d, J = 1.7 Hz, 1H), 5.89 – 5.86 (m, 1H), 5.25 (s, 1H), 4.92 (bs, 1H), 2.44 (d, J = 1.2 Hz, 3H), 1.26 (s, 9H). **^{13}C{^{1}H} NMR** (101 MHz, $CDCl_3$) δ 164.7, 164.3, 157.2 (d, J = 249.8 Hz), 141.8, 140.5, 139.6 (d, J = 3.8 Hz), 138.5, 129.6, 127.1 (d, J = 7.5 Hz), 121.4 (d, J = 18.9 Hz), 117.1 (d, J = 22.3 Hz), 111.9, 107.9, 60.3, 52.3, 28.4, 20.9. **HRMS** (ESI): m/z $[M+H]^+$ for $C_{19}H_{21}ClFN_4O_2^+$, calcd 391.1332, found 391.1337.
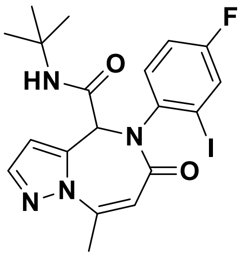	**N-(tert-butyl)-5-(4-fluoro-2-iodophenyl)-8-methyl-6-oxo-5,6-dihydro-4H-pyrazolo[1,5-a][1,4]diazepine-4-carboxamide (16i).** Yield: 82 mg, 85%, white powder, m.p. = 191-193 °C; **^{1}H NMR** (400 MHz, DMSO-*d*6, observed as a mixture of rotamers ≈ 95:5, only signals belonging to the major rotamer are listed) δ 7.78 (d, J = 1.6 Hz, 1H), 7.76 (dd, J = 8.1, 2.7 Hz, 1H), 7.44 (dd, J = 8.8, 5.7 Hz, 1H), 7.39 (td, J = 8.5, 2.8 Hz, 1H), 6.62 (d, J = 1.7 Hz, 1H), 6.17 (bs, 1H), 5.81 – 5.77 (m, 1H), 5.61 (s, 1H), 2.37 (d, J = 1.2 Hz, 3H), 1.18 (s, 9H). **^{13}C{^{1}H} NMR** (101 MHz, DMSO-

	*d*6) δ 165.1, 164.8, 160.5 (d, J = 249.3 Hz), 143.3 (d, J = 3.1 Hz), 141.5, 139.9, 139.8, 130.1 (d, J = 8.9 Hz), 125.4 (d, J = 24.3 Hz), 116.5 (d, J = 22.1 Hz), 111.2, 109.1, 100.9 (d, J = 8.4 Hz), 59.3, 51.3, 28.2, 20.6. **HRMS** (ESI): m/z $[M+H]^+$ for $C_{19}H_{21}FIN_4O_2^+$, calcd 483.0688, found 483.0688.
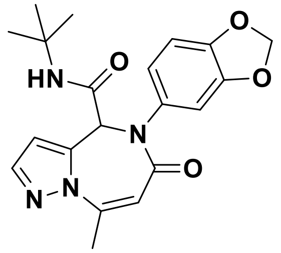	**5-(benzo[d][1,3]dioxol-5-yl)-N-(tert-butyl)-8-methyl-6-oxo-5,6-dihydro-4H-pyrazolo[1,5-a][1,4]diazepine-4-carboxamide (16j).** Yield: 67 mg, 87%, white powder, m.p. = 181-183 °C; **^{1}H NMR** (400 MHz, $CDCl_3$) δ 7.73 (d, J = 1.7 Hz, 1H), 6.88 – 6.85 (m, 1H), 6.82 – 6.76 (m, 2H), 6.41 (d, J = 1.7 Hz, 1H), 5.98 (s, 2H), 5.89 – 5.86 (m, 1H), 5.26 (s, 1H), 5.08 (bs, 1H), 2.43 (d, J = 1.1 Hz, 3H), 1.27 (s, 9H). **^{13}C{^{1}H} NMR** (101 MHz, $CDCl_3$) δ 164.9, 164.7, 148.2, 147.1, 141.7, 140.4, 139.0, 137.2, 120.0, 112.2, 108.6, 108.4, 107.7, 101.8, 61.0, 52.3, 28.5, 20.8. **HRMS** (ESI): m/z $[M+H]^+$ for $C_{20}H_{23}N_4O_4^+$, calcd 383.1714, found 383.1706.
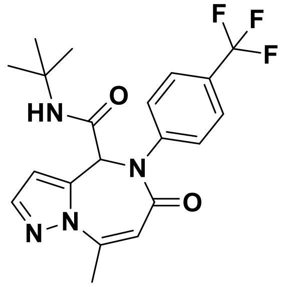	**N-(tert-butyl)-8-methyl-6-oxo-5-(4-(trifluoromethyl)phenyl)-5,6-dihydro-4H-pyrazolo[1,5-a][1,4]diazepine-4-carboxamide (16k).** Yield: 69 mg, 85%, light brown powder, m.p. = 181-183 °C; **^{1}H NMR** (400 MHz, $CDCl_3$) δ 7.76 (d, J = 1.7 Hz, 1H), 7.67 (d, J = 8.4 Hz, 2H), 7.50 (d, J = 8.3 Hz, 2H), 6.44 (d, J = 1.7 Hz, 1H), 5.94 – 5.86 (m, 1H), 5.33 (s, 1H), 5.00 (bs, 1H), 2.46 (d, J = 1.1 Hz, 3H), 1.27 (s, 9H). **^{13}C{^{1}H} NMR** (101 MHz, $CDCl_3$) δ 164.5, 164.3, 146.1, 141.8, 140.6, 138.6, 129.3 (q, J = 32.8 Hz), 127.2, 126.4 (q, J = 3.7 Hz), 123.8 (q, J = 272.2 Hz), 111.9, 107.9, 60.0, 52.3, 28.4, 20.8. **HRMS** (ESI): m/z $[M+H]^+$ for $C_{20}H_{22}F_3N_4O_2^+$, calcd 407.1689, found 407.1688.
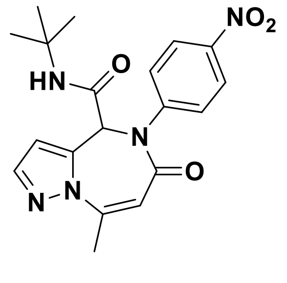	**N-(tert-butyl)-8-methyl-5-(4-nitrophenyl)-6-oxo-5,6-dihydro-4H-pyrazolo[1,5-a][1,4]diazepine-4-carboxamide (16l).** Yield: 74 mg, 96%, white powder, m.p. = 230-233 °C; **^{1}H NMR** (400 MHz, $CDCl_3$) δ 8.26 (d, J = 9.0 Hz, 2H), 7.77 (d, J = 1.7 Hz, 1H), 7.57 (d, J = 9.0 Hz, 2H), 6.48 (d, J = 1.7 Hz, 1H), 5.92 – 5.87 (m, 1H), 5.38 (s, 1H), 4.93 (bs, 1H), 2.47 (d, J = 1.1 Hz, 3H), 1.27 (s, 9H). **^{13}C{^{1}H} NMR** (101 MHz, $CDCl_3$) δ 164.4, 164.2, 148.6, 146.4, 142.0, 141.0, 138.3, 127.6, 124.9, 111.9, 108.2, 60.0, 52.6, 28.6, 21.0. **HRMS** (ESI): m/z $[M+H]^+$ for $C_{19}H_{22}N_5O_4^+$, calcd 384.1666, found 384.1671.
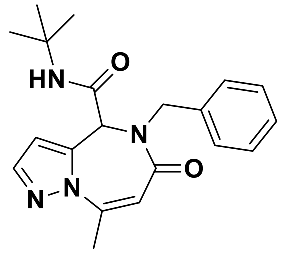	**5-benzyl-N-(tert-butyl)-8-methyl-6-oxo-5,6-dihydro-4H-pyrazolo[1,5-a][1,4]diazepine-4-carboxamide (16m).** Yield: 57 mg, 80%, white powder, m.p. = 198-201 °C; **^{1}H NMR** (400 MHz, $CDCl_3$) δ. 7.63 (d, J = 1.7 Hz, 1H), 7.41 – 7.34 (m, 5H), 6.18 (d, J = 1.7 Hz, 1H), 5.79 – 5.75 (m, 1H), 5.06 (bs, 1H), 5.03 (d, J = 14.5 Hz, 1H), 4.96 (s, 1H), 4.47 (d, J = 14.5 Hz, 1H), 2.40 (d, J = 1.1 Hz, 3H), 1.08 (s, 9H). **^{13}C{^{1}H} NMR** (101 MHz, $CDCl_3$) δ 165.4, 165.0, 141.7, 141.5, 139.2, 136.5, 129.5,

	129.2, 128.6, 110.9, 107.7, 56.8, 52.10, 52.06, 28.3, 20.9. **HRMS** (ESI): m/z $[M+H]^+$ for $C_{20}H_{25}N_4O_2^+$, calcd 353.1972, found 353.1979.
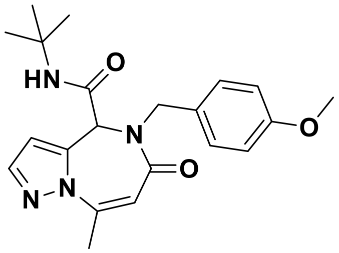	**N-(tert-butyl)-5-(4-methoxybenzyl)-8-methyl-6-oxo-5,6-dihydro-4H-pyrazolo[1,5-a][1,4]diazepine-4-carboxamide (16n).** Yield: 68 mg, 87%, white powder, m.p. = 194-197 °C; **^{1}H NMR** (400 MHz, $CDCl_3$) δ 7.63 (d, J = 1.7 Hz, 1H), 7.32 (d, J = 8.6 Hz, 2H), 6.89 (d, J = 8.6 Hz, 2H), 6.19 (d, J = 1.7 Hz, 1H), 5.78 – 5.72 (m, 1H), 5.07 (bs, 1H), 4.99 (d, J = 14.4 Hz, 1H), 4.96 (s, 1H), 4.35 (d, J = 14.4 Hz, 1H), 3.81 (s, 3H), 2.39 (d, J = 1.1 Hz, 3H), 1.08 (s, 9H). **$^{13}C\{^{1}H\}$ NMR** (101 MHz, $CDCl_3$) δ 165.2, 165.1, 159.9, 141.7, 141.4, 139.3, 130.5, 128.5, 114.8, 110.8, 107.6, 56.6, 55.5, 52.0, 51.4, 28.3, 20.8. **HRMS** (ESI): m/z $[M+H]^+$ for $C_{21}H_{27}N_4O_3^+$, calcd 383.2078, found 383.2077.
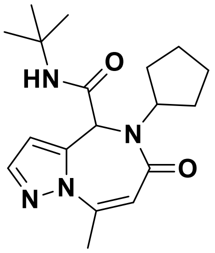	**N-(tert-butyl)-5-cyclopentyl-8-methyl-6-oxo-5,6-dihydro-4H-pyrazolo[1,5-a][1,4]diazepine-4-carboxamide(16o).** Yield: 59 mg, 89%, white powder, m.p. = 177-179 °C; **^{1}H NMR** (400 MHz, $CDCl_3$) δ 7.67 (d, J = 1.7 Hz, 1H), 6.36 (d, J = 1.7 Hz, 1H), 5.77 – 5.70 (m, 1H), 5.37 (bs, 1H), 5.11 – 5.01 (m, 1H), 5.01 (s, 1H), 2.37 (d, J = 1.1 Hz, 3H), 2.09 – 1.98 (m, 1H), 1.83 – 1.40 (m, 7H), 1.27 (s, 9H). **$^{13}C\{^{1}H\}$ NMR** (101 MHz, $CDCl_3$) δ 165.6, 165.3, 141.5, 140.5, 140.3, 111.8, 107.2, 55.6, 52.5, 52.3, 29.2, 29.1, 28.5, 23.9, 23.8, 20.6. **HRMS** (ESI): m/z $[M+H]^+$ for $C_{18}H_{27}N_4O_2^+$, calcd 331.2129, found 331.2121.
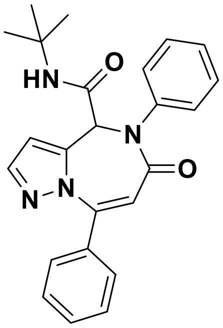	**N-(tert-butyl)-6-oxo-5,8-diphenyl-5,6-dihydro-4H-pyrazolo[1,5-a][1,4]diazepine-4-carboxamide(16p).** Yield: 55 mg, 69%, white powder, m.p. = 197-201 °C; **^{1}H NMR** (400 MHz, $CDCl_3$) δ 7.75 (d, J = 1.7 Hz, 1H), 7.50 – 7.40 (m, 7H), 7.39 – 7.31 (m, 3H), 6.49 (d, J = 1.8 Hz, 1H), 6.17 (s, 1H), 5.52 (bs, 1H), 5.46 (s, 1H), 1.23 (s, 9H). **$^{13}C\{^{1}H\}$ NMR** (101 MHz, $CDCl_3$) δ 165.0, 164.9, 143.0, 142.9, 142.2, 140.5, 134.7, 130.6, 129.8, 129.0, 128.6, 127.9, 126.5, 113.6, 107.8, 61.0, 52.6, 28.5. **HRMS** (ESI): m/z $[M+H]^+$ for $C_{24}H_{25}N_4O_2^+$, calcd 401.1972, found 401.1969.
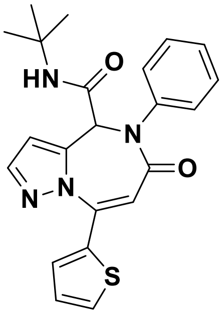	**N-(tert-butyl)-6-oxo-5-phenyl-8-(thiophen-2-yl)-5,6-dihydro-4H-pyrazolo[1,5-a][1,4]diazepine-4-carboxamide (16q).** Yield: 41 mg, 50%, white powder, m.p. = 229-231 °C; **^{1}H NMR** (400 MHz, $CDCl_3$) δ 7.77 (d, J = 1.7 Hz, 1H), 7.72 (dd, J = 3.0, 1.3 Hz, 1H), 7.46 – 7.40 (m, 2H), 7.40 – 7.30 (m, 4H), 7.19 (dd, J = 5.1, 1.3 Hz, 1H), 6.48 (d, J = 1.8 Hz, 1H), 6.28 (s, 1H), 5.42 (s, 1H), 5.35 (bs, 1H), 1.18 (s, 9H). **$^{13}C\{^{1}H\}$ NMR** (101 MHz, $CDCl_3$) δ 165.0, 164.8, 143.0, 142.0, 140.3, 137.6, 135.3, 129.7, 127.92, 127.85, 127.3, 126.6, 126.4, 112.5, 107.7, 60.9, 52.5, 28.5. **HRMS** (ESI): m/z $[M+H]^+$ for $C_{22}H_{23}N_4O_2S^+$, calcd 407.1536, found 407.1537.

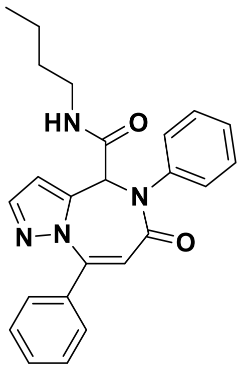	**N-butyl-6-oxo-5,8-diphenyl-5,6-dihydro-4H-pyrazolo[1,5-a][1,4]diazepine-4-carboxamide (16r).** Yield: 47 mg, 59%, white powder, m.p. = 210-213 °C; **^{1}H NMR** (400 MHz, $CDCl_3$) δ 7.75 (d, J = 1.7 Hz, 1H), 7.48 – 7.41 (m, 7H), 7.39 – 7.31 (m, 3H), 6.49 (d, J = 1.7 Hz, 1H), 6.14 (s, 1H), 5.86 (bt, J = 5.8 Hz, 1H), 5.53 (s, 1H), 3.36 – 3.16 (m, 2H), 1.41 – 1.30 (m, 2H), 1.24 – 1.14 (m, 2H), 0.77 (t, J = 7.3 Hz, 3H). **^{13}C{^{1}H} NMR** (101 MHz, $CDCl_3$) δ 165.9, 165.1, 143.0, 142.9, 142.2, 140.2, 134.5, 130.5, 129.7, 129.0, 128.5, 127.9, 126.5, 113.4, 108.0, 60.7, 40.3, 31.7, 20.0, 13.6. **HRMS** (ESI): m/z $[M+H]^+$ for $C_{24}H_{25}N_4O_2^+$, calcd 401.1972, found 401.1966.
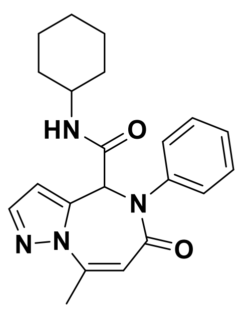	**N-cyclohexyl-8-methyl-6-oxo-5-phenyl-5,6-dihydro-4H-pyrazolo[1,5-a][1,4]diazepine-4-carboxamide (16s).** Yield: 143 mg, 78% (0.5 mmol scale), white powder, m.p. = 188-190 °C; **^{1}H NMR** (500 MHz, $CDCl_3$) δ 7.74 (d, J = 1.7 Hz, 1H), 7.44 – 7.38 (m, 2H), 7.37 – 7.33 (m, 2H), 7.33 – 7.28 (m, 1H), 6.43 (d, J = 1.7 Hz, 1H), 5.88 (q, J = 1.0 Hz, 1H), 5.40 (s, 1H), 5.21 (bd, J = 8.4 Hz, 1H), 3.85 – 3.75 (m, 1H), 2.43 (d, J = 1.1 Hz, 3H), 1.86 – 1.76 (m, 2H), 1.68 – 1.55 (m, 3H), 1.40 – 1.27 (m, 2H), 1.17 – 0.92 (m, 3H). **^{13}C{^{1}H} NMR** (126 MHz, $CDCl_3$) δ 164.8, 164.6, 143.1, 141.7, 140.4, 138.7, 129.5, 127.7, 126.7, 112.1, 107.9, 60.2, 49.2, 33.0, 32.7, 25.3, 24.74, 24.71, 20.9. **HRMS** (ESI): m/z $[M+H]^+$ for $C_{21}H_{25}N_4O_2^+$, calcd 365.1972, found 365.1976.
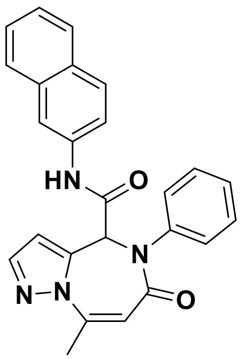	**8-methyl-N-(naphthalen-2-yl)-6-oxo-5-phenyl-5,6-dihydro-4H-pyrazolo[1,5-a][1,4]diazepine-4-carboxamide (16t).** Yield**:** 61 mg, 75%, white powder, m.p. = 220-223 °C; **^{1}H NMR** (400 MHz, $CDCl_3$) δ 8.11 (d, J = 2.1 Hz, 1H), 7.82 (d, J = 1.7 Hz, 1H), 7.81 – 7.76 (m, 3H), 7.51 – 7.40 (m, 6H), 7.38 – 7.31 (m, 2H), 7.30 (dd, J = 8.8, 2.2 Hz, 1H), 6.57 (d, J = 1.7 Hz, 1H), 5.94 – 5.89 (m, 1H), 5.66 (s, 1H), 2.44 (d, J = 1.1 Hz, 3H). **^{13}C{^{1}H} NMR** (101 MHz, $CDCl_3$) δ 164.8, 164.3, 143.0, 141.9, 141.2, 138.2, 134.2, 133.7, 131.2, 129.7, 129.1, 128.0, 127.8, 127.7, 127.0, 126.7, 125.7, 119.8, 117.5, 111.8, 108.5, 61.0, 21.1. **HRMS** (ESI): m/z $[M+H]^+$ for $C_{25}H_{21}N_4O_2^+$, calcd 409.1659, found 409.1666.

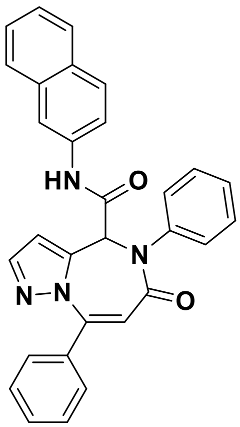	**N-(naphthalen-2-yl)-6-oxo-5,8-diphenyl-5,6-dihydro-4H-pyrazolo[1,5-a][1,4]diazepine-4-carboxamide (16u).**<br>Yield**:** 68 mg, 72%, white powder, m.p. = 231-233 °C; **^{1}H NMR** (400 MHz, DMSO-*d*6) δ 9.73 (bs, 1H), 8.07 (d, J = 2.1 Hz, 1H), 7.86 (d, J = 1.7 Hz, 1H), 7.84 – 7.74 (m, 3H), 7.57 (dd, J = 8.9, 2.1 Hz, 1H), 7.54 – 7.30 (m, 12H), 6.82 (d, J = 1.7 Hz, 1H), 6.39 (s, 1H), 6.13 (s, 1H). **^{13}C{^{1}H} NMR** (101 MHz, DMSO-*d*6) δ 165.5, 164.6, 142.8, 142.2, 141.6, 140.3, 135.5, 134.7, 133.0, 130.3, 130.1, 129.0, 128.9, 128.2, 128.0, 127.42, 127.38, 127.1, 126.7, 126.5, 125.2, 121.7, 118.3, 113.7, 109.0, 59.7. **HRMS** (ESI): m/z [M+H]$^+$ for $C_{30}H_{23}N_4O_2^+$, calcd 471.1816, found 471.1816.
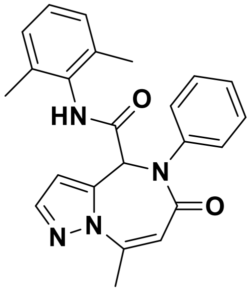	**N-(2,6-dimethylphenyl)-8-methyl-6-oxo-5-phenyl-5,6-dihydro-4H-pyrazolo[1,5-a][1,4]diazepine-4-carboxamide (16v).**<br>Yield: 61 mg, 79%, white powder, m.p. = 241-243 °C; **^{1}H NMR** (400 MHz, $CDCl_3$) δ 7.76 (d, J = 1.7 Hz, 1H), 7.46 – 7.30 (m, 5H), 7.14 – 7.03 (m, 3H), 6.87 (bs, 1H), 6.52 (d, J = 1.7 Hz, 1H), 5.95 – 5.88 (m, 1H), 5.65 (s, 1H), 2.52 – 2.46 (m, 3H), 2.13 (s, 6H). **^{13}C{^{1}H} NMR** (101 MHz, $CDCl_3$) δ 164.8, 164.1, 143.3, 141.8, 140.7, 138.4, 135.0, 133.0, 129.6, 128.5, 127.89, 127.87, 126.8, 112.0, 108.4, 60.5, 21.2, 18.4. **HRMS** (ESI): m/z [M+H]$^+$ for $C_{23}H_{23}N_4O_2^+$, calcd 387.1816, found 387.1809.
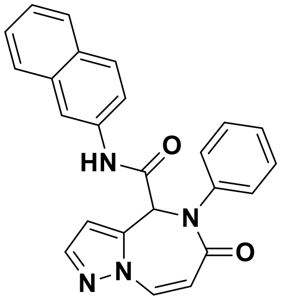	**N-(naphthalen-2-yl)-6-oxo-5-phenyl-5,6-dihydro-4H-pyrazolo[1,5-a][1,4]diazepine-4-carboxamide (16w).**<br>Yield: 79 mg, 40% (0.5 mmol scale), pale brownish powder, m.p. = 212-213 °C; **^{1}H NMR** (500 MHz, DMSO-*d*6) δ 9.72 (bs, 1H), 8.18 (d, J = 2.2 Hz, 1H), 7.89 – 7.82 (m, 4H), 7.58 (d, J = 10.2 Hz, 1H), 7.54 – 7.40 (m, 5H), 7.35 – 7.30 (m, 1H), 7.29 – 7.24 (m, 2H), 6.80 (d, J = 1.1 Hz, 1H), 6.15 (s, 1H), 5.86 (d, J = 10.2 Hz, 1H). **^{13}C{^{1}H} NMR** (126 MHz, DMSO-*d*6) δ 202.8, 202.4, 181.4, 180.2, 175.6, 173.1, 170.8, 168.3, 167.8, 166.9, 166.1, 165.13, 165.09, 164.8, 164.4, 164.2, 162.8, 158.3, 154.6, 150.3, 147.5, 97.6. **HRMS** (ESI): m/z [M+H]$^+$ for $C_{24}H_{19}N_4O_2^+$, calcd 395.1503, found 395.1506.
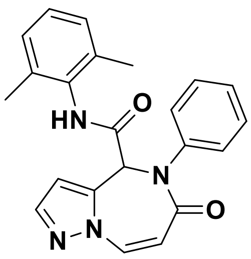	**N-(2,6-dimethylphenyl)-6-oxo-5-phenyl-5,6-dihydro-4H-pyrazolo[1,5-a][1,4]diazepine-4-carboxamide (16x).**<br>Yield: 48 mg, 64%, white powder, m.p. = 191-193 °C; **^{1}H NMR** (400 MHz, $CDCl_3$) δ 7.75 (d, J = 1.7 Hz, 1H), 7.44 – 7.29 (m, 6H), 7.12 – 7.06 (m, 1H), 7.06 – 7.00 (m, 2H), 6.79 (bs, 1H), 6.51 (d, J = 1.7 Hz, 1H), 5.91 (d, J = 10.2 Hz, 1H), 5.69 (s, 1H), 2.07 (s, 6H). **^{13}C{^{1}H} NMR** (101 MHz, $CDCl_3$) δ 164.8, 163.7, 143.7, 142.9, 138.2, 135.0, 132.8, 130.9, 129.7, 128.5, 128.04, 127.99, 127.0, 113.3, 108.6, 60.6, 18.5. **HRMS** (ESI): m/z [M+H]$^+$ for $C_{22}H_{21}N_4O_2^+$, calcd 373.1659, found 373.1655.

Telescope procedure for the synthesis of pyrazolodiazepine 16a:

Pyrazole-3-carboxaldehyde **14a** (144 mg, 1.5 mmol, 1.0 equiv) was placed in screw cap vial charged with magnetic stirrer followed by the addition of methanol (7.5 mL), 2-butynoic acid **8a**, (126 mg, 1.5 mmol, 1.0 equiv), aniline **2** (140 mg, 1.5 mmol, 1.0 equiv) and tert-Butyl isocyanide **4a** (125 mg, 1.5 mmol, 1.0 equiv). The reaction mixture was sealed and stirred at 70 °C for 24 h. The resulting mixture was transferred to the round bottom flask. The volatiles were removed and the crude product was dried under reduced pressure. The crude Ugi adduct **15a** was quantitatively transferred to the screw cap vial using 1,4-dioxane (7.5 mL), followed by addition of Ag(OTf) catalyst (77 mg, 20 mol%). The reaction mixture was then stirred for 7 hours at 90 °C. After completion of the reaction, the reaction vial was cooled down to room temperature and the resulting mixture was diluted with EtOAc. After dilution silica gel was added, and then solvent was removed under reduced pressure. Column chromatography was performed using a hexane/ethyl acetate mixture as the eluent (the ratio was adjusted based on TLC analysis), affording pyrazolodiazepine **16a** (330 mg, 65%).

Procedure for heterogeneous hydrogenation of 16a:

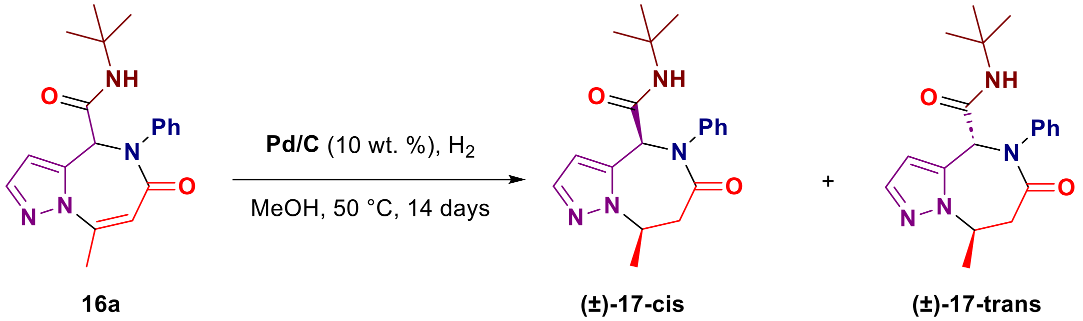

Compound **16a** (423 mg, 1.25 mmol) was placed in a round bottom flask and dissolved in methanol (12.5 mL). The solution was purged with argon followed by addition of Pd/C (10 wt.% Pd, 30 mol%, 400 mg). The resulting suspension was consecutively flashed with argon and hydrogen and left stirred under the atmosphere of hydrogen gas (1 atm) for 14 days at 50 °C. Upon completion of this time the reaction mixture was diluted with DCM and filtered through a glass filter with an extra fine porosity to remove Pd catalyst. Silica gel was then added to the filtrate, and the solvent was removed under reduced pressure. Column chromatography was performed using a hexane/ethyl acetate mixture as the eluent (30% to 100% of ethyl acetate), affording diastereomeric products **17-cis** and **17-trans**.

Procedure for the synthesis of pyrazolodiazepine 18:

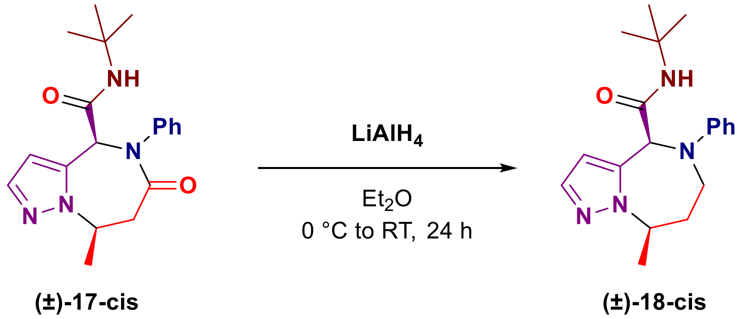

Compound **17-cis** (170 mg, 0.5 mmol) was placed in a screw cap vial and suspended in anhydrous diethyl ether (5 mL) followed by addition of 4 equiv. of lithium aluminium hydride (76 mg, 2.0 mmol) in one portion at 0 °C. The reaction vial was purged with argon, sealed, and removed from the ice bath. After stirring for 24 hours the reaction mixture was subjected to Fieser workup. Upon the Fieser workup, silica gel was added to the filtrate, and the solvent was removed on rotary evaporator. Then, column chromatography was performed using a hexane/ethyl acetate mixture as the eluent (20% to 40% of ethyl acetate), affording product **18-cis.**

Fieser workup procedure. The reaction mixture was cooled down to 0 °C. Then, water (76 µL) was carefully added followed by slow addition of 15% aqueous sodium hydroxide solution (76 µL). Then, the second portion of water (228 µL) was added and the mixture was allowed to warm up to room temperature under

stirring for approximately 15 minutes. The resulting mixture was dried over anhydrous sodium sulfate. The solids were filtered off and washed with diethyl ether. The filtrate was collected.

Subjecting compound **17-trans** (32 mg, 0. 094 mmol) afforded product **18-trans**.

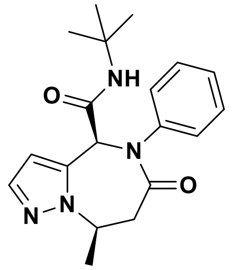	**(±)-cis-N-(tert-butyl)-8-methyl-6-oxo-5-phenyl-5,6,7,8-tetrahydro-4H-pyrazolo[1,5-a][1,4]diazepine-4-carboxamide ((±)-17-cis).** Yield: 325 mg, 76%, white powder, m.p. = 184-185 °C; **^{1}H NMR** (500 MHz, $CDCl_3$) δ 7.60 (d, J = 1.7 Hz, 1H), 7.42 – 7.35 (m, 2H), 7.33 – 7.26 (m, 3H), 6.27 (d, J = 1.8 Hz, 1H), 5.48 (bs, 1H), 5.31 (s, 1H), 4.64 (dqd, J = 12.7, 6.3, 3.5 Hz, 1H), 3.07 (dd, J = 14.5, 13.1 Hz, 1H), 2.93 (dd, J = 14.7, 3.4 Hz, 1H), 1.70 (d, J = 6.3 Hz, 3H), 1.32 (s, 9H). **^{13}C{^{1}H} NMR** (126 MHz, $CDCl_3$) δ 170.1, 166.4, 143.5, 139.3, 135.8, 129.6, 127.7, 126.7, 107.7, 62.6, 54.0, 52.4, 41.7, 28.6, 23.5. **HRMS** (ESI): m/z $[M+H]^+$ for $C_{19}H_{25}N_4O_2^+$, calcd 341.1972, found 341.1976.
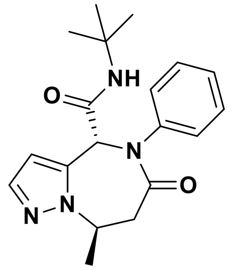	**(±)-trans-N-(tert-butyl)-8-methyl-6-oxo-5-phenyl-5,6,7,8-tetrahydro-4H-pyrazolo[1,5-a][1,4]diazepine-4-carboxamide ((±)-17-trans).** Yield: 69 mg, 16%, white powder, m.p. = 222-223 °C; **^{1}H NMR** (500 MHz, $CDCl_3$) δ 7.59 (d, J = 1.8 Hz, 1H), 7.42 – 7.37 (m, 2H), 7.37 – 7.33 (m, 2H), 7.32 – 7.27 (m, 1H), 6.23 (d, J = 1.8 Hz, 1H), 5.54 (bs, 1H), 5.32 (s, 1H), 4.83 – 4.76 (m, 1H), 3.24 (dd, J = 15.0, 3.4 Hz, 1H), 2.89 (dd, J = 15.0, 4.8 Hz, 1H), 1.59 (d, J = 6.7 Hz, 3H), 1.29 (s, 9H). **^{13}C{^{1}H} NMR** (126 MHz, $CDCl_3$) δ 169.8, 166.0, 143.8, 139.3, 135.2, 129.5, 127.6, 126.9, 106.8, 62.7, 53.7, 52.3, 40.1, 28.5, 21.5. **HRMS** (ESI): m/z $[M+H]^+$ for $C_{19}H_{25}N_4O_2^+$, calcd 341.1972, found 341.1972.
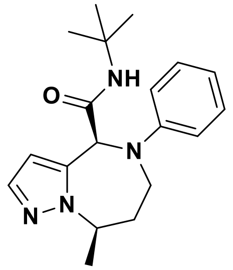	**(±)-cis-N-(tert-butyl)-8-methyl-5-phenyl-5,6,7,8-tetrahydro-4H-pyrazolo[1,5-a][1,4]diazepine-4-carboxamide ((±)-18-cis).** Yield: 96 mg, 59%, white powder, m.p. = 161-162 °C; **^{1}H NMR** (500 MHz, $CDCl_3$) δ 7.51 (d, J = 1.9 Hz, 1H), 7.35 – 7.29 (m, 2H), 6.93 – 6.87 (m, 1H), 6.85 – 6.79 (m, 2H), 6.44 (bs, 1H), 6.31 – 6.27 (m, 1H), 5.03 (s, 1H), 4.30 – 4.17 (m, 1H), 3.84 – 3.76 (m, 1H), 2.96 (ddd, J = 15.9, 11.7, 4.2 Hz, 1H), 2.38 (ddt, J = 13.9, 11.7, 5.2 Hz, 1H), 1.66 (d, J = 6.6 Hz, 3H), 1.60 – 1.50 (m, 1H), 1.37 (s, 9H). **^{1}H NMR** (500 MHz, DMSO-*d*6) δ 7.88 (bs, 1H), 7.42 (d, J = 1.8 Hz, 1H), 7.25 – 7.17 (m, 2H), 6.76 – 6.71 (m, 2H), 6.71 – 6.65 (m, 1H), 6.19 – 6.12 (m, 1H), 5.26 (s, 1H), 4.26 – 4.16 (m, 1H), 3.74 (dd, J = 15.9, 5.8 Hz, 1H), 2.97 (ddd, J = 16.3, 11.8, 4.8 Hz, 1H), 2.30 – 2.19 (m, 1H), 1.54 (d, J = 6.6 Hz, 3H), 1.43 – 1.29 (m, 10H). **^{13}C{^{1}H} NMR** (126 MHz, DMSO-*d*6) δ 168.6, 147.0, 138.1, 137.1, 129.3, 116.7, 111.8, 105.6, 60.0, 52.9, 50.6, 43.1, 33.1, 28.3, 17.5. **HRMS** (ESI): m/z $[M+H]^+$ for $C_{19}H_{27}N_4O^+$, calcd 327.2179, found 327.2179.

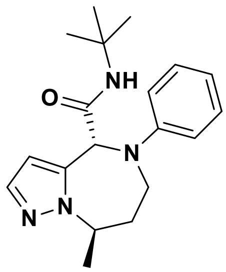	**(±)-trans-N-(tert-butyl)-8-methyl-5-phenyl-5,6,7,8-tetrahydro-4H-pyrazolo[1,5-a][1,4]diazepine-4-carboxamide ((±)-18-cis).** Yield: 12 mg, 39%, white powder; **^{1}H NMR** (500 MHz, $CDCl_3$) δ 7.44 (d, J = 1.8 Hz, 1H), 7.28 – 7.22 (m, 2H), 6.91 – 6.81 (m, 3H), 6.38 (d, J = 1.9 Hz, 1H), 6.08 (bs, 1H), 5.36 (s, 1H), 4.79 – 4.69 (m, 1H), 3.79 (ddd, J = 14.5, 8.0, 3.9 Hz, 1H), 3.64 (ddd, J = 14.4, 7.3, 3.8 Hz, 1H), 2.12 (ddt, J = 15.3, 7.7, 3.7 Hz, 1H), 1.99 (dtd, J = 14.6, 7.0, 3.9 Hz, 1H), 1.49 (d, J = 6.9 Hz, 3H), 1.32 (s, 9H). **^{13}C{^{1}H} NMR** (126 MHz, $CDCl_3$) δ 167.3, 148.4, 138.4, 137.7, 129.6, 119.7, 115.1, 108.2, 61.2, 57.0, 51.6, 46.0, 31.4, 28.6, 19.9. **HRMS** (ESI): m/z $[M+H]^+$ for $C_{19}H_{27}N_4O^+$, calcd 327.2179, found 327.2172.

X-ray diffraction analysis

The single crystals of **16m** and **(±)-17-cis** were obtained by slow evaporation of ethyl acetate solution.

Single crystals of **16m** were mounted at the top of a glass fiber with grease at RT. Reflections of **16m** were collected on Agilent Gemini Atlas with MoKα (λ = 0.71073 Å) by ω scan mode.

The single crystals of **(±)-17-cis** were immersed in a film of NVH mounted on a polyimide micro loop (MiTeGen), and measured at a temperature of 300 K. The X-ray diffraction data were collected on a Bruker D8 QUEST diffractometer with a Micro-Focus Mo Kα radiation source (IμS 3.0) and a high-resolution PHOTON III detector.

The frames were integrated with the CrysAlisPro software package using a narrow-frame algorithm. The CrysAlisPro program package was used for cell refinements and data reductions. The structure was solved using the intrinsic phasing method,[1,2] refined and visualized with the OLEX2-1.5 program.[3] A semiempirical absorption correction (SADABS) was applied to all data. All non-hydrogen atoms were refined anisotropically. Hydrogen atoms were included in structure factors calculations. All Hydrogen atoms were assigned to idealized geometric positions.

The crystallographic details are summarized in Table S1.

CCDC 2410526, and 2441192 contain supplementary crystallographic data for this paper.

---

1. Sheldrick, G. M. SHELXT–Integrated space-group and crystal-structure determination. *Acta Crystallographica Section A: Foundations and Advances*, **2015**, *71*, 3-8.
2. Sheldrick, G. M. Crystal structure refinement with SHELXL. *Acta Crystallographica Section C: Structural Chemistry*, **2015**, *71*, 3-8.
3. Dolomanov, O. V., Bourhis, L. J., Gildea, R. J., Howard, J. A., & Puschmann, H. OLEX2: a complete structure solution, refinement and analysis program. *Journal of Applied Crystallography*, **2009**, *42*, 339-341.

Table S1. Crystal data and structure refinement for **16m** and **(±)-17-cis**.

Identification code	**16m**	**(±)-17-cis**
CCDC number	2410526	2441192
Empirical formula	$C_{20}H_{24}N_4O_2$	$C_{19}H_{24}N_4O_2$
Formula weight	352.43	340.42
Temperature [K]	296.15	301.00
Crystal system	orthorhombic	monoclinic
Space group	$P2_12_12_1$	$P2_1/c$
a [Å]<br>b [Å]<br>c [Å]<br>α [°]<br>β [°]<br>γ [°]	6.4352(3)<br>17.0654(10)<br>18.0932(10)<br>90<br>90<br>90	14.3633(4)<br>11.1380(3)<br>12.0108(3)<br>90<br>95.5880(10)<br>90
Volume [Å^3]	1986.98(18)	1912.34(9)
Z	4	4
ρ_{calc} [g/cm^3]	1.178	1.182
μ [mm^{-1}]	0.078	0.079
F(000)	752.0	728.0
Crystal size [mm^3]	0.3 × 0.2 × 0.1	0.2 × 0.12 × 0.076
Radiation type	MoKα (λ = 0.71073)	MoKα (λ = 0.71073)
2Θ range for data collection [°]	6.564 to 48.988	5.916 to 52.754
Index ranges	-7 ≤ h ≤ 7, -19 ≤ k ≤ 19, -21 ≤ l ≤ 21	-17 ≤ h ≤ 17, -13 ≤ k ≤ 13, -14 ≤ l ≤ 15
Reflections collected	42032	31076
Independent reflections	3291 [R_{int} = 0.0606, R_{sigma} = 0.0239]	3859 [R_{int} = 0.0331, R_{sigma} = 0.0198]
Data/restraints/parameters	3291/18/240	3859/0/261
Goodness-of-fit on F^2 [(a)]	1.179	1.054
Final R indexes [I>=2σ (I)] [(b)]	R_1 = 0.0692, wR_2 = 0.1857	R_1 = 0.0483, wR_2 = 0.1275
Final R indexes, all data [(b)]	R_1 = 0.0951, wR_2 = 0.2339	R_1 = 0.0615, wR_2 = 0.1374
Largest diff. peak/hole [e/Å^{-3}]	0.30/-0.31	0.21/-0.20

[(a)] GooF = S = $[[\sum w(F_o^2-F_c^2)^2]/(m-n)]^{1/2}$, where m = number of reflexes and n = number of parameters; [(b)] $R_1 = \sum||F_o|-|F_c||/\sum|F_o|$; $wR_2 = [\sum[w(F_o^2-F_c^2)^2/\sum[(wF_o^2)^2]]^{1/2}$; $w = 1/[\sigma^2(F_o^2)+(aP)^2+bP]$, where $P = (F_o^2+2F_c^2)/3$

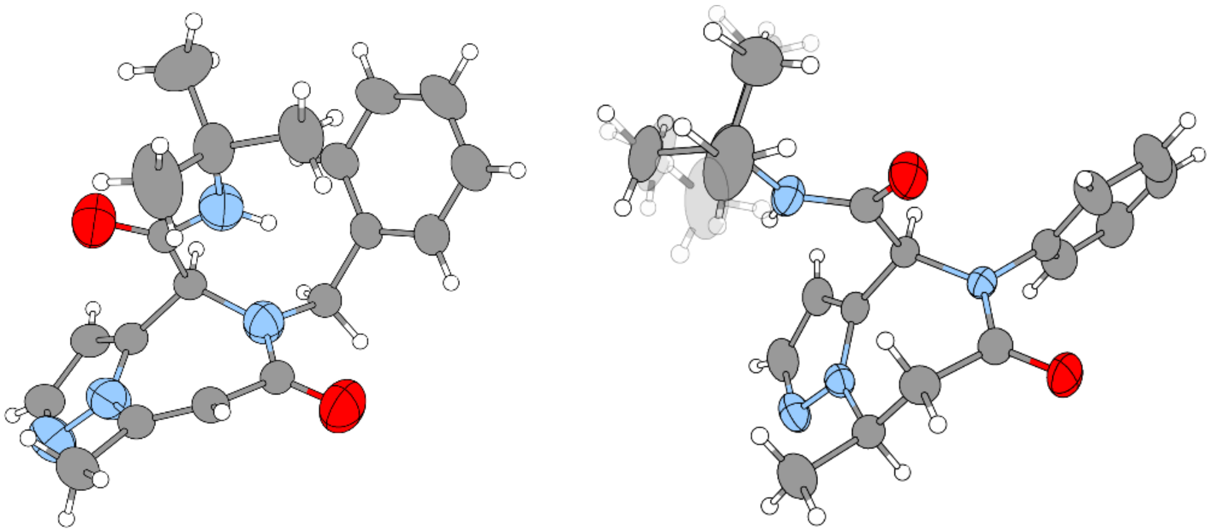

Figure S1. Molecular view of **16m** (left) and **(±)-17-cis** (right) (thermal ellipsoids shown at the 30% probability level, color code: grey – carbon, blue – nitrogen, red – oxygen, white – hydrogen).

Copies of ^{1}H and ^{13}C{^{1}H} spectra

Copies of ^{1}H and ^{13}C{^{1}H} spectra of compound **15a**

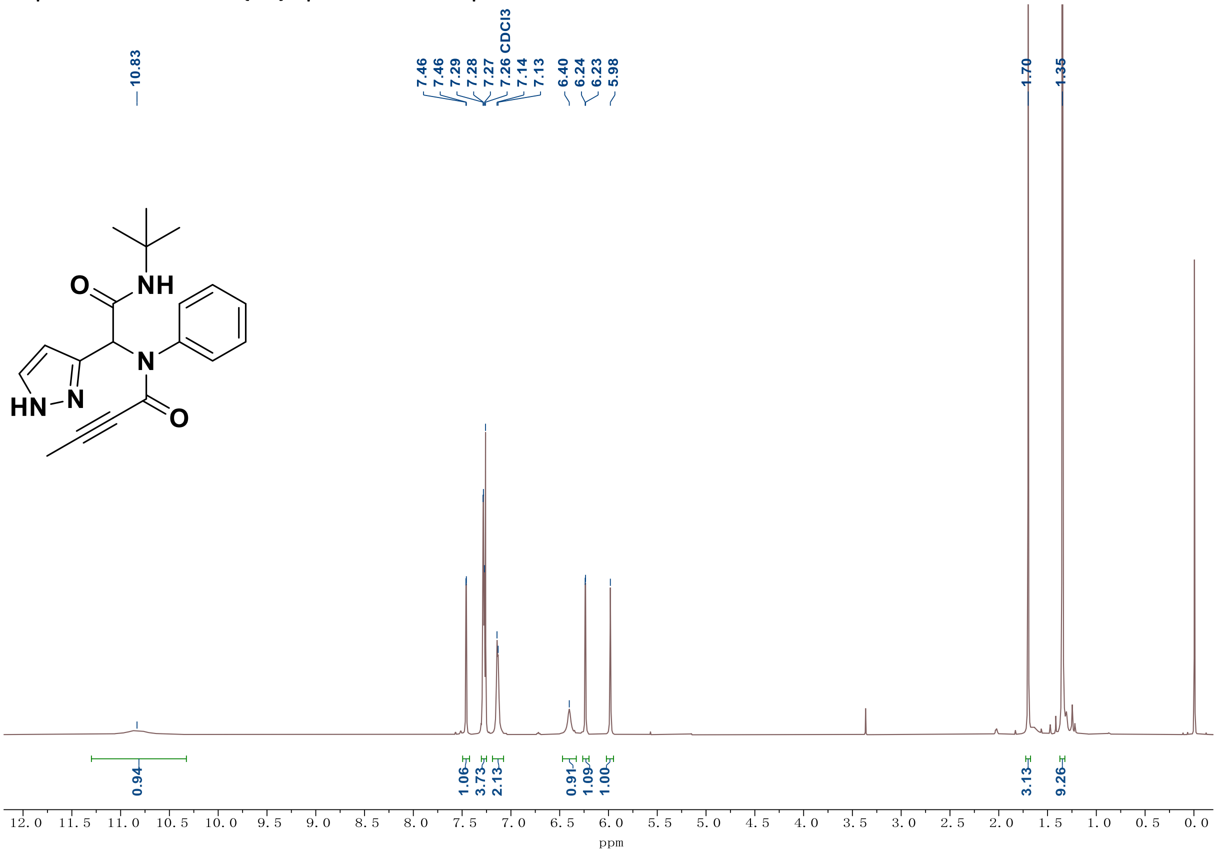

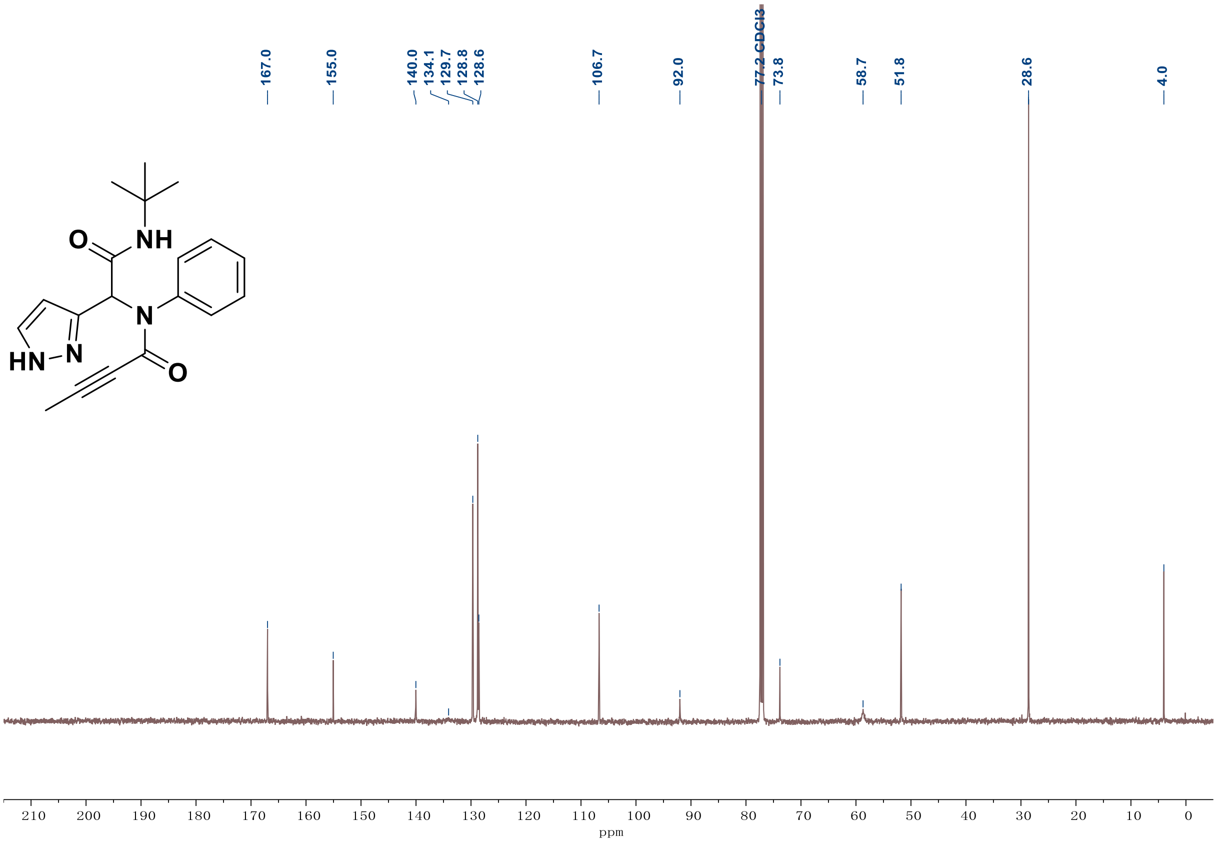

Copies of ^{1}H and ^{13}C{^{1}H} spectra of compound **15b**

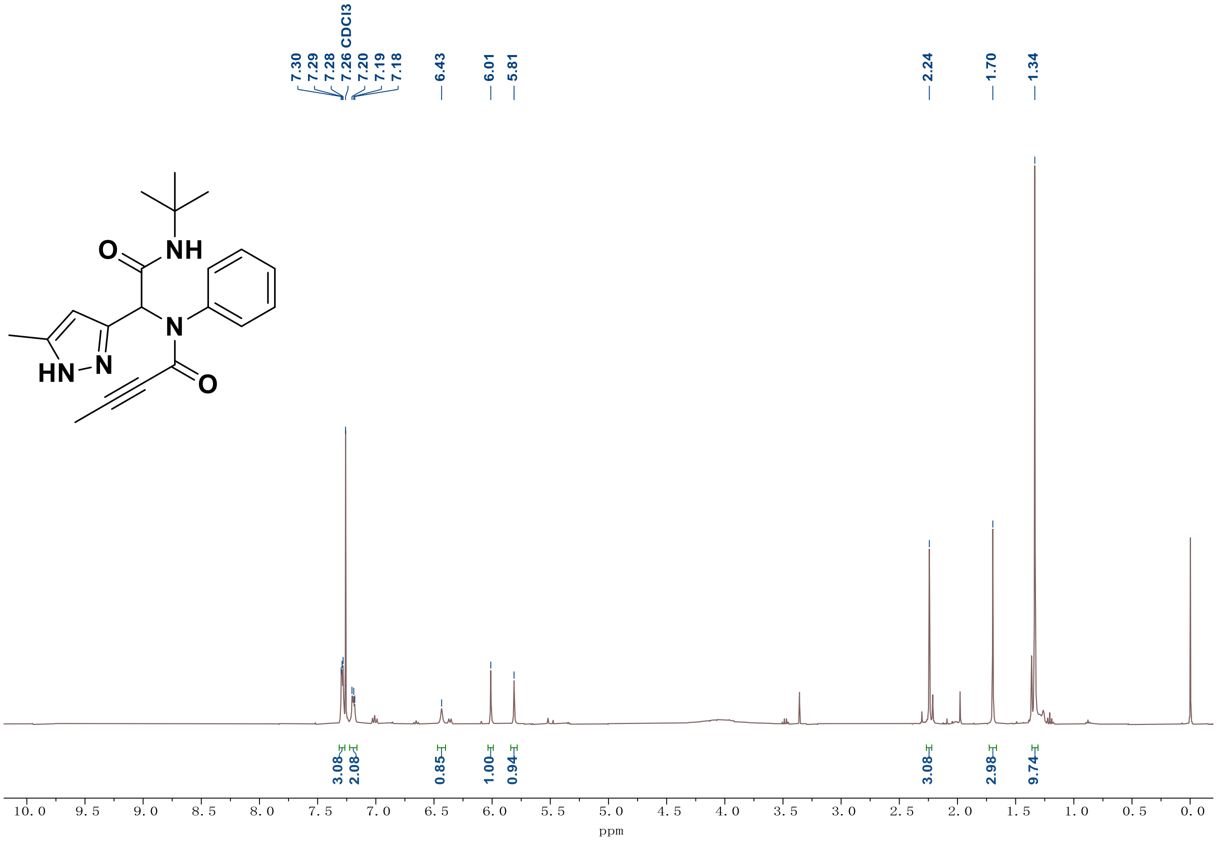

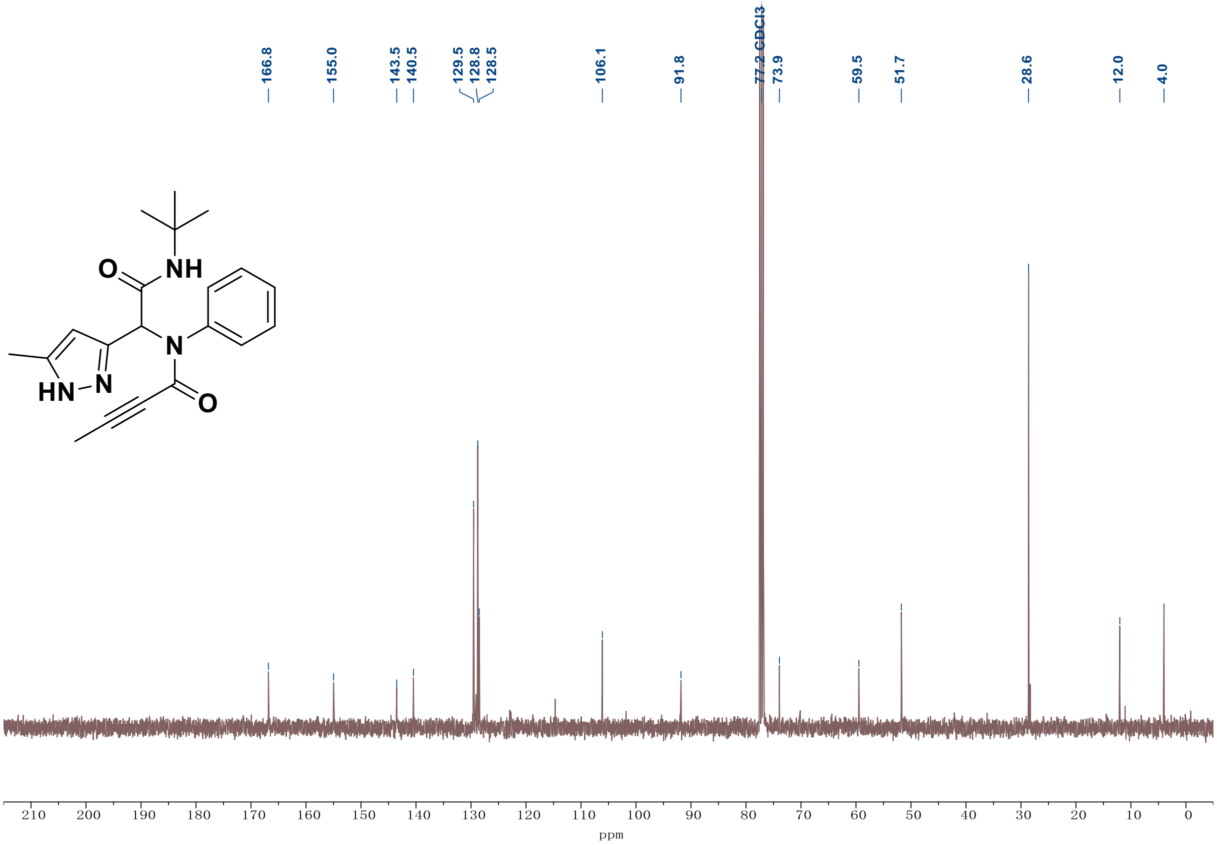

Copies of ^{1}H and $^{13}C\{^{1}H\}$ spectra of compound **15c**

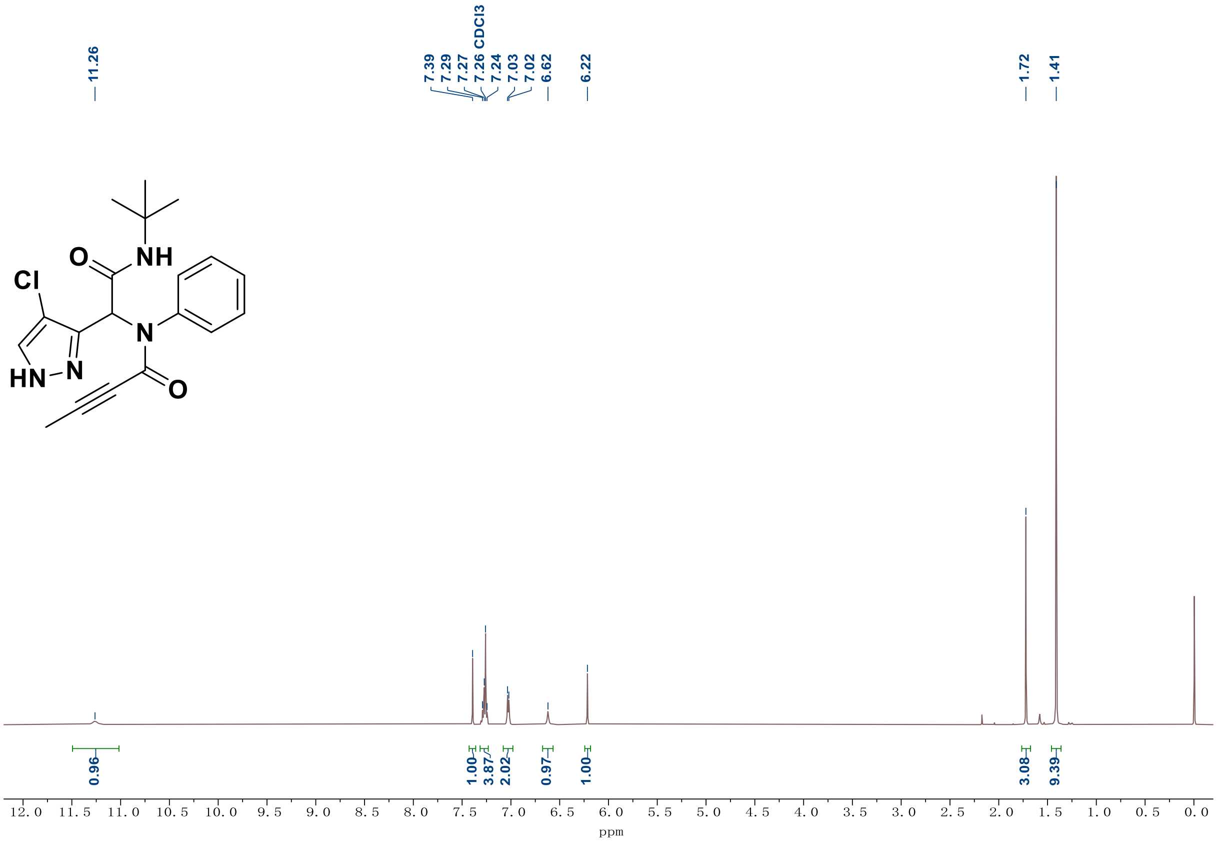

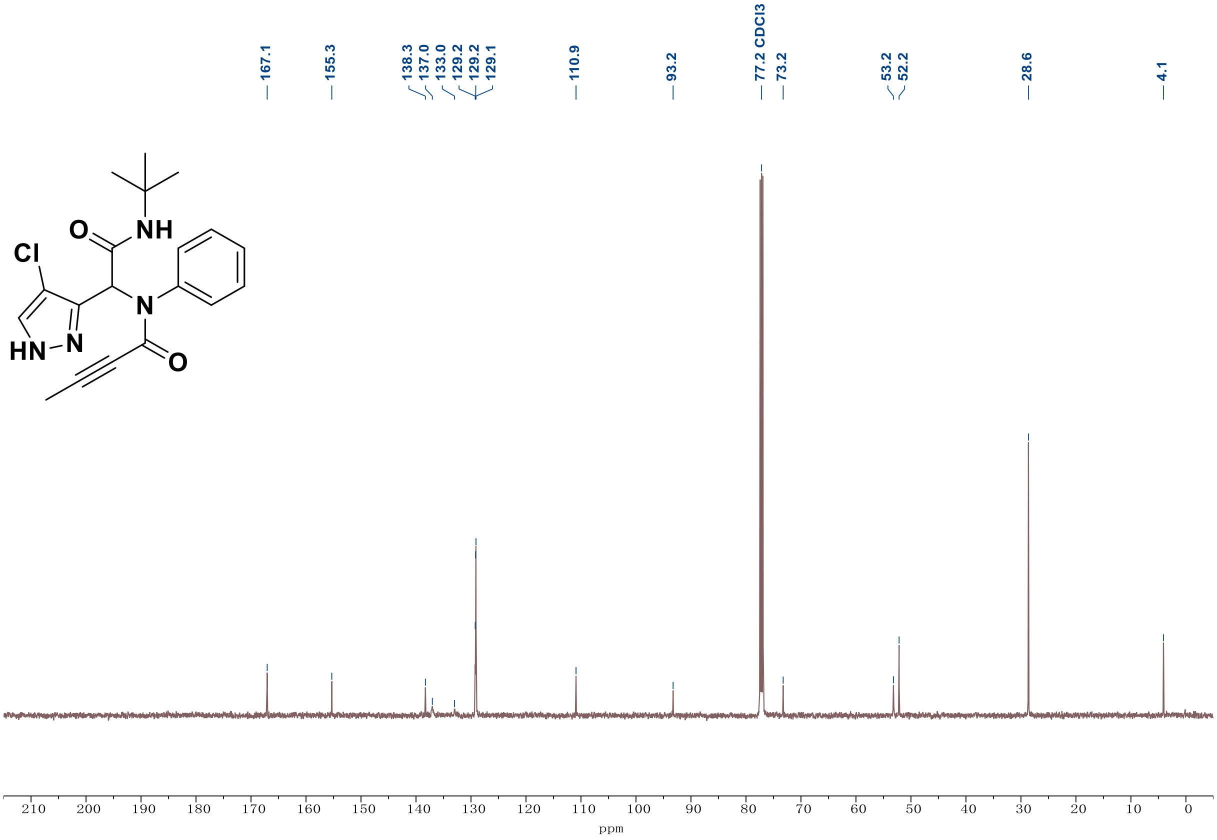

Copies of ^{1}H and ^{13}C{^{1}H} spectra of compound **15d**

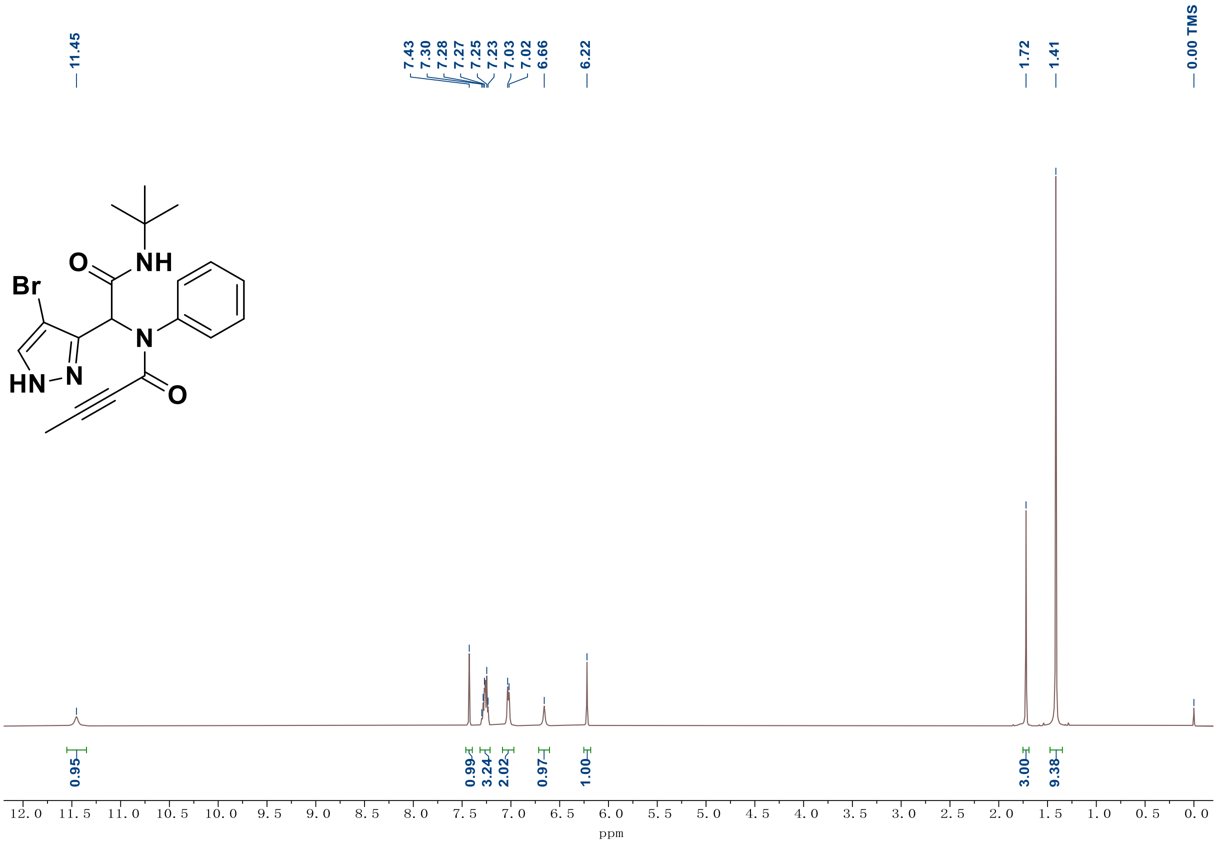

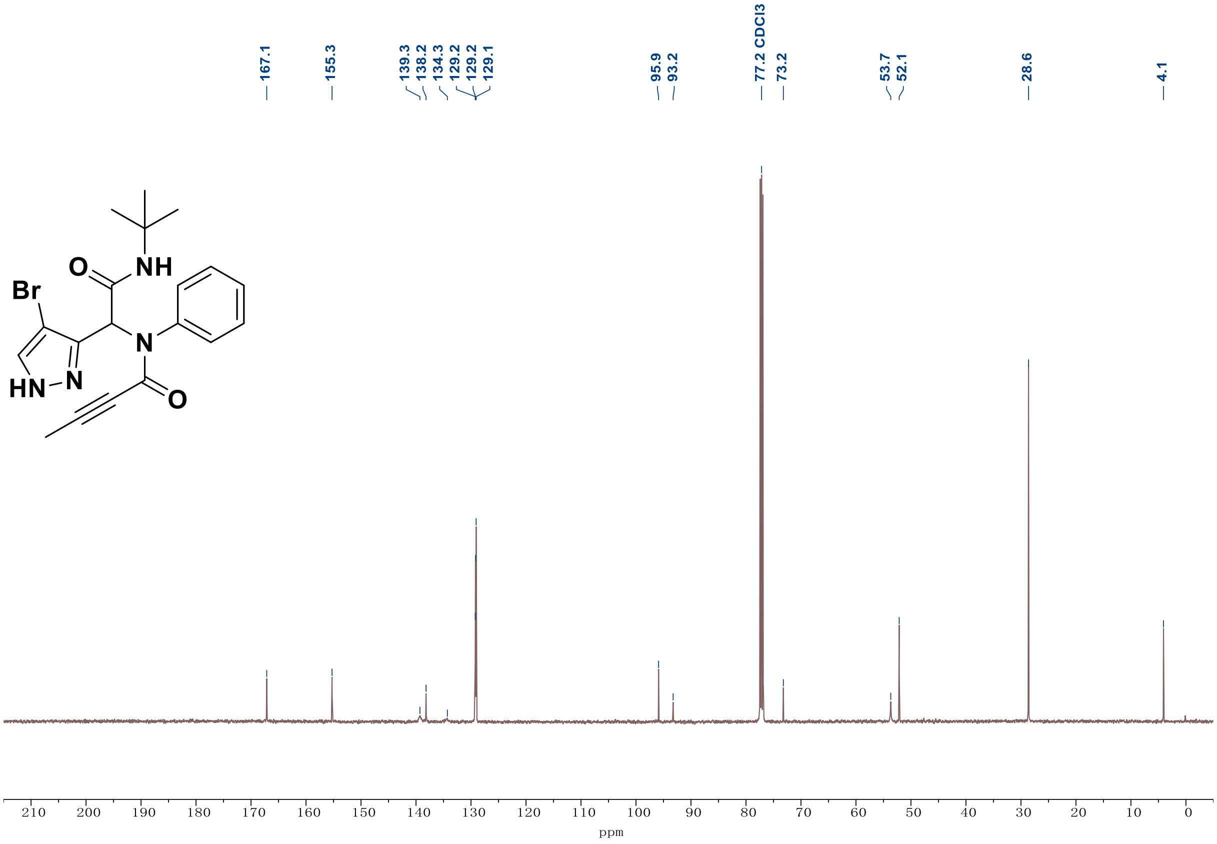

Copies of ^{1}H and $^{13}C\{^{1}H\}$ spectra of compound **15e**

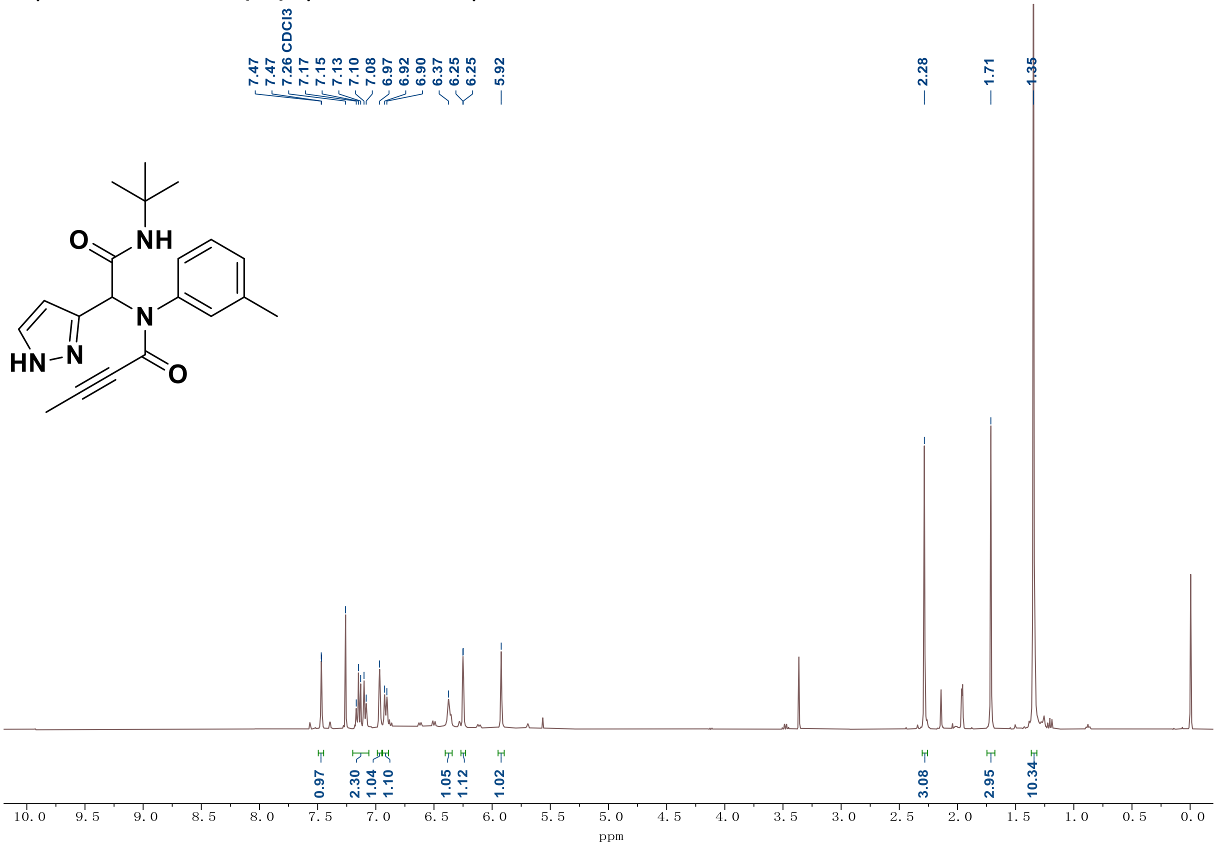

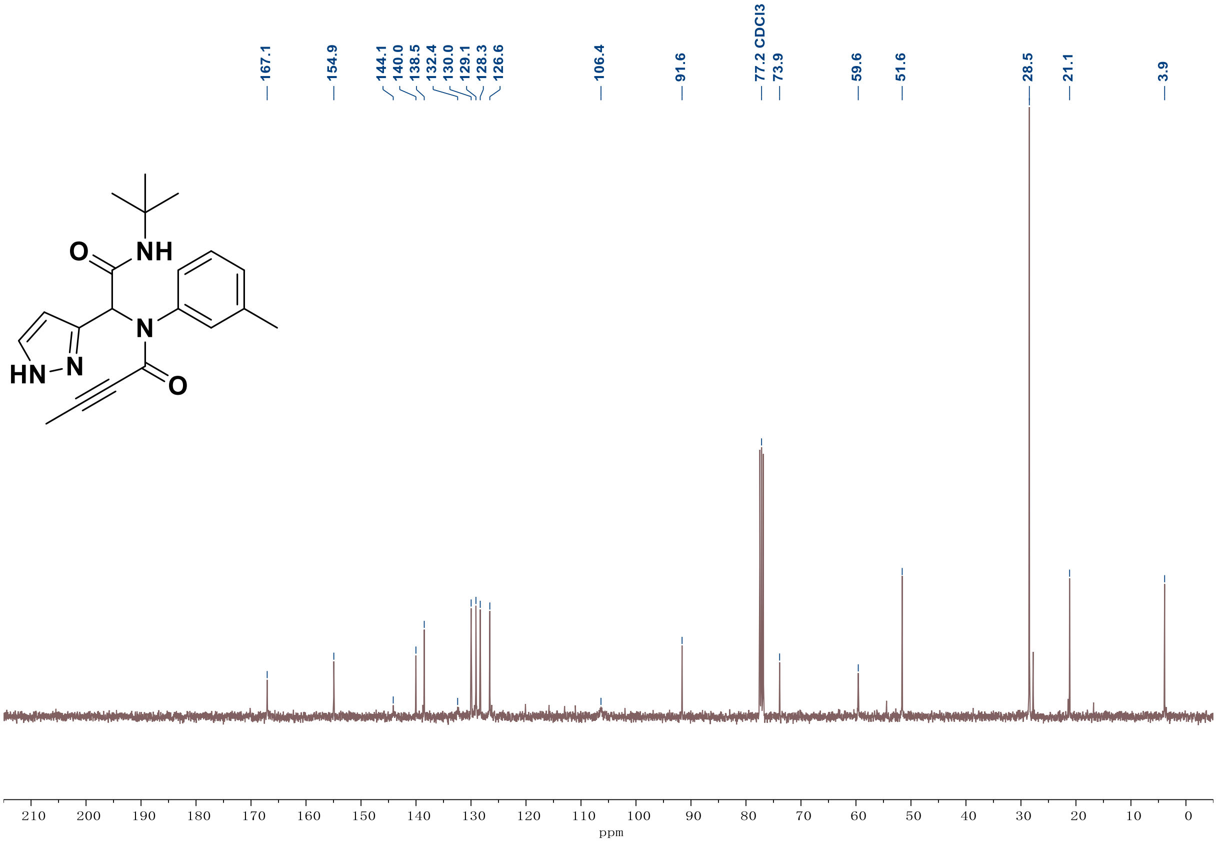

Copies of ^{1}H and $^{13}C\{^{1}H\}$ spectra of compound **15f**

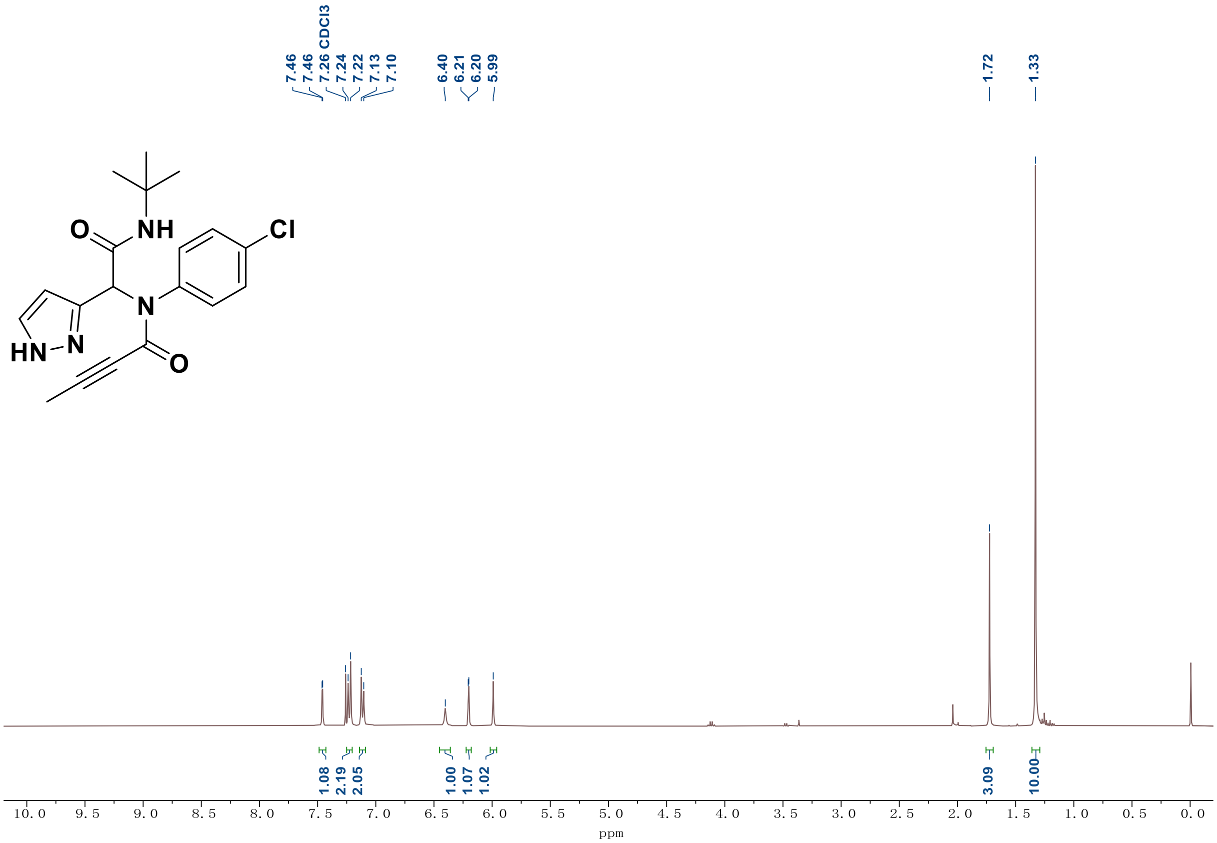

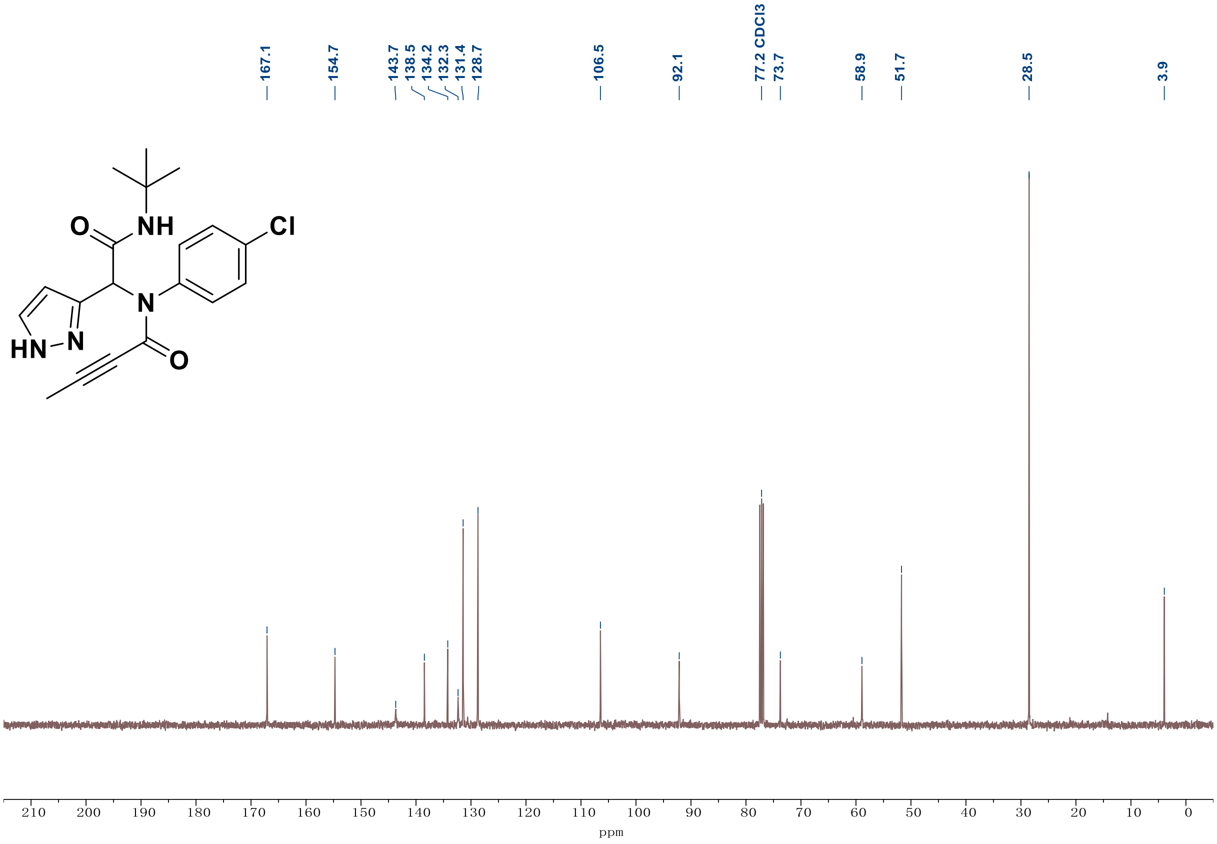

Copies of ^{1}H and $^{13}C\{^{1}H\}$ spectra of compound **15g**

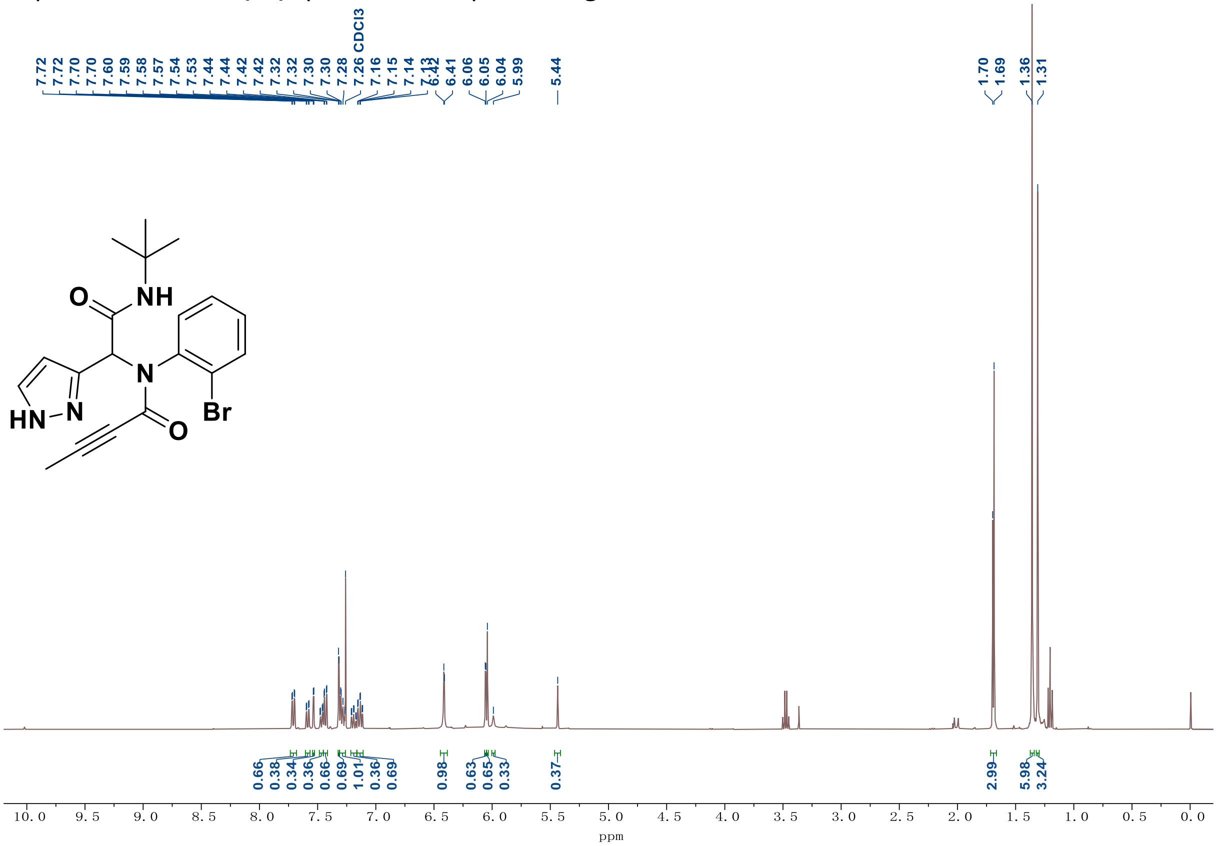

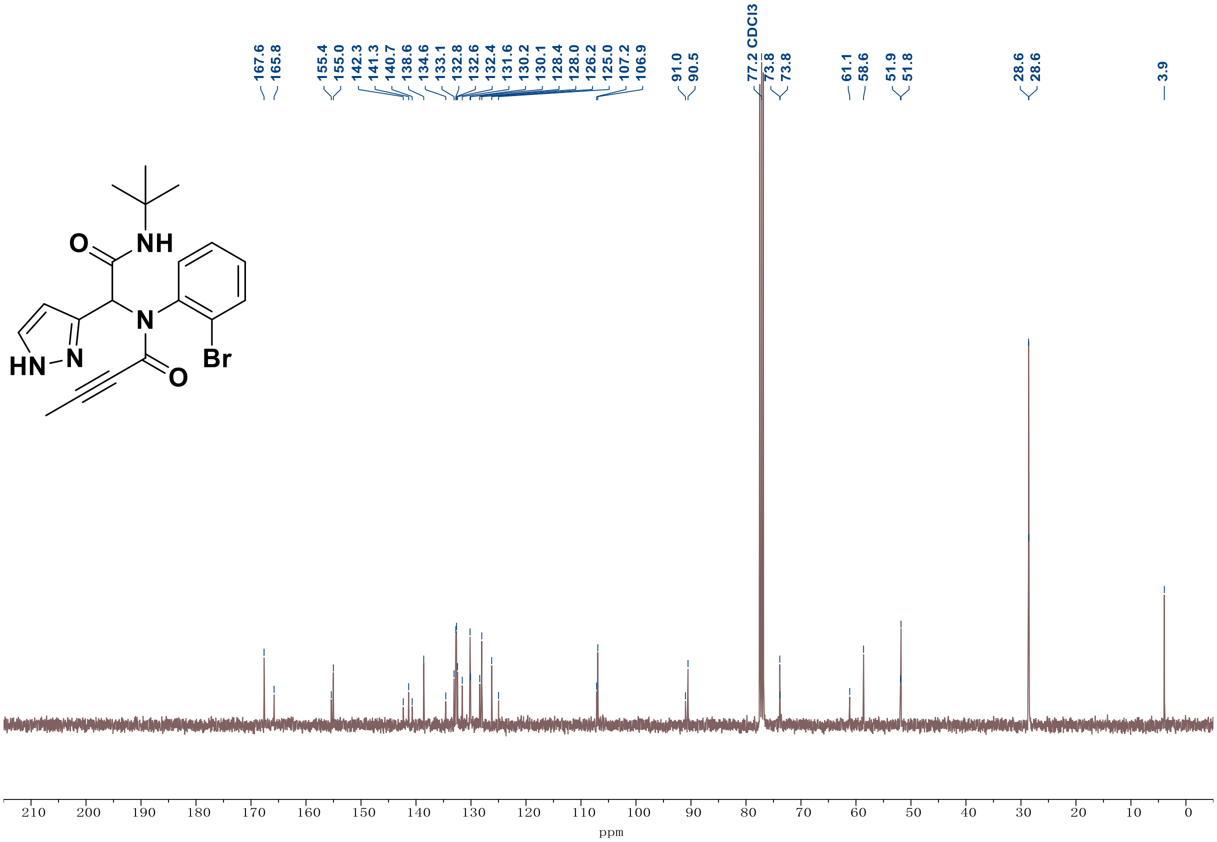

Copies of ^{1}H and $^{13}C\{^{1}H\}$ spectra of compound **15h**

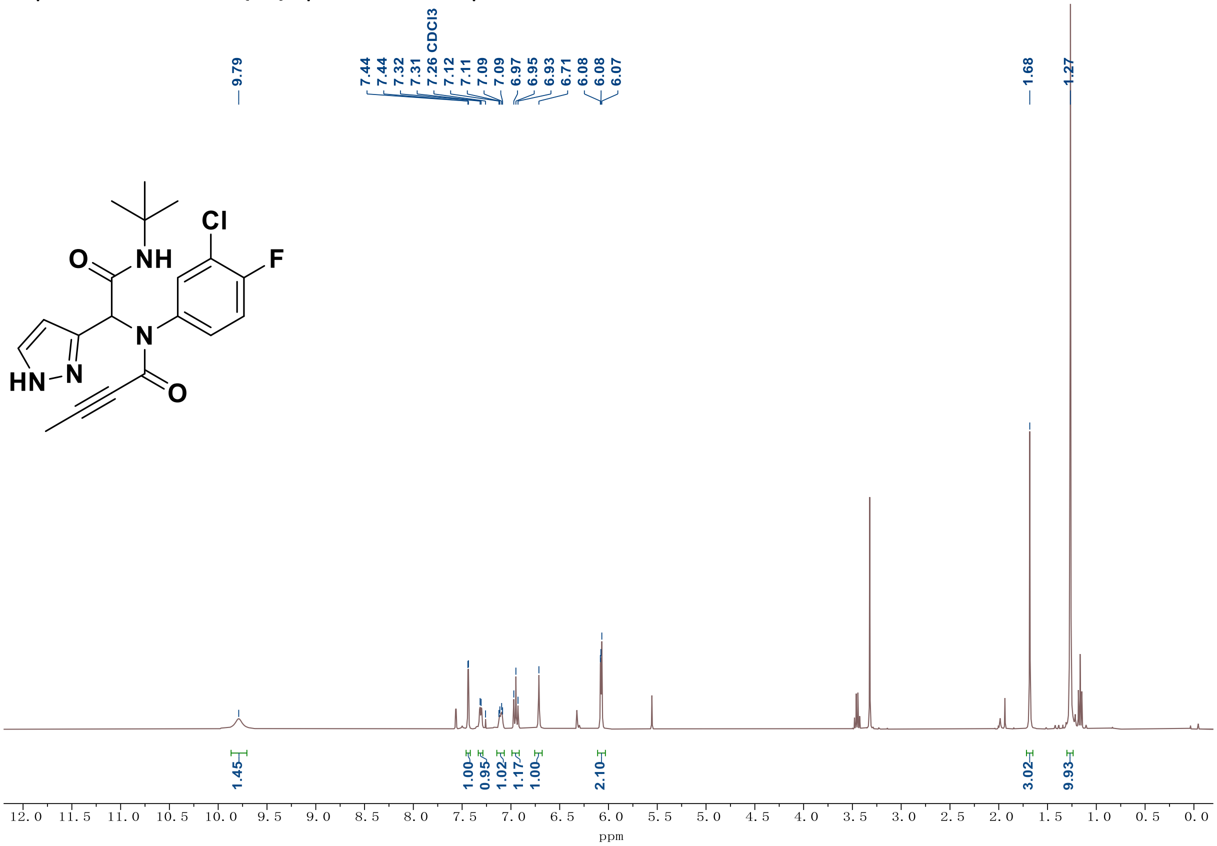

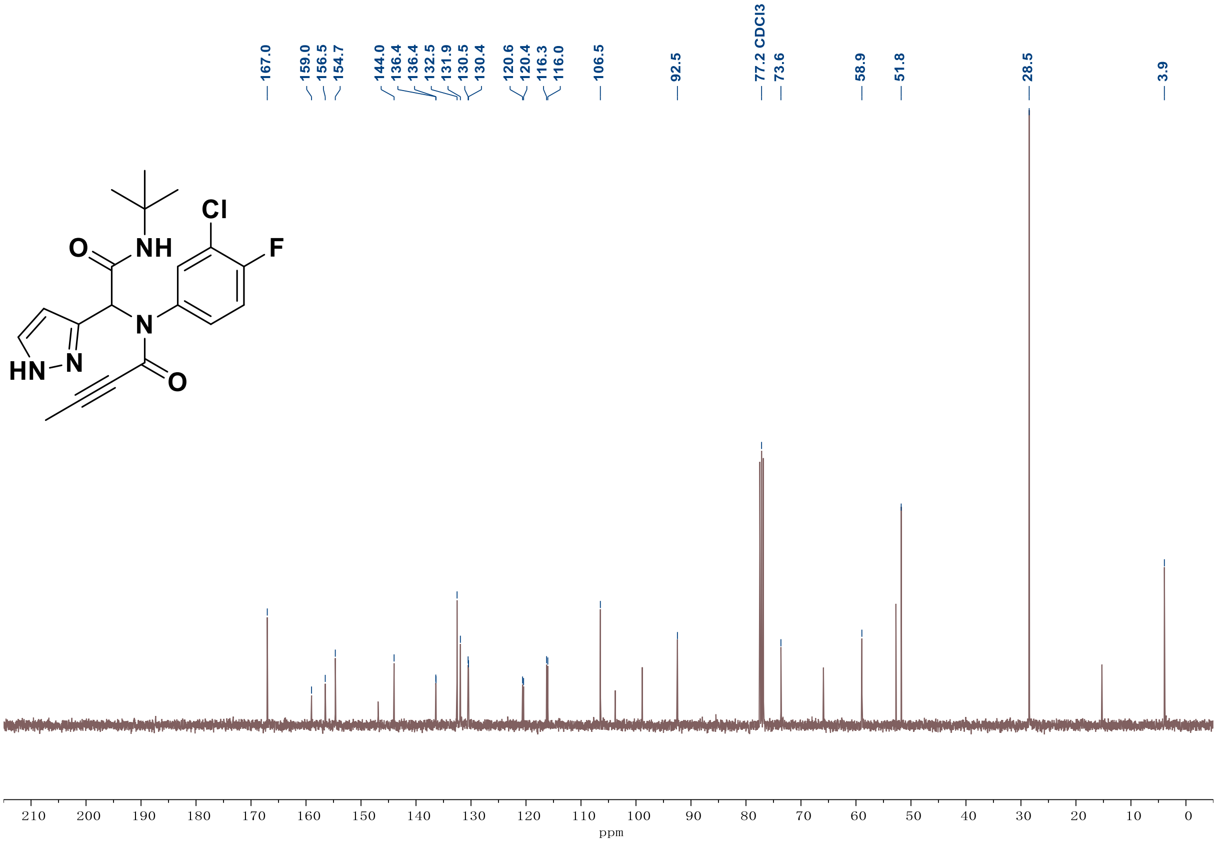

Copies of ^{1}H and $^{13}C\{^{1}H\}$ spectra of compound **15i**

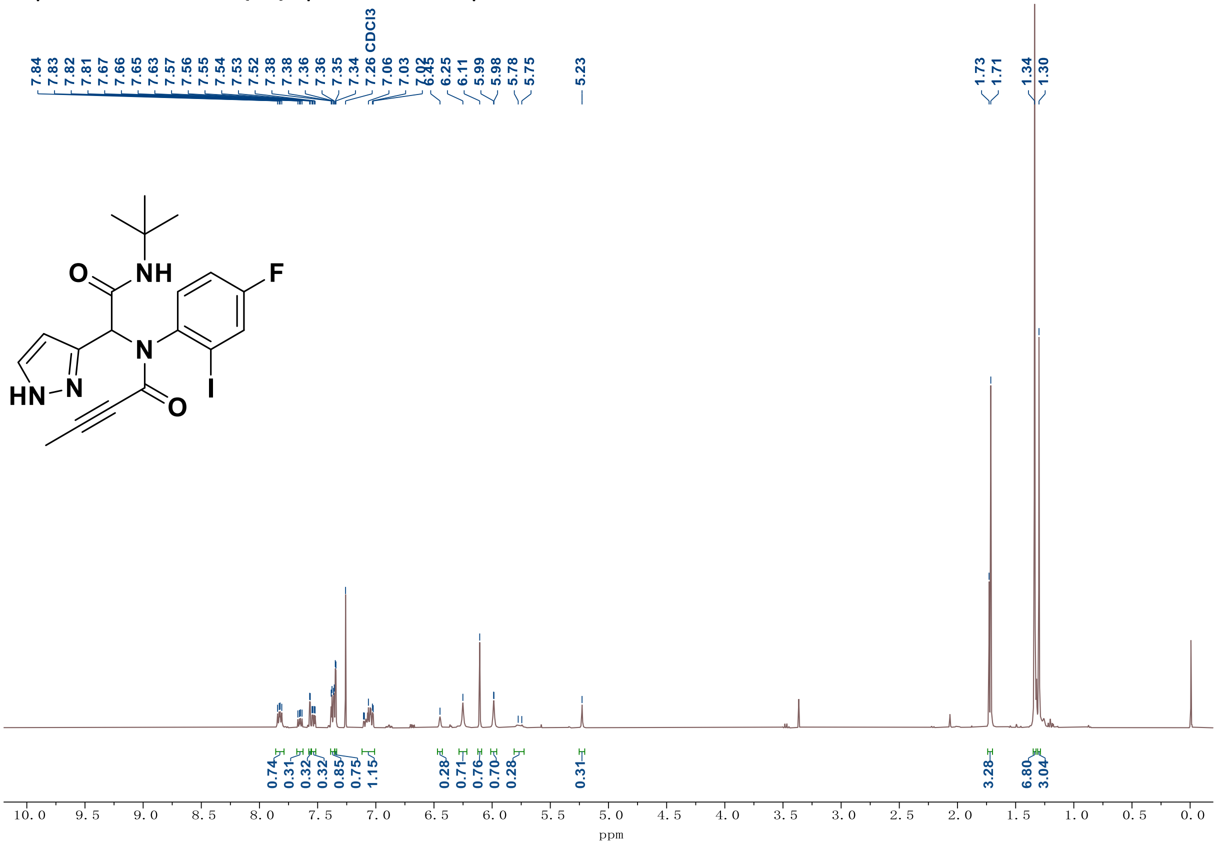

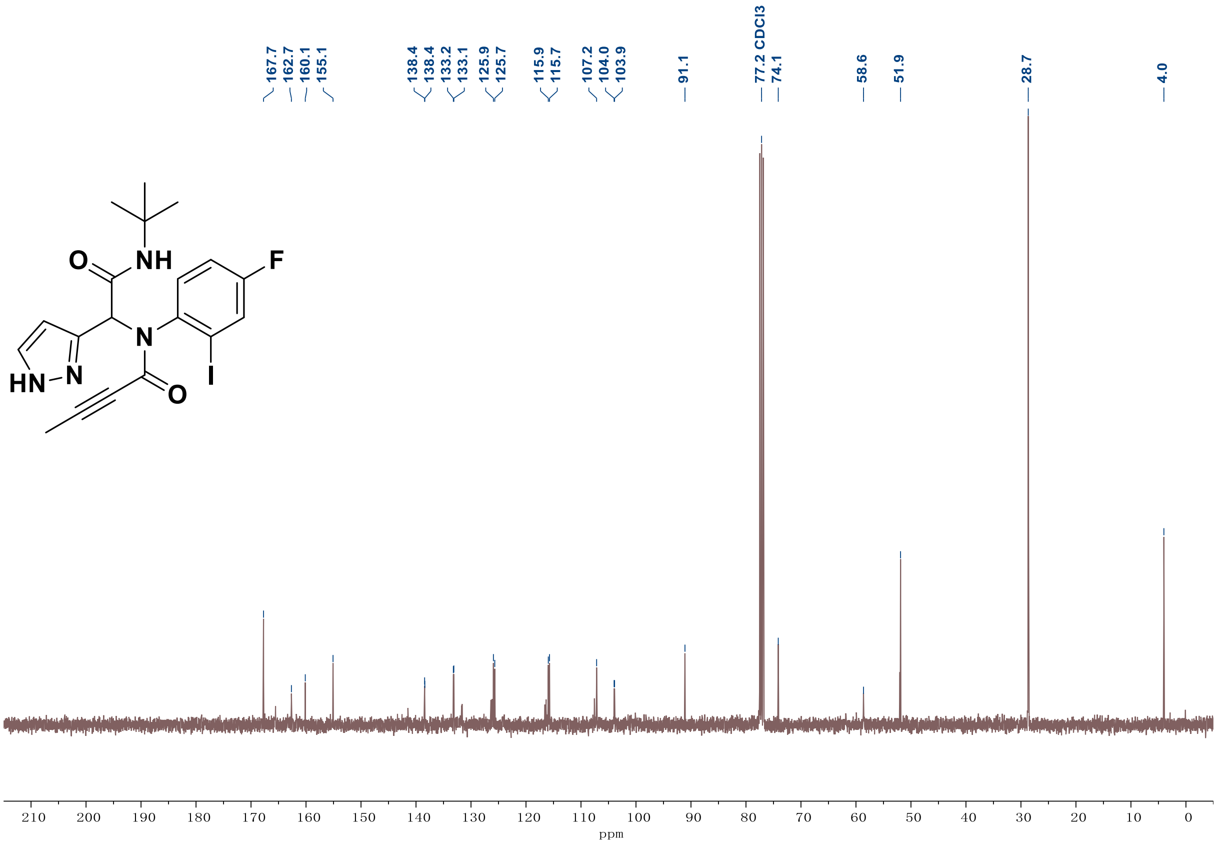

Copies of ^{1}H and $^{13}C\{^{1}H\}$ spectra of compound **15j**

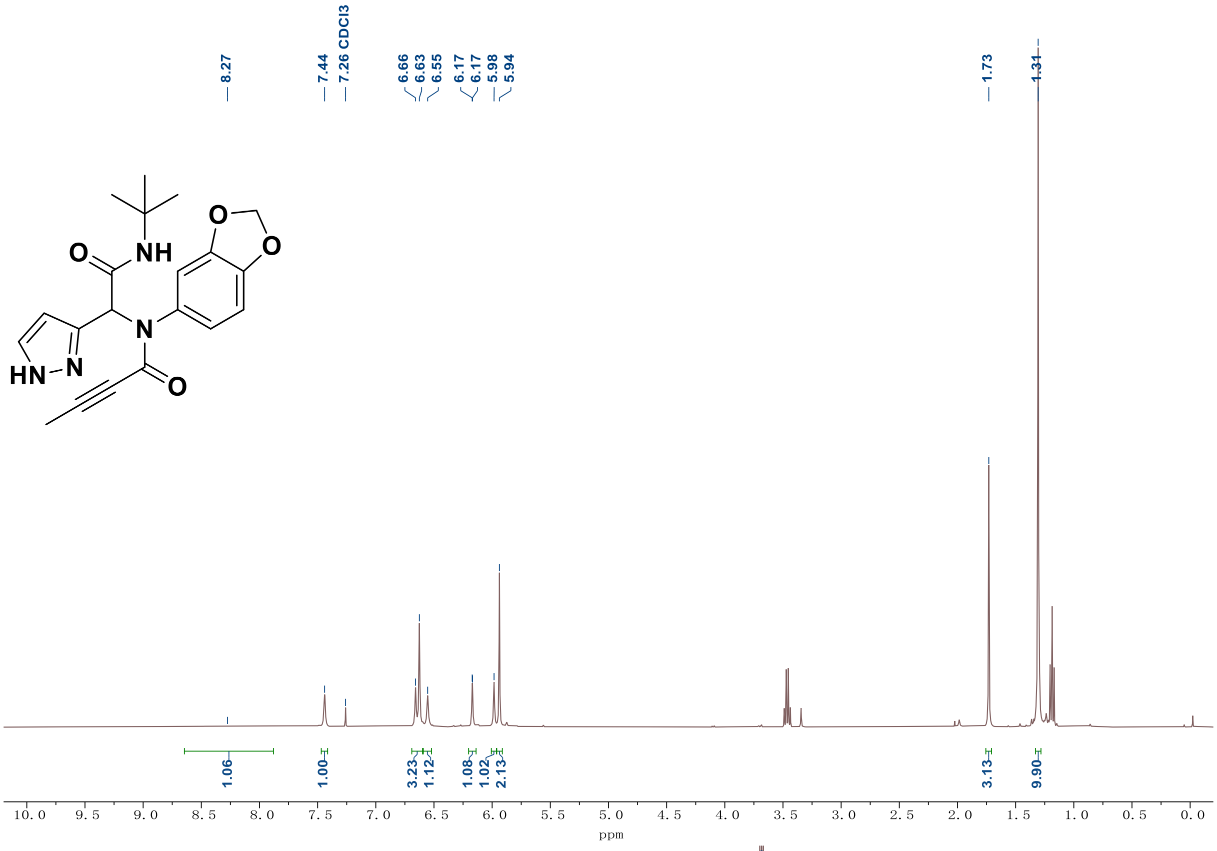

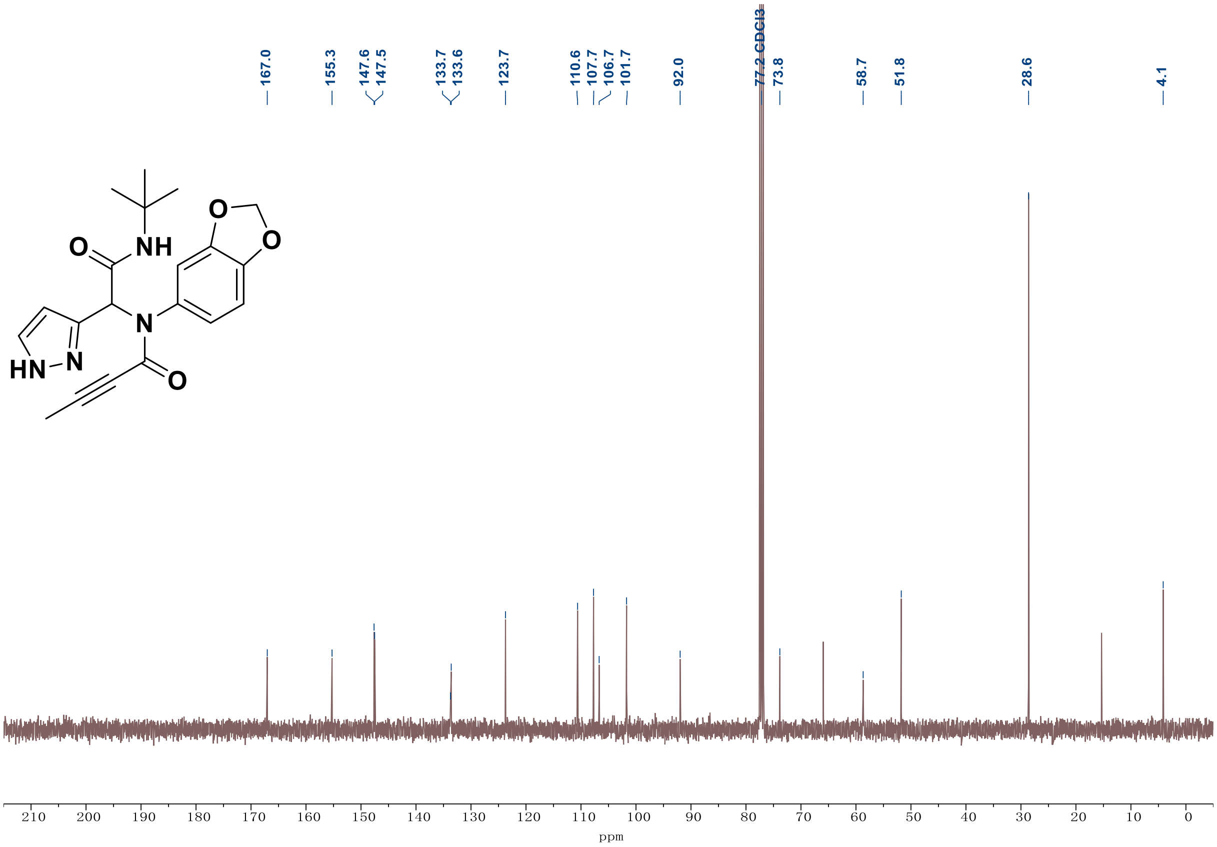

Copies of ^{1}H and $^{13}C\{^{1}H\}$ spectra of compound **15k**

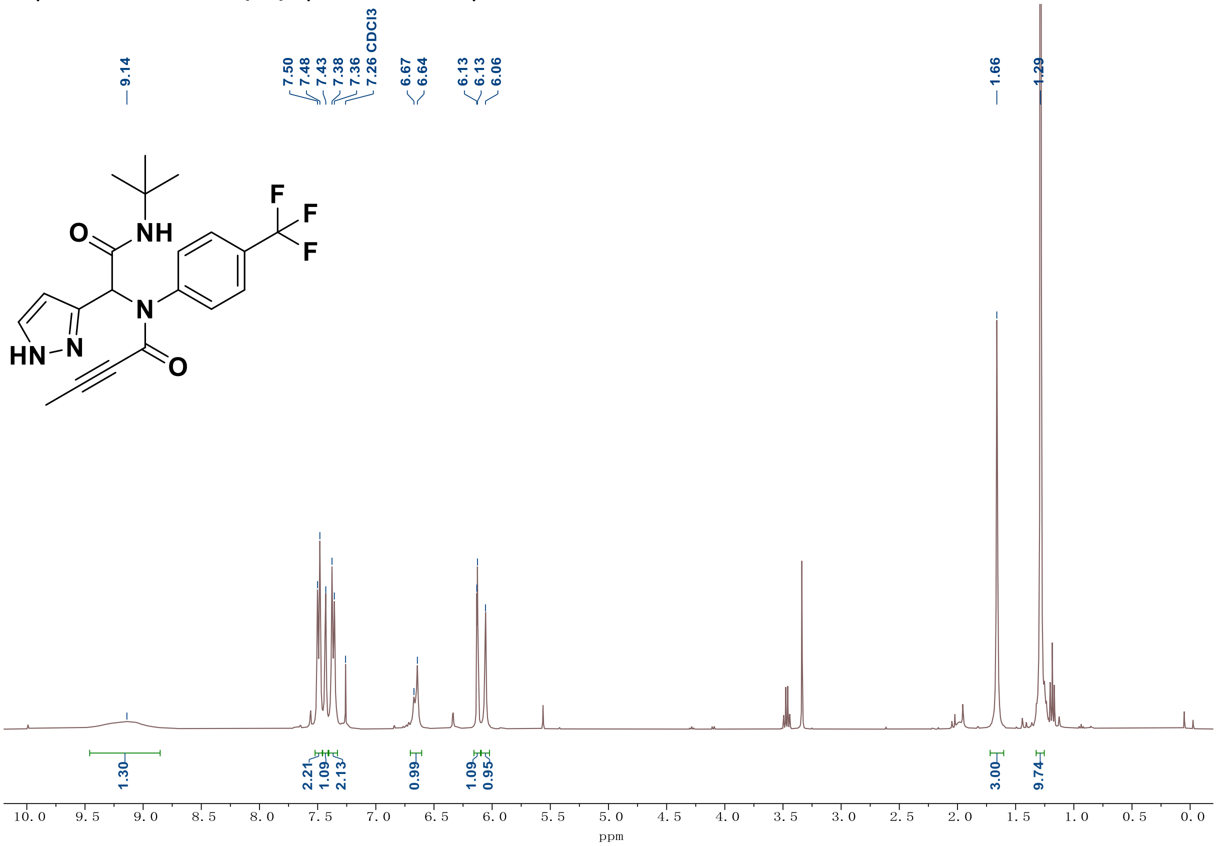

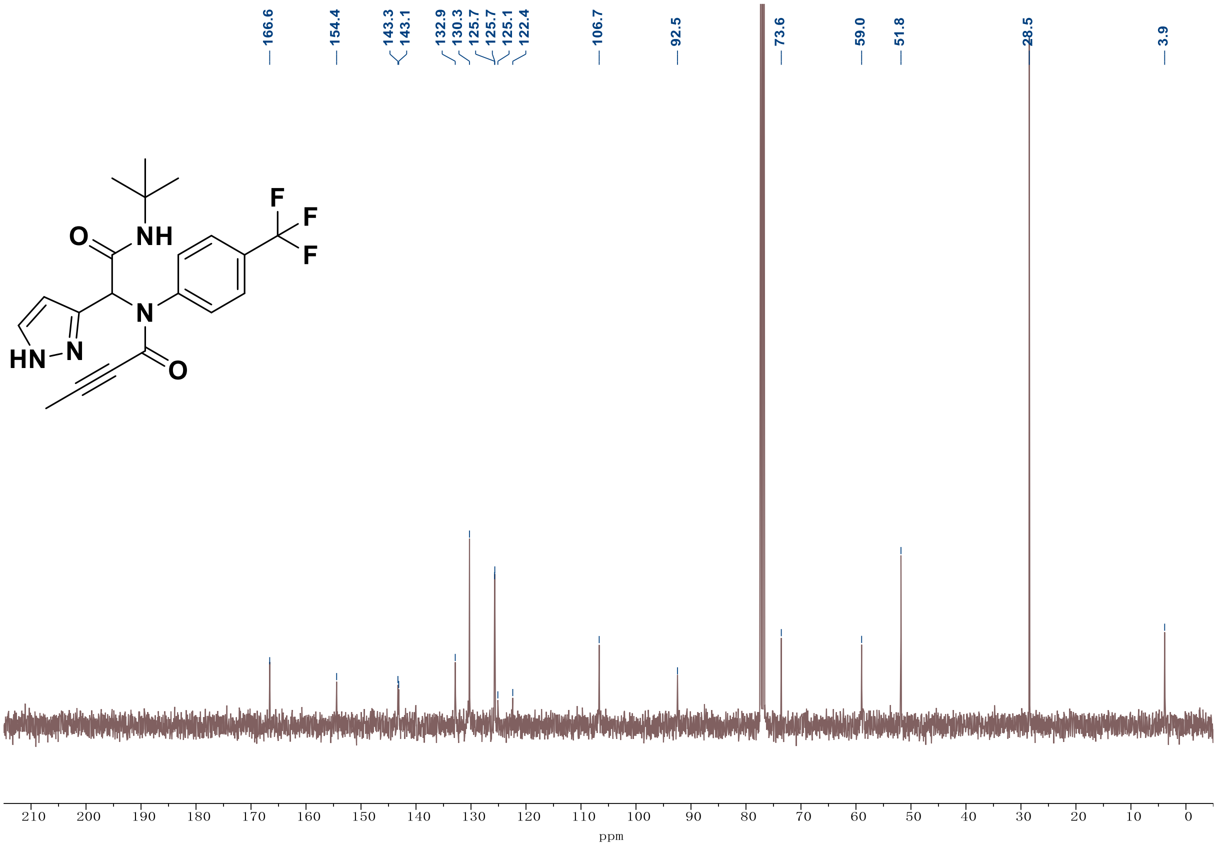

Copies of ^{1}H and $^{13}C\{^{1}H\}$ spectra of compound **15l**

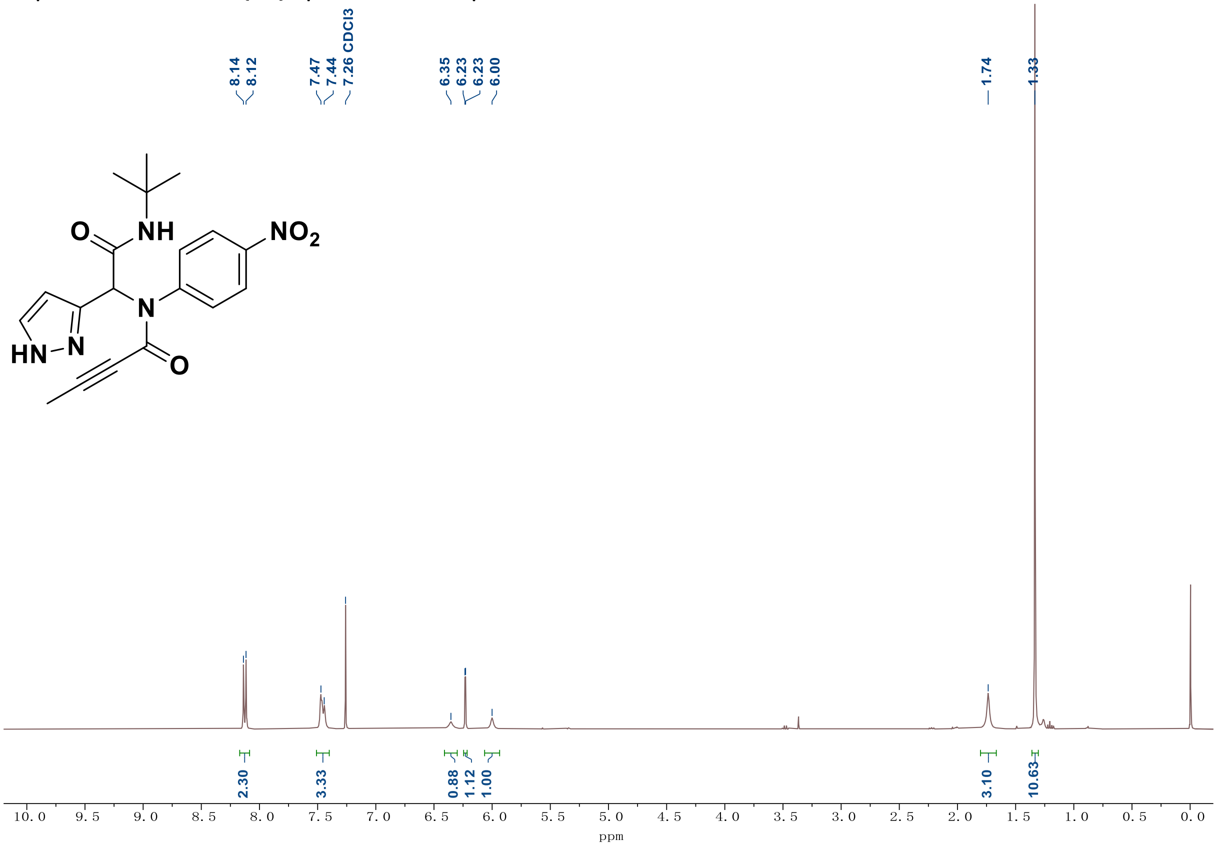

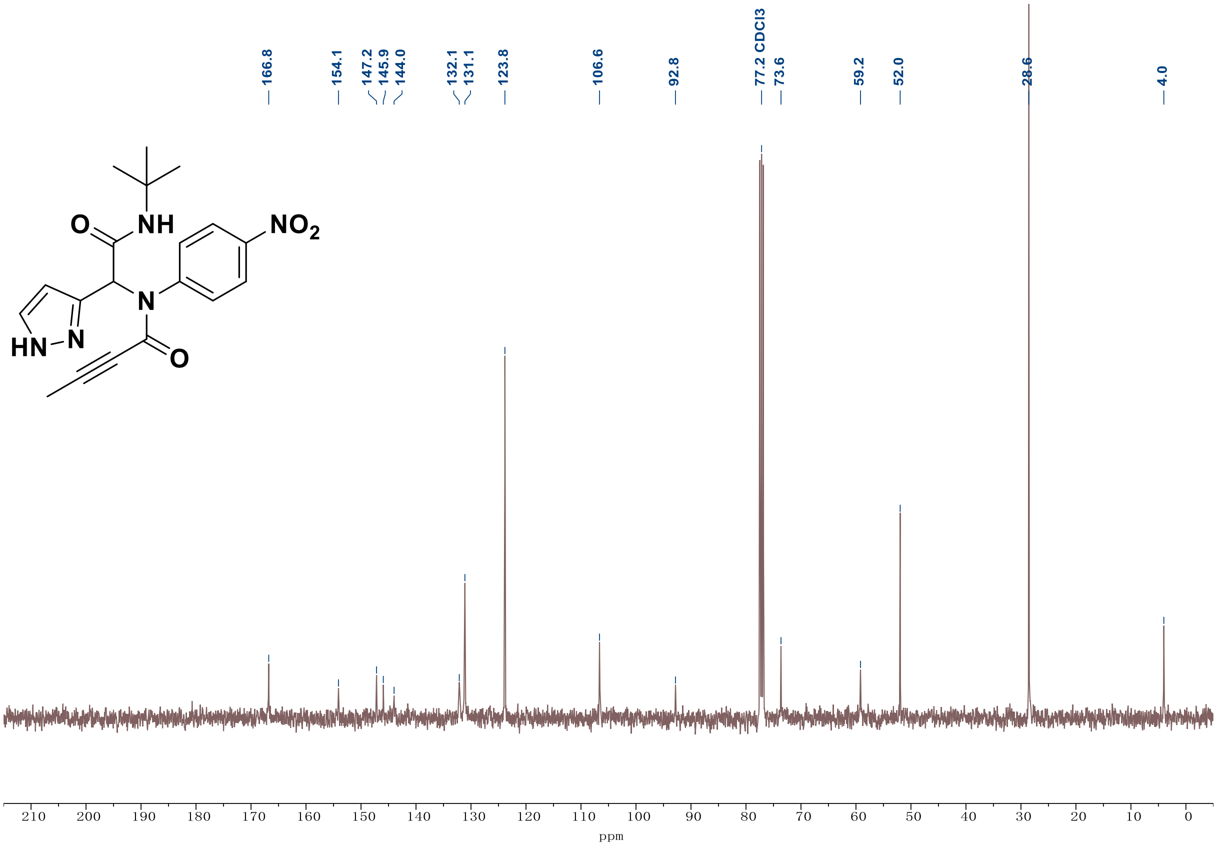

Copies of ^{1}H and $^{13}C\{^{1}H\}$ spectra of compound **15m**

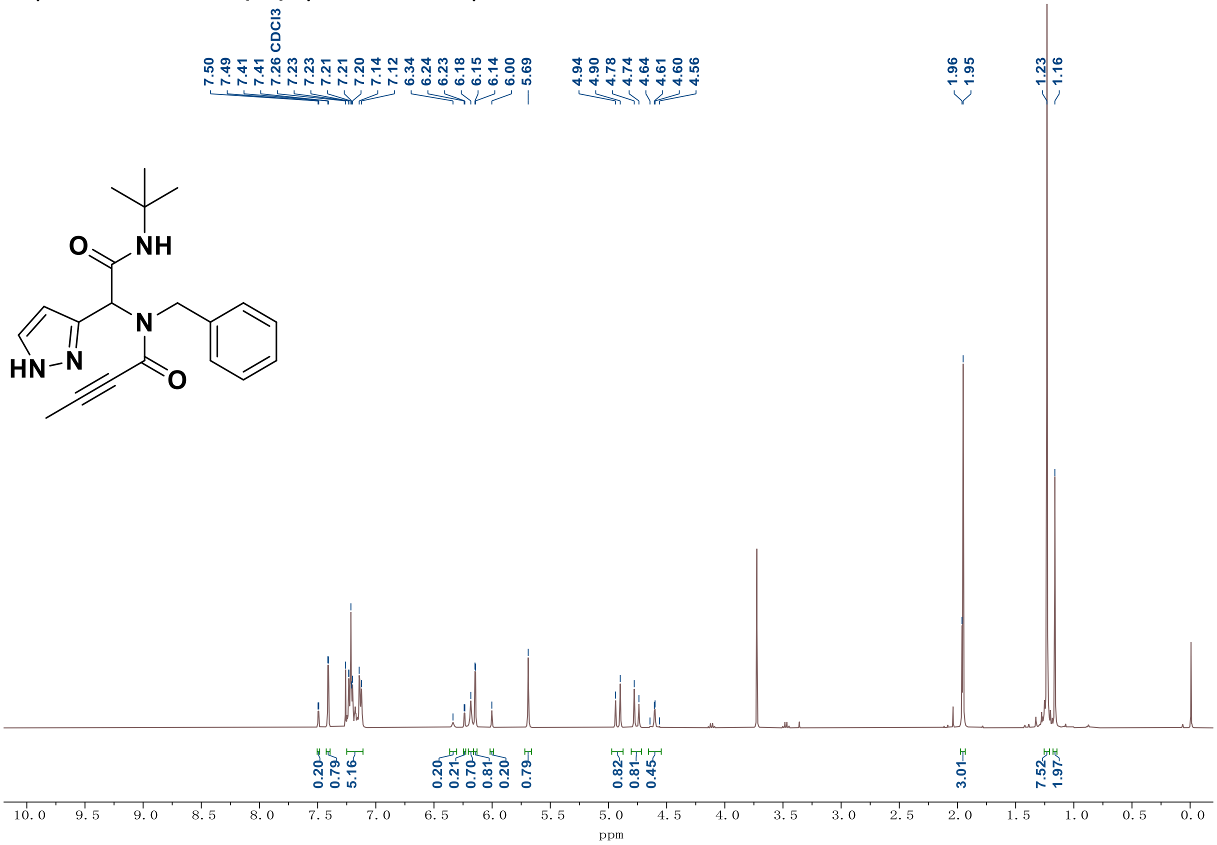

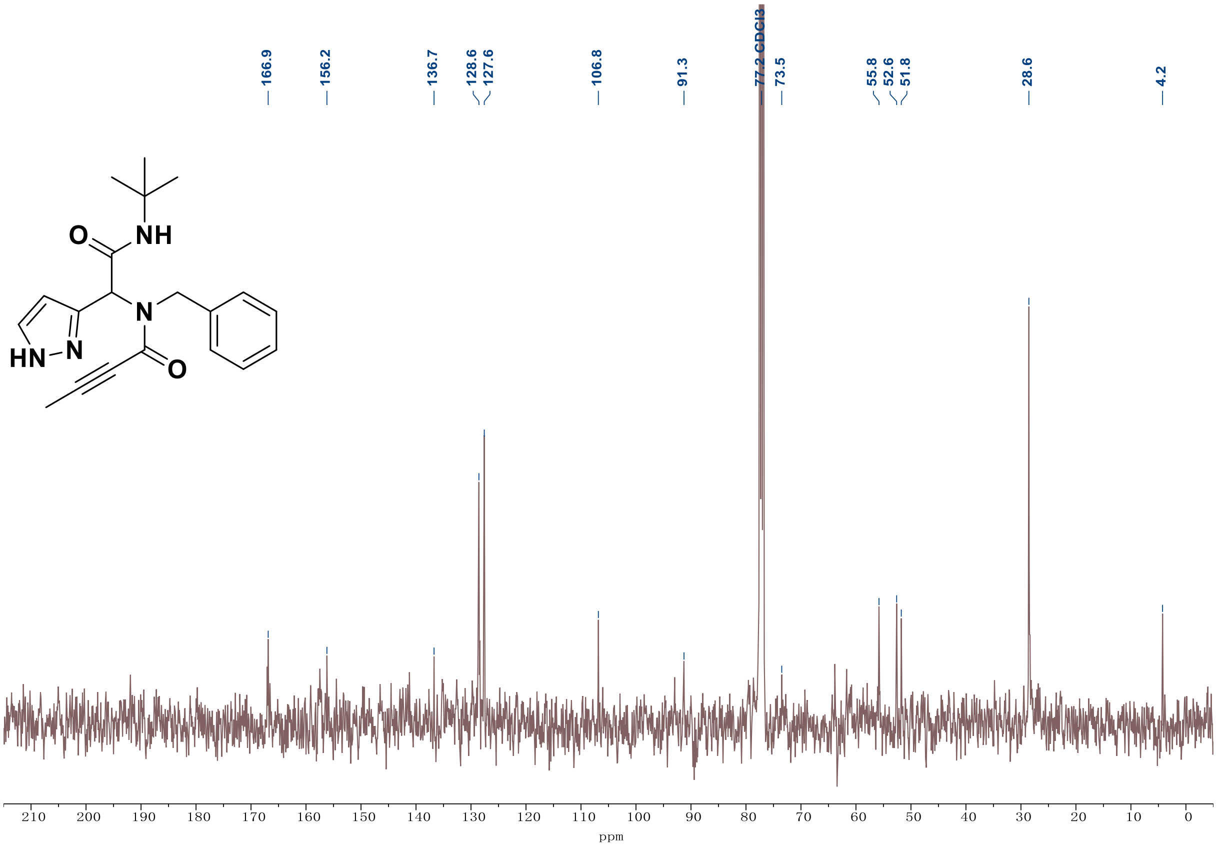

Copies of ^{1}H and $^{13}C\{^{1}H\}$ spectra of compound **15n**

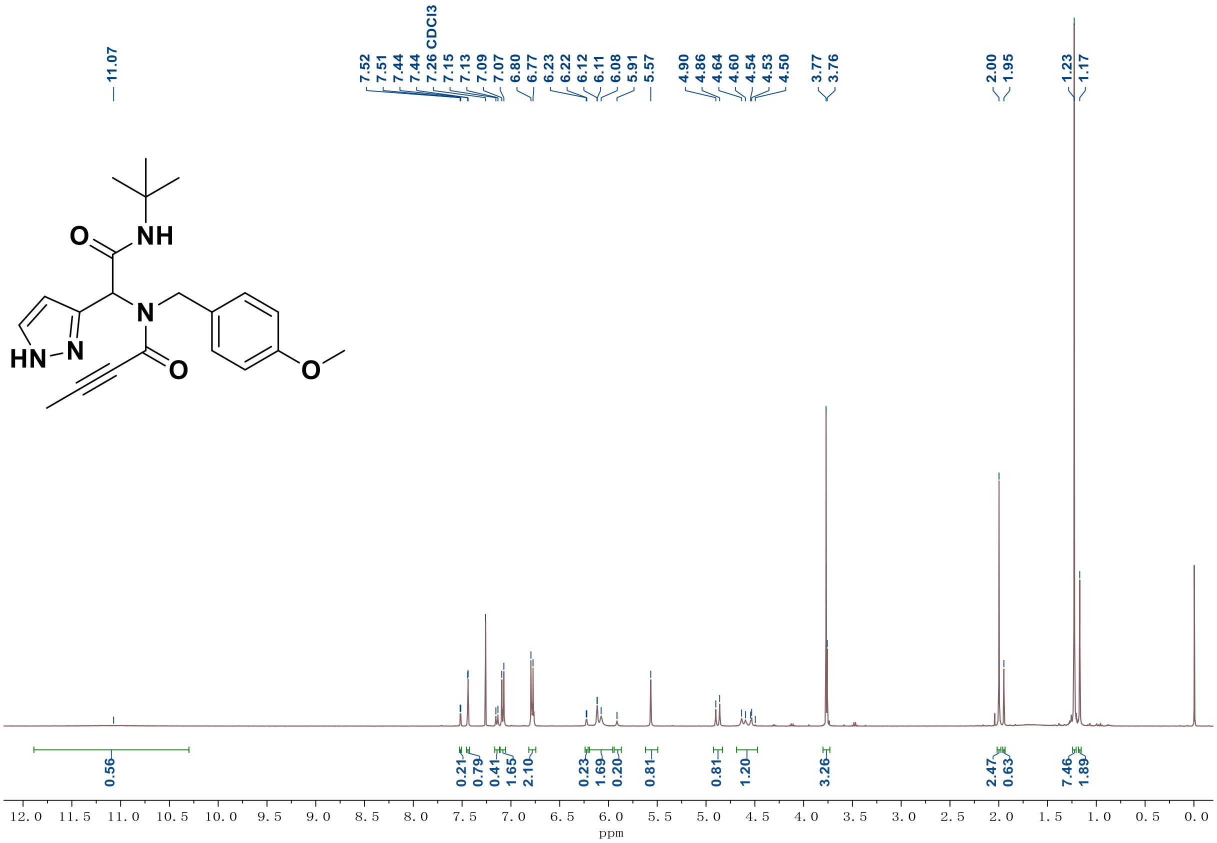

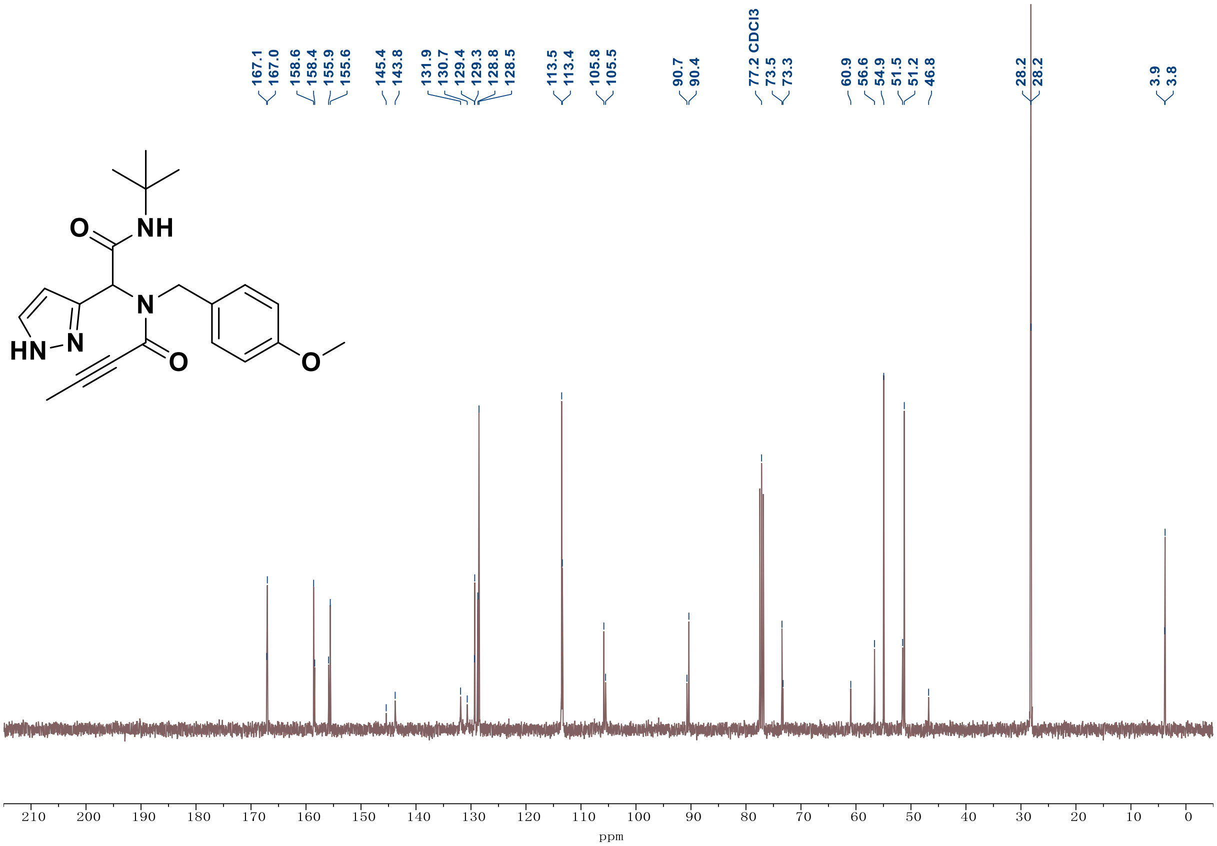

Copies of ^{1}H and $^{13}C\{^{1}H\}$ spectra of compound **15o**

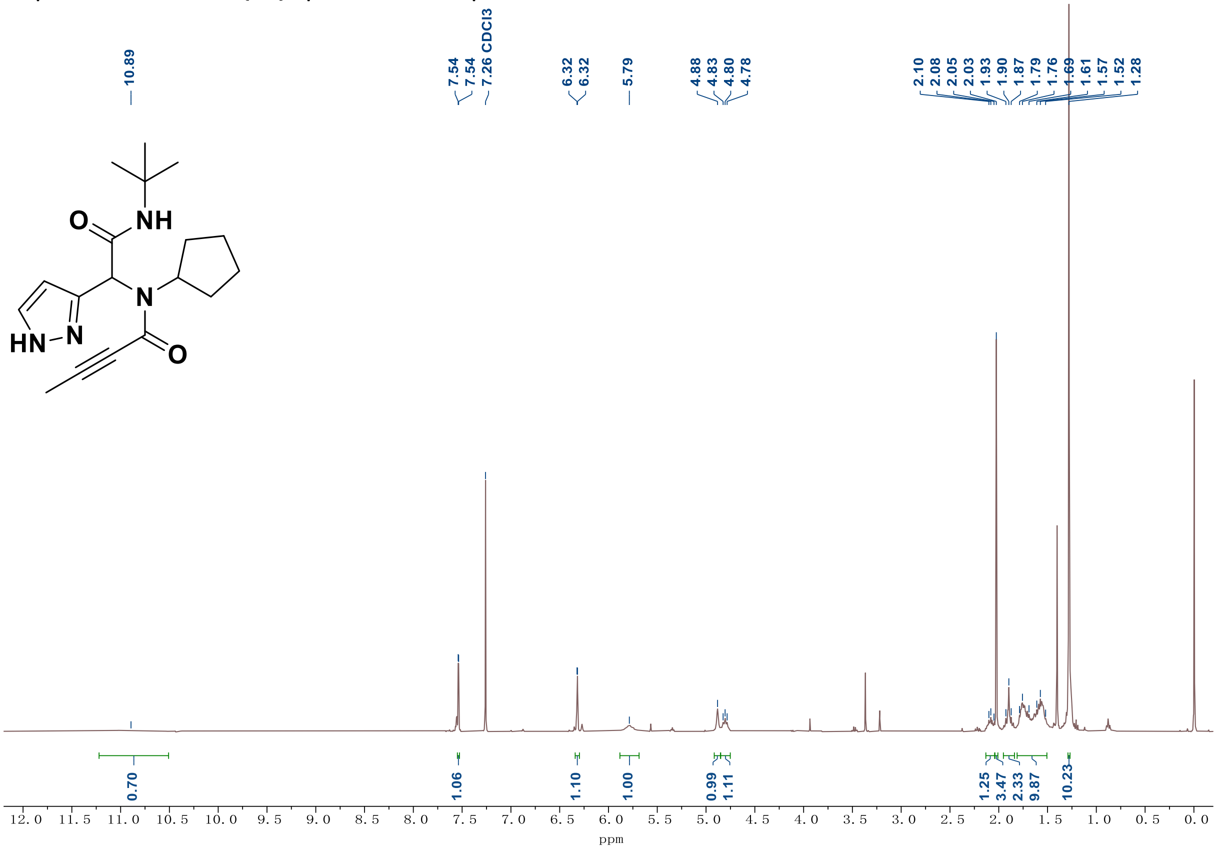

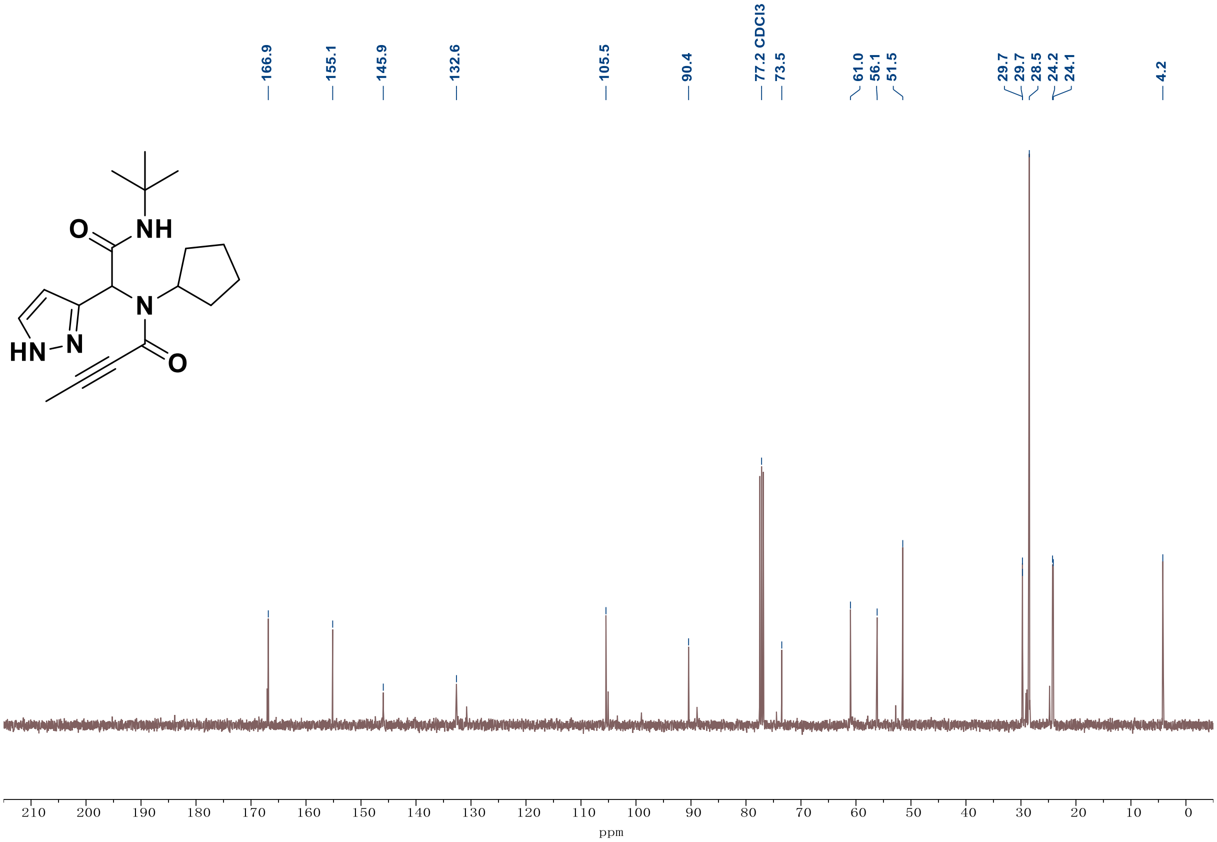

Copies of ^{1}H and ^{13}C{^{1}H} spectra of compound **15p**

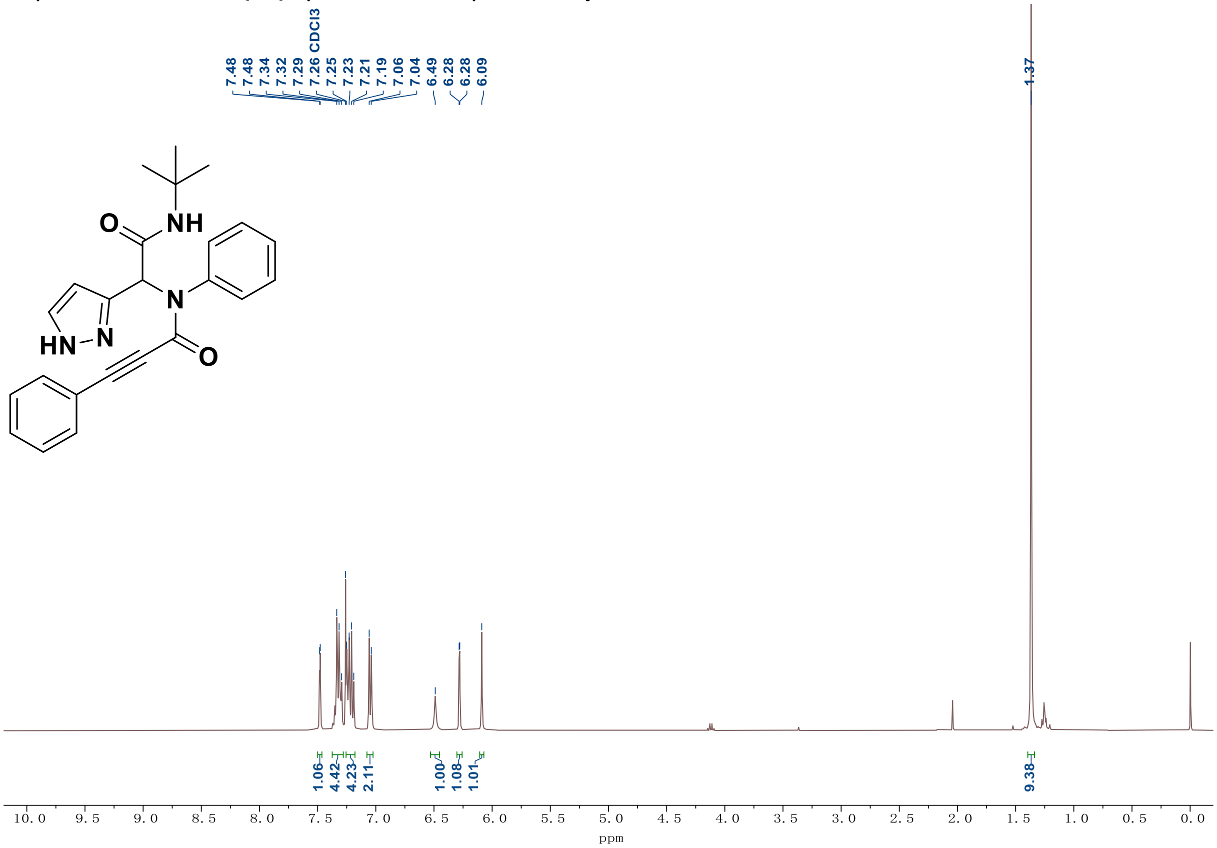

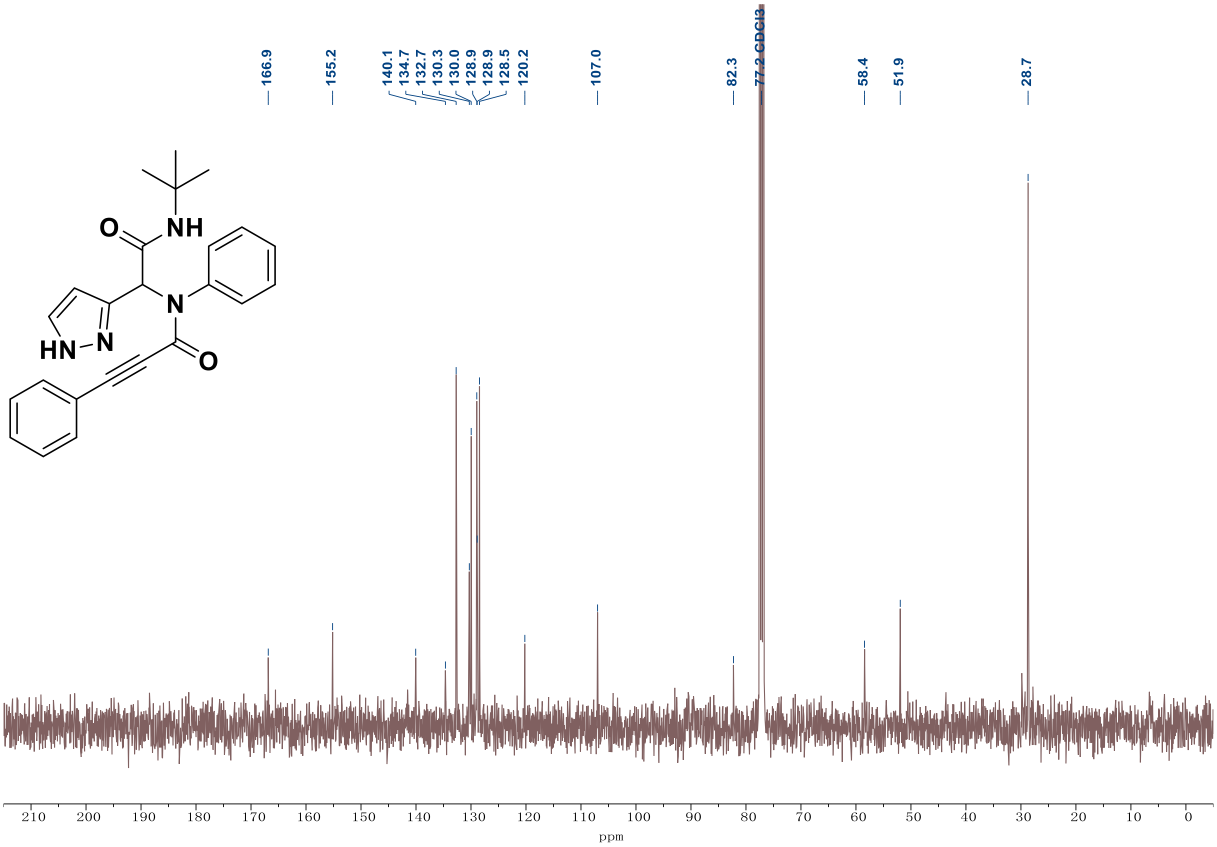

Copies of ^{1}H and $^{13}C\{^{1}H\}$ spectra of compound **15q**

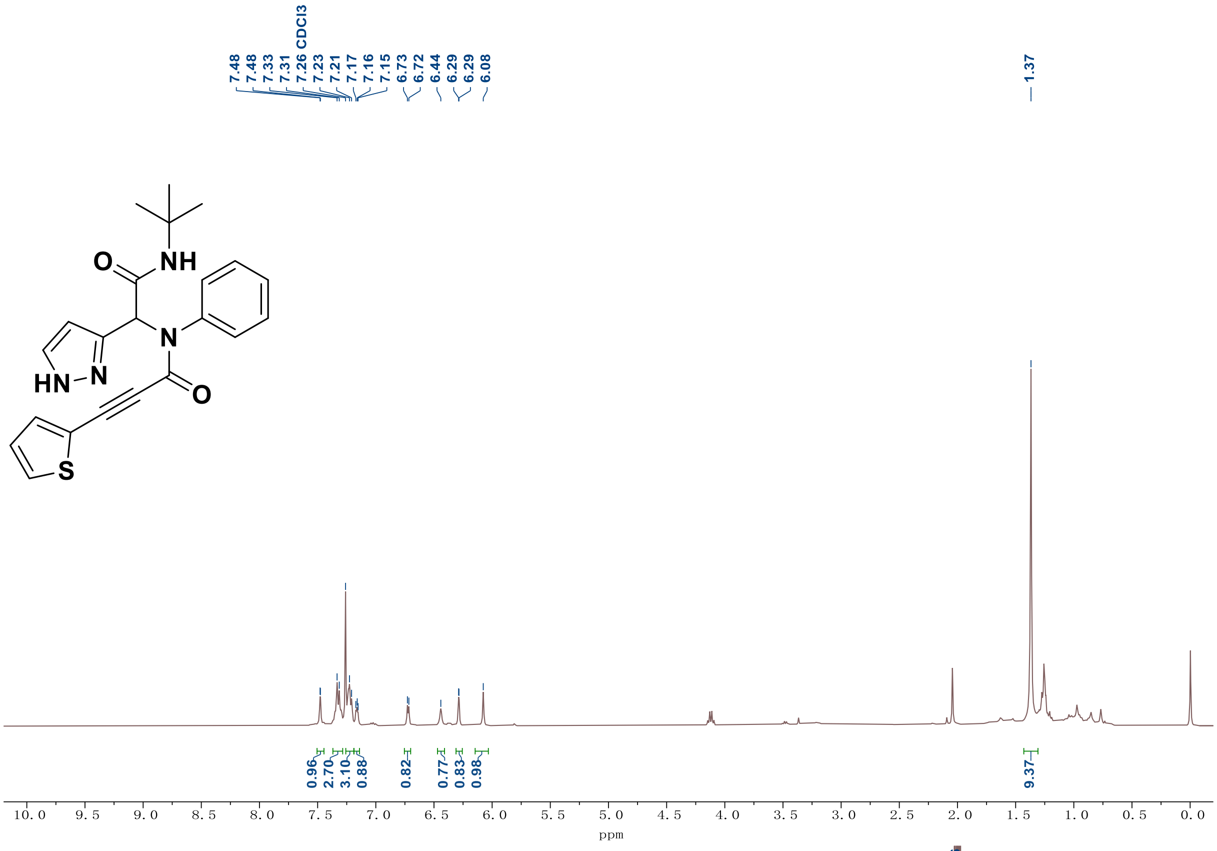

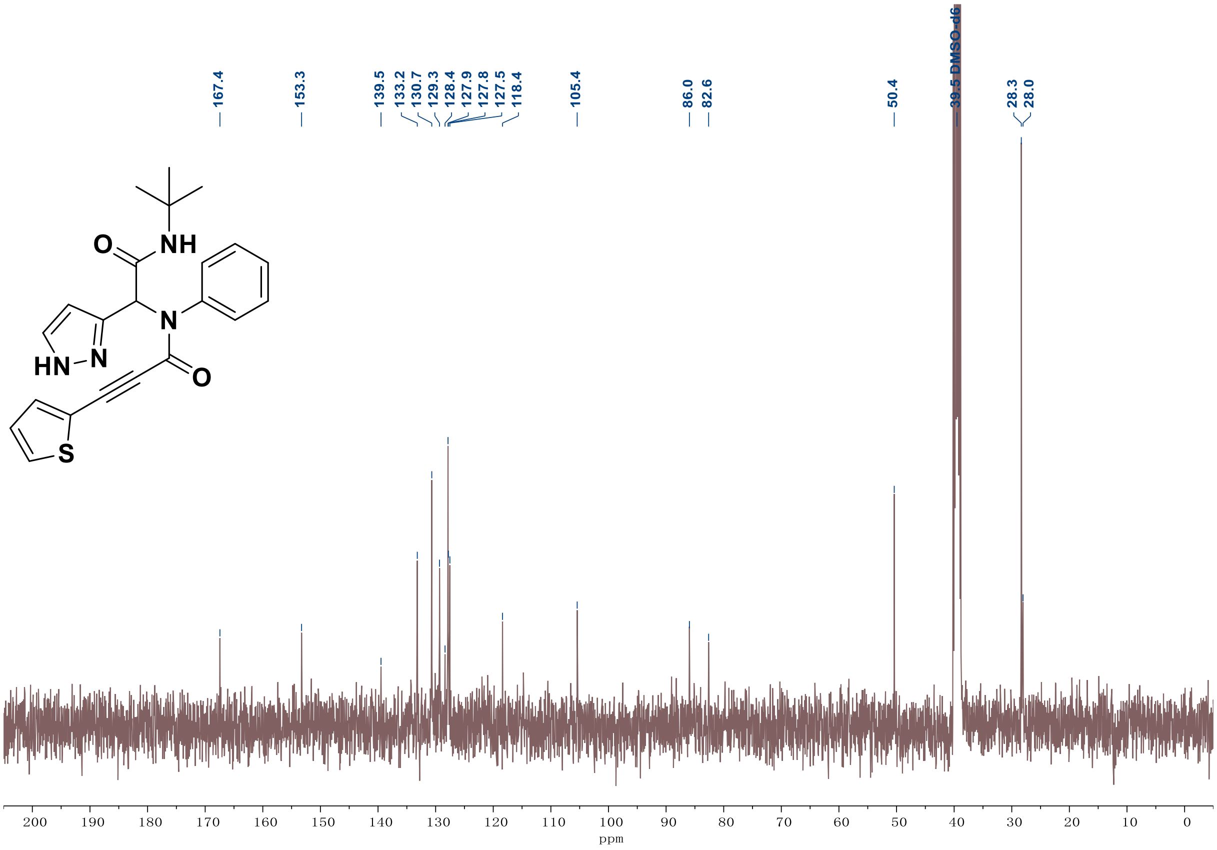

Copies of ^{1}H and ^{13}C{^{1}H} spectra of compound **15r**

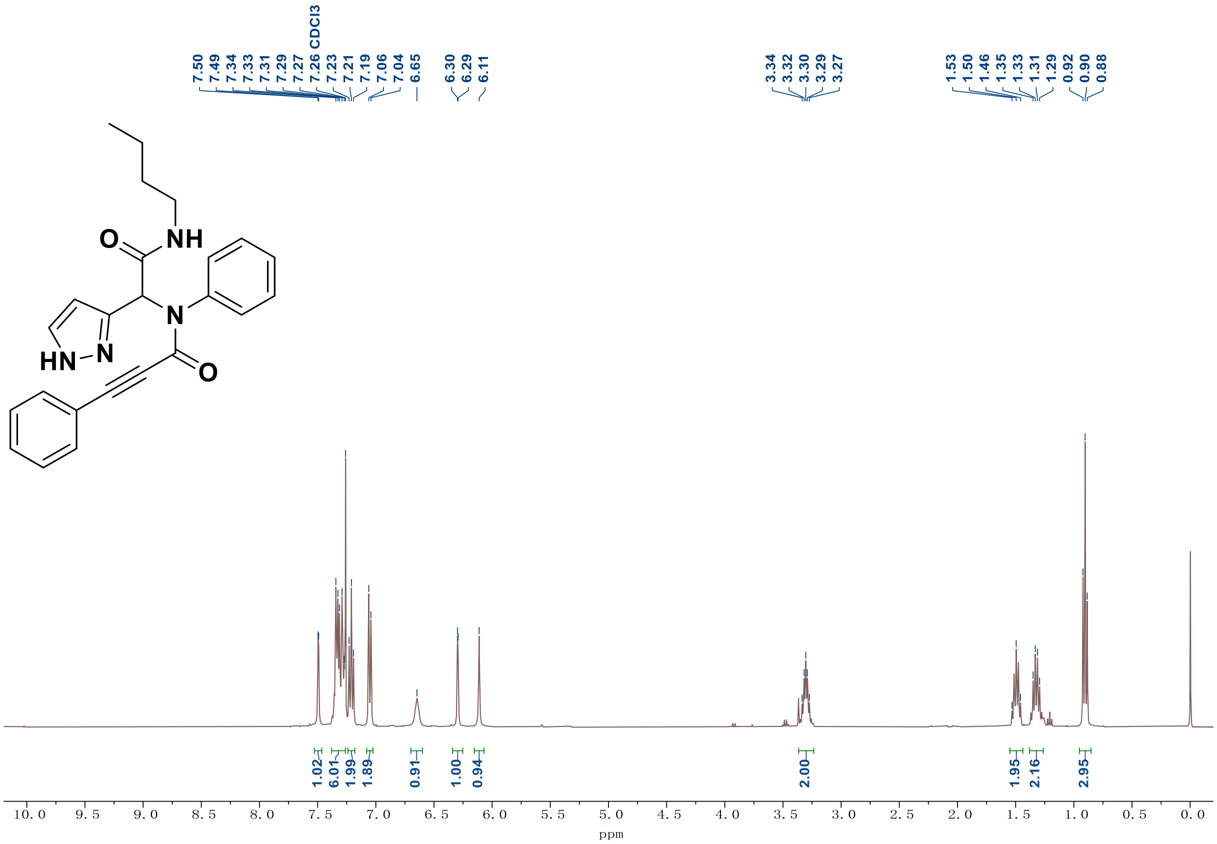

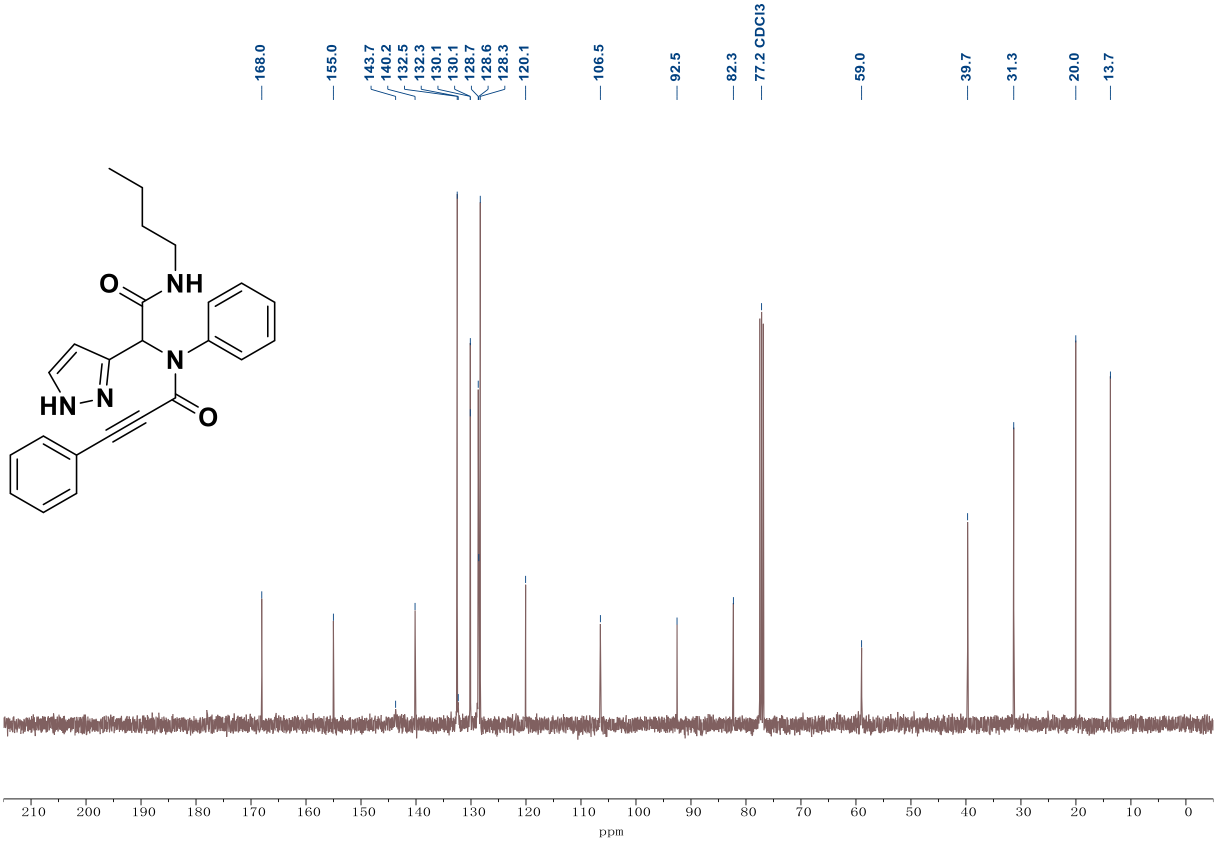

Copies of ^{1}H and $^{13}C\{^{1}H\}$ spectra of compound **15s**

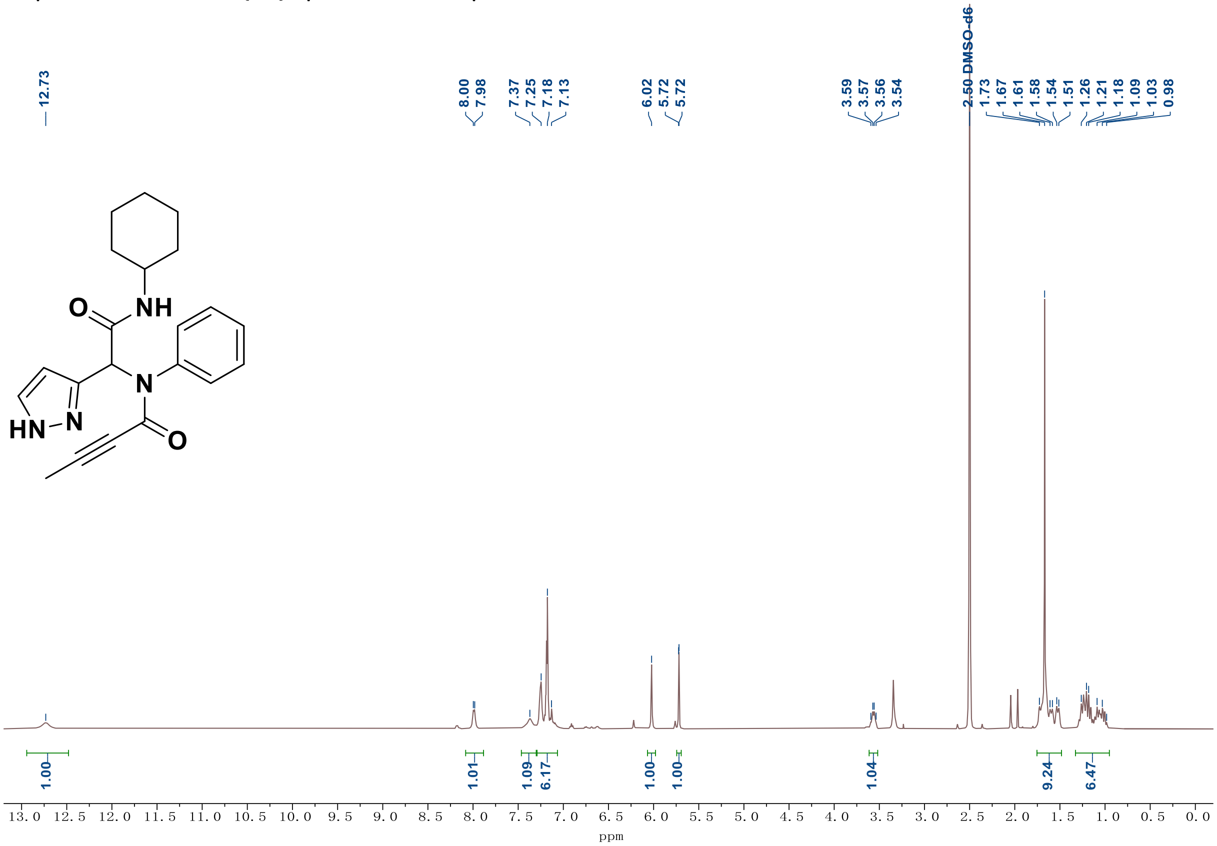

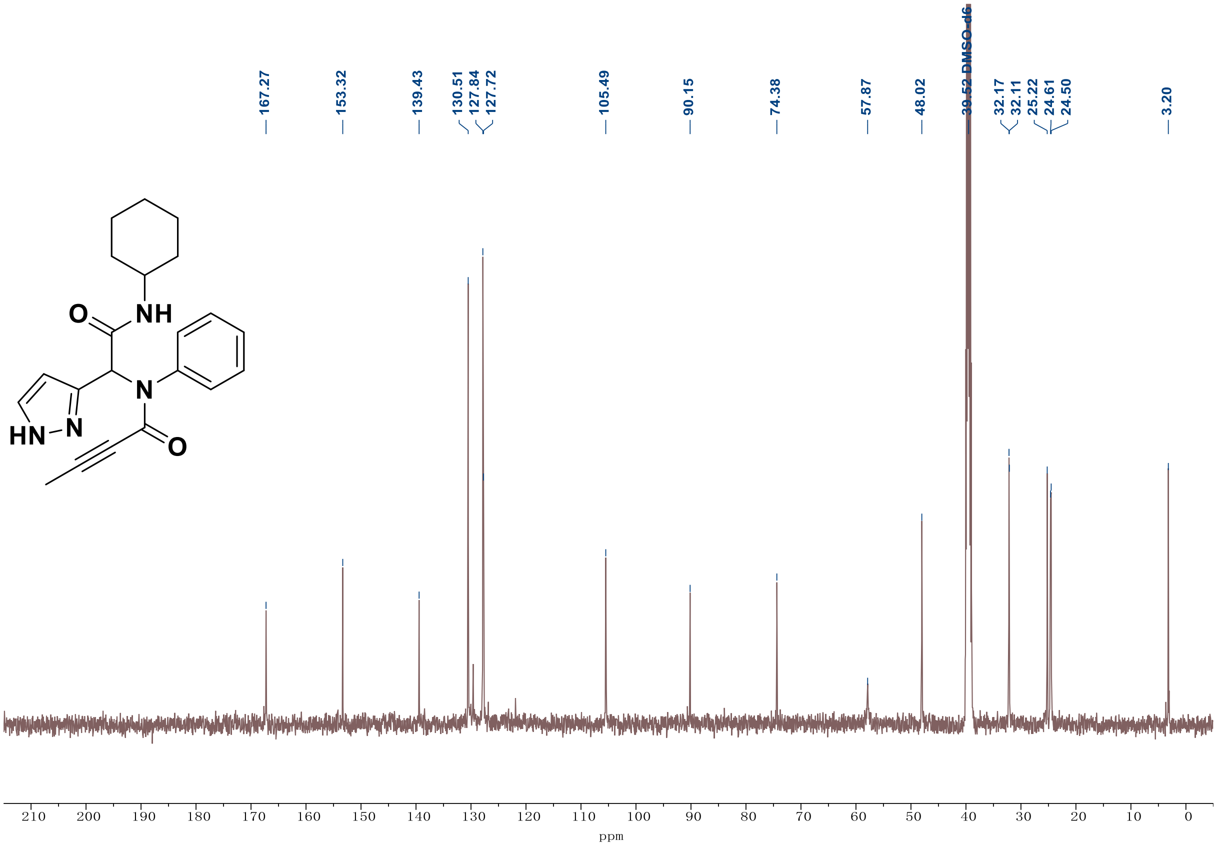

Copies of ^{1}H and $^{13}C\{^{1}H\}$ spectra of compound **15t**

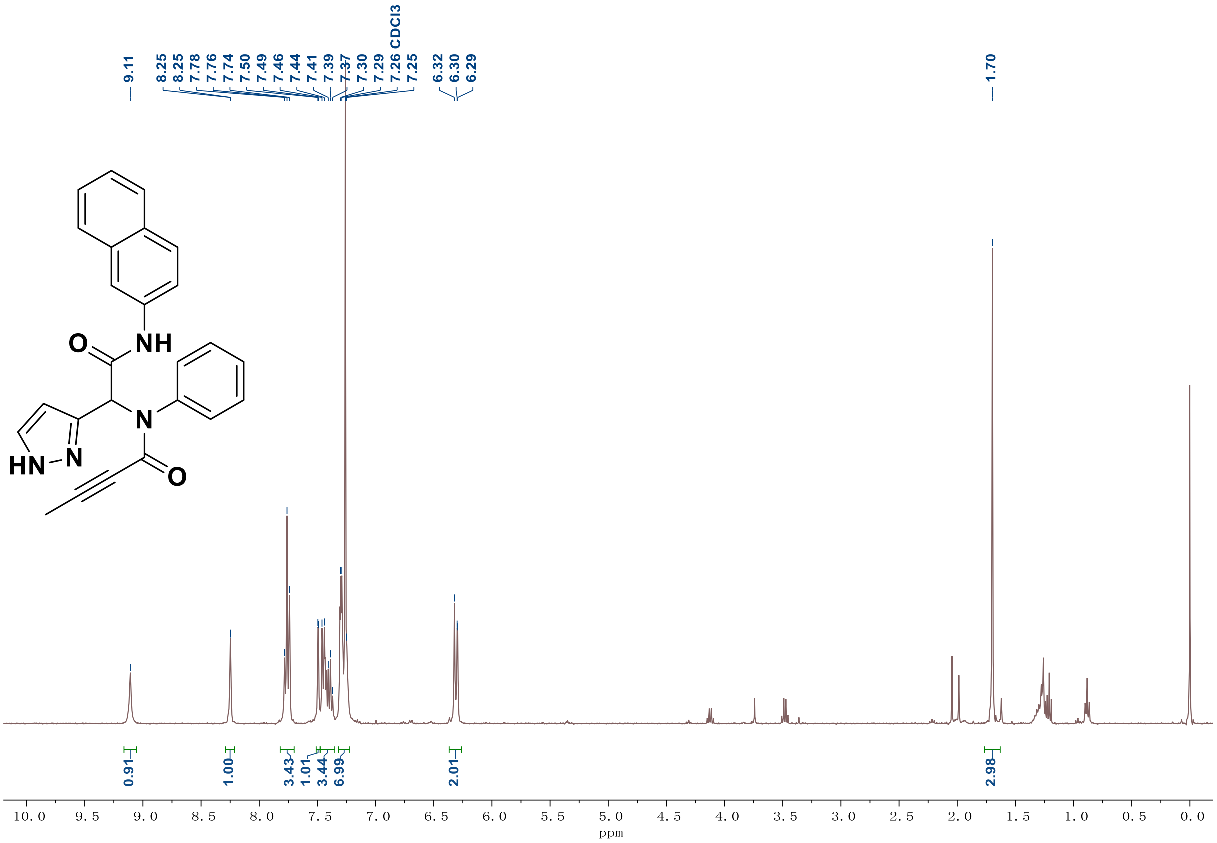

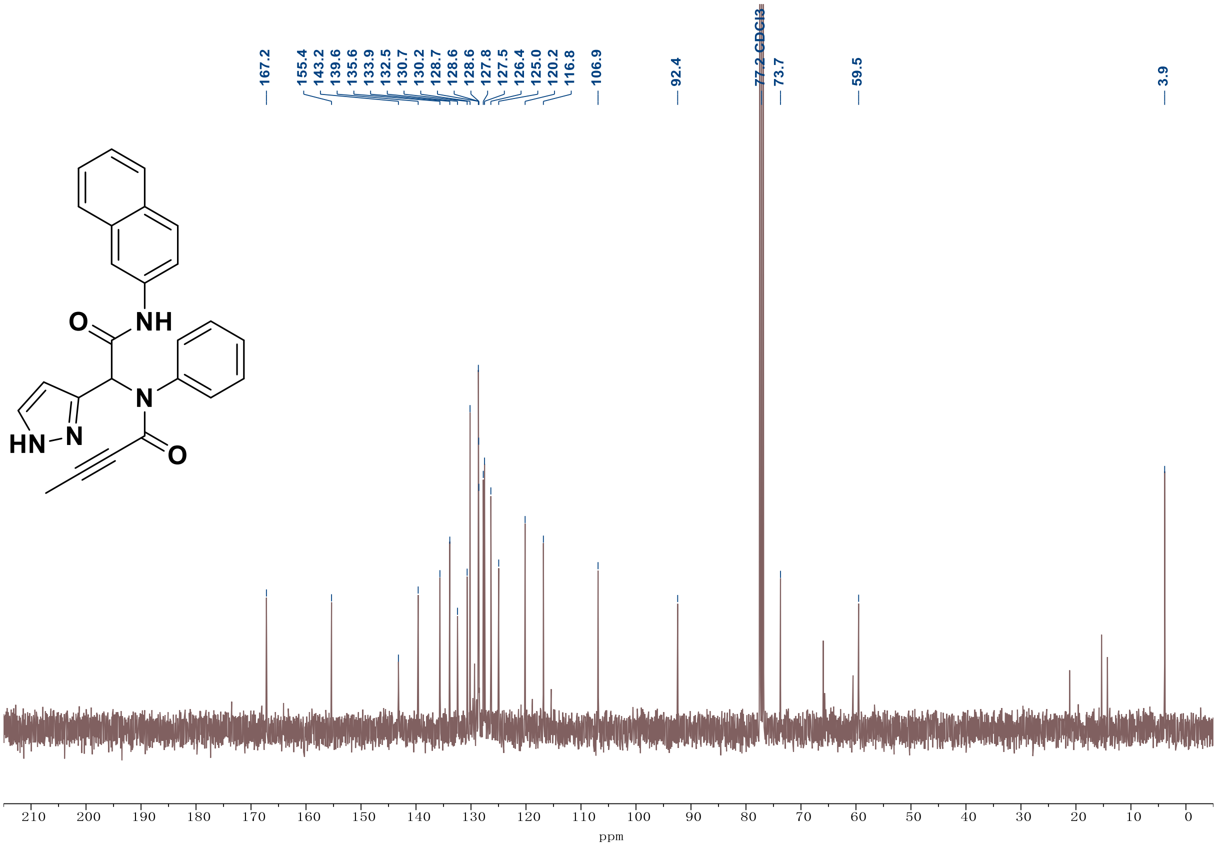

Copies of ^{1}H and $^{13}C\{^{1}H\}$ spectra of compound **15u**

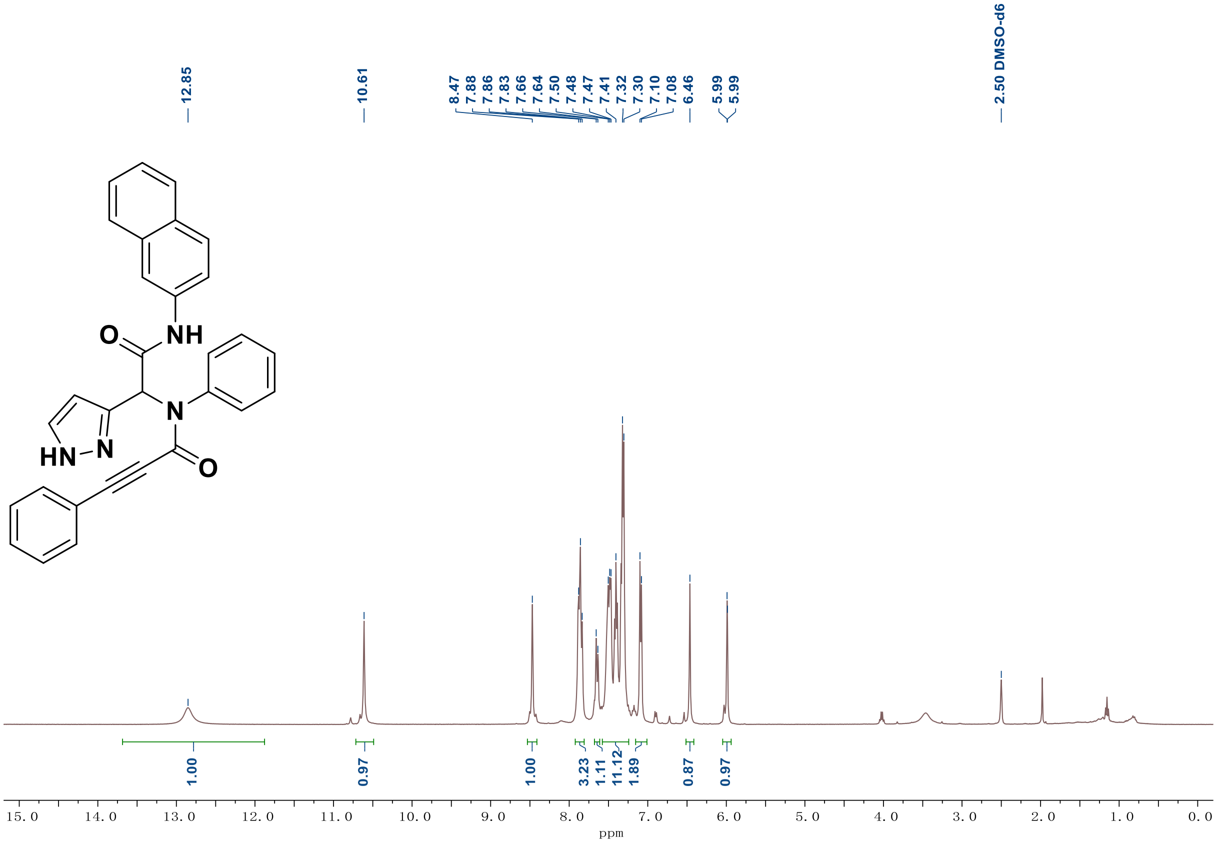

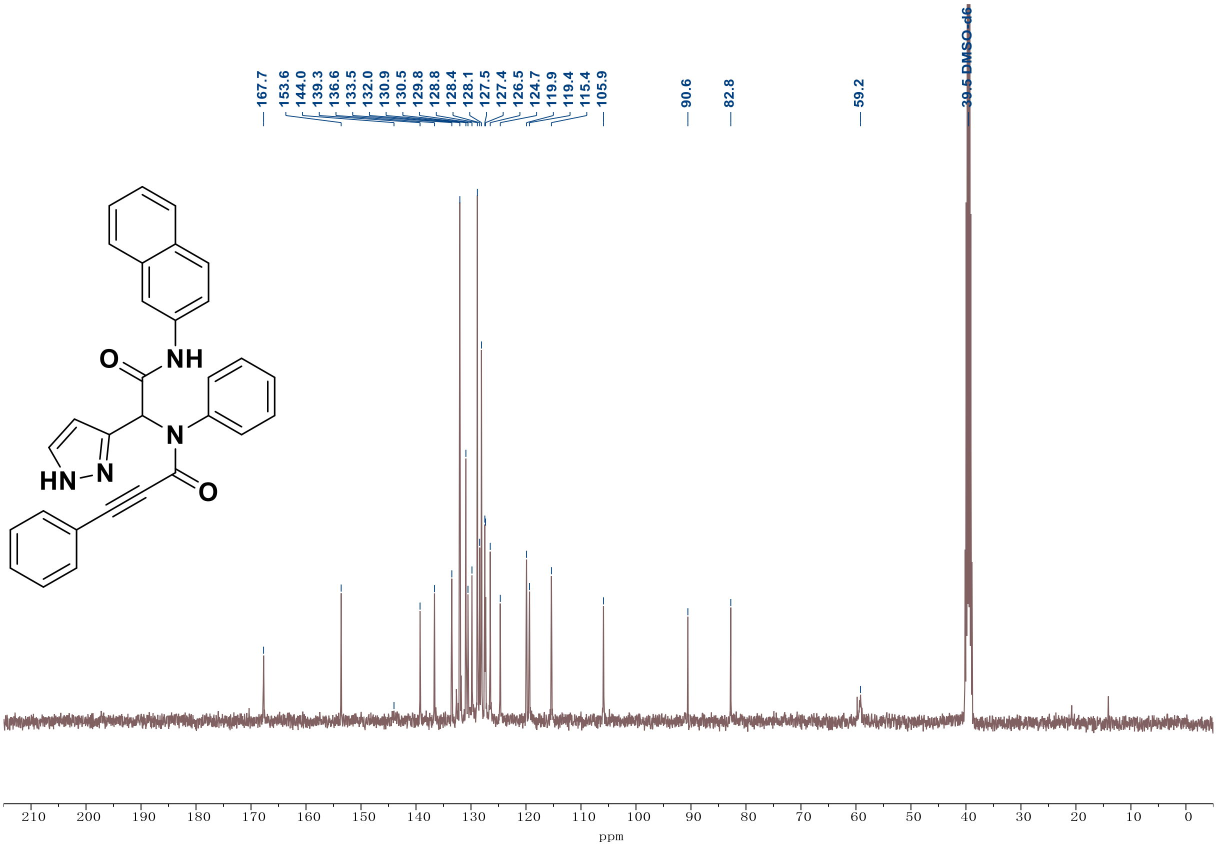

Copies of ^{1}H and $^{13}C\{^{1}H\}$ spectra of compound **15v**

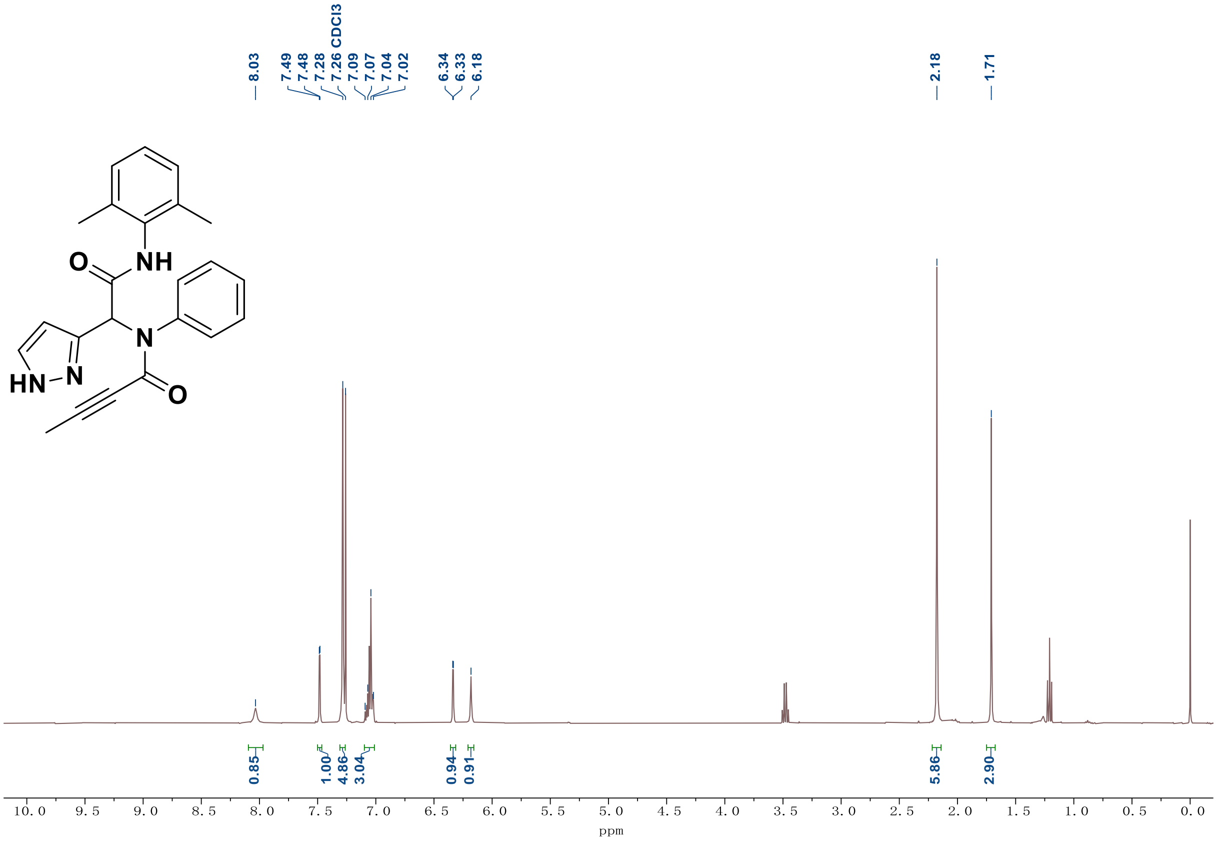

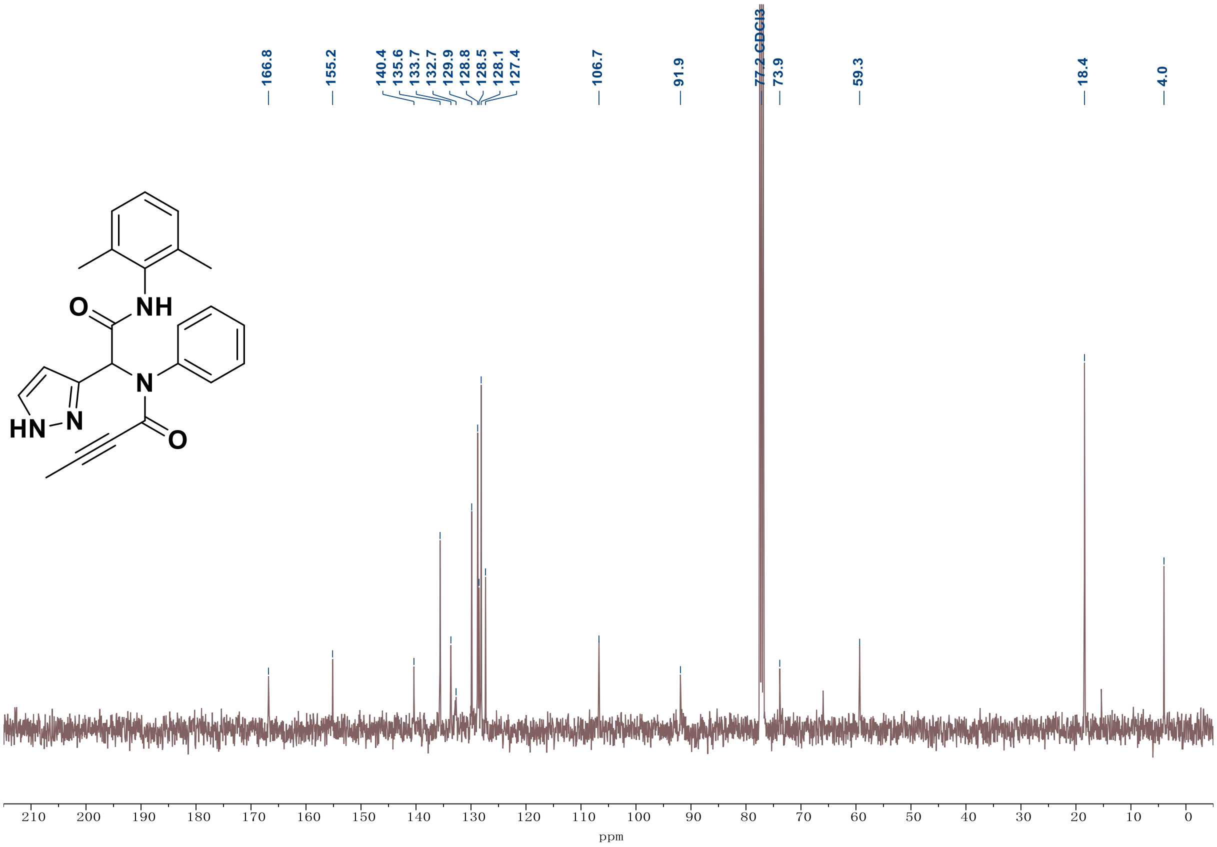

Copies of ^{1}H and ^{13}C{^{1}H} spectra of compound **15w**

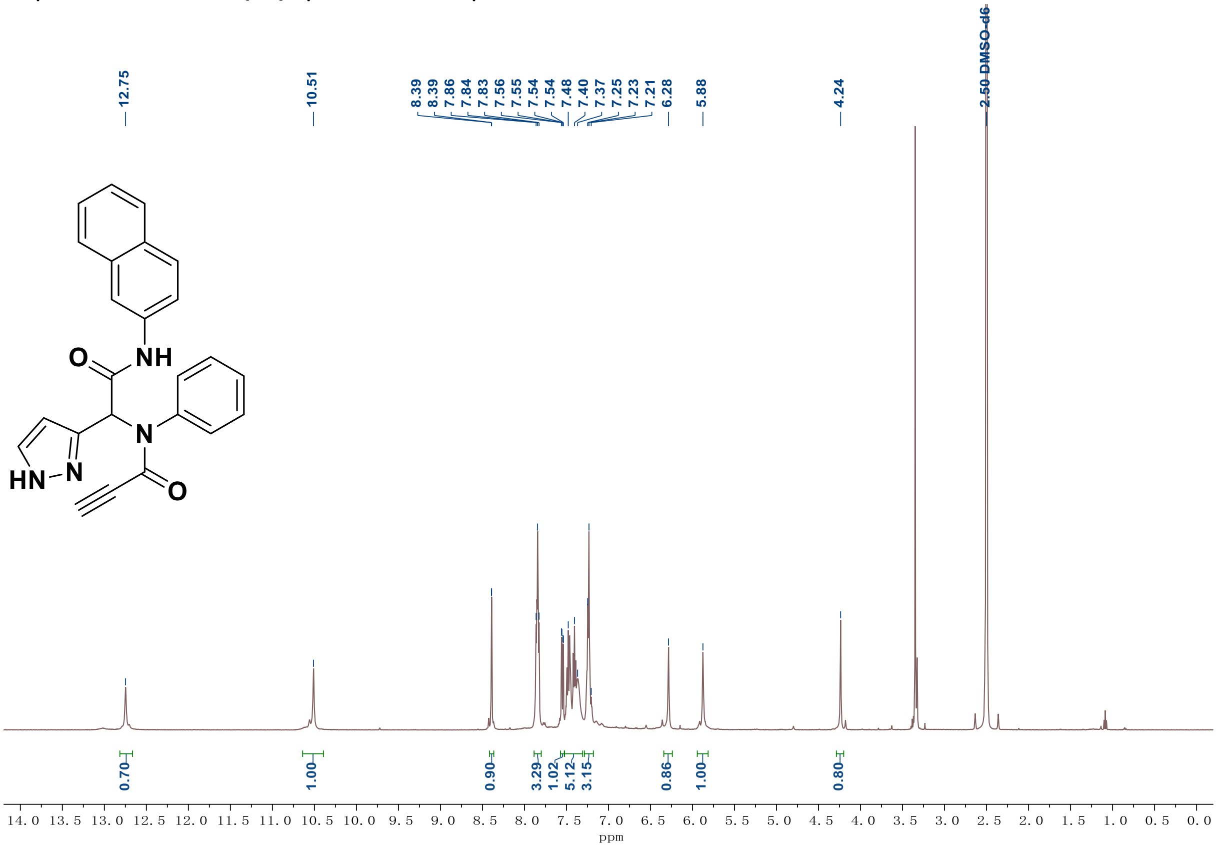

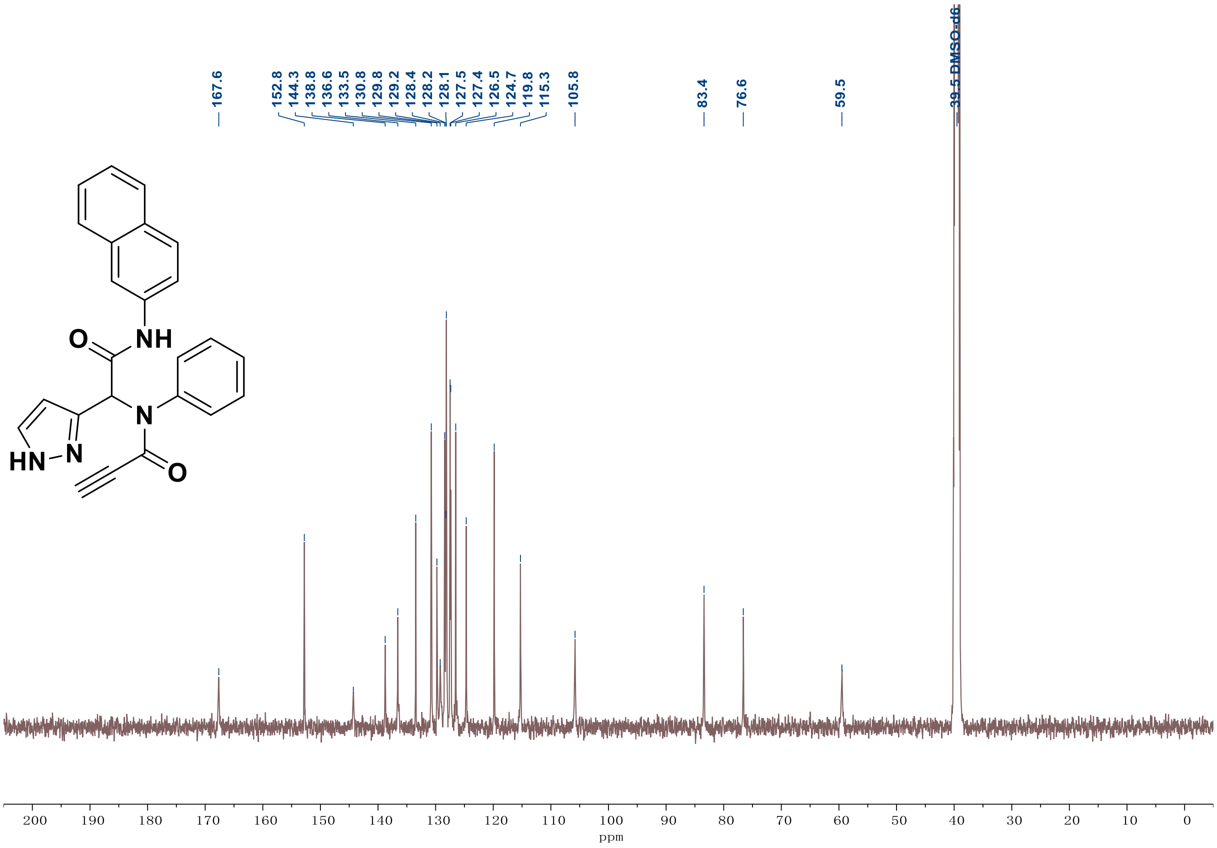

Copies of ^{1}H and ^{13}C{^{1}H} spectra of compound **15x**

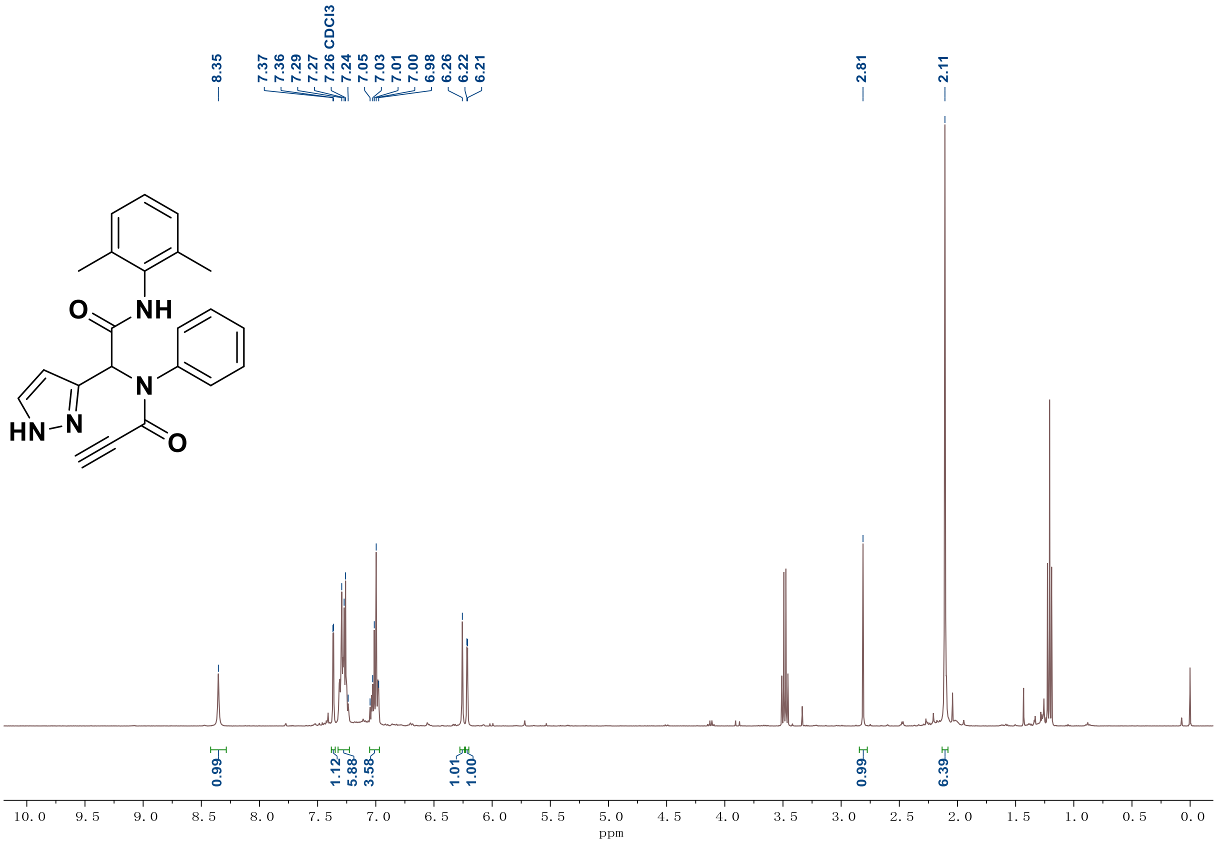

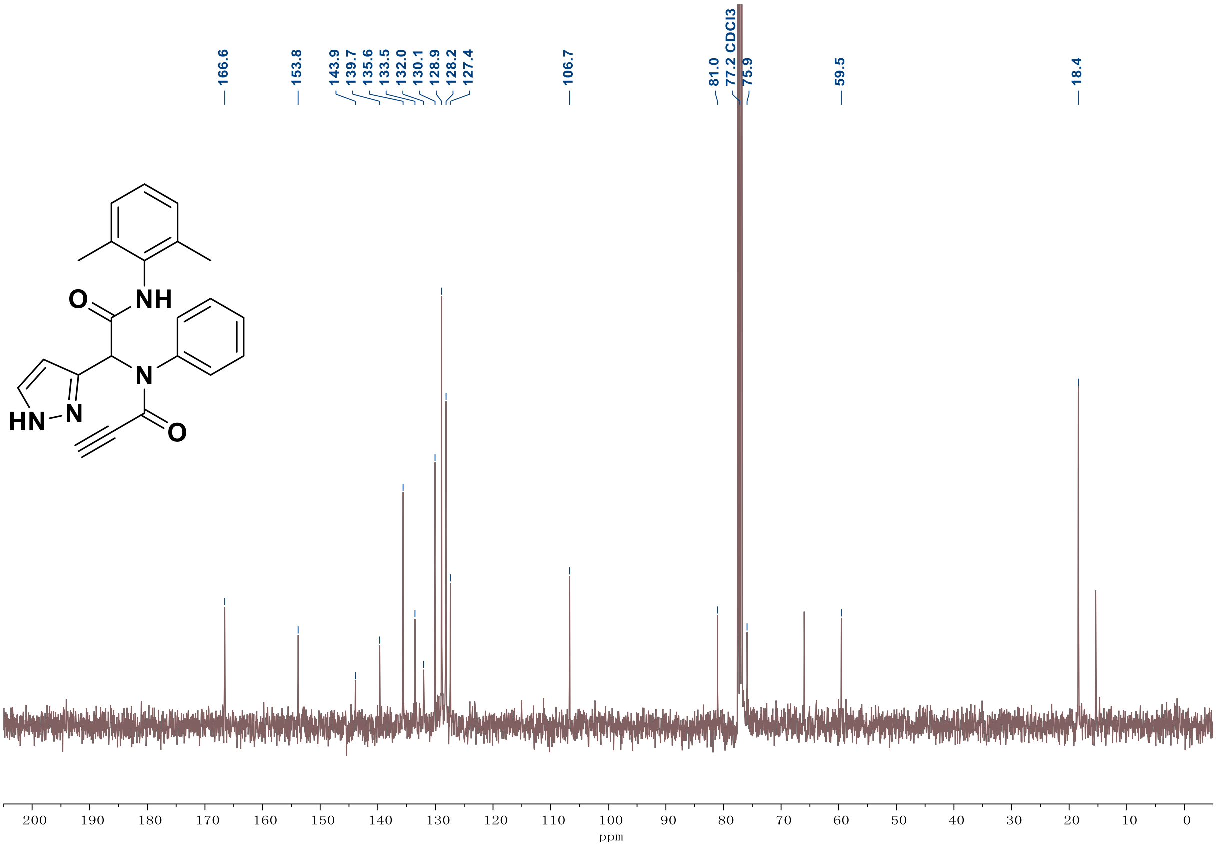

Copies of ^{1}H and $^{13}C\{^{1}H\}$ spectra of compound **15y**

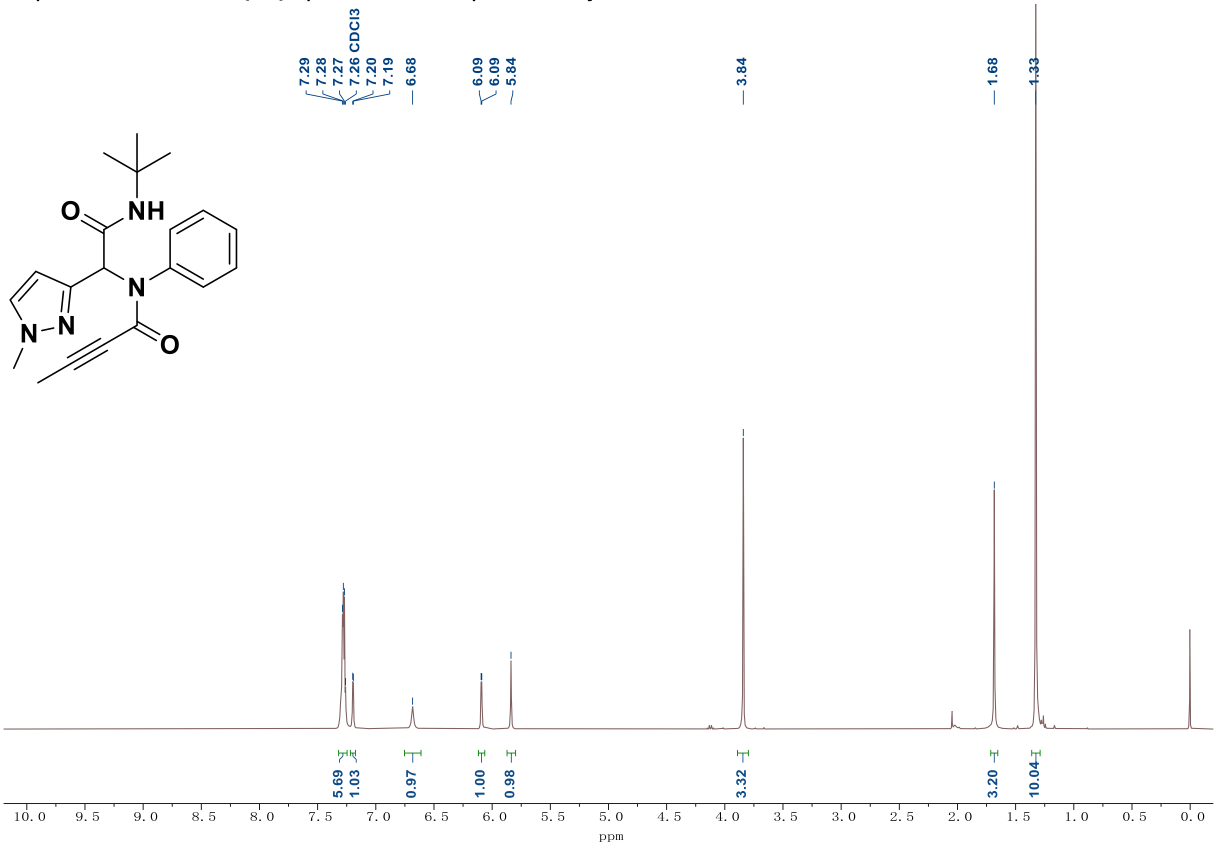

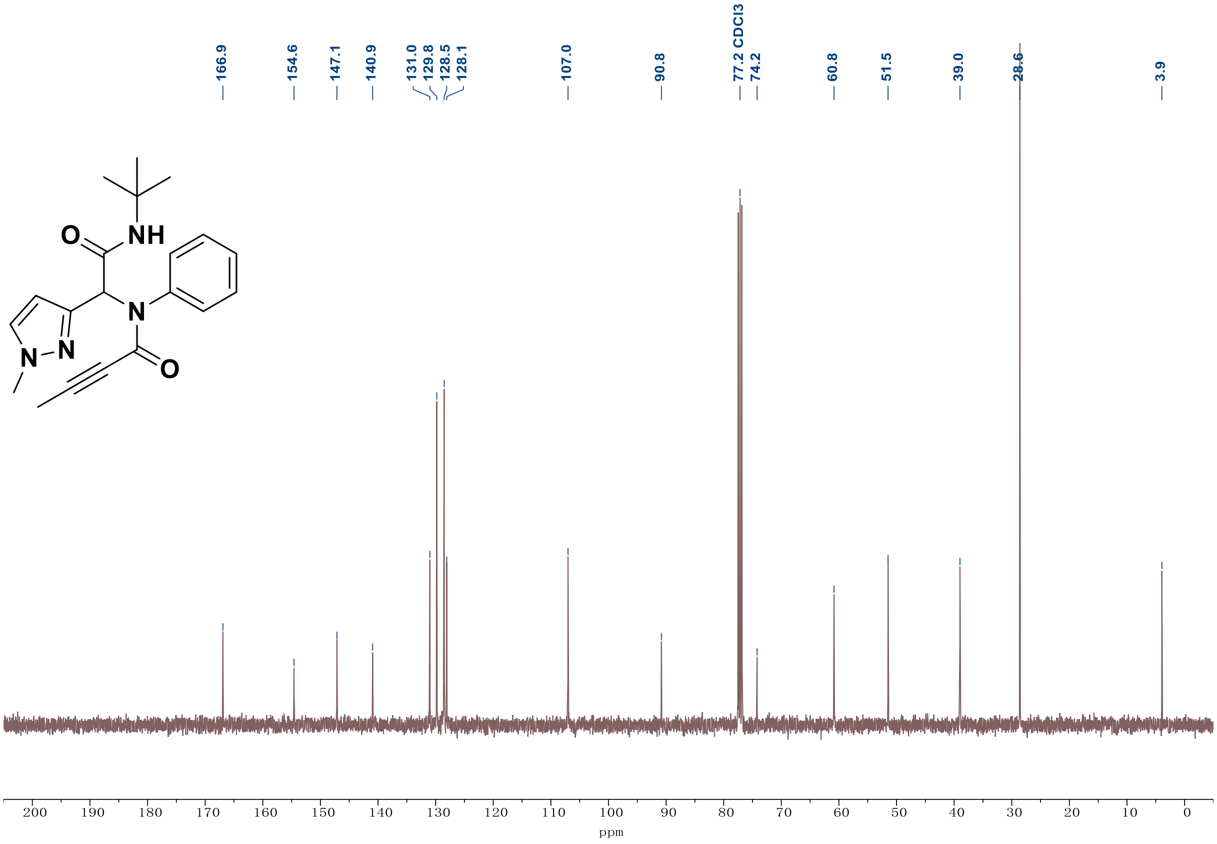

Copies of ^{1}H and $^{13}C\{^{1}H\}$ spectra of compound **15z**

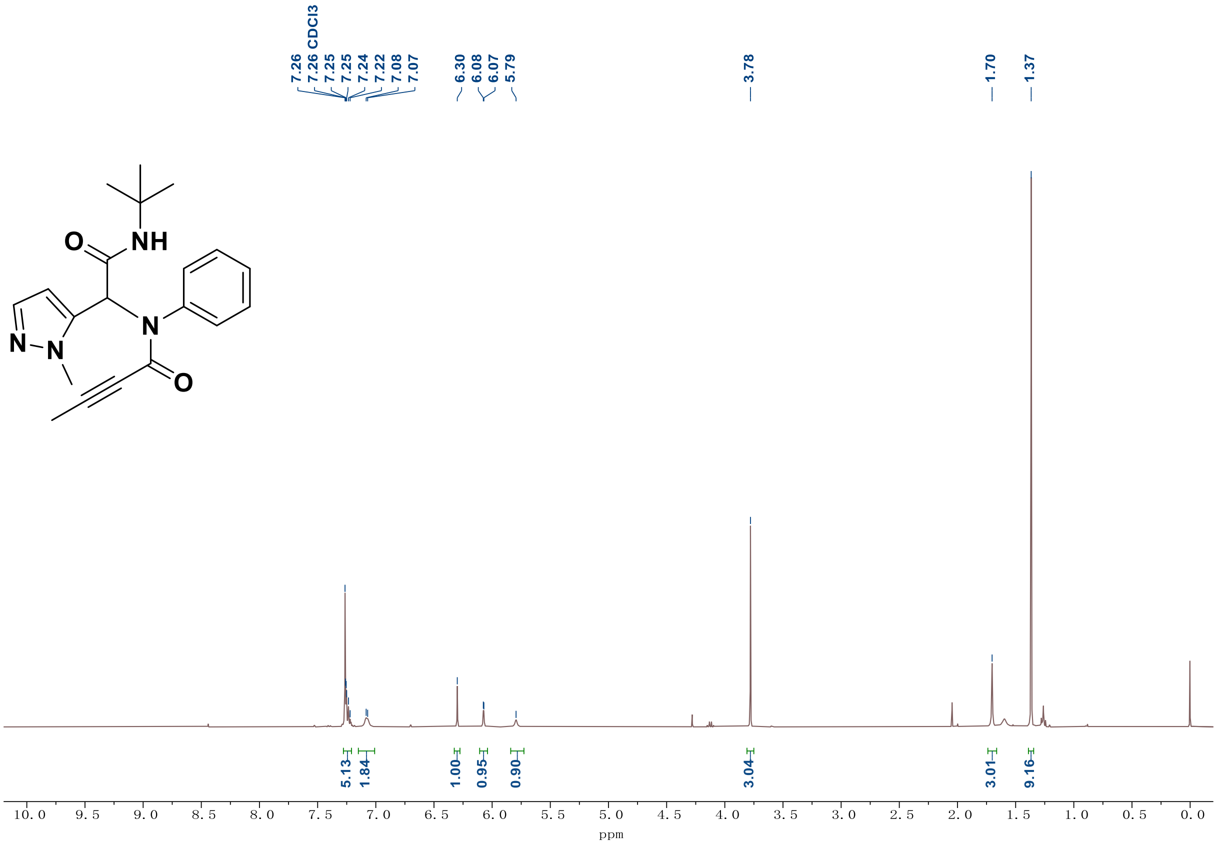

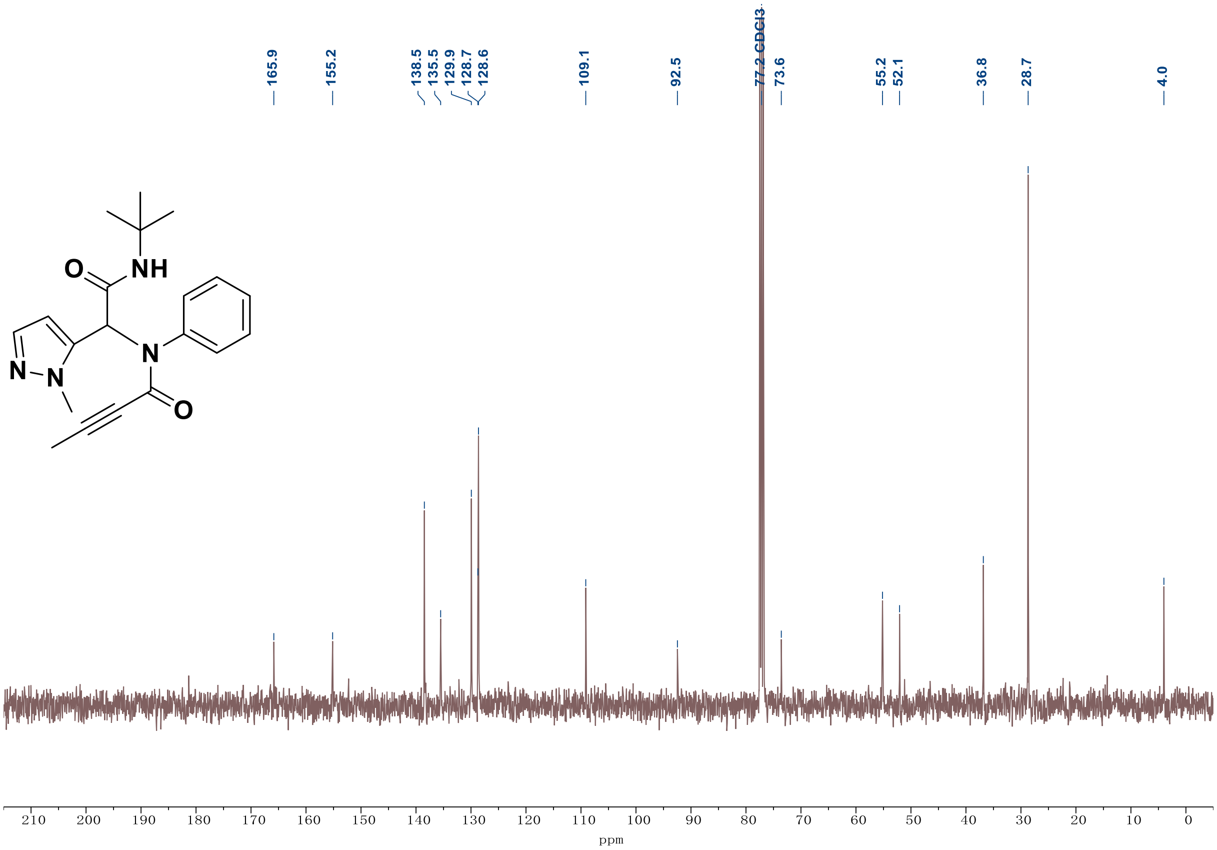

Copies of ^{1}H and $^{13}C\{^{1}H\}$ spectra of compound **16a**

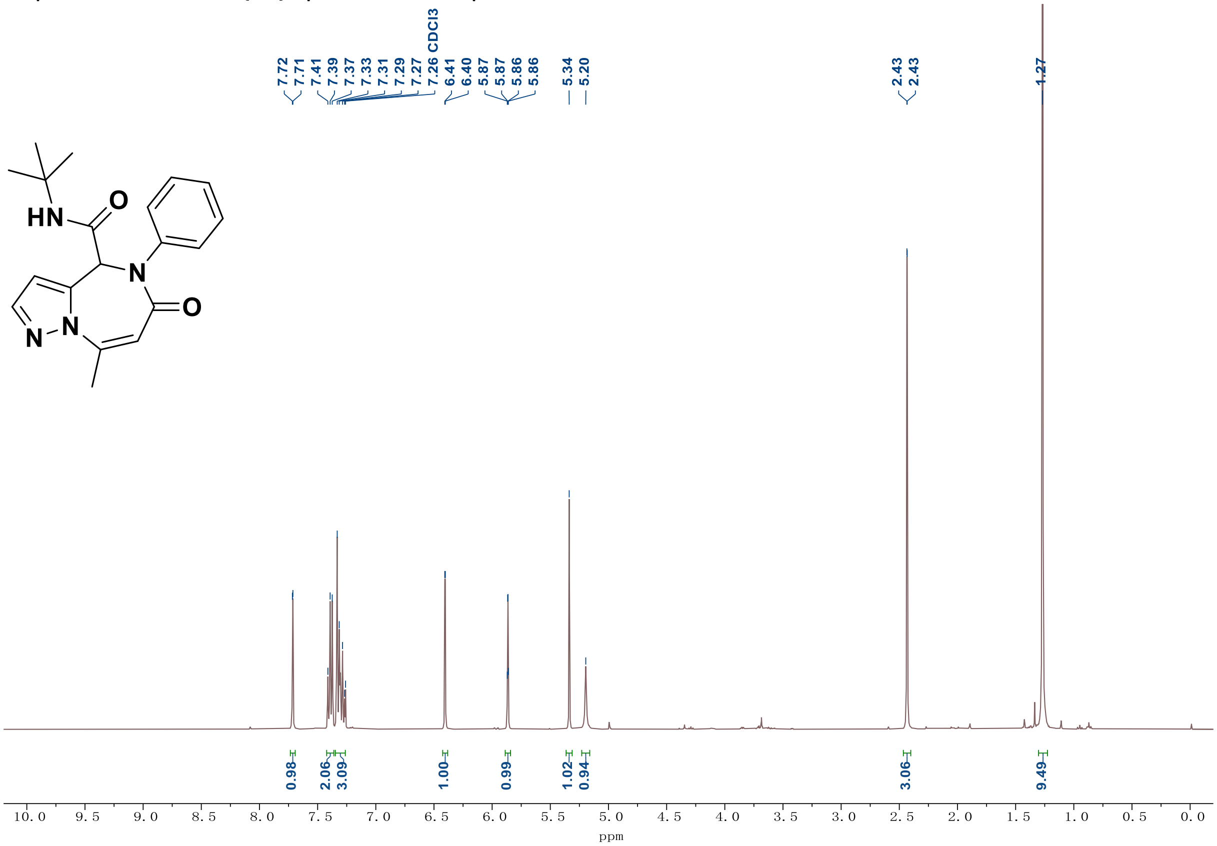

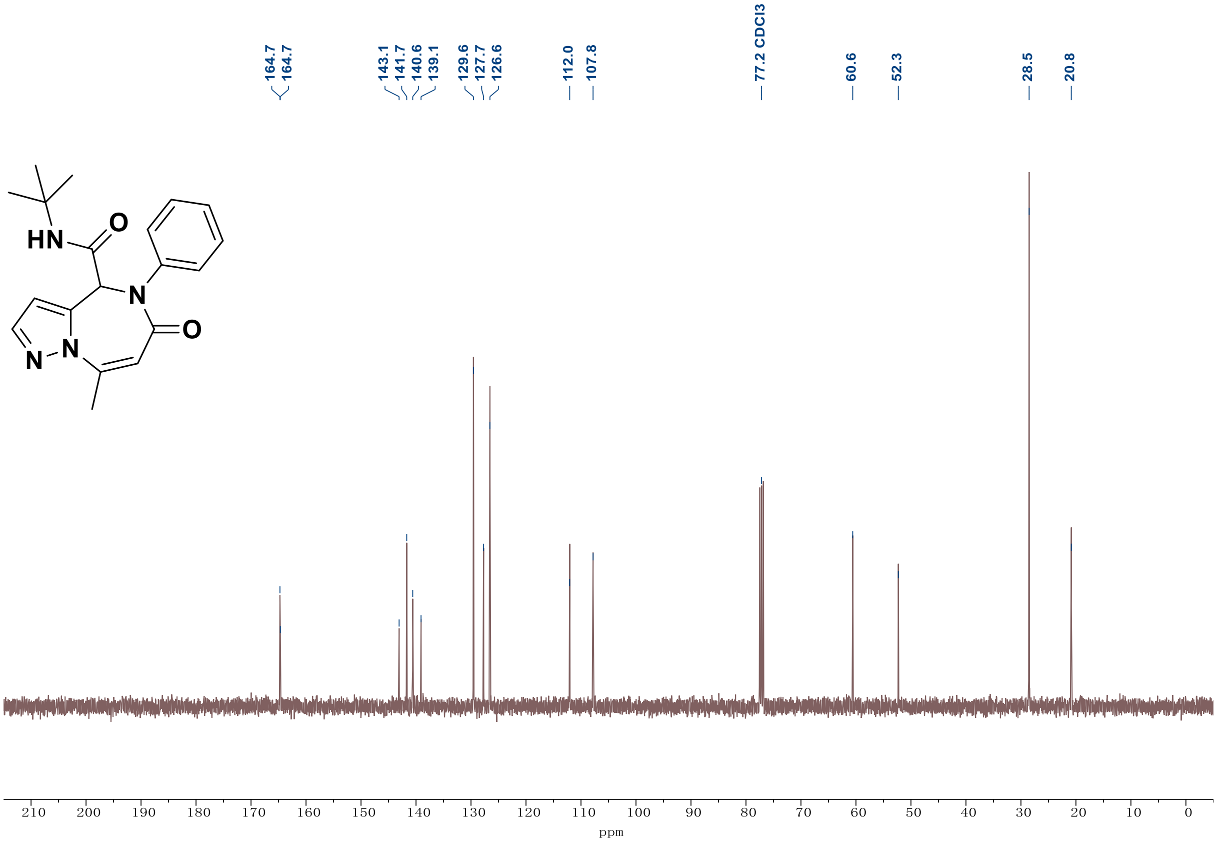

Copies of ^{1}H and ^{13}C{^{1}H} spectra of compound **16b**

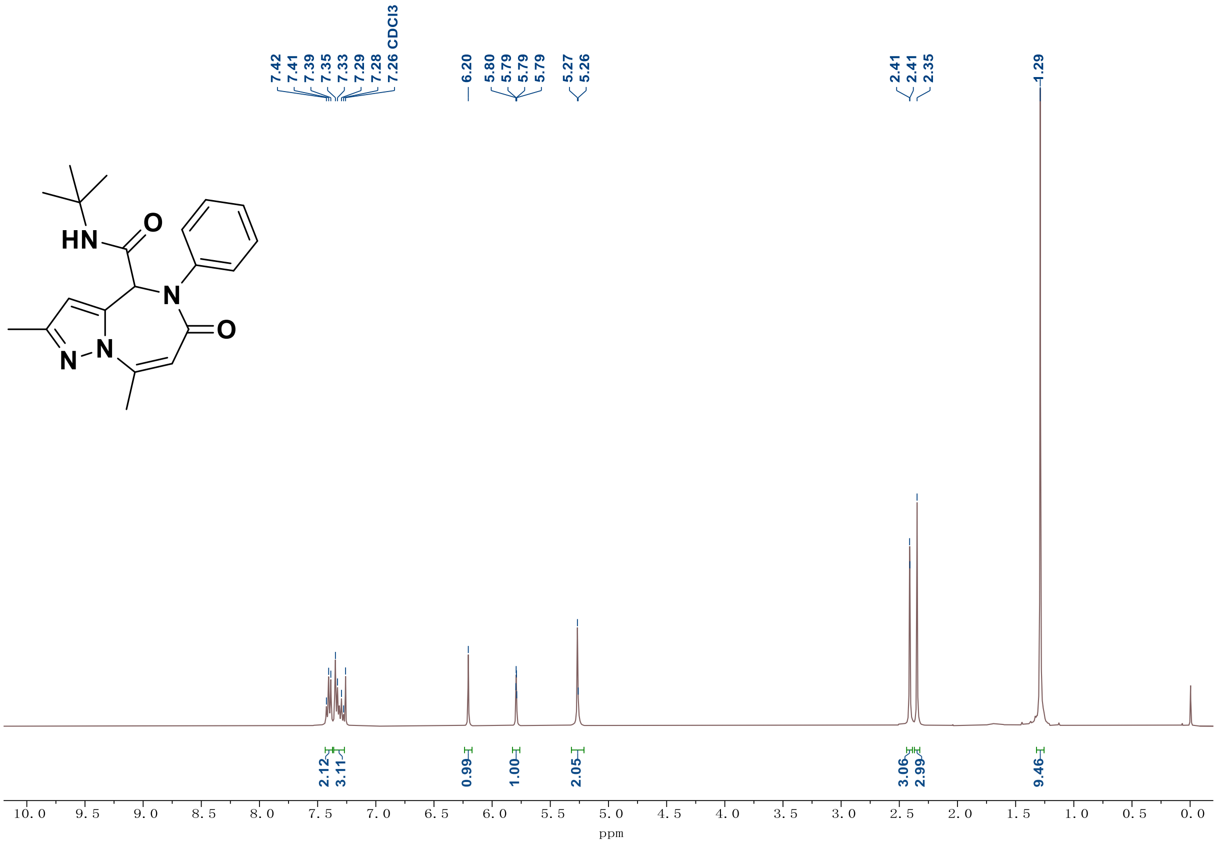

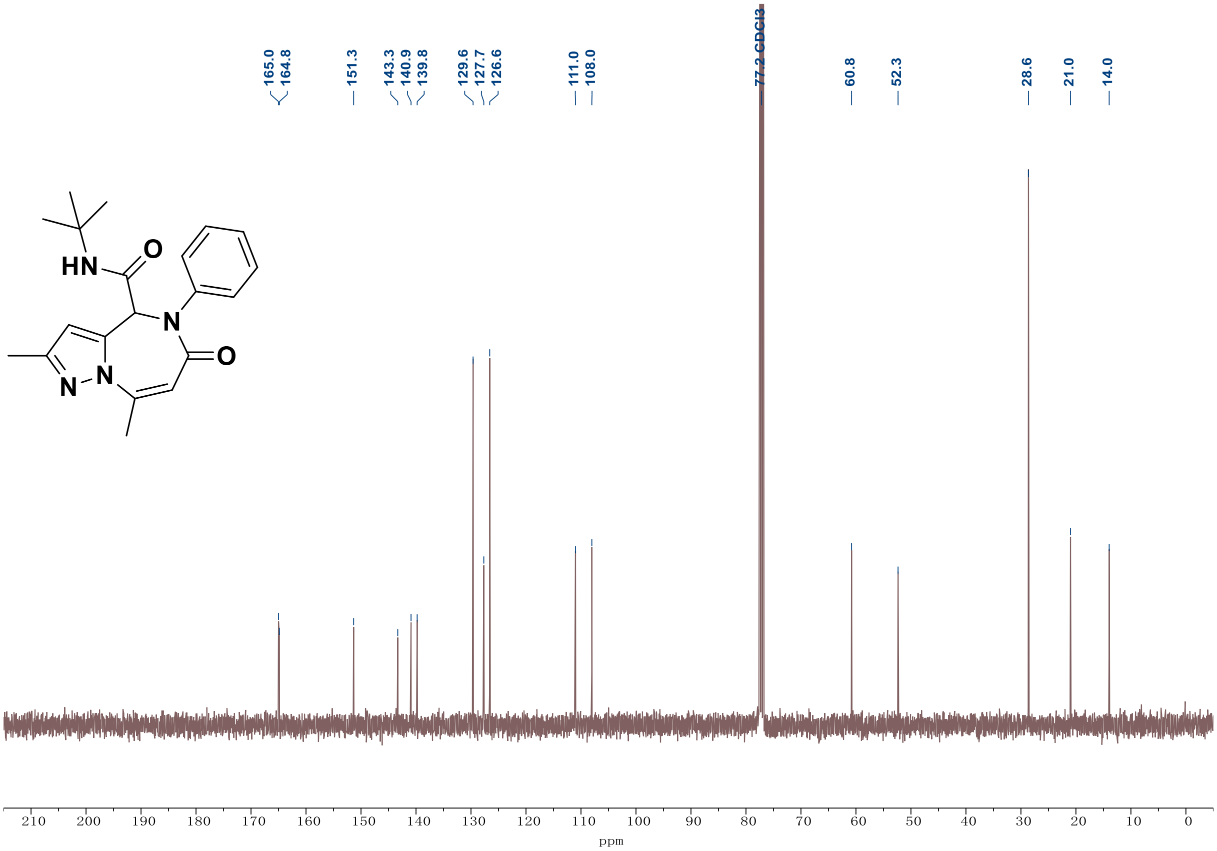

Copies of ^{1}H and $^{13}C\{^{1}H\}$ spectra of compound **16c**

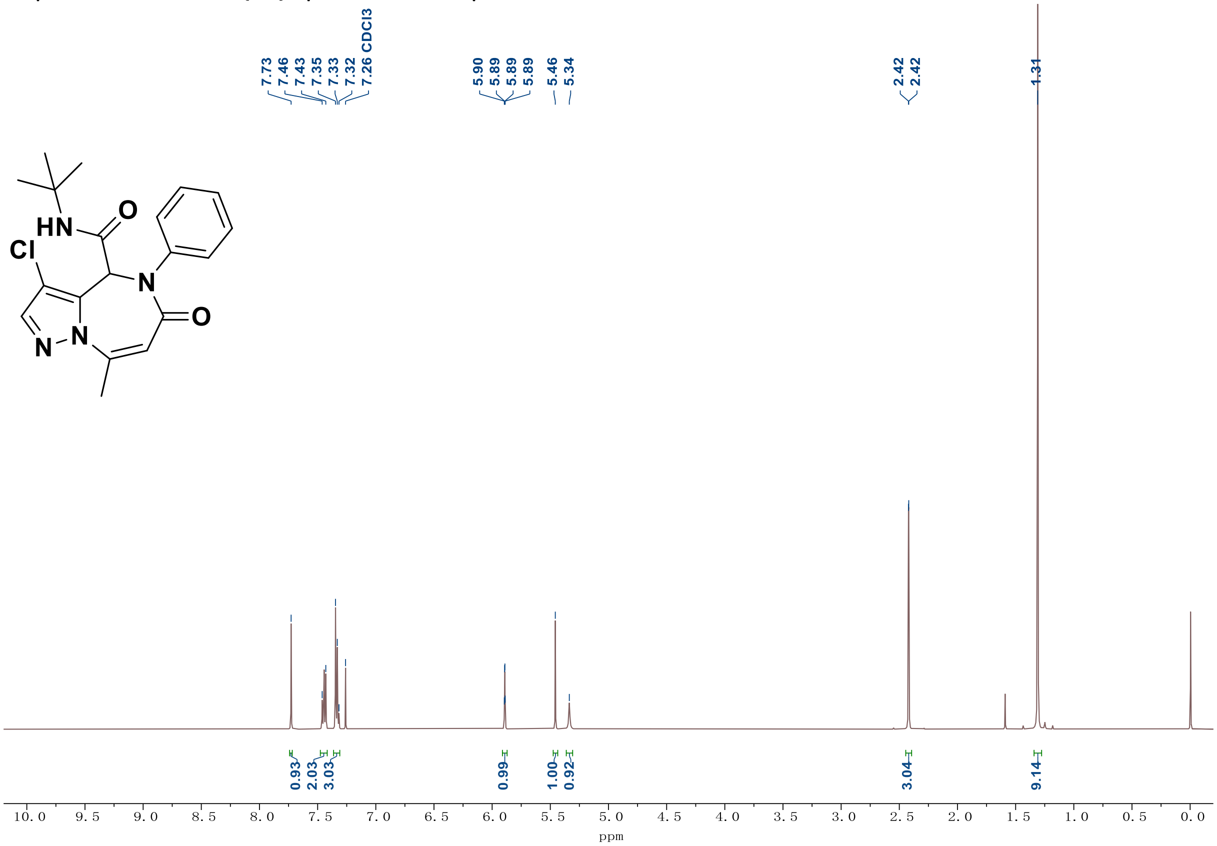

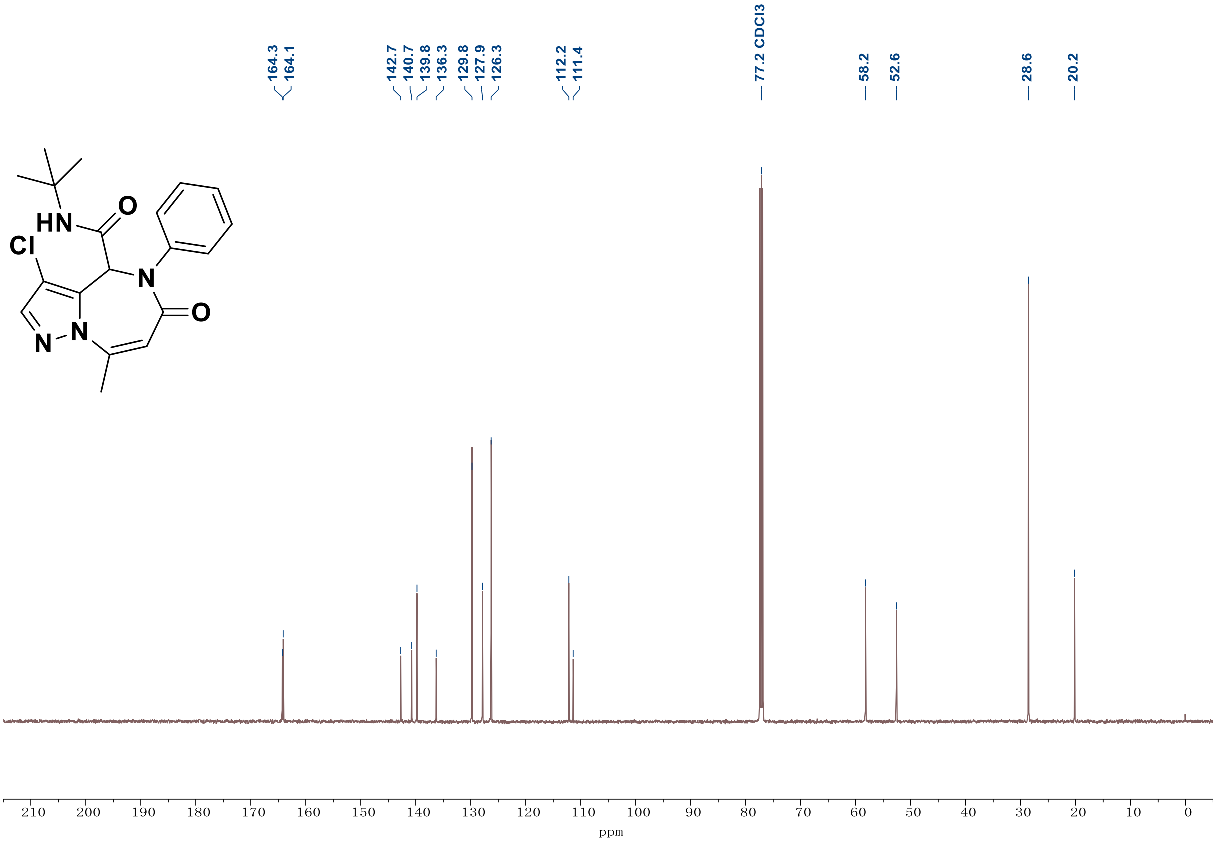

Copies of ^{1}H and $^{13}C\{^{1}H\}$ spectra of compound **16d**

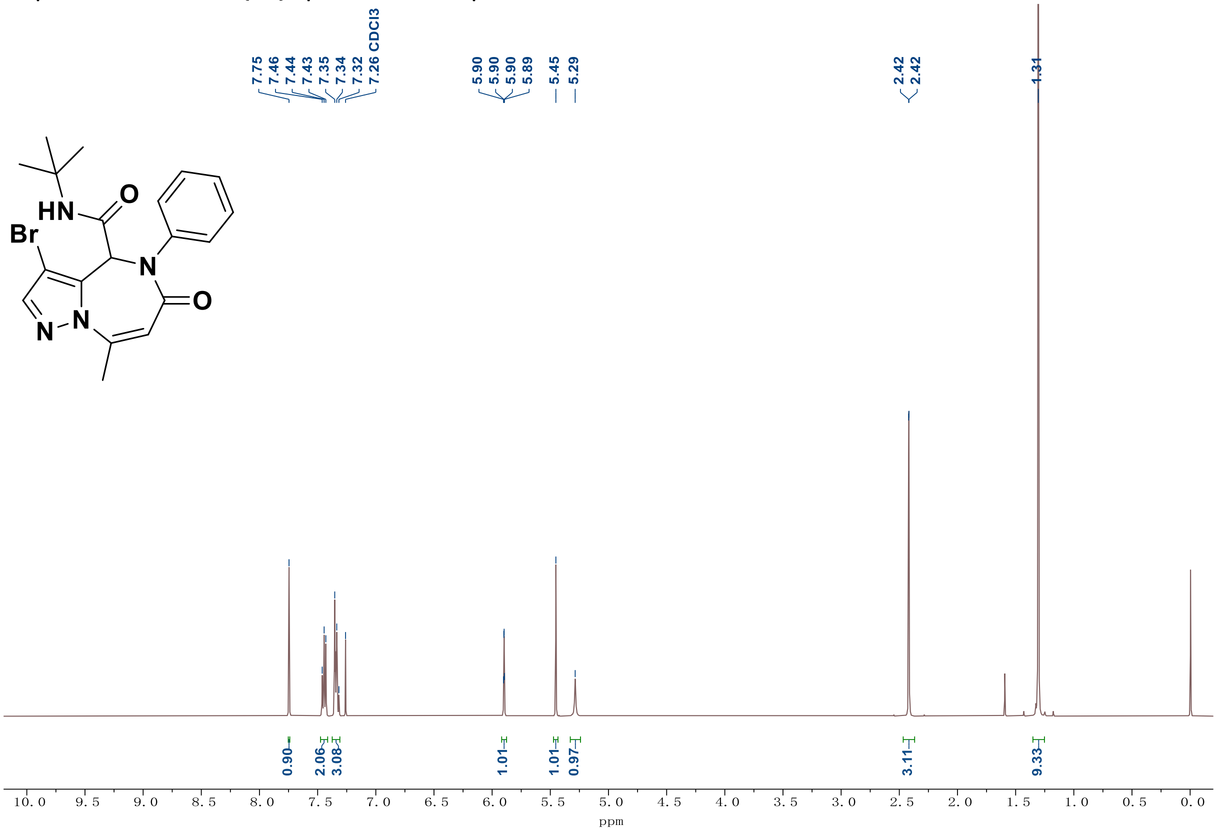

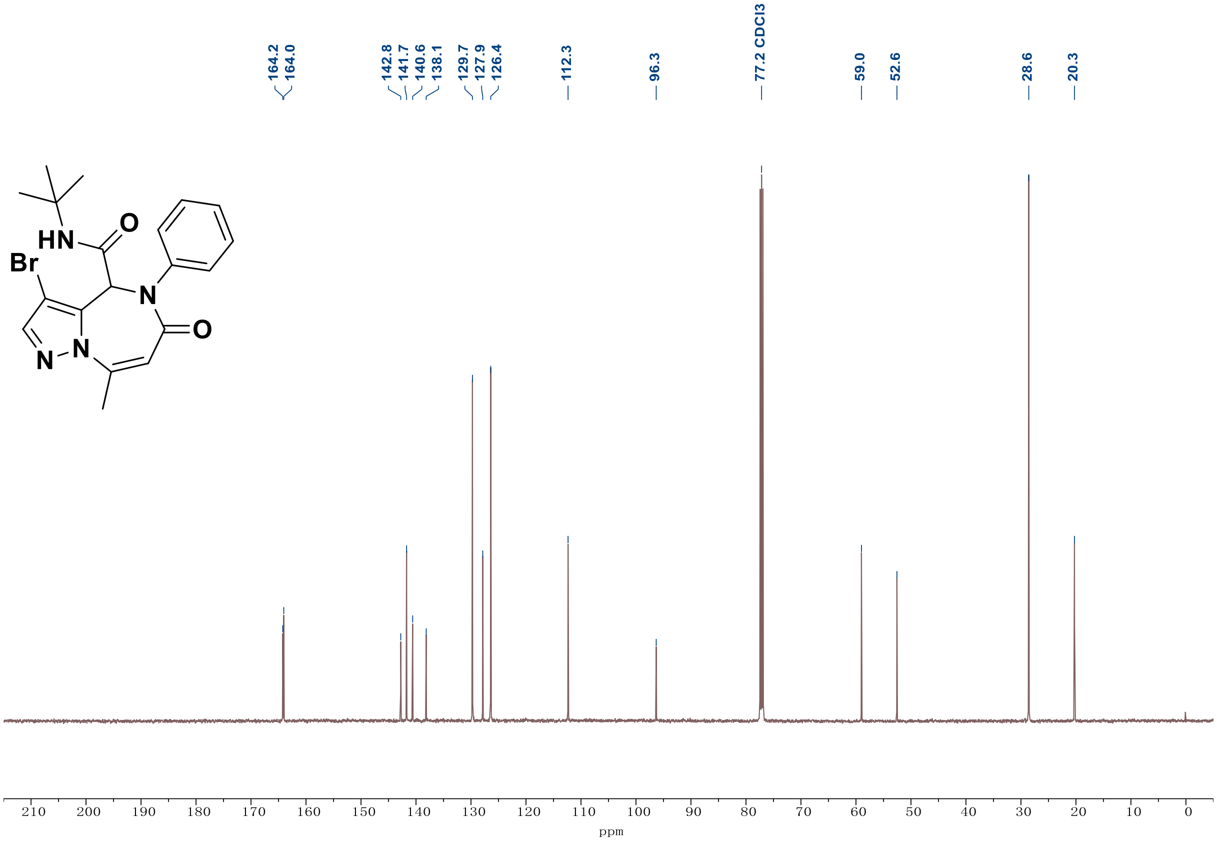

Copies of ^{1}H and $^{13}C\{^{1}H\}$ spectra of compound **16e**

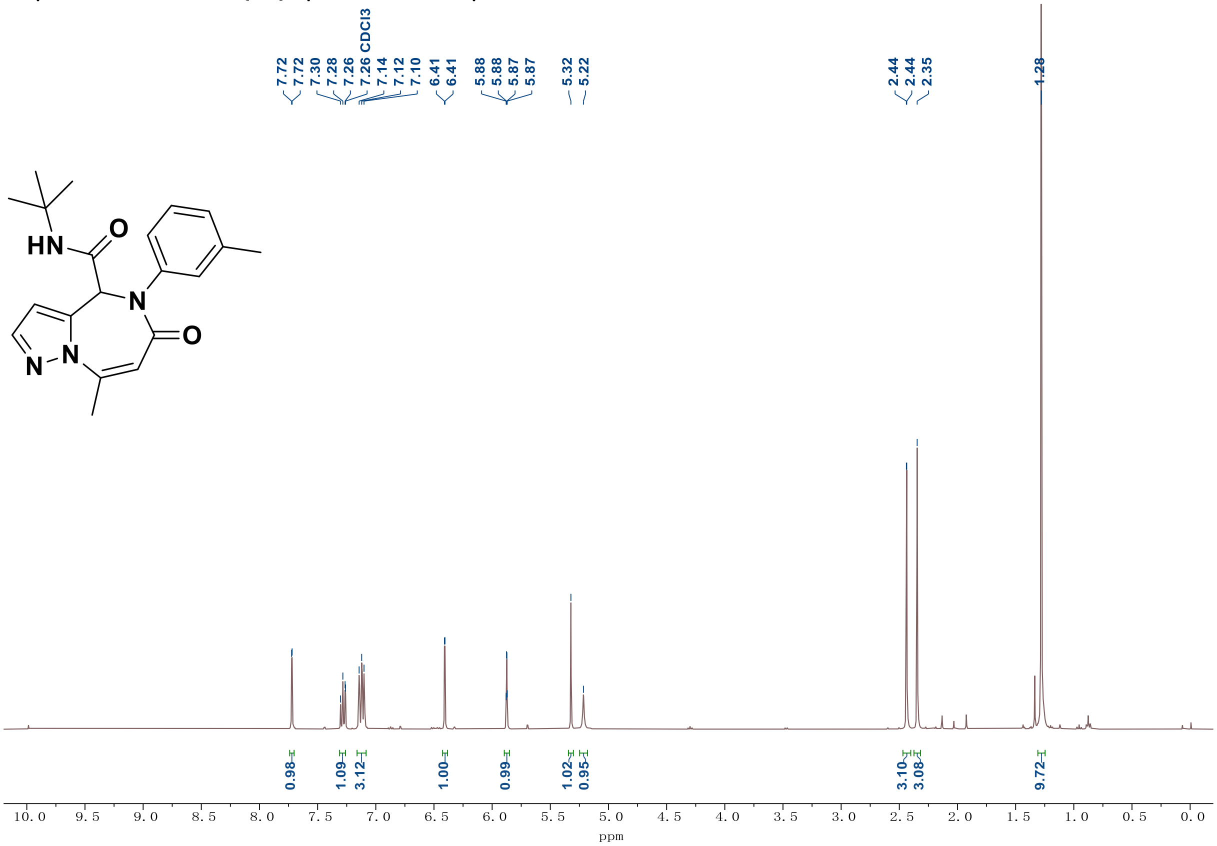

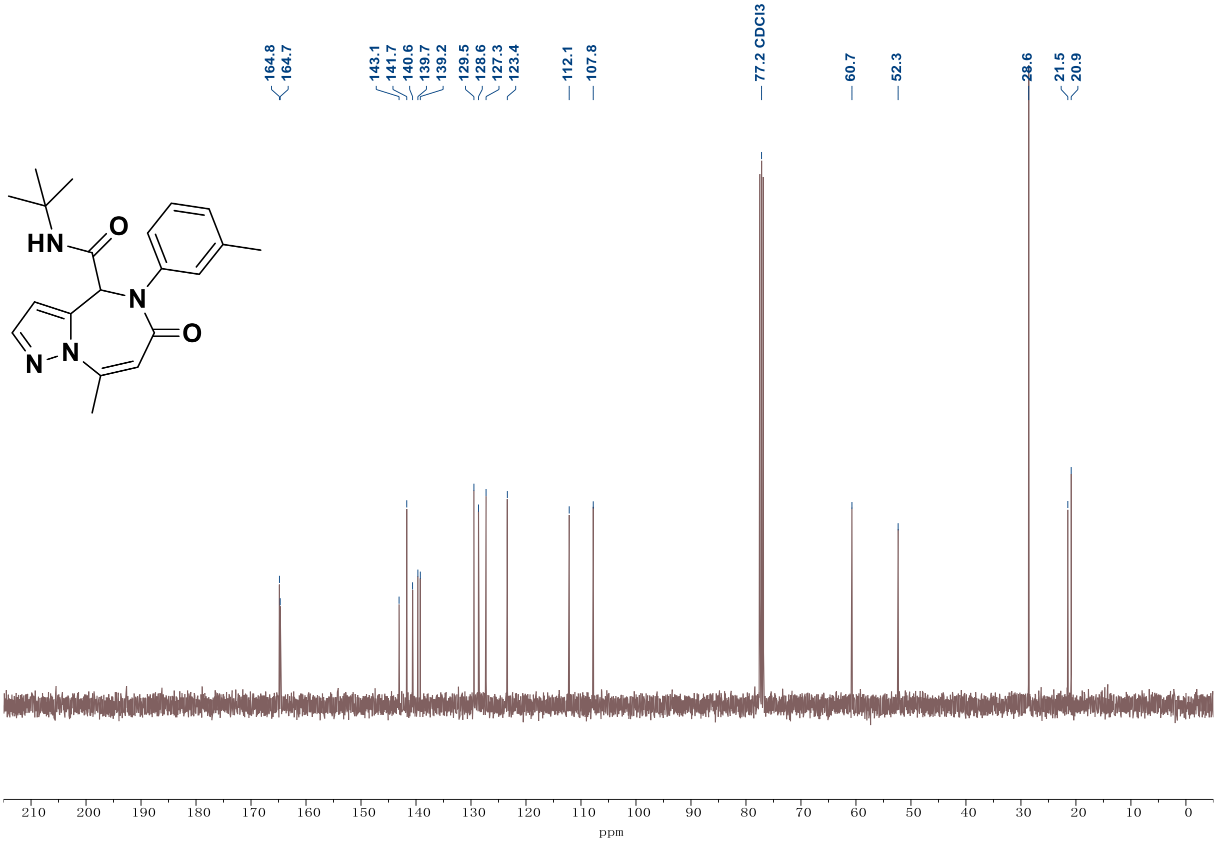

Copies of ^{1}H and $^{13}C\{^{1}H\}$ spectra of compound **16f**

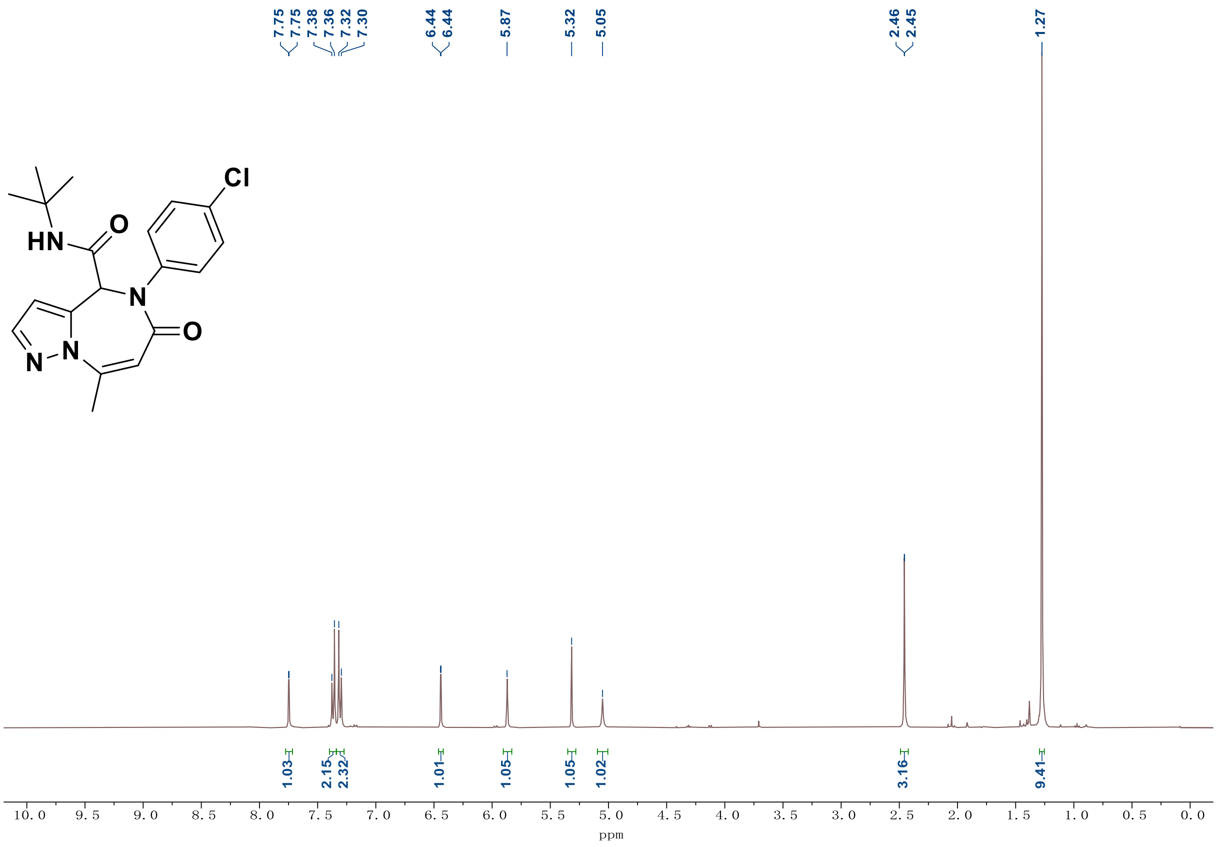

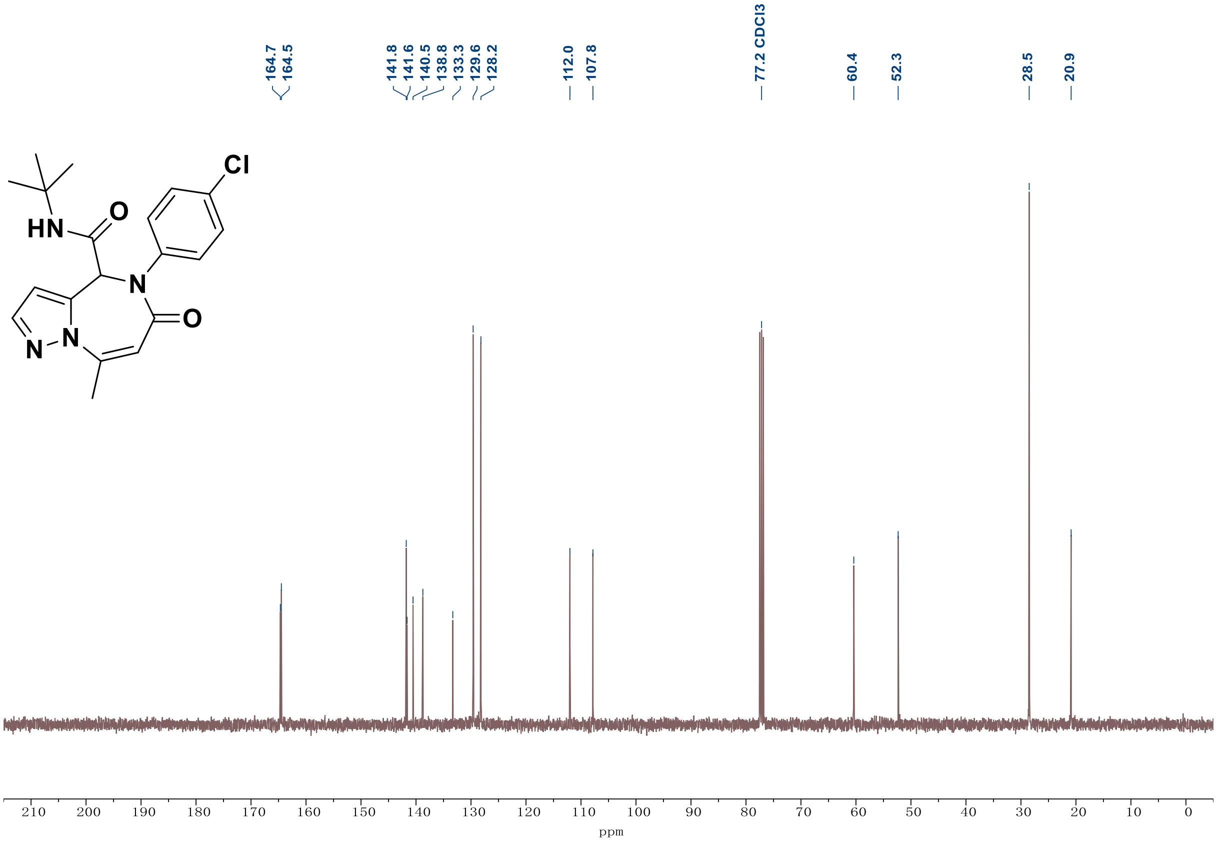

Copies of ^{1}H and $^{13}C\{^{1}H\}$ spectra of compound **16g**

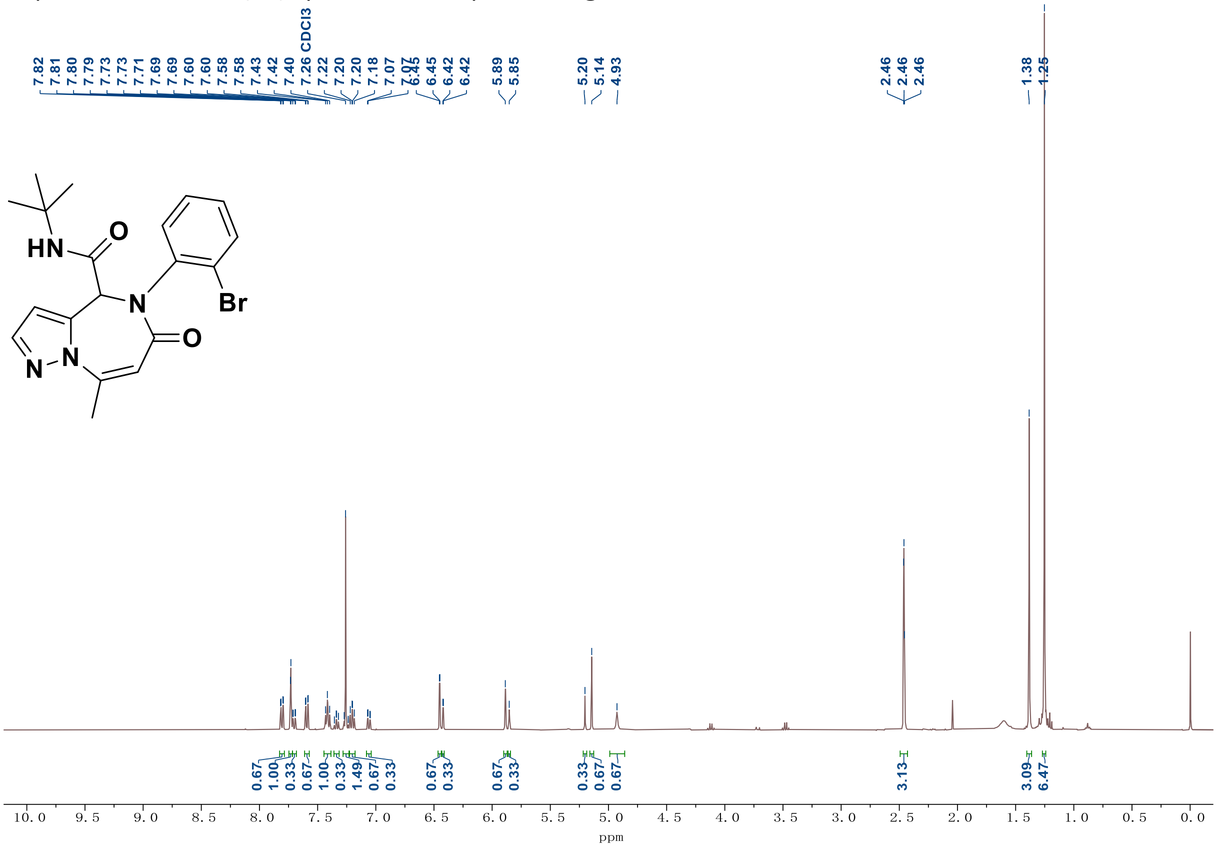

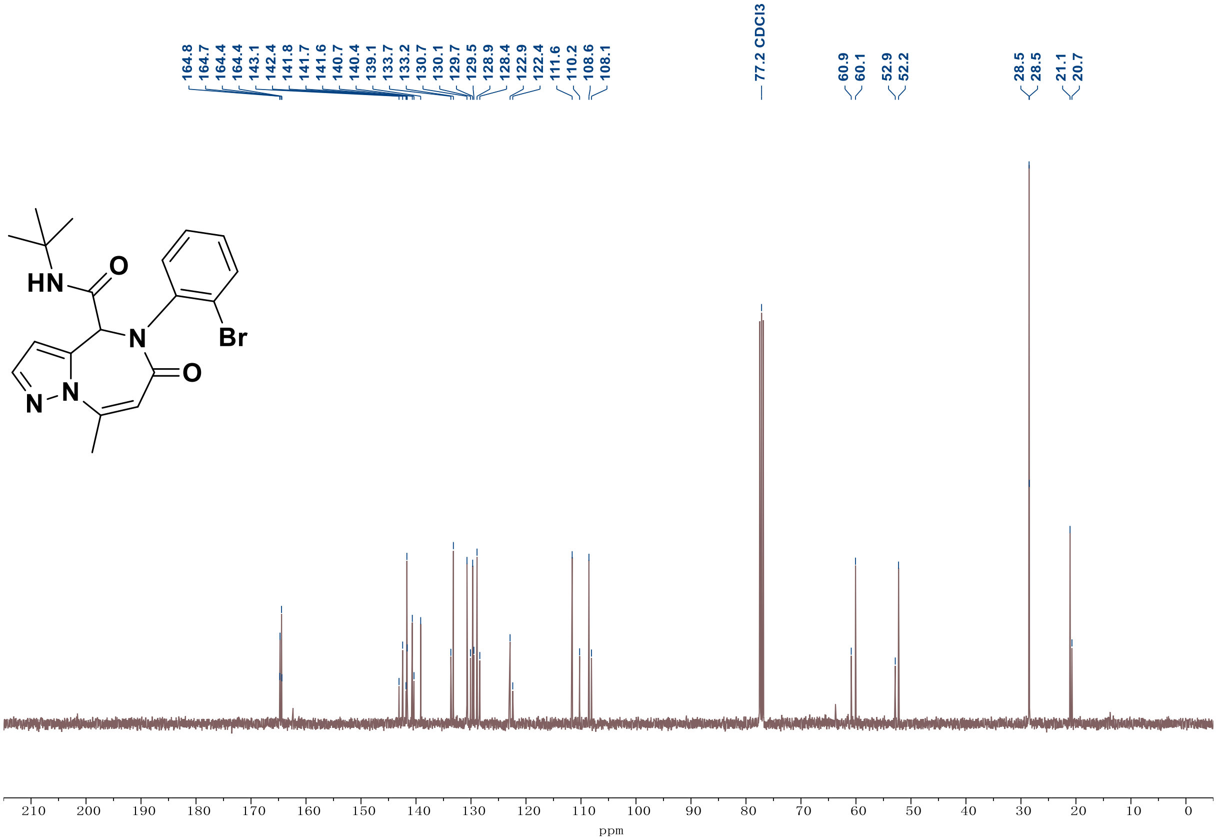

Copies of ^{1}H and $^{13}C\{^{1}H\}$ spectra of compound **16h**

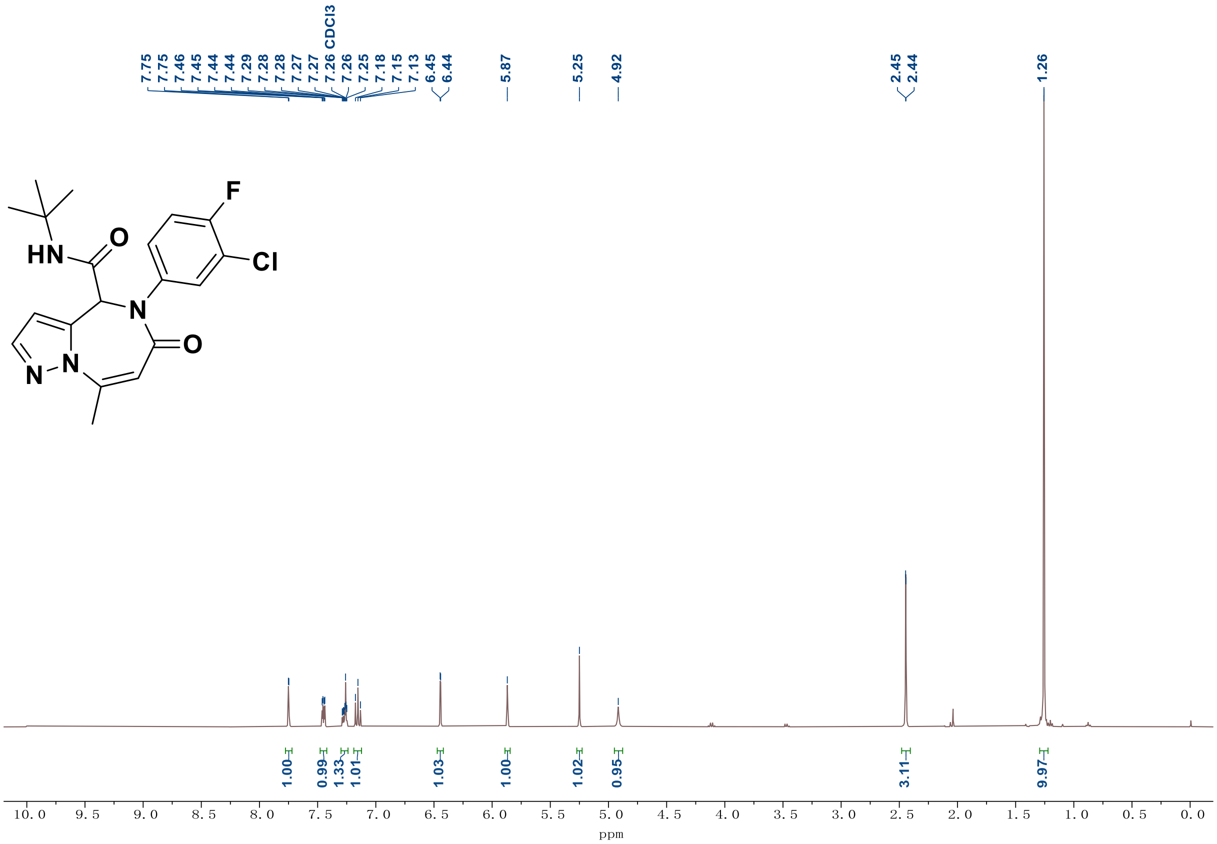

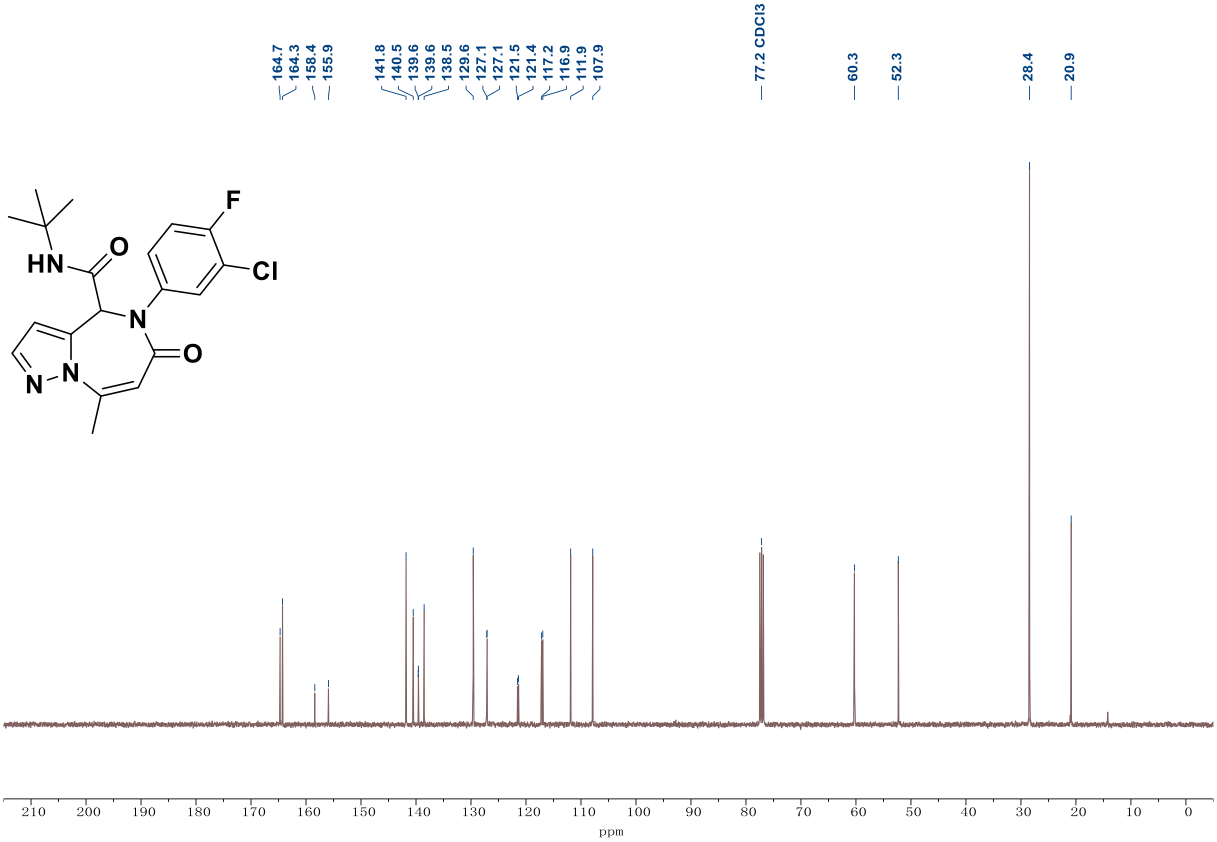

Copies of ^{1}H and $^{13}C\{^{1}H\}$ spectra of compound **16i**

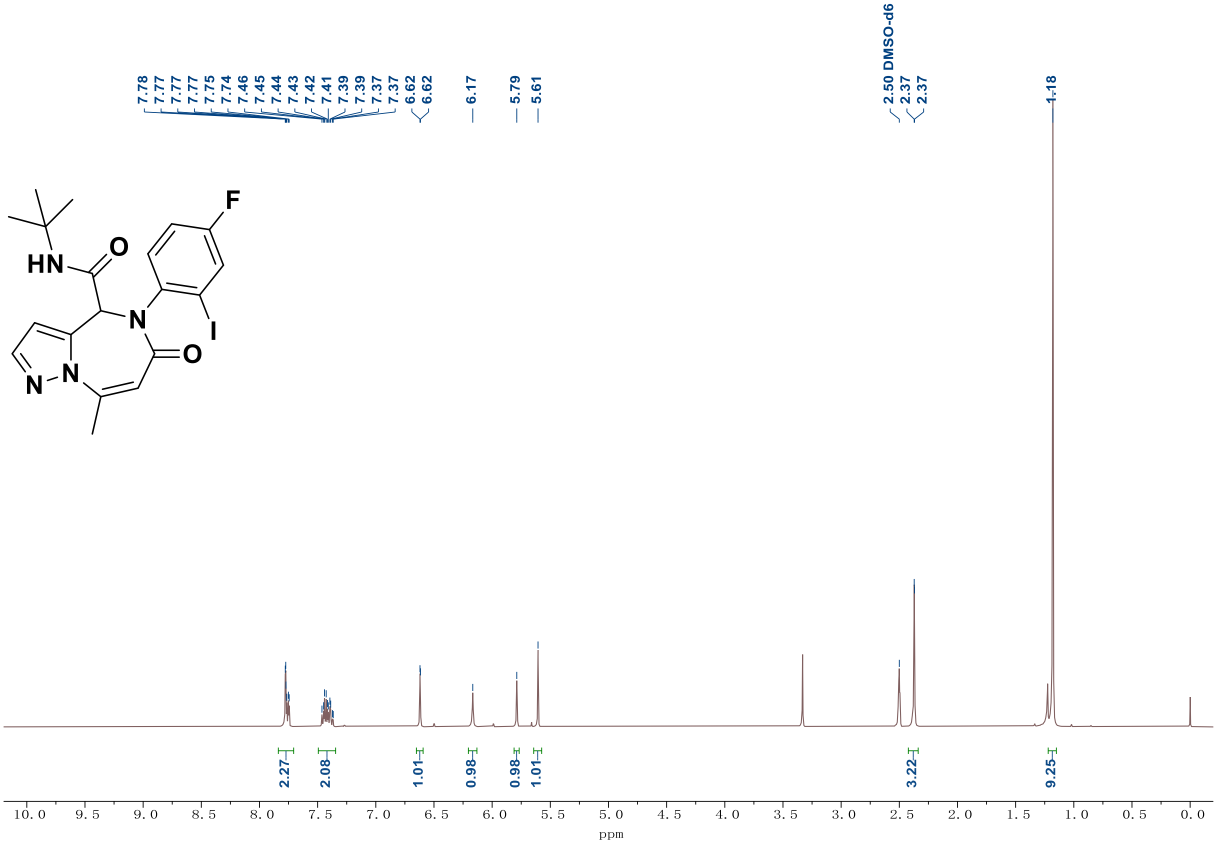

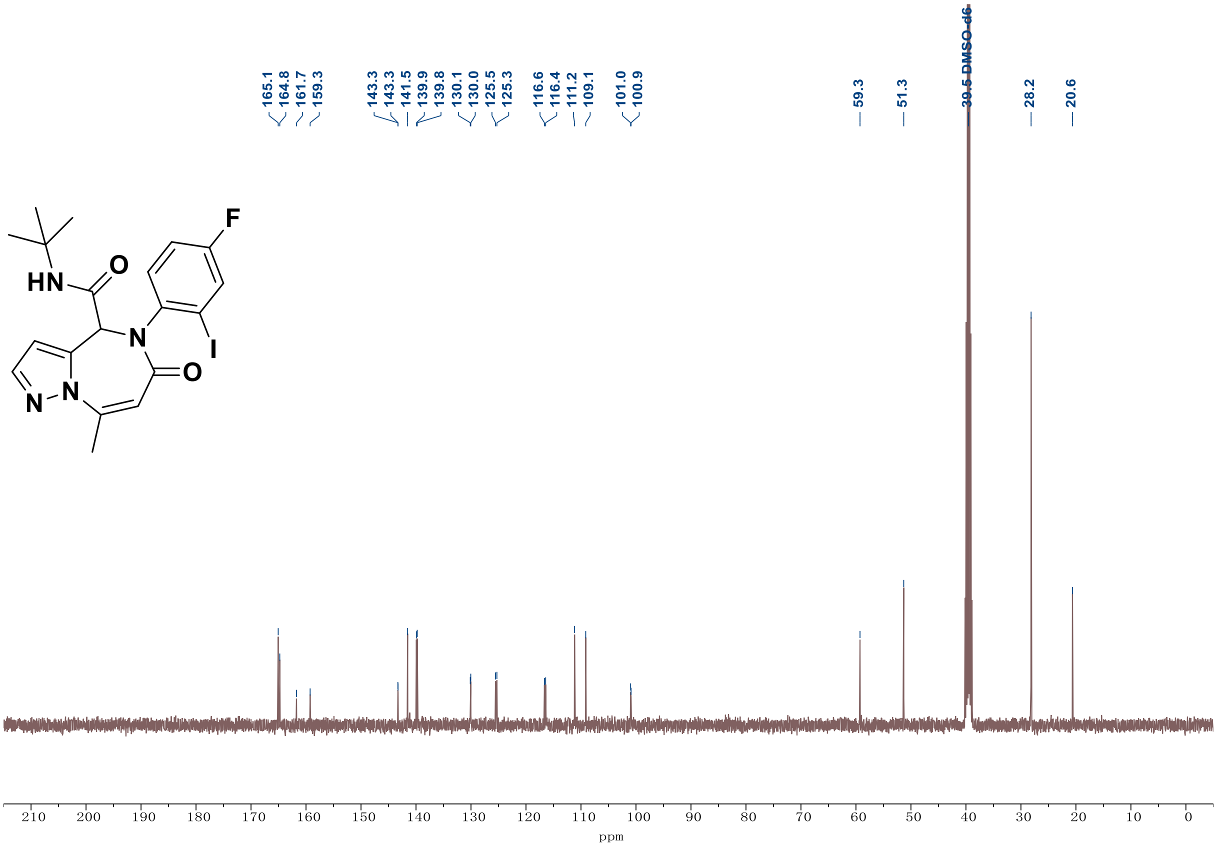

Copies of ^{1}H and ^{13}C{^{1}H} spectra of compound **16j**

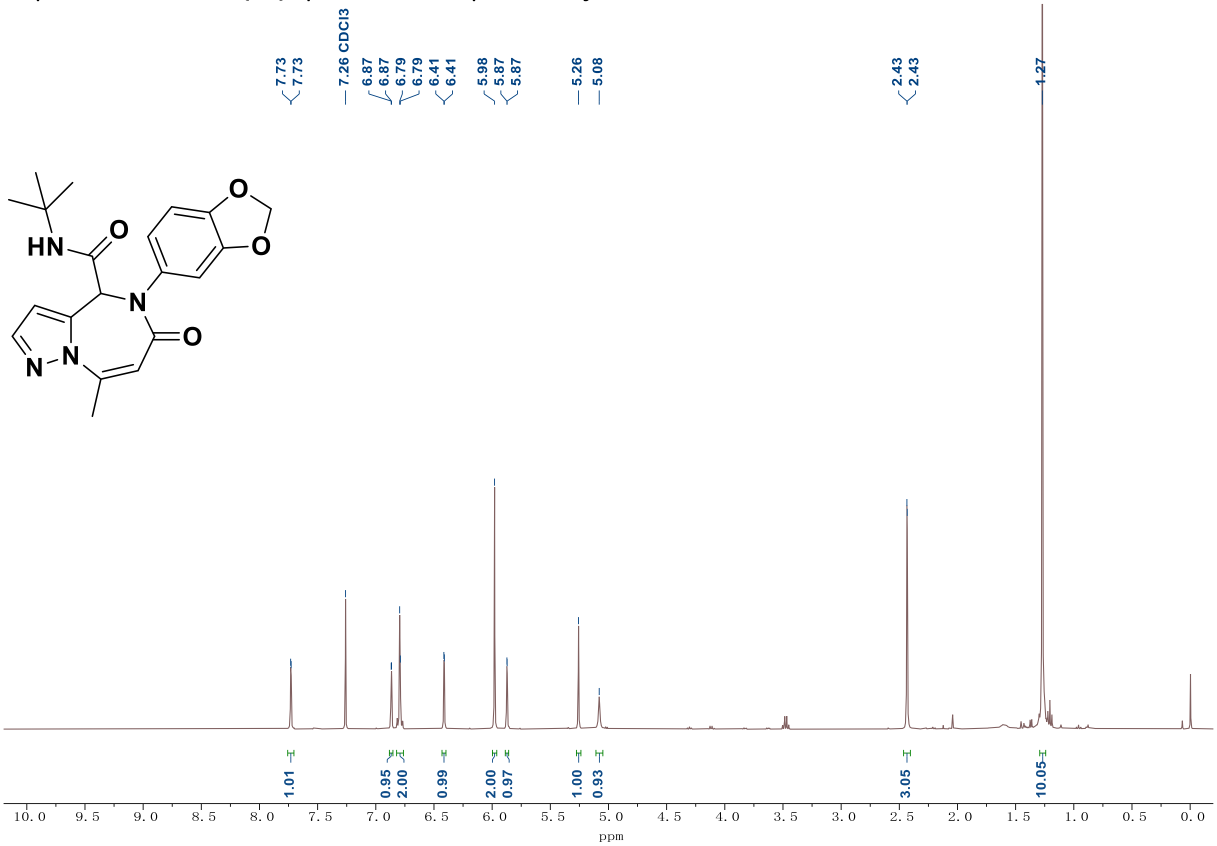

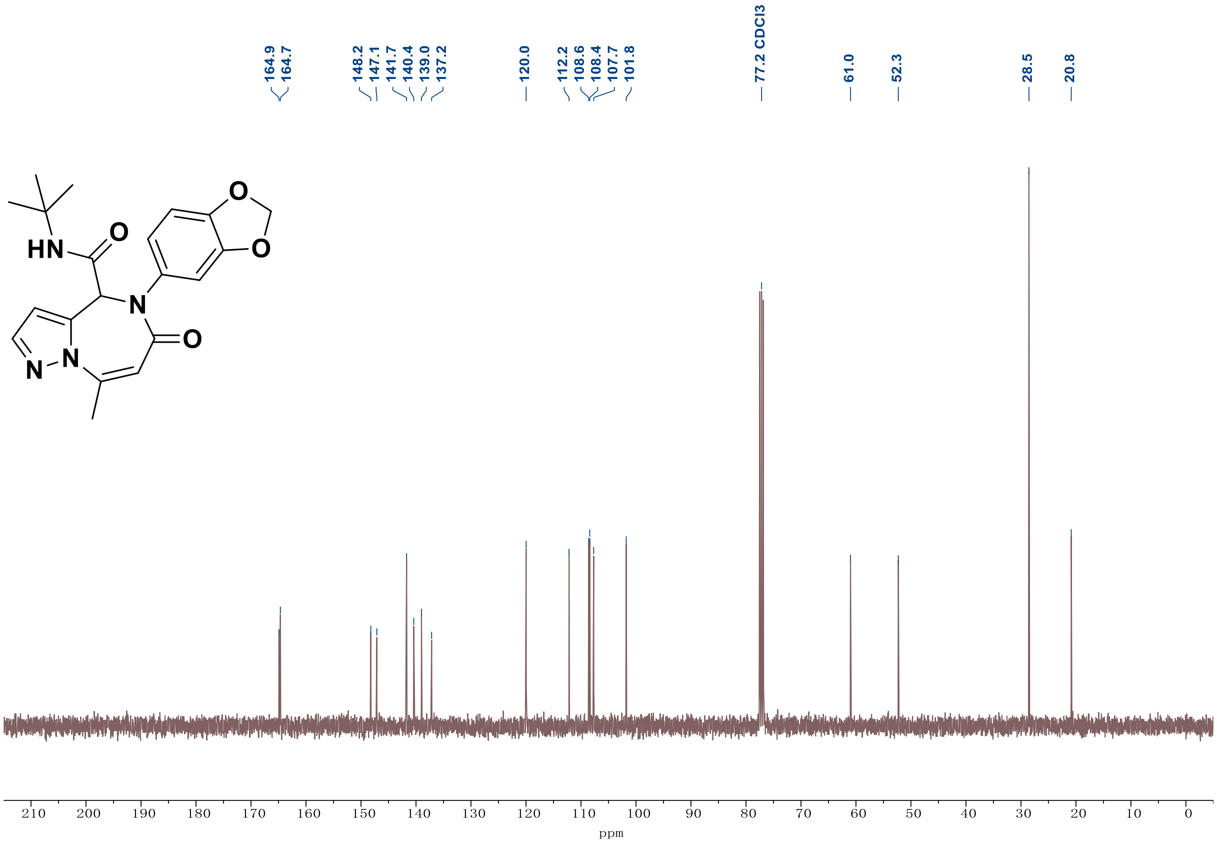

Copies of ^{1}H and $^{13}C\{^{1}H\}$ spectra of compound **16k**

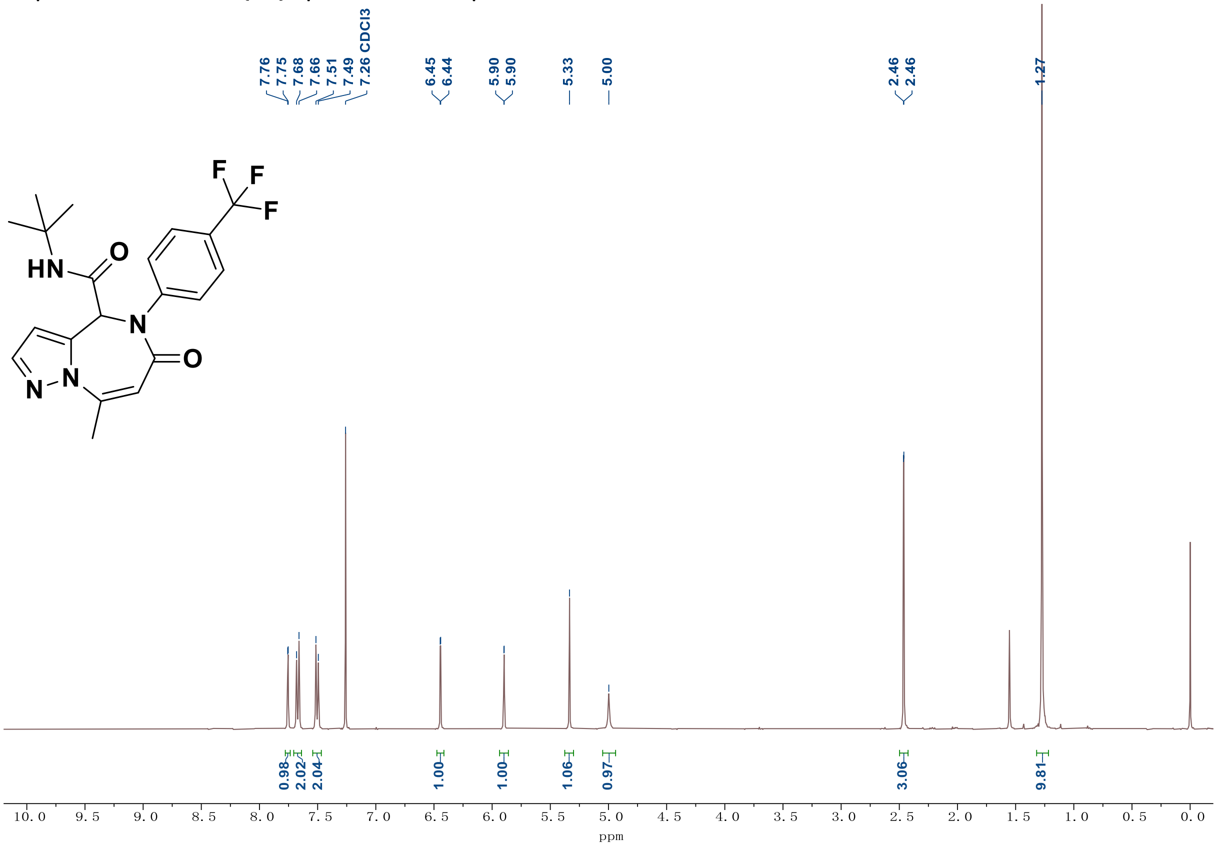

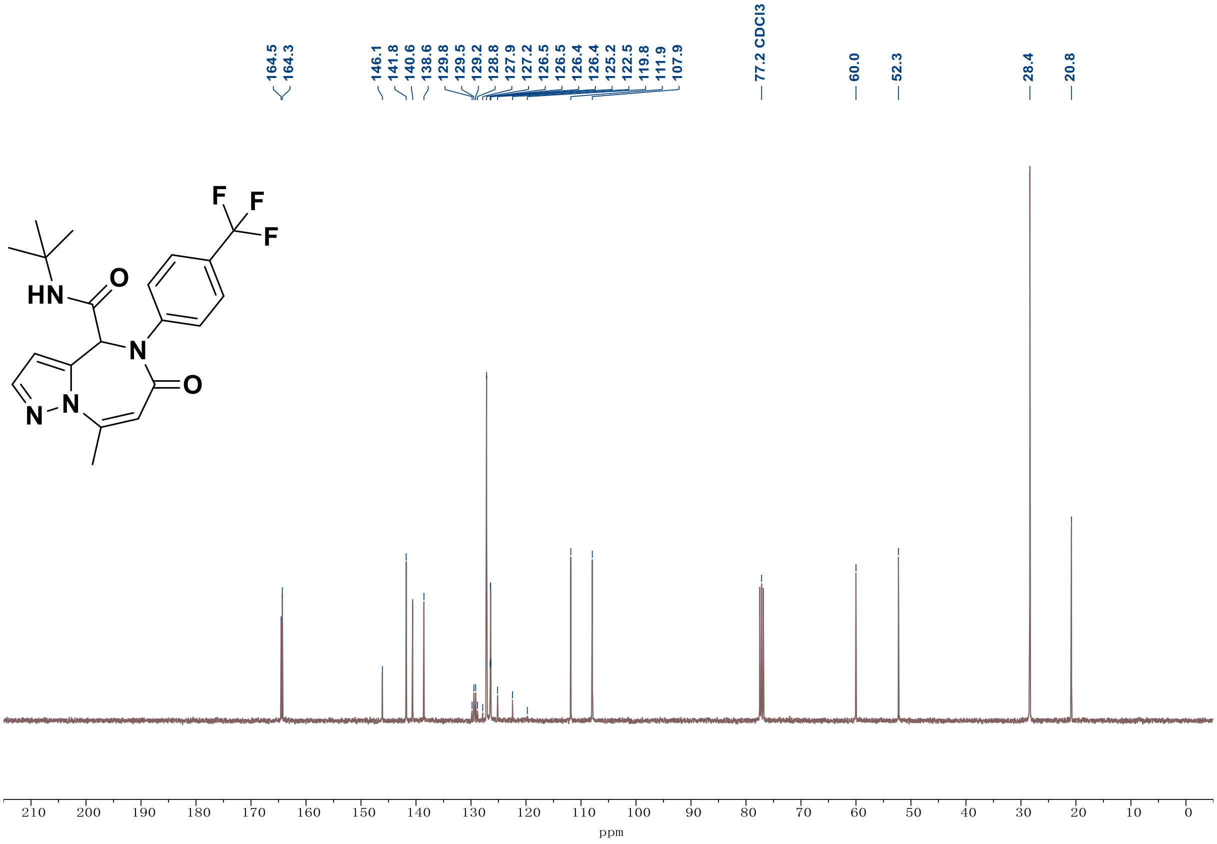

Copies of ^{1}H and ^{13}C{^{1}H} spectra of compound **16l**

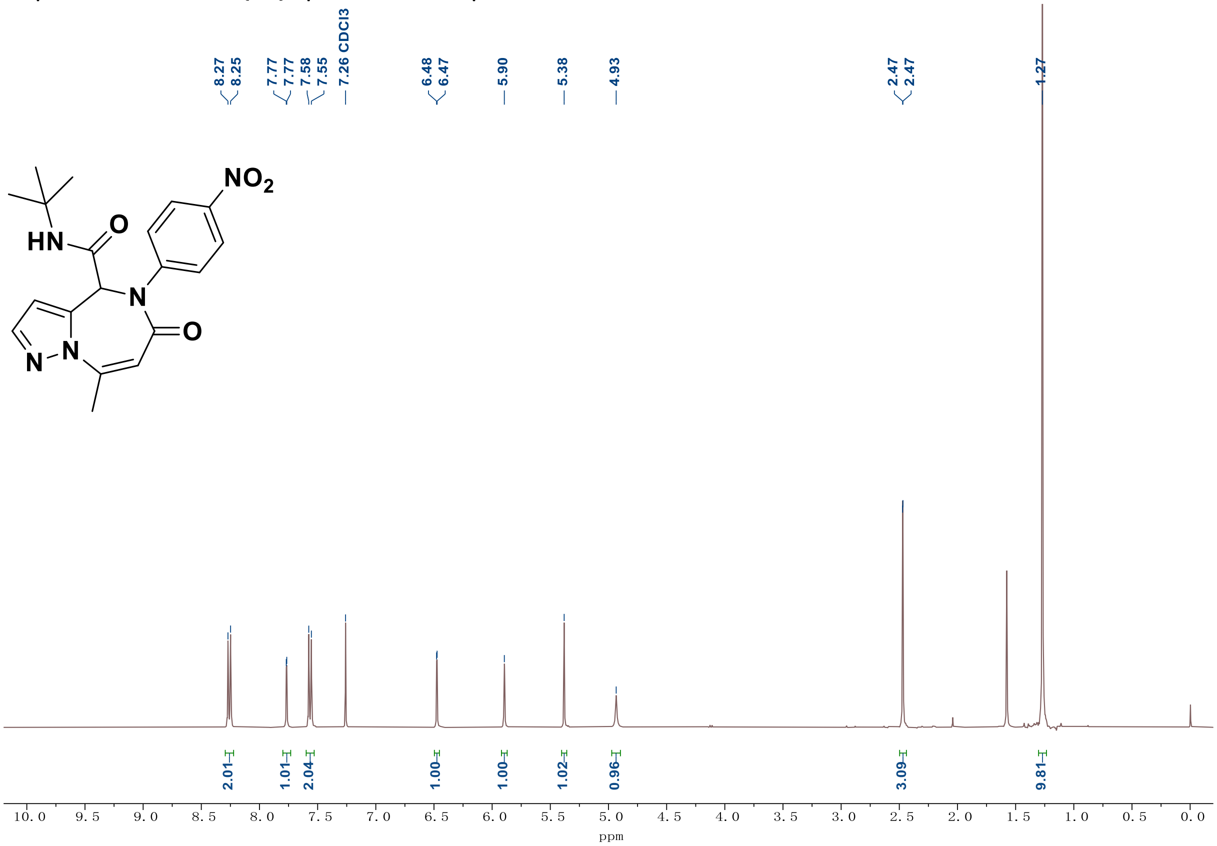

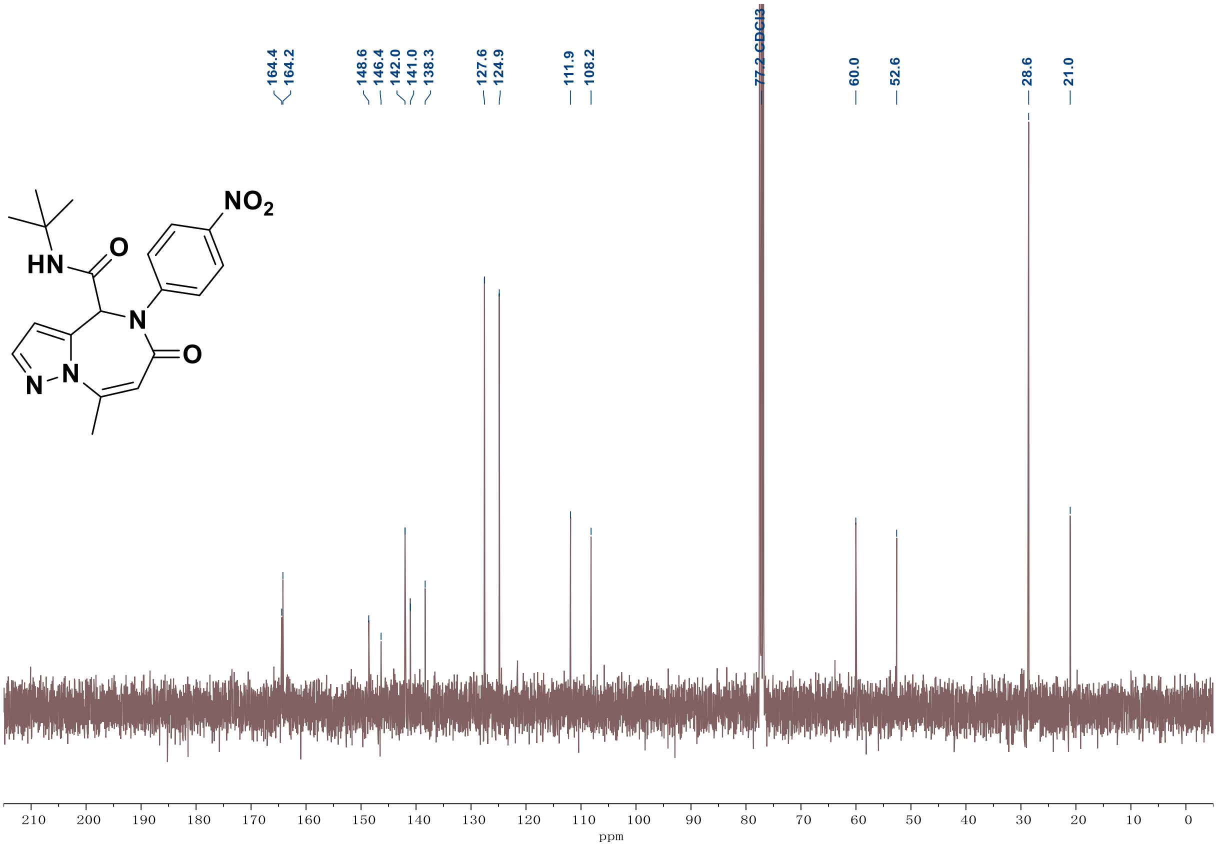

Copies of ^{1}H and $^{13}C\{^{1}H\}$ spectra of compound **16m**

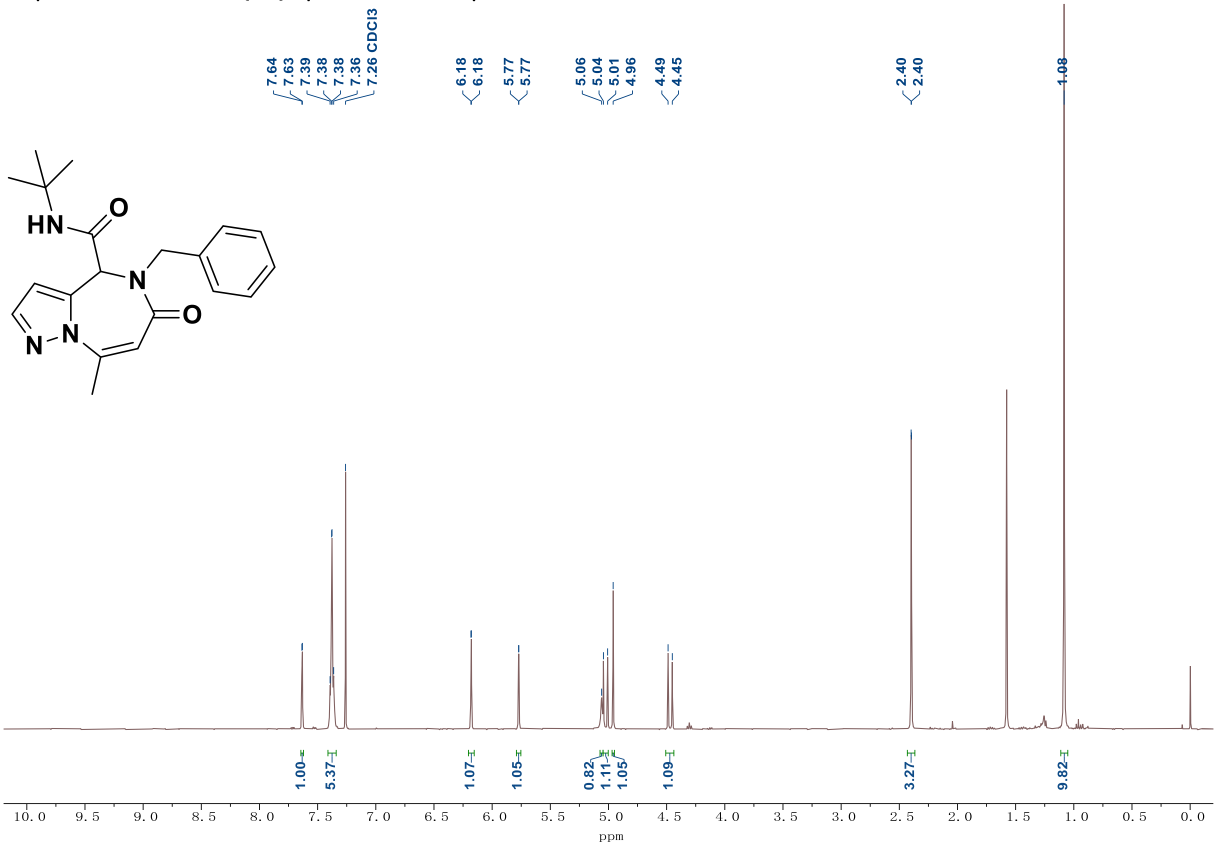

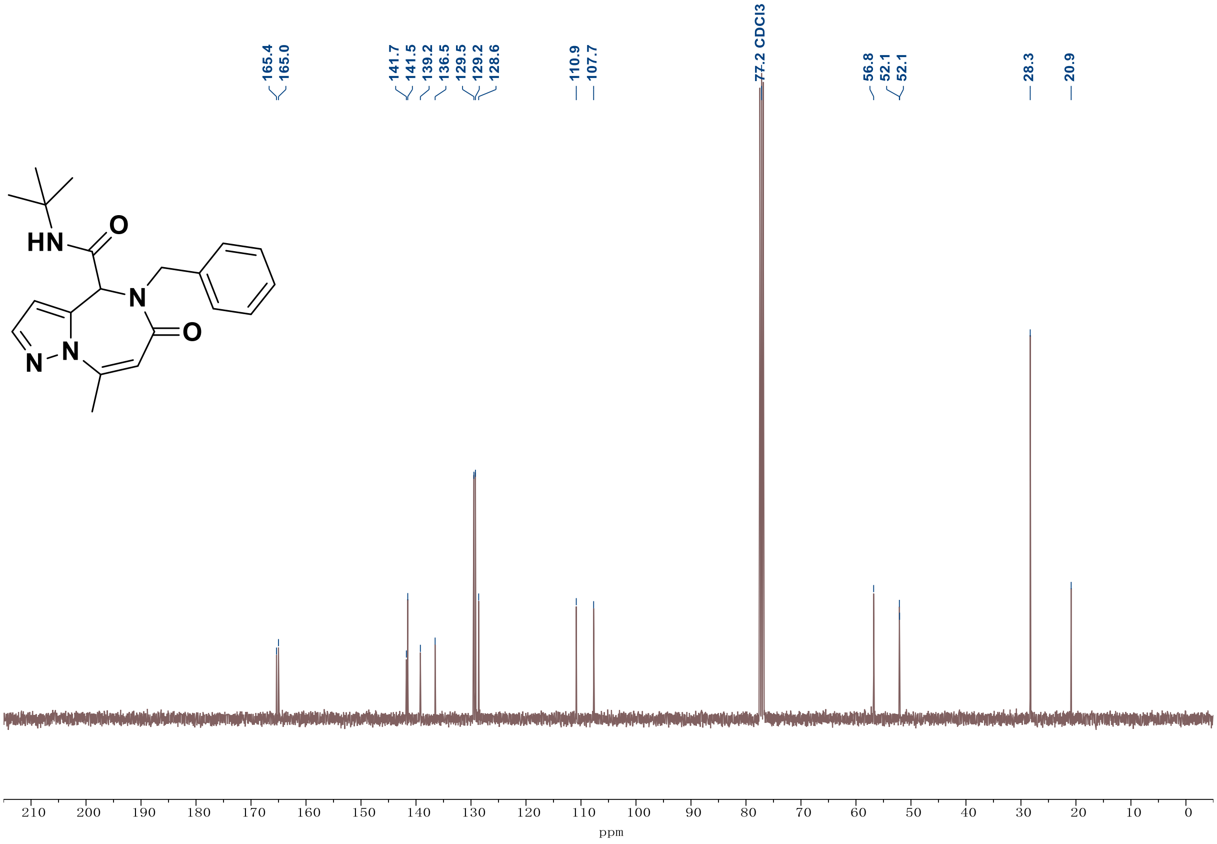

Copies of ^{1}H and $^{13}C\{^{1}H\}$ spectra of compound **16n**

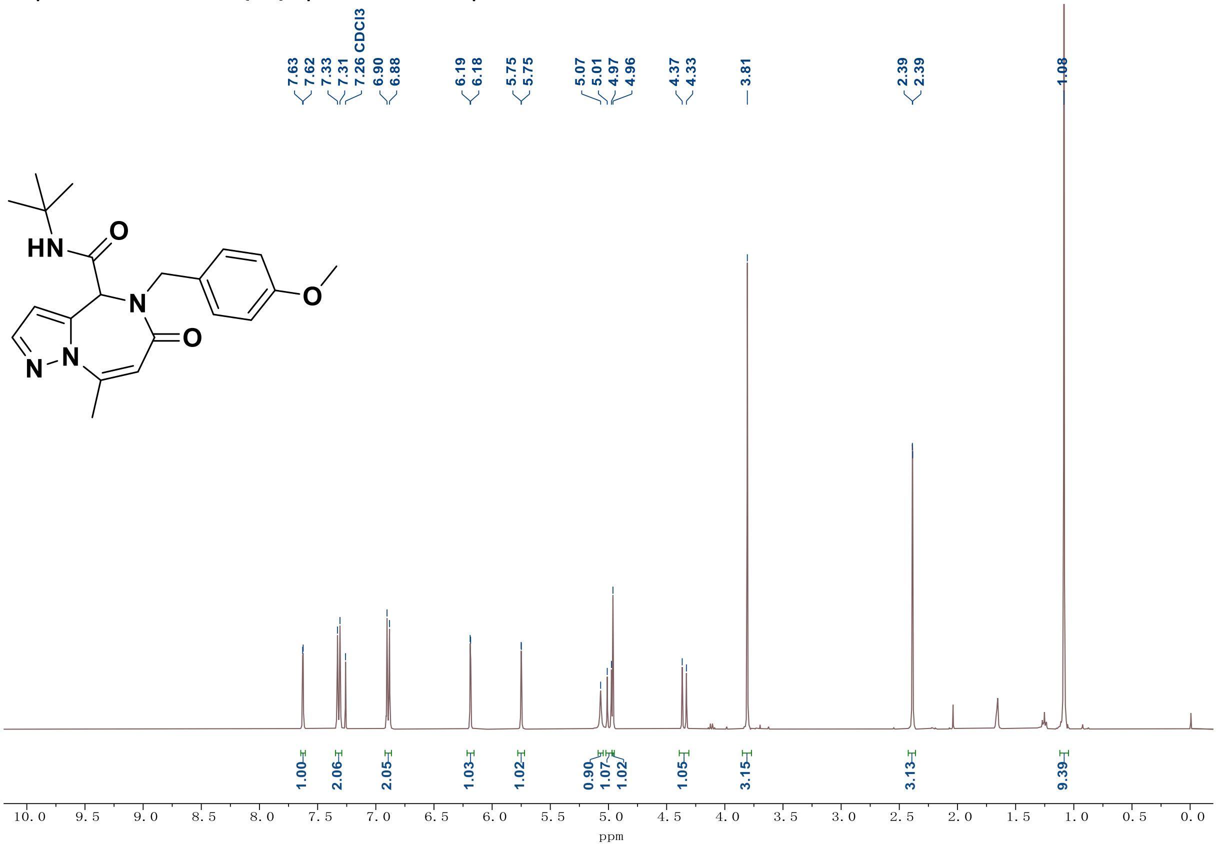

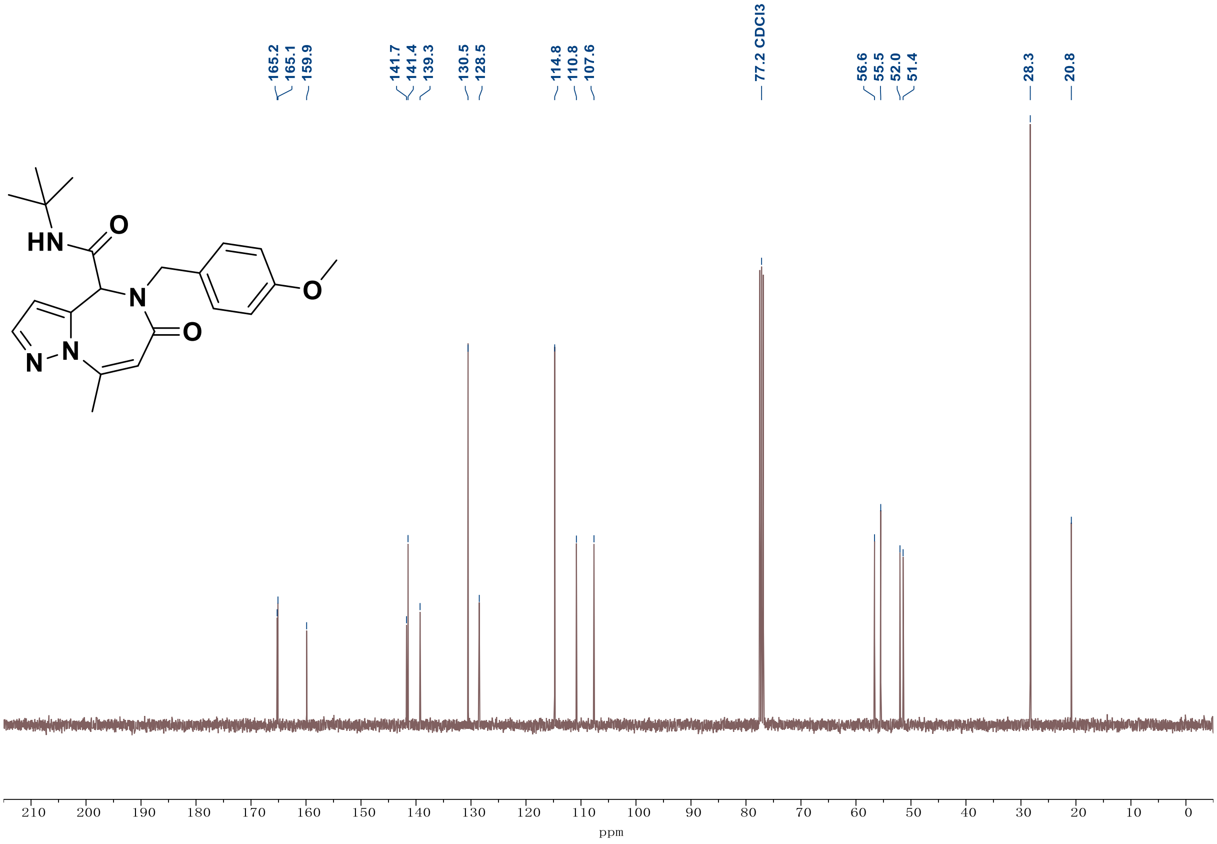

Copies of ^{1}H and $^{13}C\{^{1}H\}$ spectra of compound **16o**

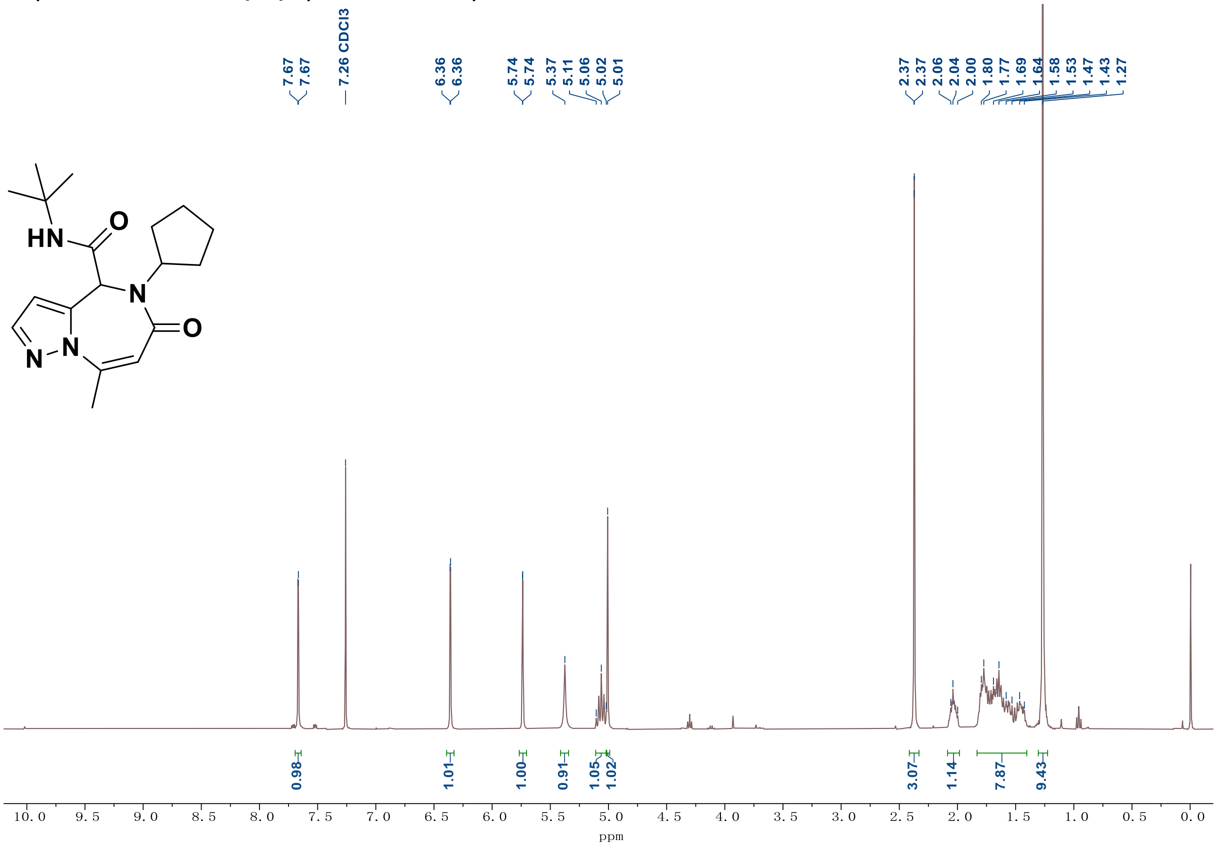

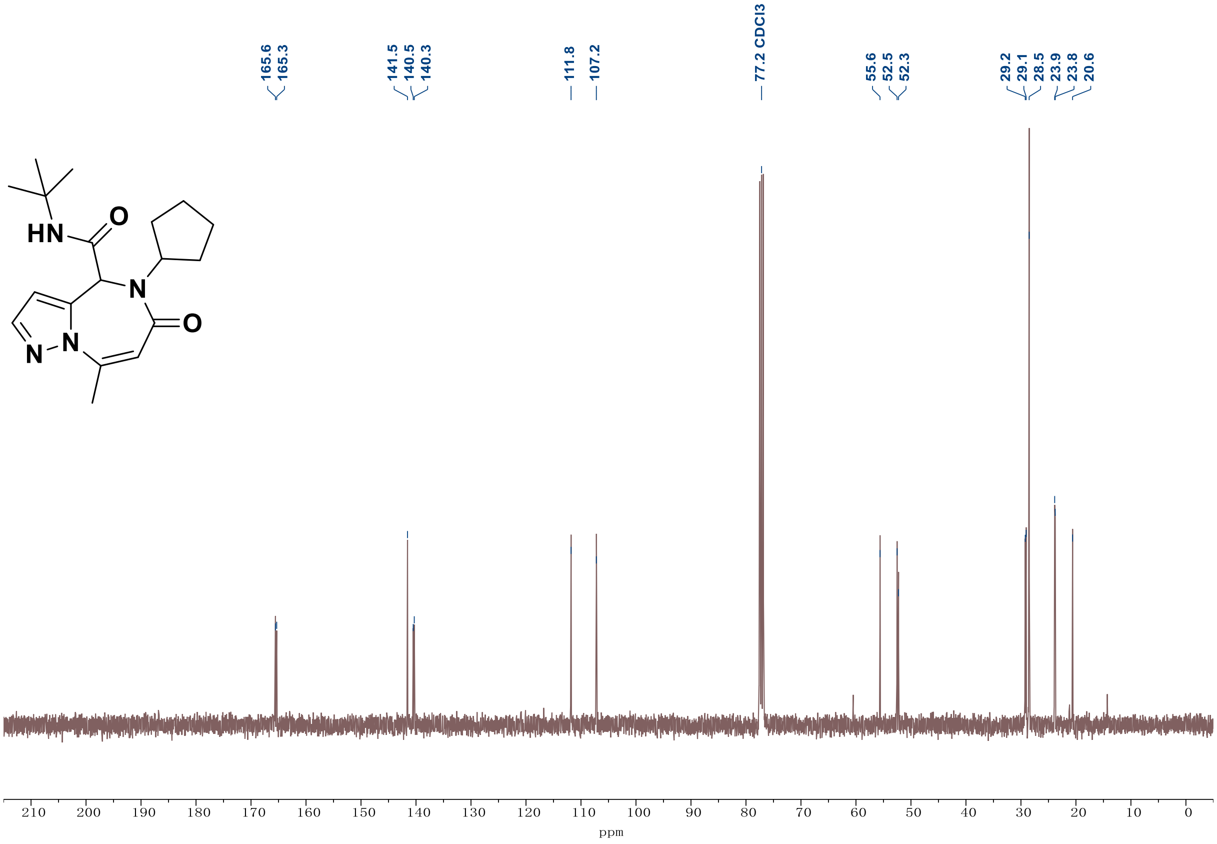

Copies of ^{1}H and $^{13}C\{^{1}H\}$ spectra of compound **16p**

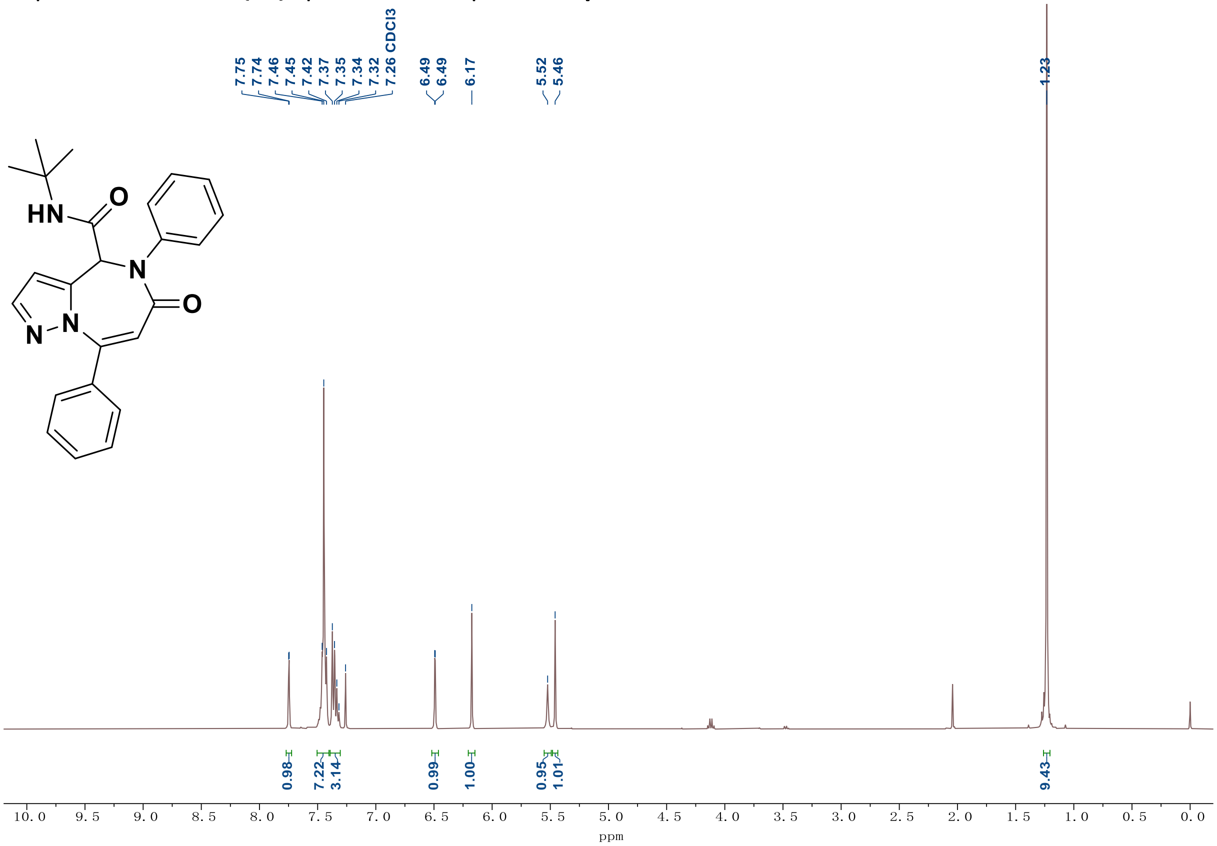

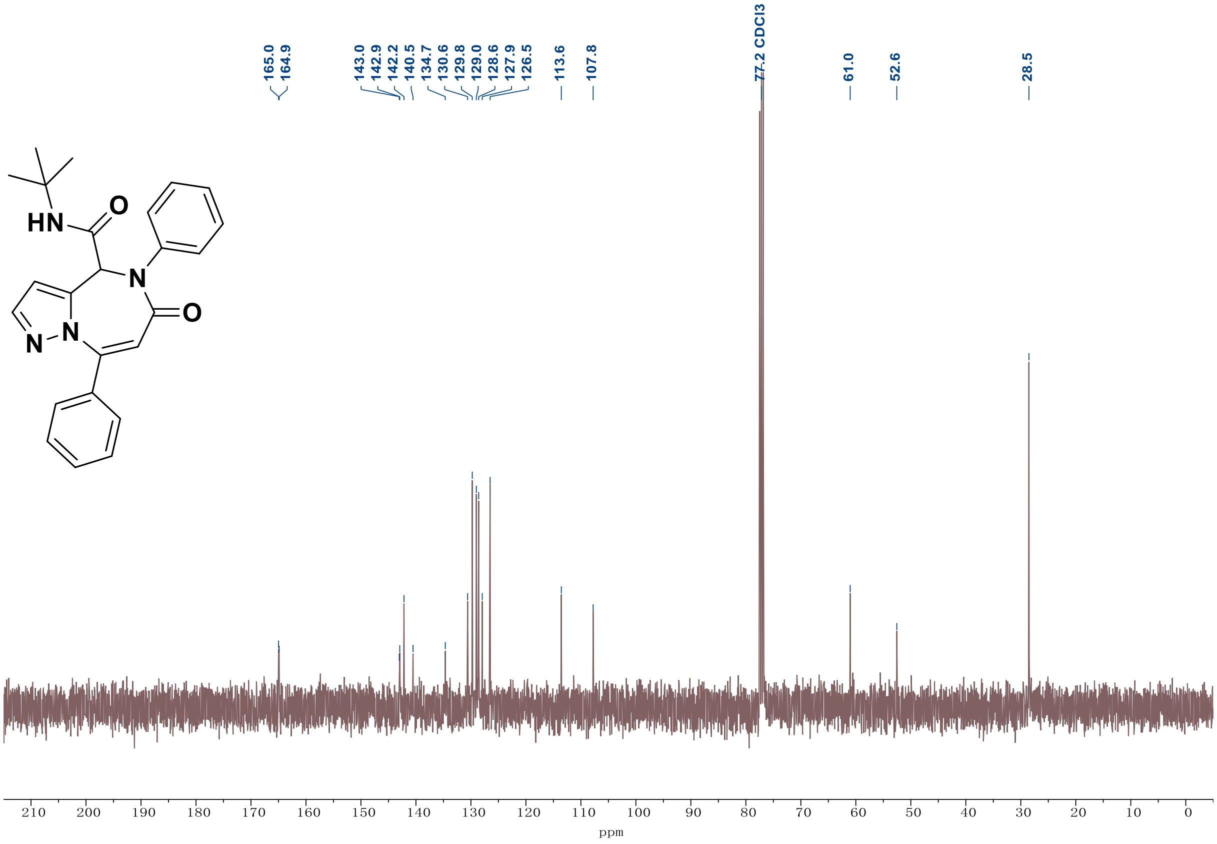

Copies of ^{1}H and $^{13}C\{^{1}H\}$ spectra of compound **16q**

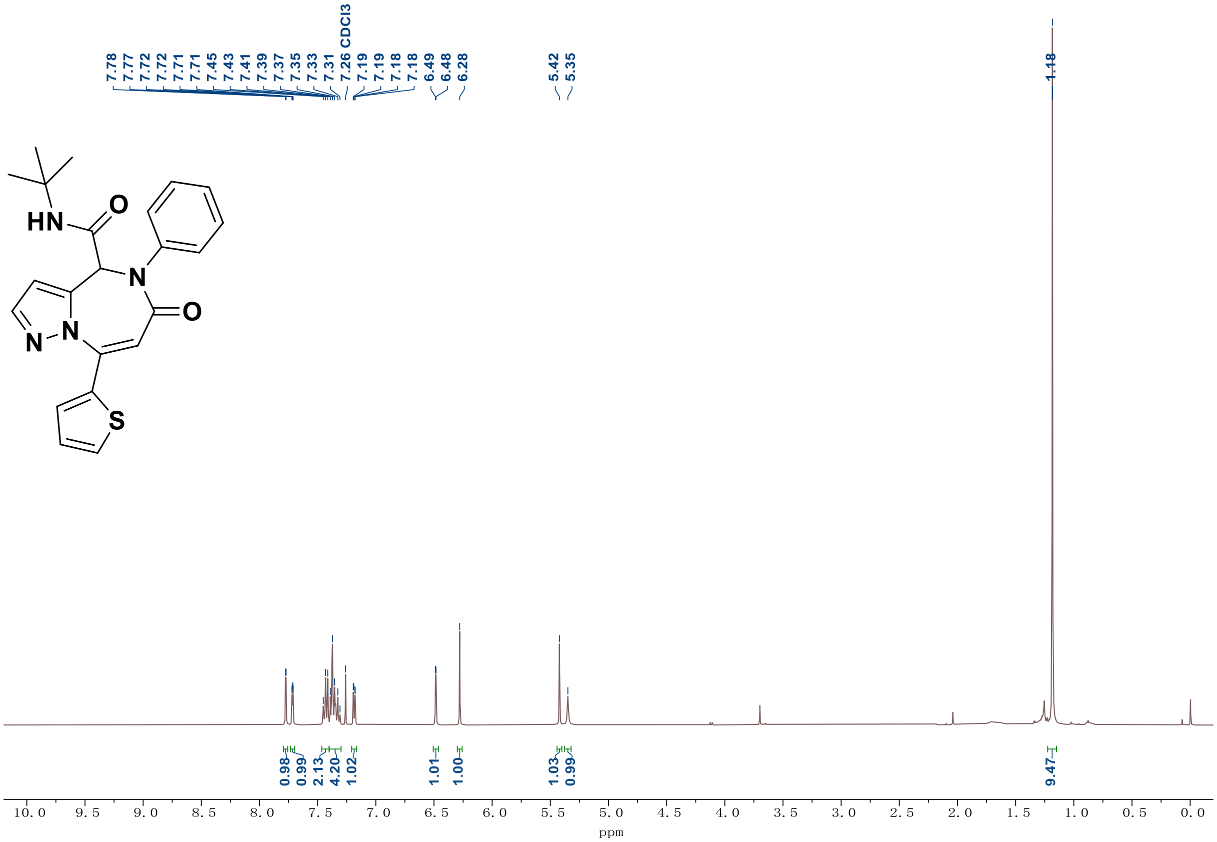

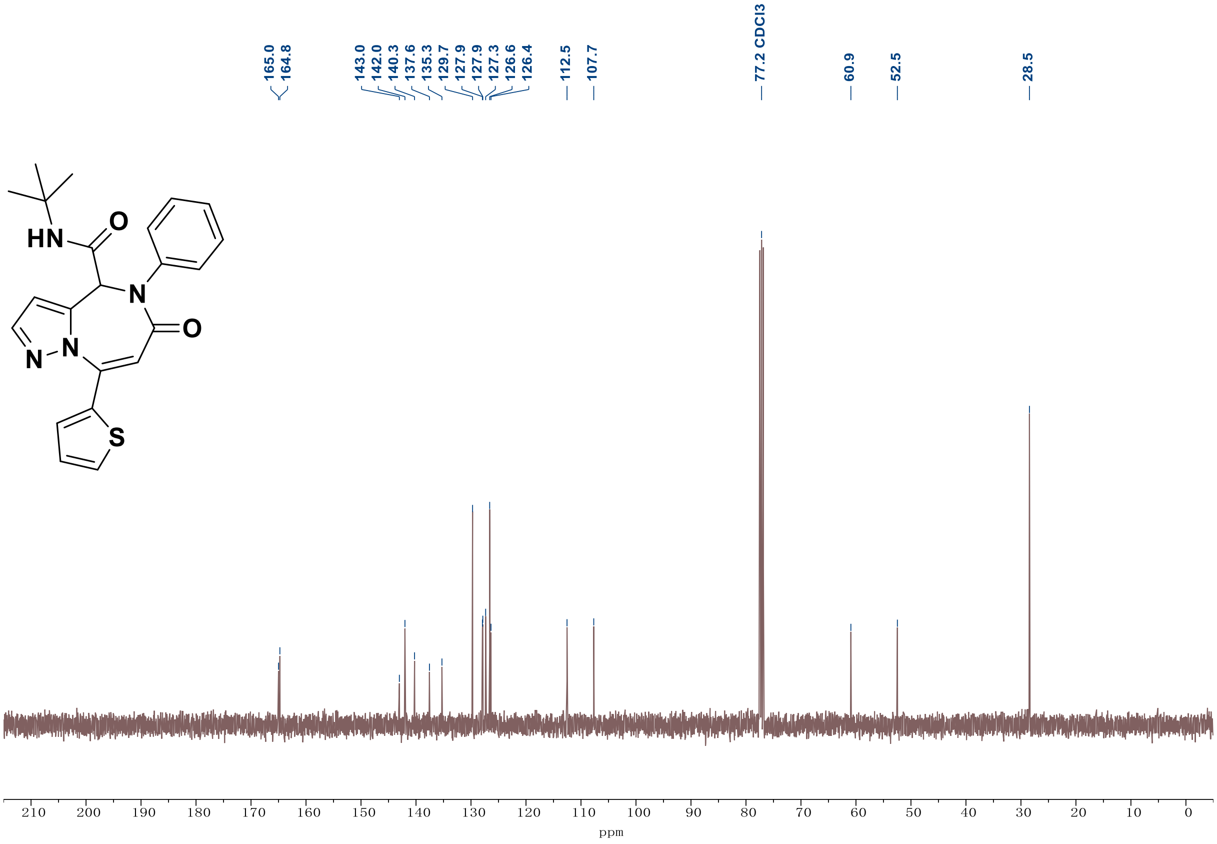

Copies of ^{1}H and $^{13}C\{^{1}H\}$ spectra of compound **16r**

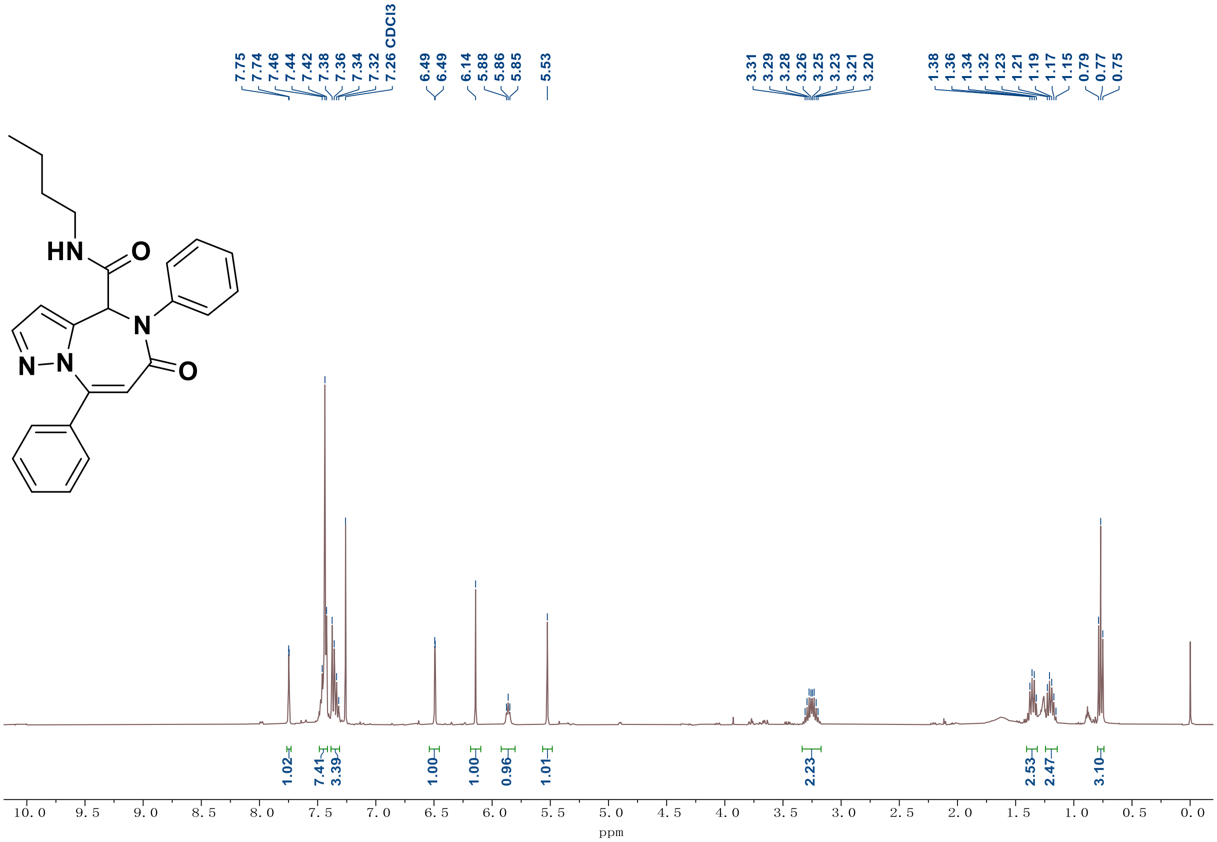

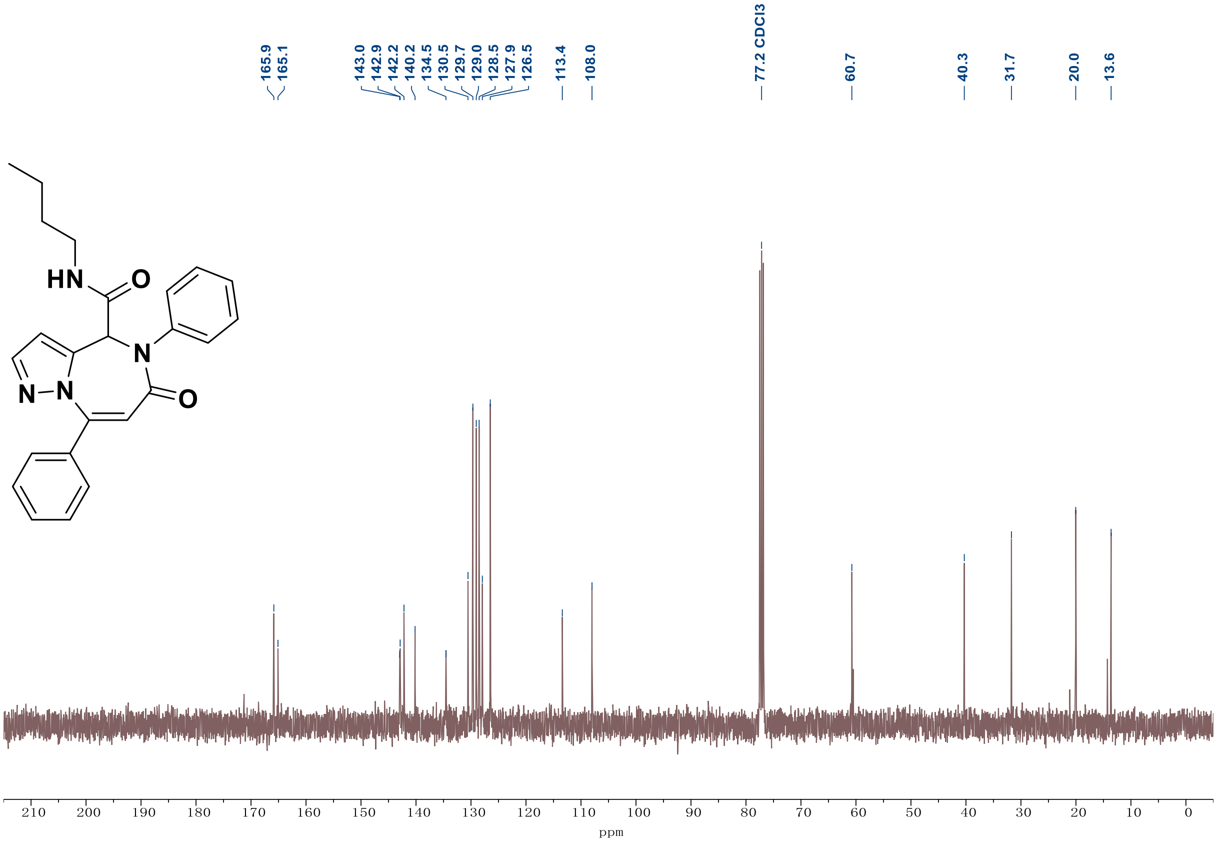

Copies of ^{1}H and $^{13}C\{^{1}H\}$ spectra of compound **16s**

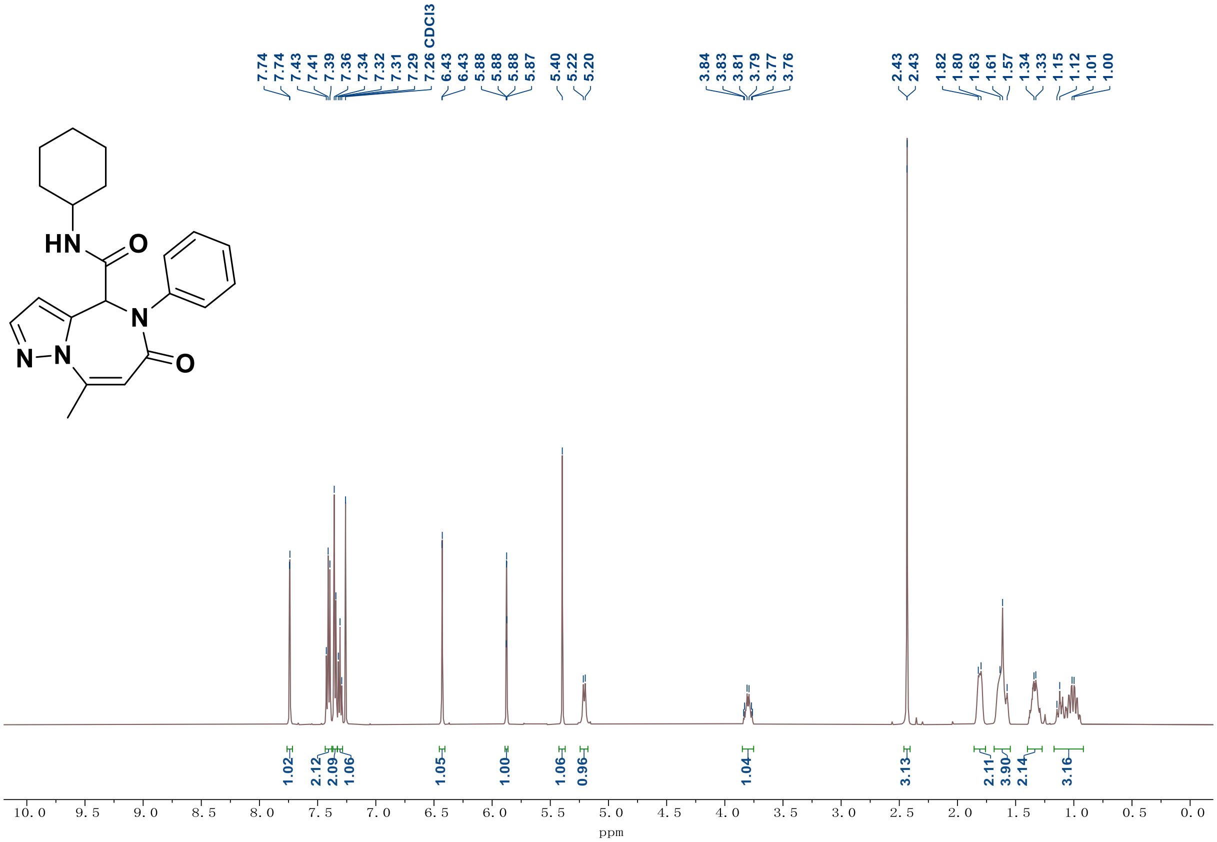

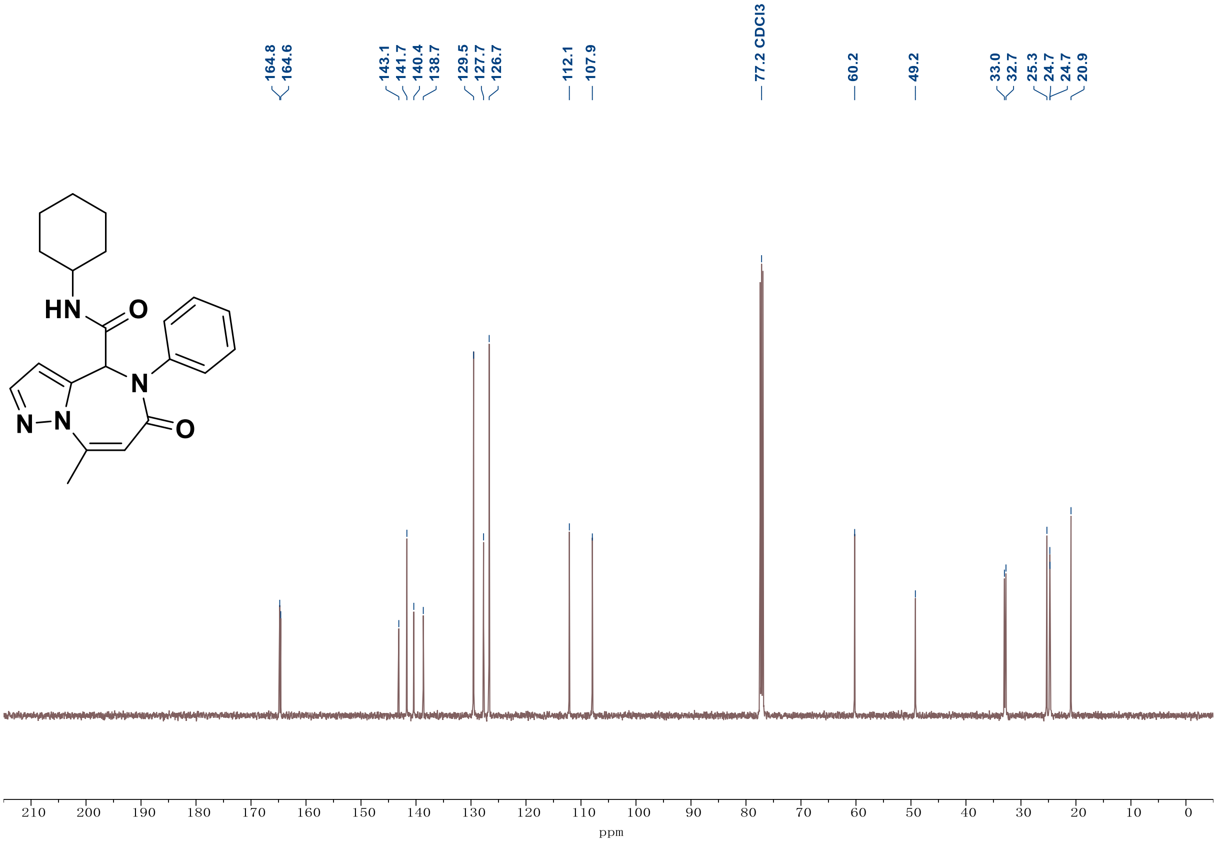

Copies of ^{1}H and $^{13}C\{^{1}H\}$ spectra of compound **16t**

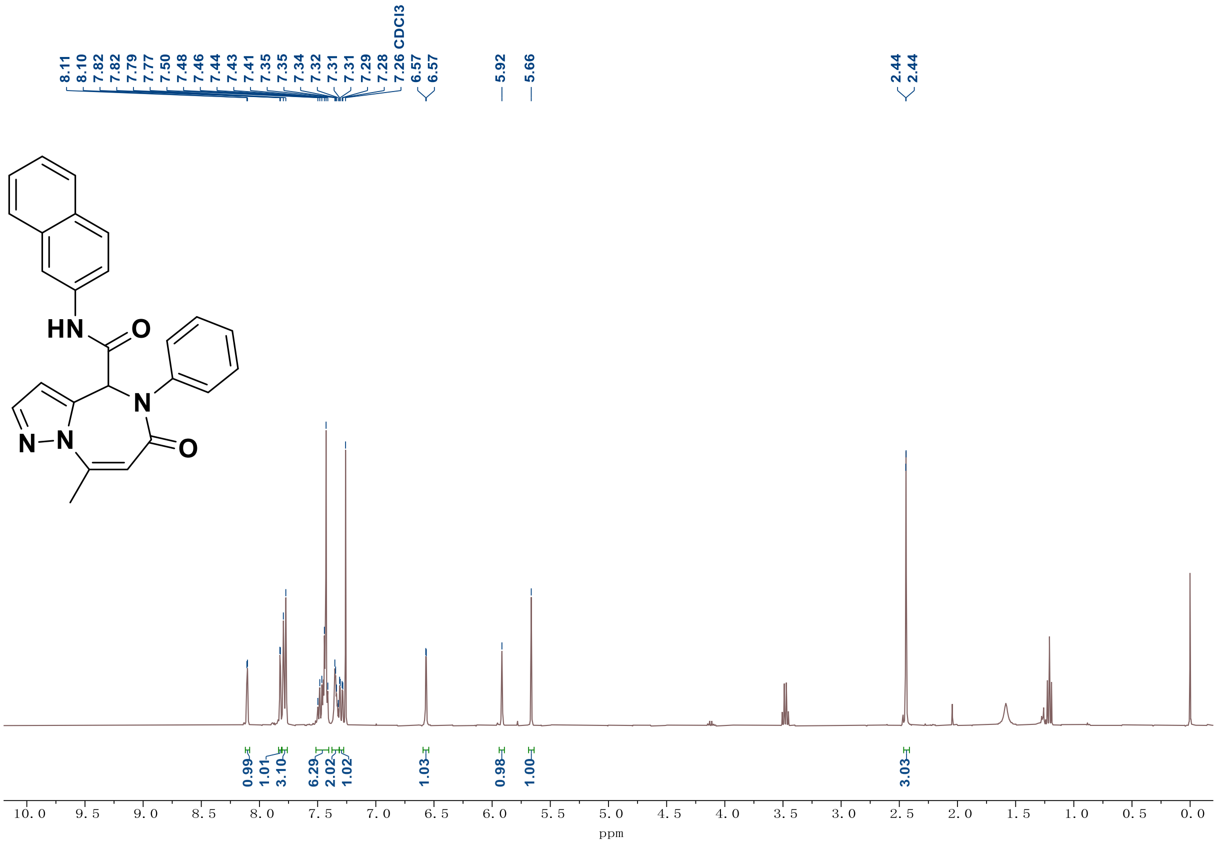

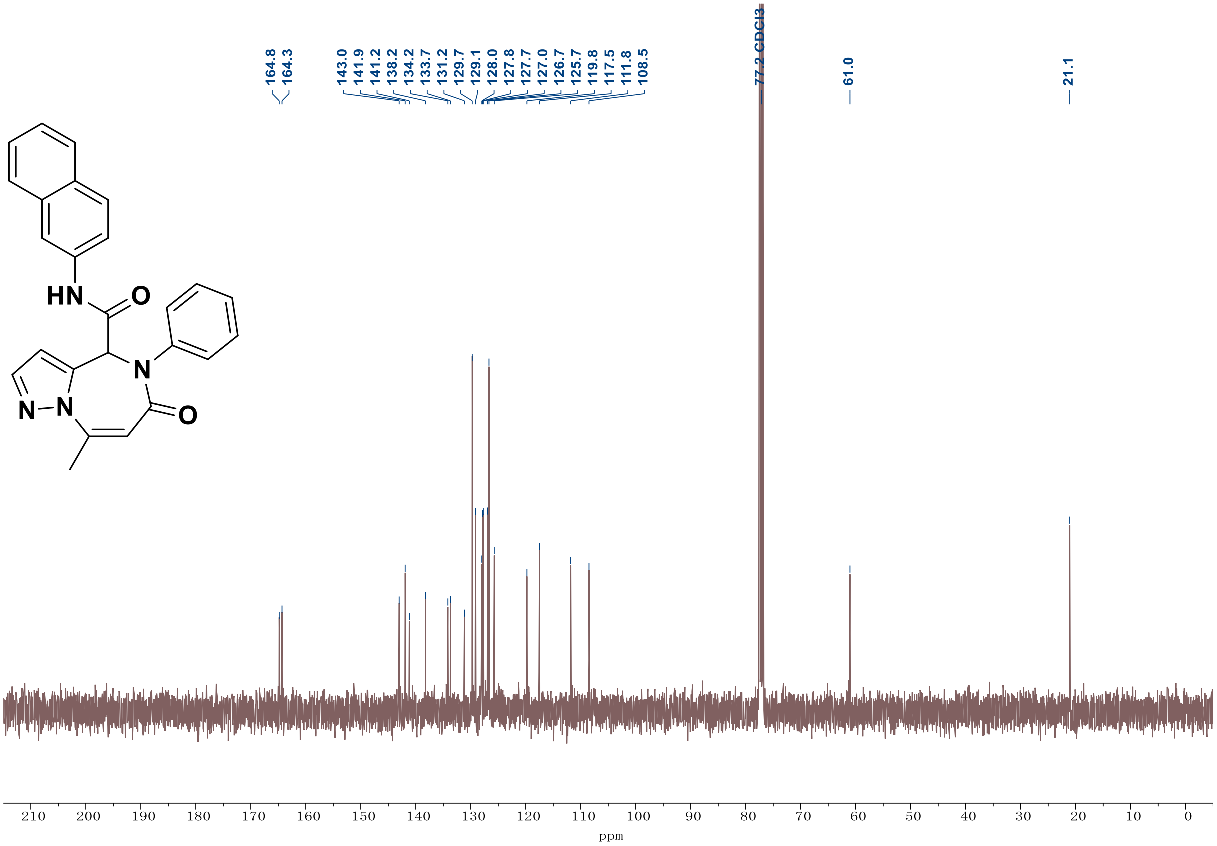

Copies of ^{1}H and ^{13}C{^{1}H} spectra of compound **16u**

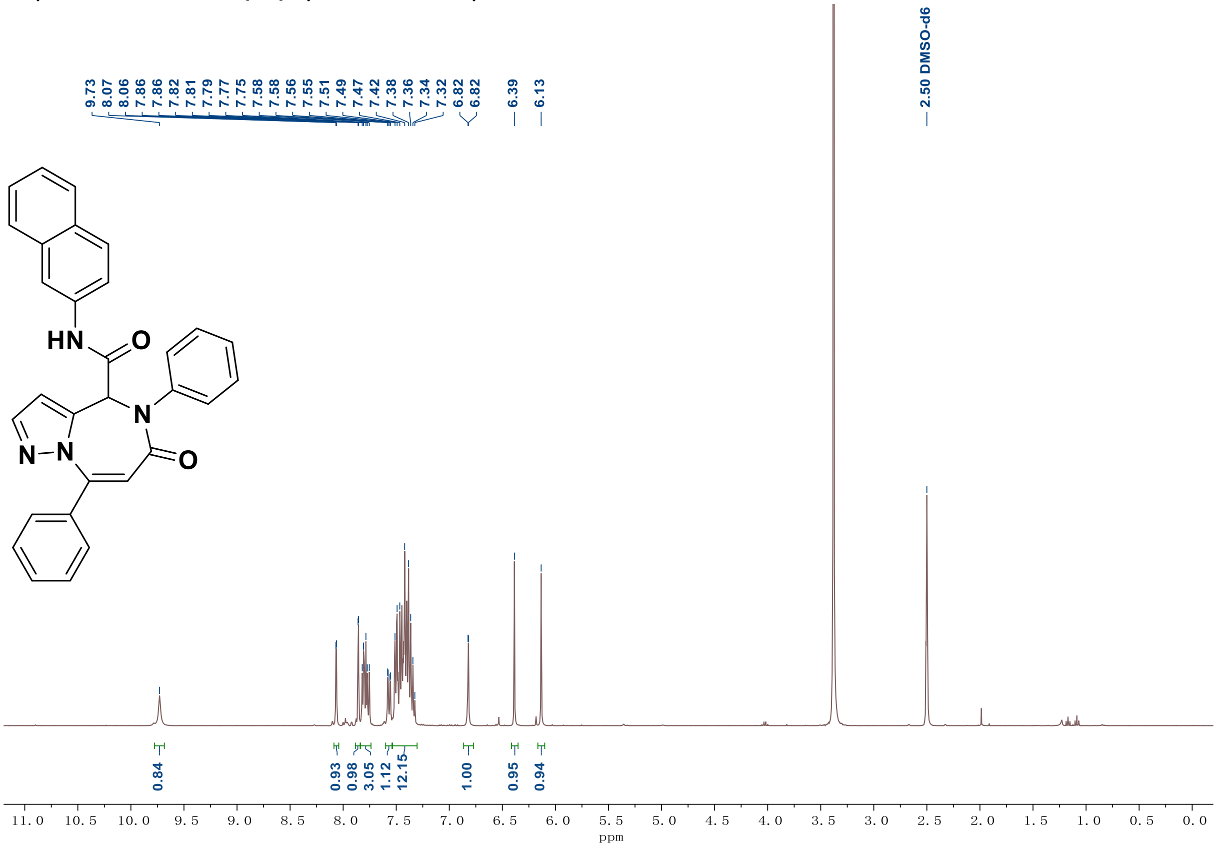

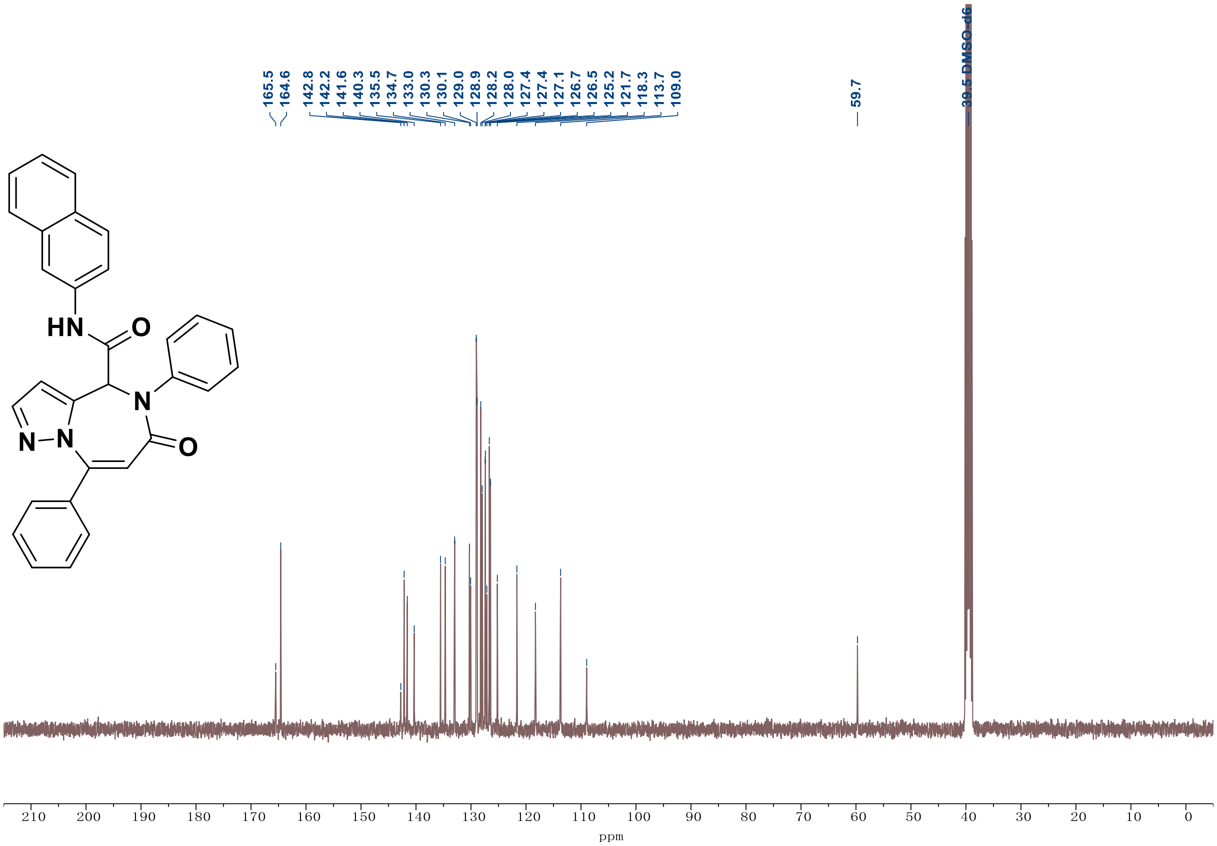

Copies of ^{1}H and $^{13}C\{^{1}H\}$ spectra of compound **16v**

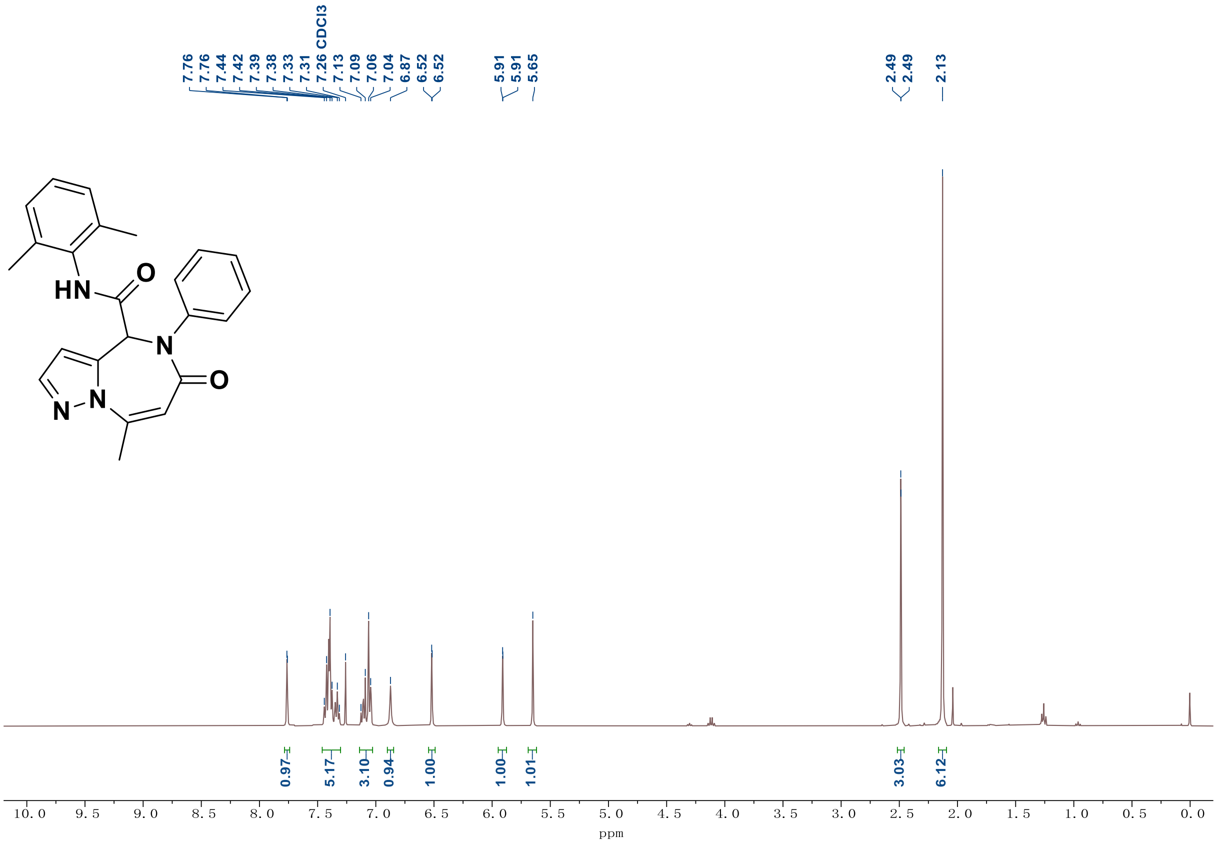

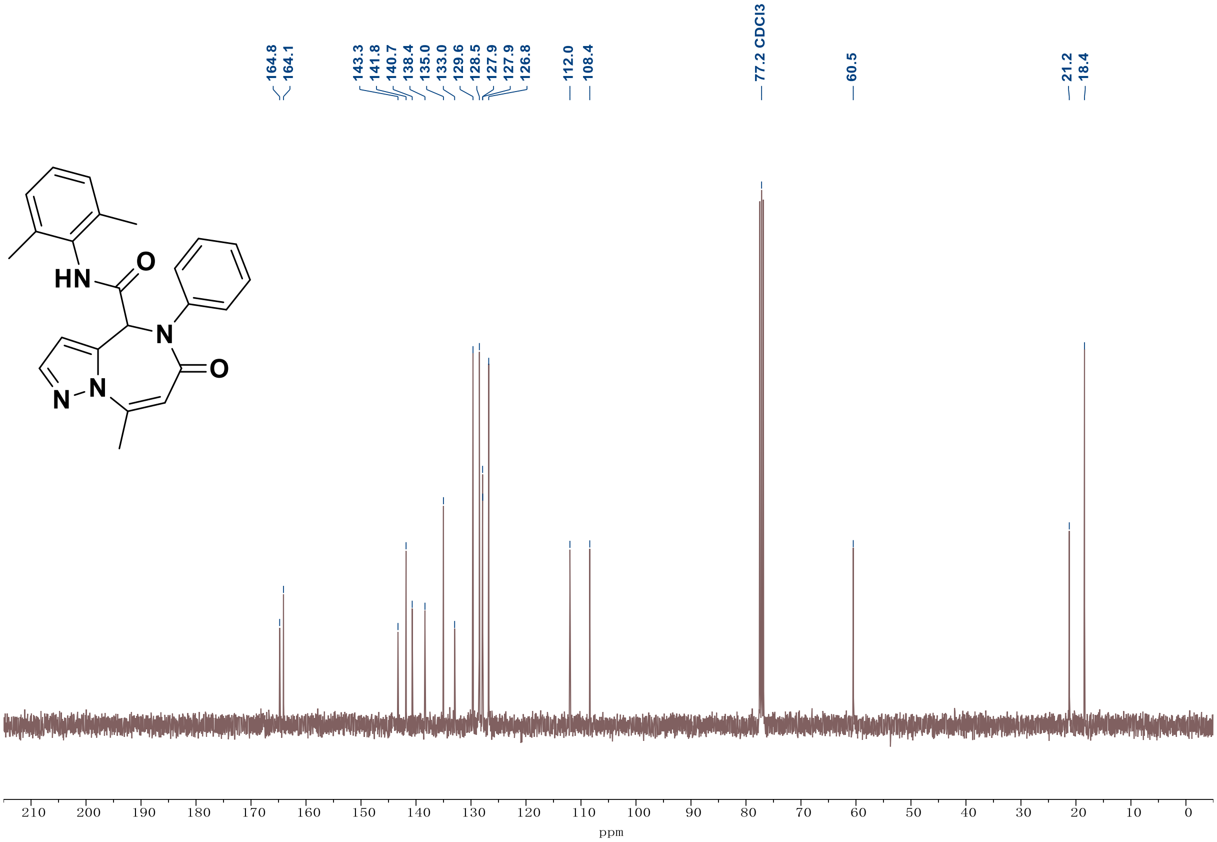

Copies of ^{1}H and ^{13}C{^{1}H} spectra of compound **16w**

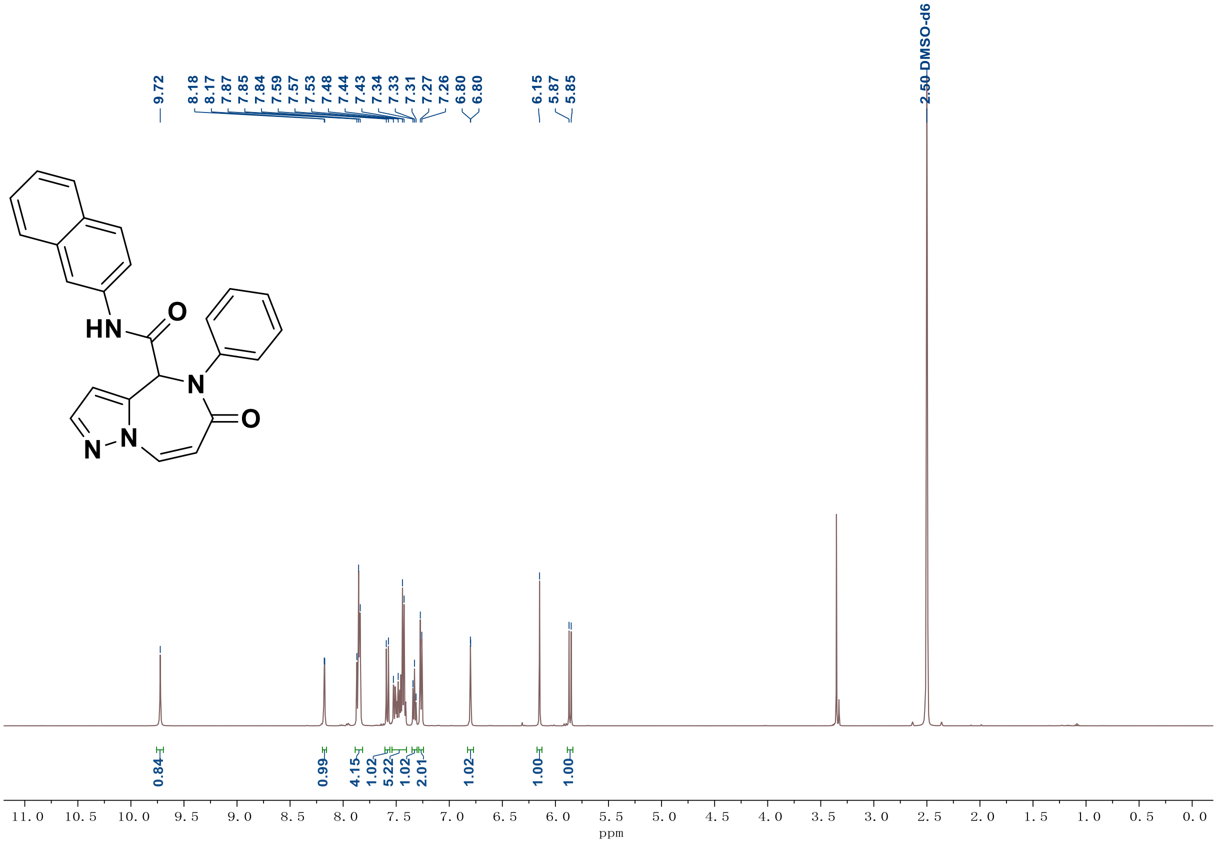

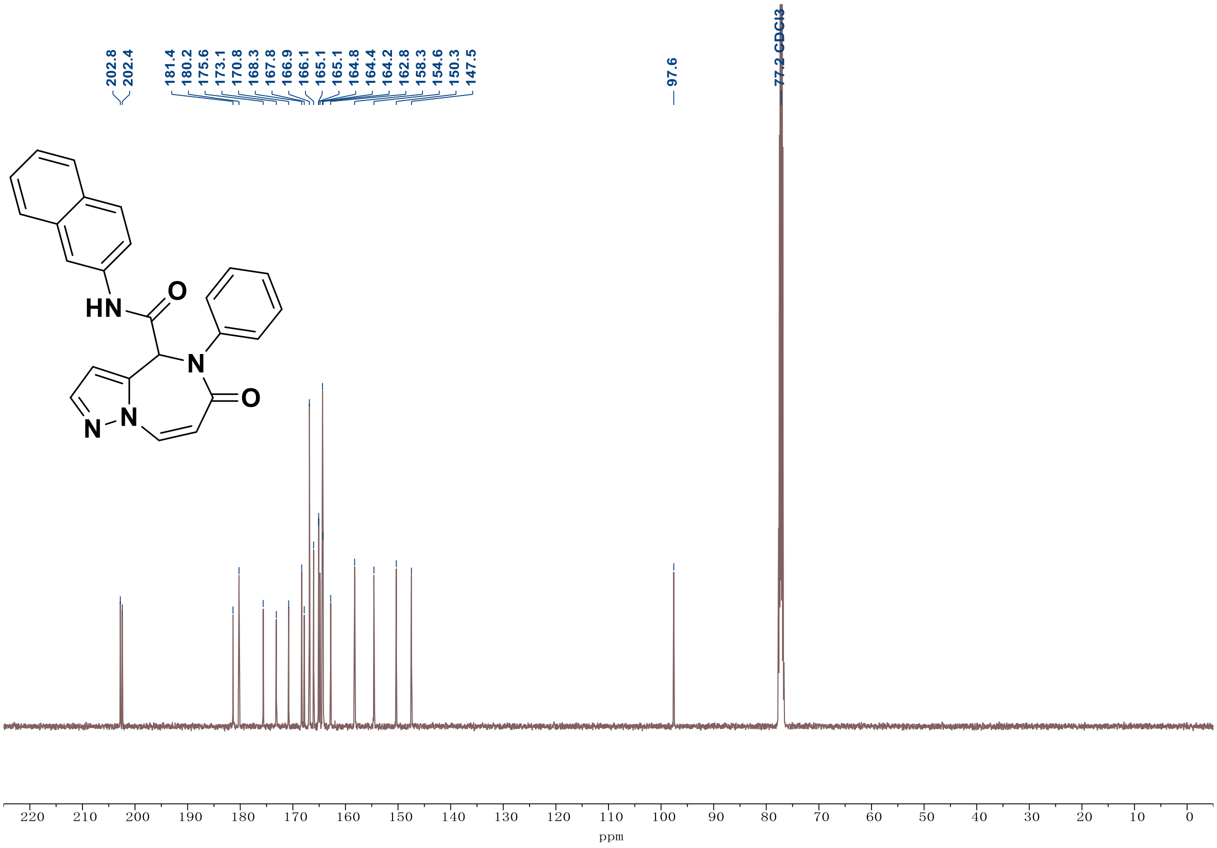

Copies of ^{1}H and $^{13}C\{^{1}H\}$ spectra of compound **16x**

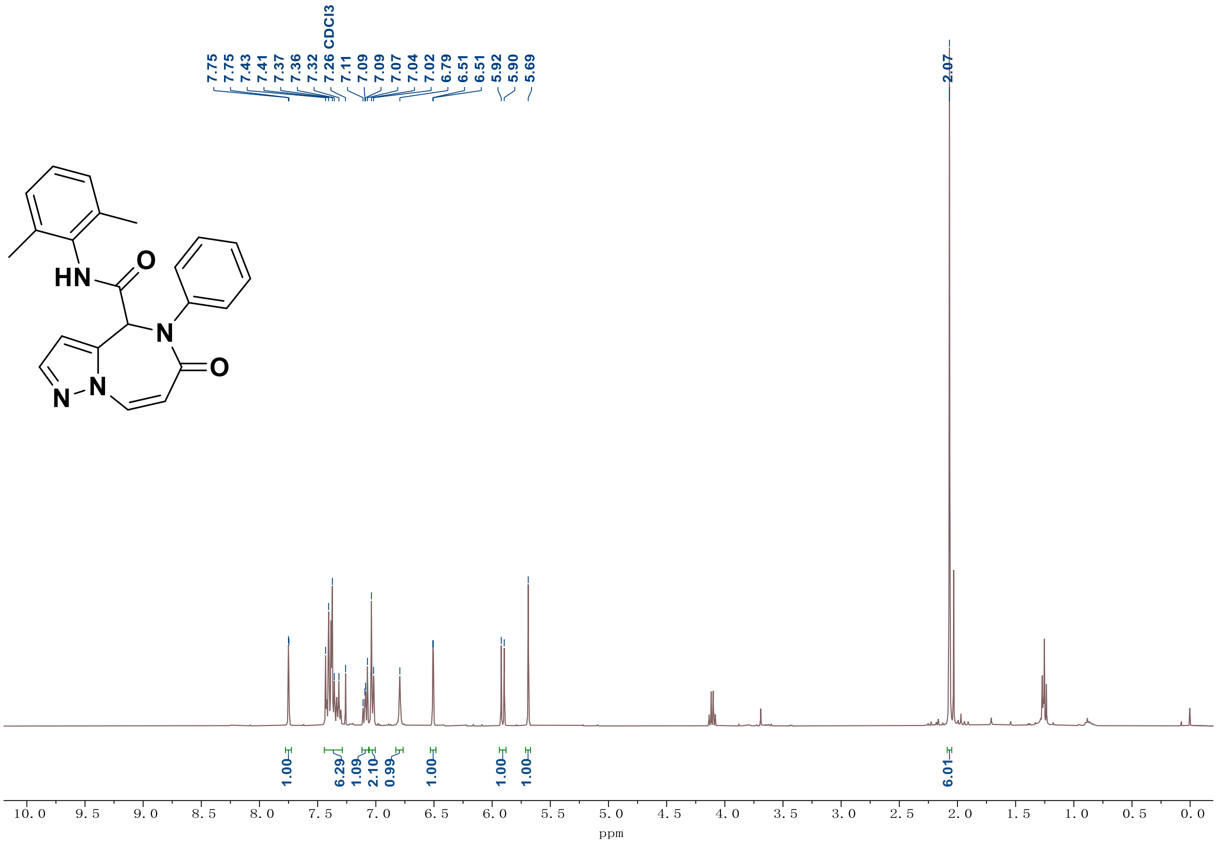

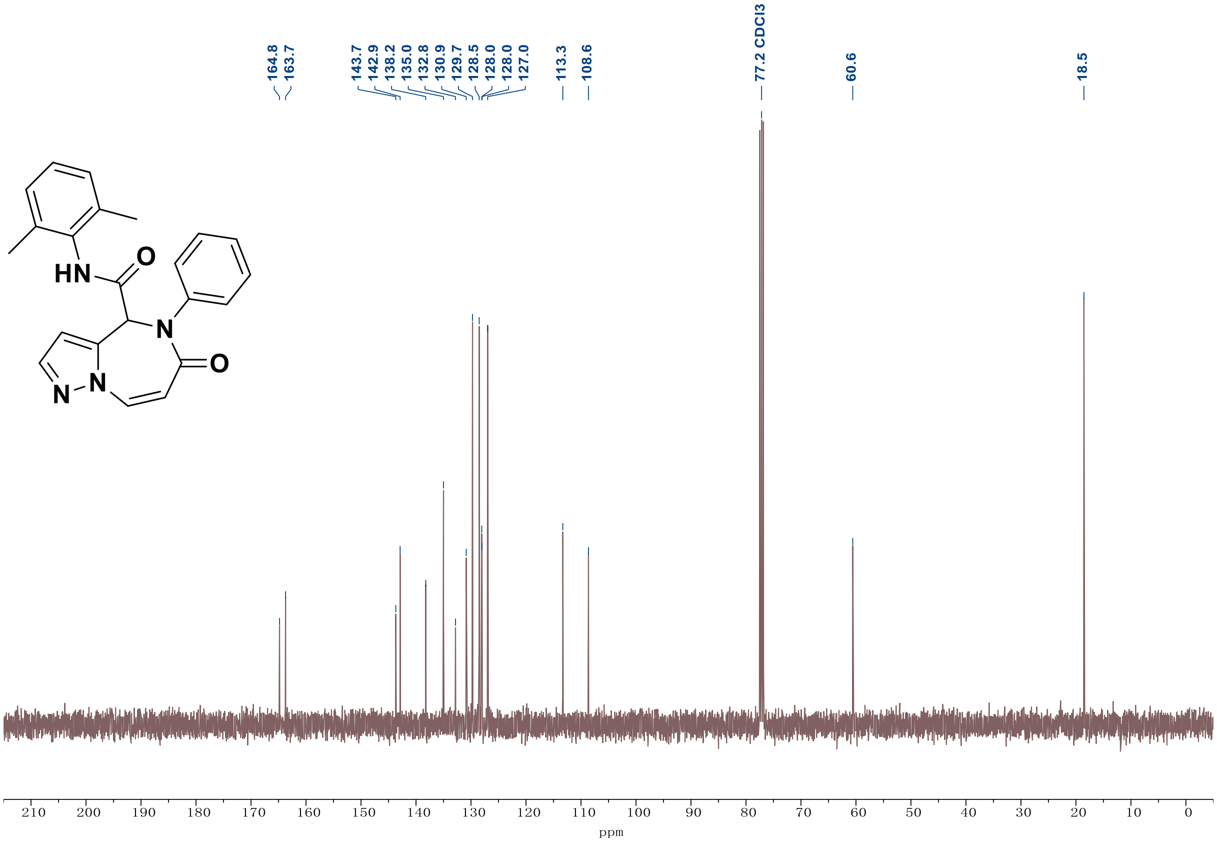

Copies of ^{1}H and $^{13}C\{^{1}H\}$ spectra of compound **17-cis**

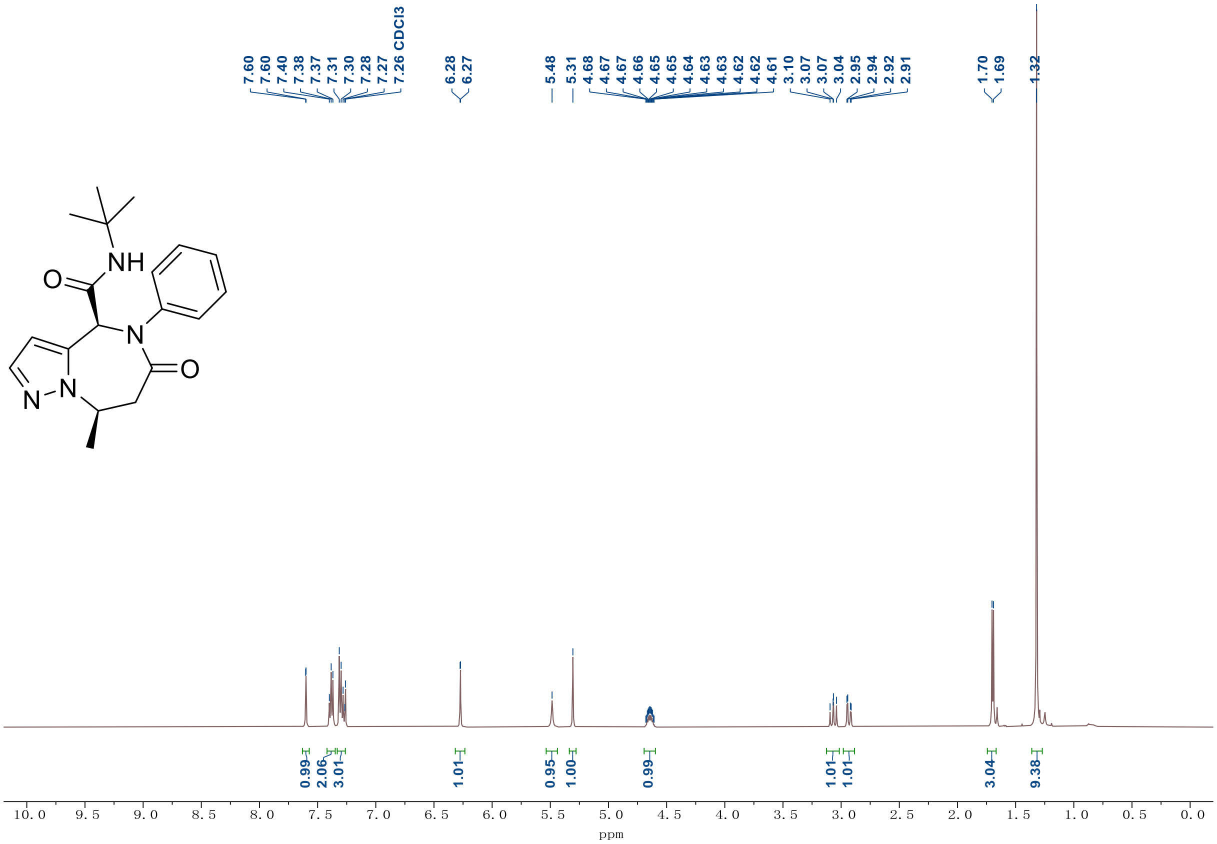

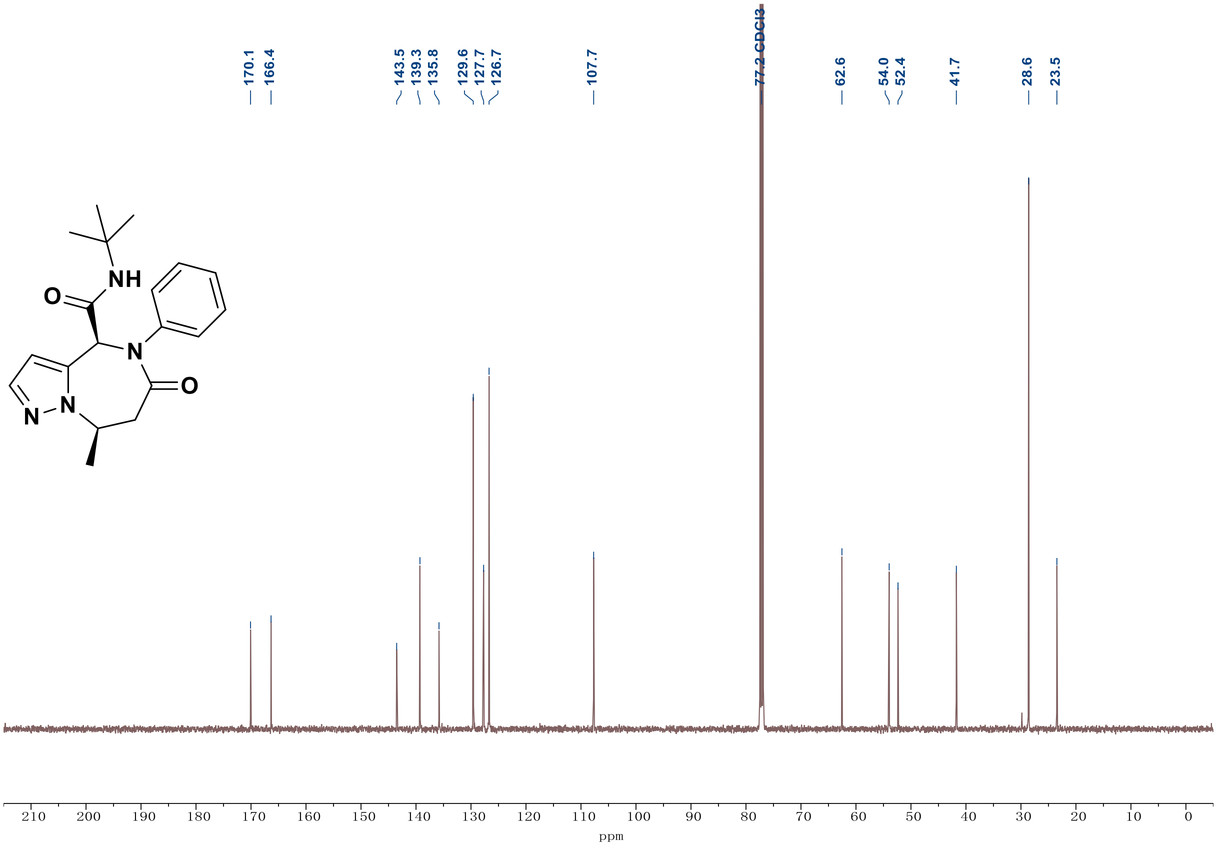

Copies of ^{1}H and ^{13}C{^{1}H} spectra of compound **17-trans**

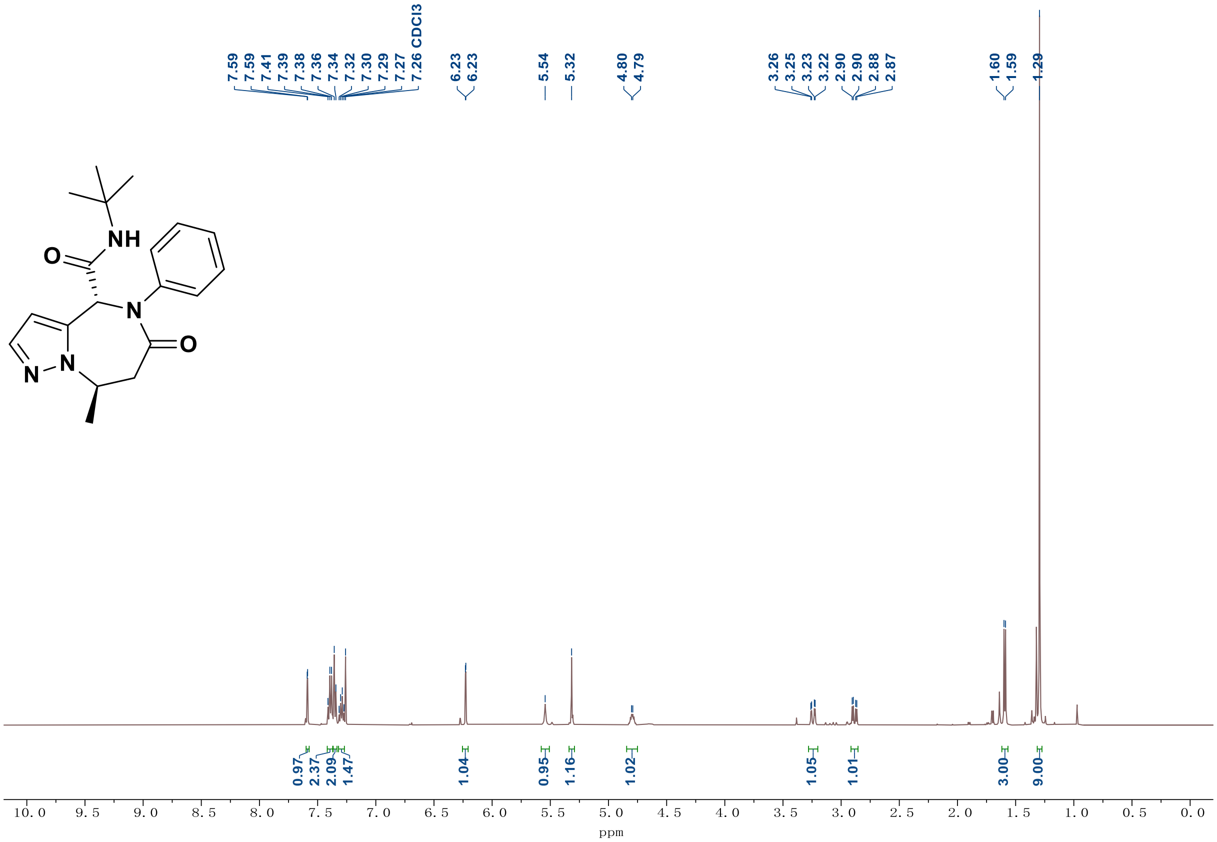

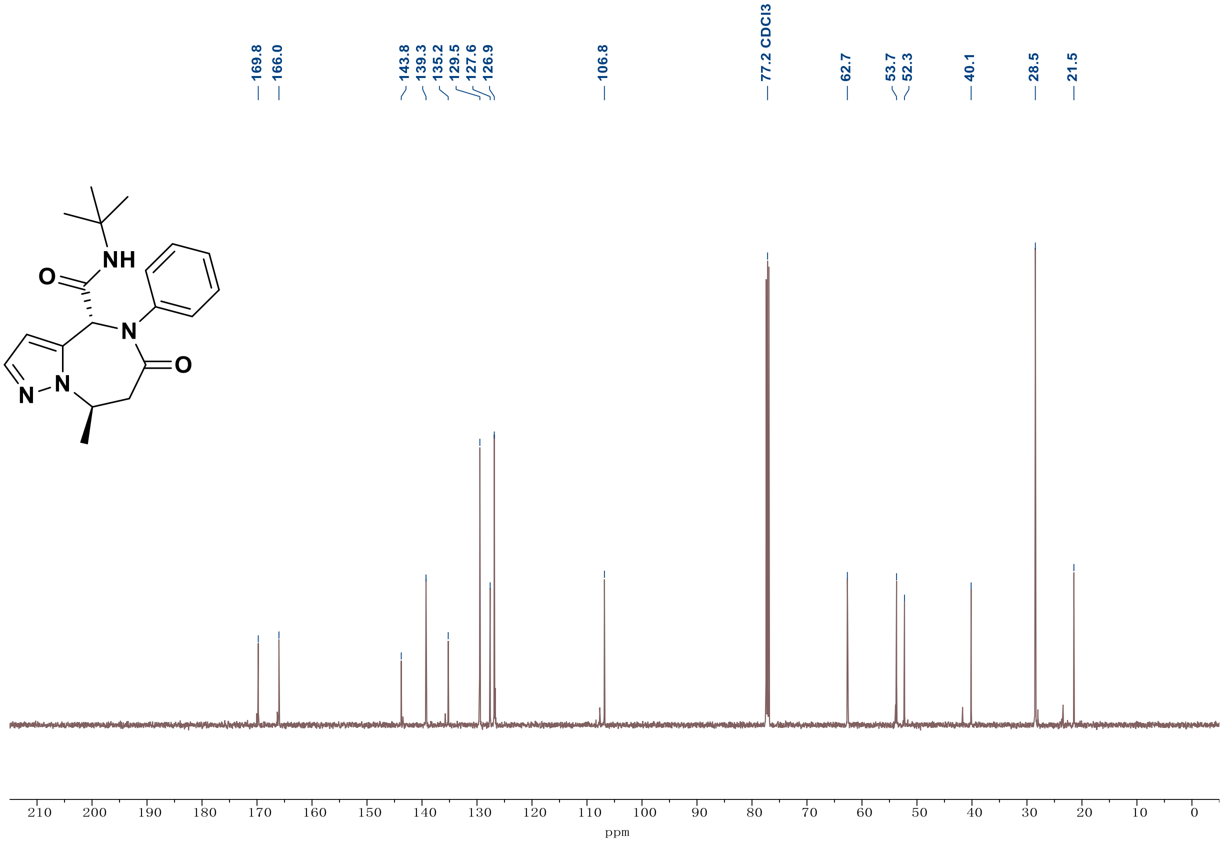

Copies of ^{1}H and $^{13}C\{^{1}H\}$ spectra of compound **18-cis**

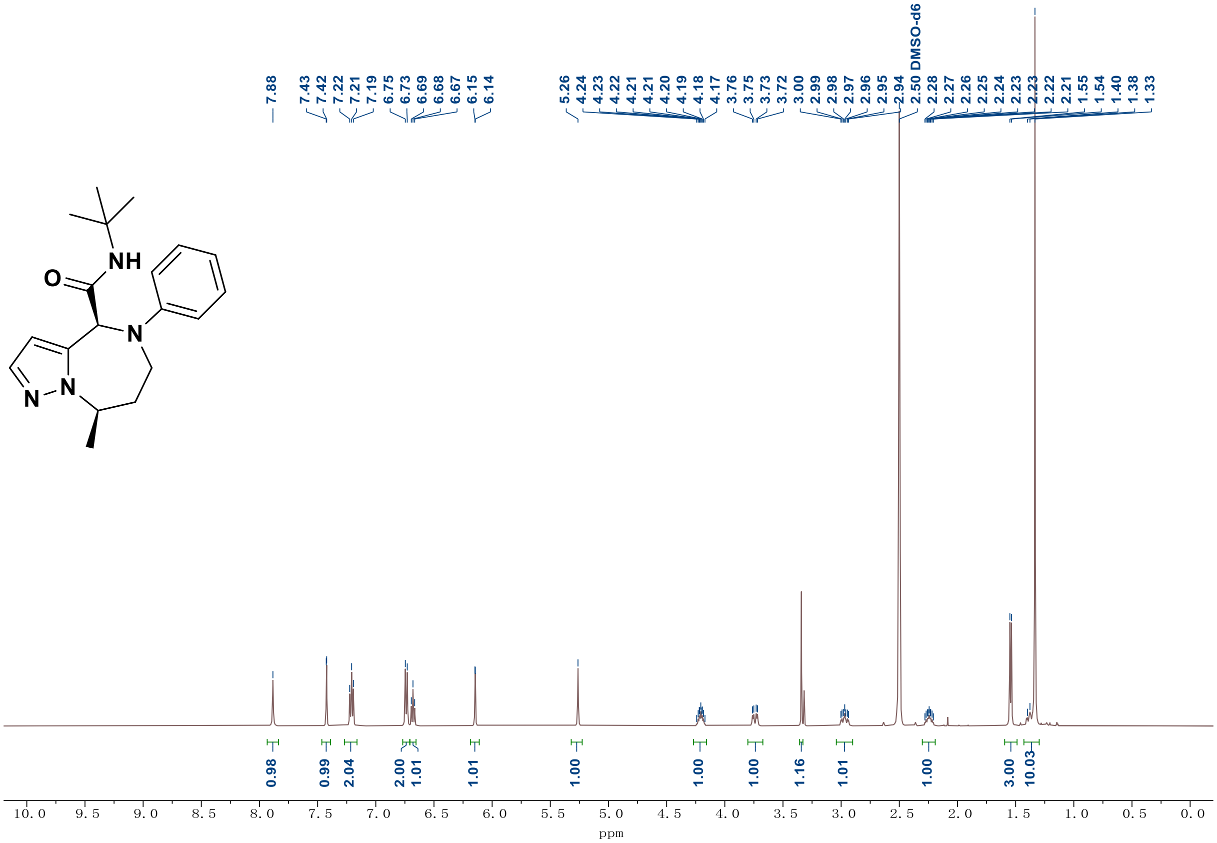

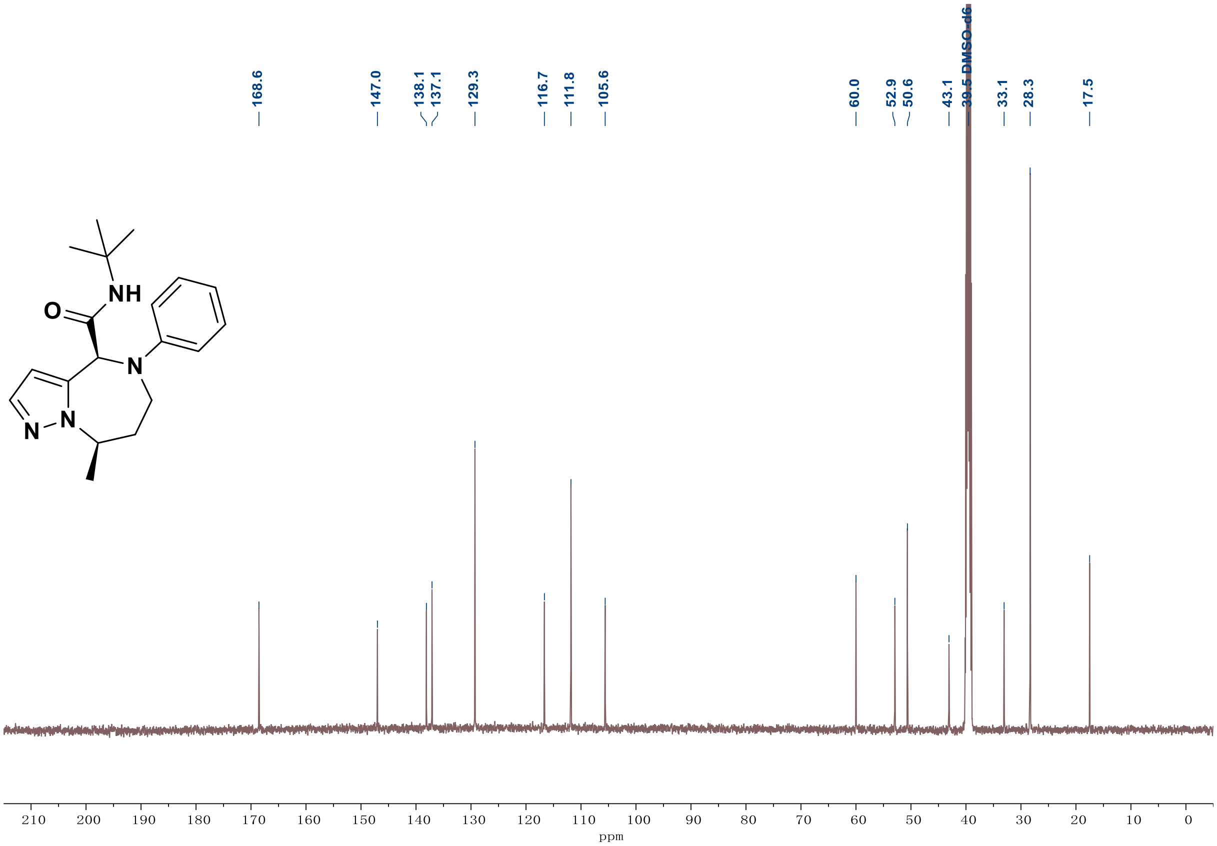

Copies of ^{1}H and $^{13}C\{^{1}H\}$ spectra of compound **18-trans**

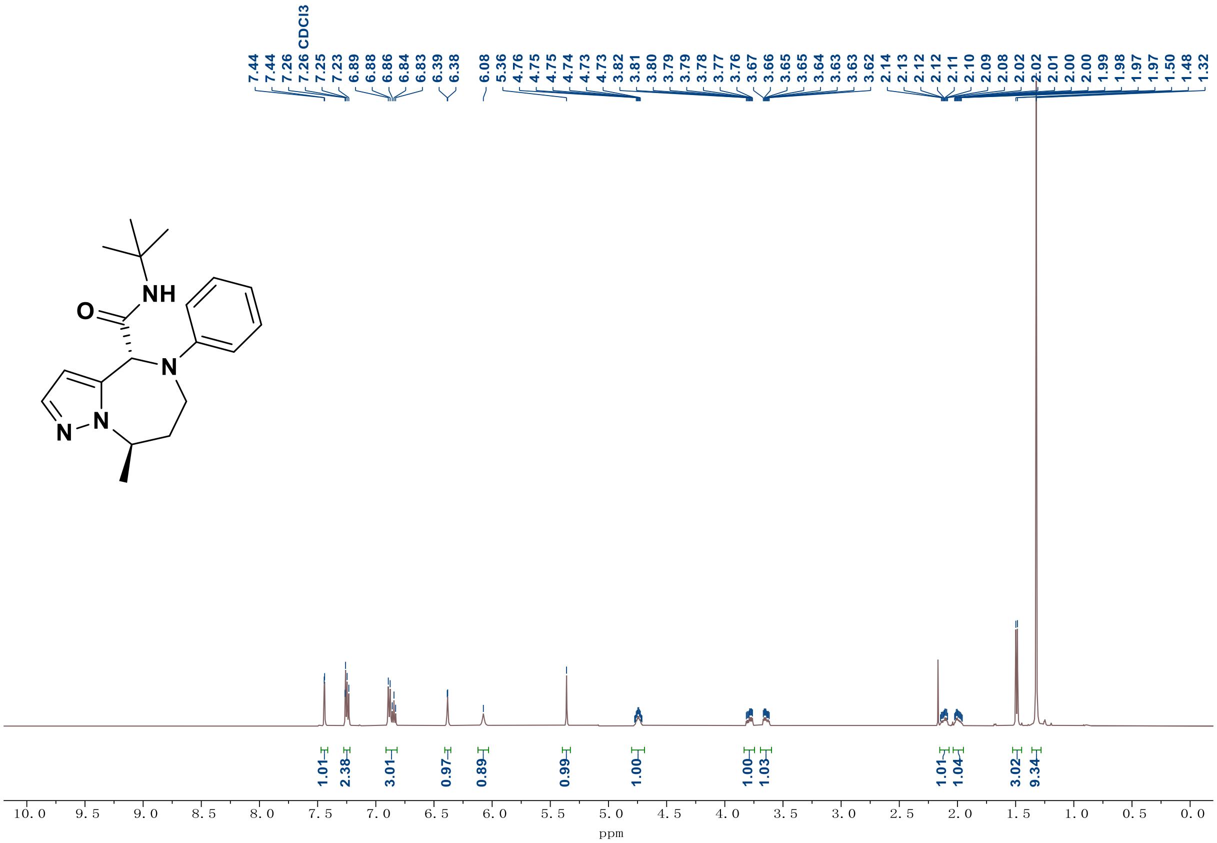

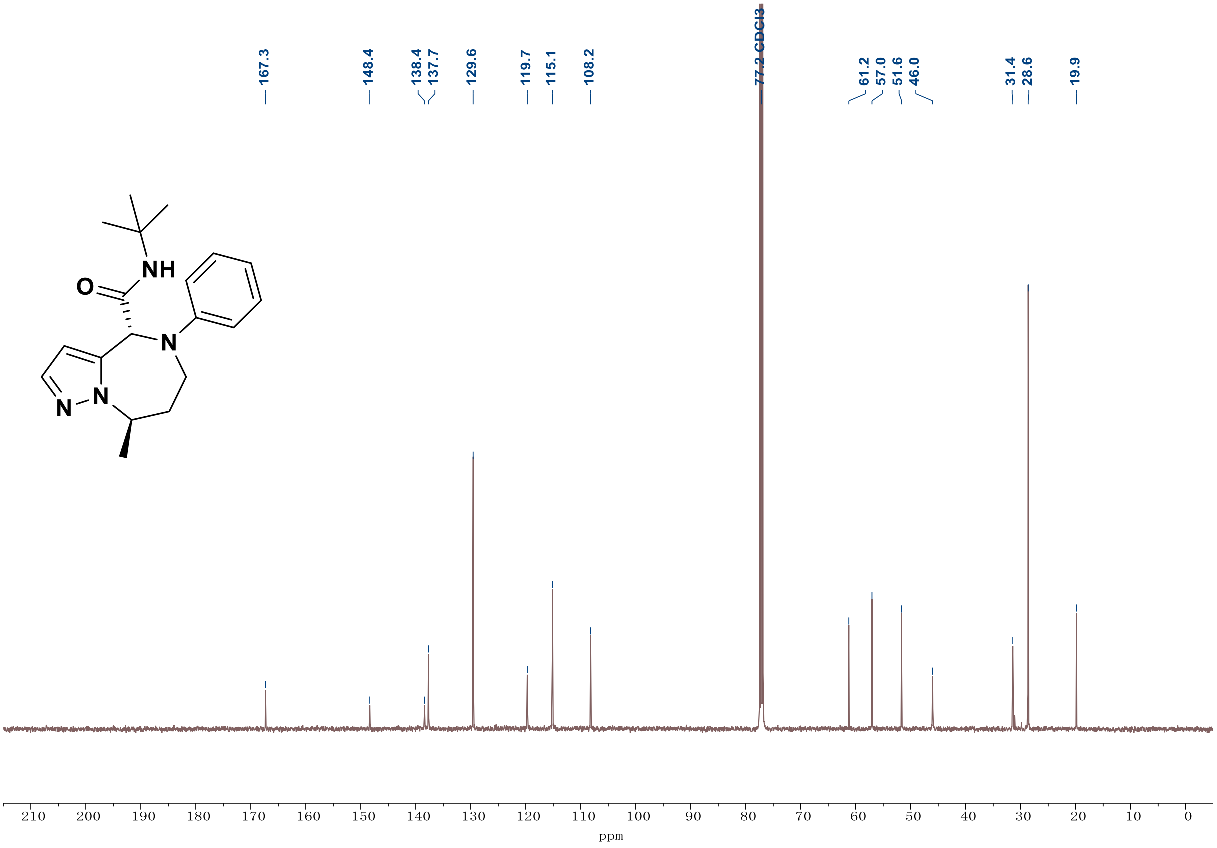

Supplement: File 1 — Detailed descriptions of the experimental procedures, product characterization data and the copies of NMR spectra. [file Beilstein_J_Org_Chem-21-915-s001.pdf]
